# *De novo* transcriptome assembly and analysis of *Phragmites karka*, an invasive halophyte, to study the mechanism of salinity stress tolerance

**DOI:** 10.1038/s41598-020-61857-8

**Published:** 2020-03-23

**Authors:** Soumya Shree Nayak, Seema Pradhan, Dinabandhu Sahoo, Ajay Parida

**Affiliations:** 1 Institute of Life Sciences, NALCO Square, Chandrasekharpur, Bhubaneswar 751023 India; 2 Institute of Bioresources and Sustainable Development, Imphal, Manipur 795001 India

**Keywords:** Molecular biology, Plant sciences

## Abstract

With the rapidly deteriorating environmental conditions, the development of stress tolerant plants has become a priority for sustaining agricultural productivity. Therefore, studying the process of stress tolerance in naturally tolerant species hold significant promise. *Phragmites karka* is an invasive plant species found abundantly in tropical and sub tropical regions, fresh water regions and brackish marshy areas, such as river banks and lake shores. The plant possesses the ability to adapt and survive under conditions of high salinity. We subjected *P. karka* seedlings to salt stress and carried out whole transcriptome profiling of leaf and root tissues. Assessing the global transcriptome changes under salt stress resulted in the identification of several genes that are differentially regulated under stress conditions in root and leaf tissue. A total of 161,403 unigenes were assembled and used as a reference for digital gene expression analysis. A number of key metabolic pathways were found to be over-represented. Digital gene expression analysis was validated using qRT-PCR. In addition, a number of different transcription factor families including WRKY, MYB, CCCH, NAC etc. were differentially expressed under salinity stress. Our data will facilitate further characterisation of genes involved in salinity stress tolerance in *P. karka*. The DEGs from our results are potential candidates for understanding and engineering abiotic stress tolerance in plants.

## Introduction

*Phragmites* is a halophyte grass belonging to the family Poaceae and found in both fresh and saline wetland systems, as well as brackish waters such as river banks and lake shores. Four species of *Phragmites* are found worldwide, namely, *Phragmites australis, P. japonicus, P. karka, and P. mauritianus*^1^*. Phragmites australis*, the most widespread species, is genetically complex with a range of ploidy levels including 2n = 3×, 4×, 8×, 12× ^2^. The plants can grow up to 6 m tall, extending from the littoral zones of lakes, rivers, irrigation canals and fresh water swamps^3^ and are mainly found in temperate climates. In India, these plants are found in Chilika Lake (Odisha), Loktak Lake (Manipur) and Harika Lake (Punjab). *Phragmites australis* is well adapted to a range of salinity, nutrient and hydrological conditions. *Phragmites australis* is known to inhibit growth of other species because its roots and rhizomes form a densely packed matrix and its root produces a toxic acid known as 3, 4, 5-trihydroxybenzoic acid (gallic acid) which disintegrate the structural protein of neighbouring plants^4^.

*Phragmites karka* is useful for biofuel industry^5^ and traditionally, has been used as a remedy for diabetes^6^. It has been reported as a source of food, edible oil and fodder^7^. It is also an excellent stabilizer of eroding river banks as well as a good candidate for phytoremediation*. Phragmites karka* has rapidly invaded north and north-western segments of Chilika lake, from an area of 76.4 sq km in 2000 to 105.1 sq km (2010). The species can tolerate salinity levels up to 18ppt and has developed different mechanisms for salt tolerance, including compartmentalization of Na^+^ in specific tissues, cells or cellular organelles, and exclusion of Na^+^ from the sensitive shoot tissue^8^. One of the reasons for invasiveness of *P. karka* is its ability to tolerate higher levels of salinity than associated species, which inhibits the growth and development of other plants by hindering various metabolic activities, cell expansion and by triggering programmed cell death^9^. This species disturbs the environment because of its ability to spread vegetatively by a vigorous system of rhizome and stolons and through seeds by establishing new plants in area free of vegetation.

Increased soil salinity has become one of the leading causes for crop yield loss in recent years^10^. Bringing back salinity stress affected land area to productive use would require development of genotypes offering tolerance/resistance to stress conditions and possibly involve identification of novel genetic combinations from naturally stress tolerant systems. Various metabolic pathways like accumulation of osmolytes, antioxidant enzymes and the genes involved in stress response like ion transporters, ion channels, and transcription factors have been utilised for the production of transgenic crops with an improved level of salinity tolerance^11^. But many queries still remain regarding the mechanism of stress tolerance in plants. Conventionally, model organisms, due to their well characterised genomes and availability of genomic resources, have been used to address these queries. However, it is hypothesized that genetic and genomic analysis of halophytes, such as *P. karka* may lead to identification of novel metabolic pathways, mechanisms and genes involved in modulating salinity stress tolerance in crop plants^12^.

To identify differentially expressed genes in *P. karka* associated with salinity stress we utilized next generation sequencing technology, which provides a high-throughput, rapid and cost-effective means to sequence and characterize the transcriptome of non-model species. Many studies have been conducted to explore the mechanisms responsible for salt stress tolerance in a variety of plant species. The pathways and genes that are involved in salt tolerance have been reported in model plants like *Arabidopsis* and rice^13^.The present communication reports a comprehensive RNASeq-based transcriptomic analysis of tissue samples (root and leaf) of *P. karka* exposed to different salinity treatments, and the validation of digital expression analysis by qRT PCR. SSR markers, due to their widespread distribution and high reproducibility, have long been utilised for marker assisted selection for plant breeding^14^. They have also proved to be valuable for assessing genetic diversity. Therefore, this study also reports identification of SSRs in the *P. karka* transcriptome.

## Results

### High throughput sequencing, assembly and quality assessment

In order to analyse the effect of salinity stress at the molecular level, eight-paired end libraries were generated for leaf and root tissue samples of *Phragmites karka* in replicates. The leaf and root tissues treated with 0 mM NaCl were taken as control (CLSS1, CLSS2, CRSS1, CRSS2) and those treated with 150 mM NaCl were designated as treated samples (LSS1, LSS2, RSS1, RSS2). A total of 165,021,860 clean reads were generated from the RNA-seq of eight samples with an average of 20.6 million reads per sample. The reads were then assembled using BinPacker (http://sourceforge.net/projects/transcriptomeassembly/files/BinPacker_1.0.tar.gz/download) and rnaSpades (cab.spbu.ru/software/rnaspades/). Multiple assemblers were used to ensure representation of all possible transcripts. The individual assemblies were merged and redundant sequences were removed to generate the final assembly of 161,403 unigenes (Table 1) incorporating 218,566,080 bases. The N50 value for the assembly was 1969, with the average length of transcript being 1354.16 bp. The raw reads have been submitted to NCBI bearing GenBank BioProject Accession number PRJNA554019.

**Table 1:** Assembly statistics for *P. karka* transcriptome.

Attributes	Value
Total number of contigs	161,403
Total count of bases	218566080
N50 value	1969
Length of largest contig	29785
No. of Contigs upto 500 bp in length	39350
No. of Contigs of size between 501 and 3000 bp	107727
No. of Contigs of size between 3001 and 4000 bp	7960
No. of Contigs of size between 4001 and 5000 bp	3297
No. of Contigs of size greater than 5000 bp	3069

BUSCO (Benchmarking Universal Single-Copy Orthologs) has been widely acknowledged as a standard for testing the completeness of an assembly^15^ and was used to determine the transcriptome assembly. A total of 2772 (84.6%) complete BUSCOs were identified in *P. karka* transcriptome, out of which 1701 (51.9%) were single-copy BUSCOs and 1071 (32.7%) were duplicated BUSCOs (Fig. S1). Prediction of long ORFs revealed that on an average, 74% of the unigenes coded for long ORFs and hence, validates the assembly parameters.

### Functional annotation

**Figure 1:**
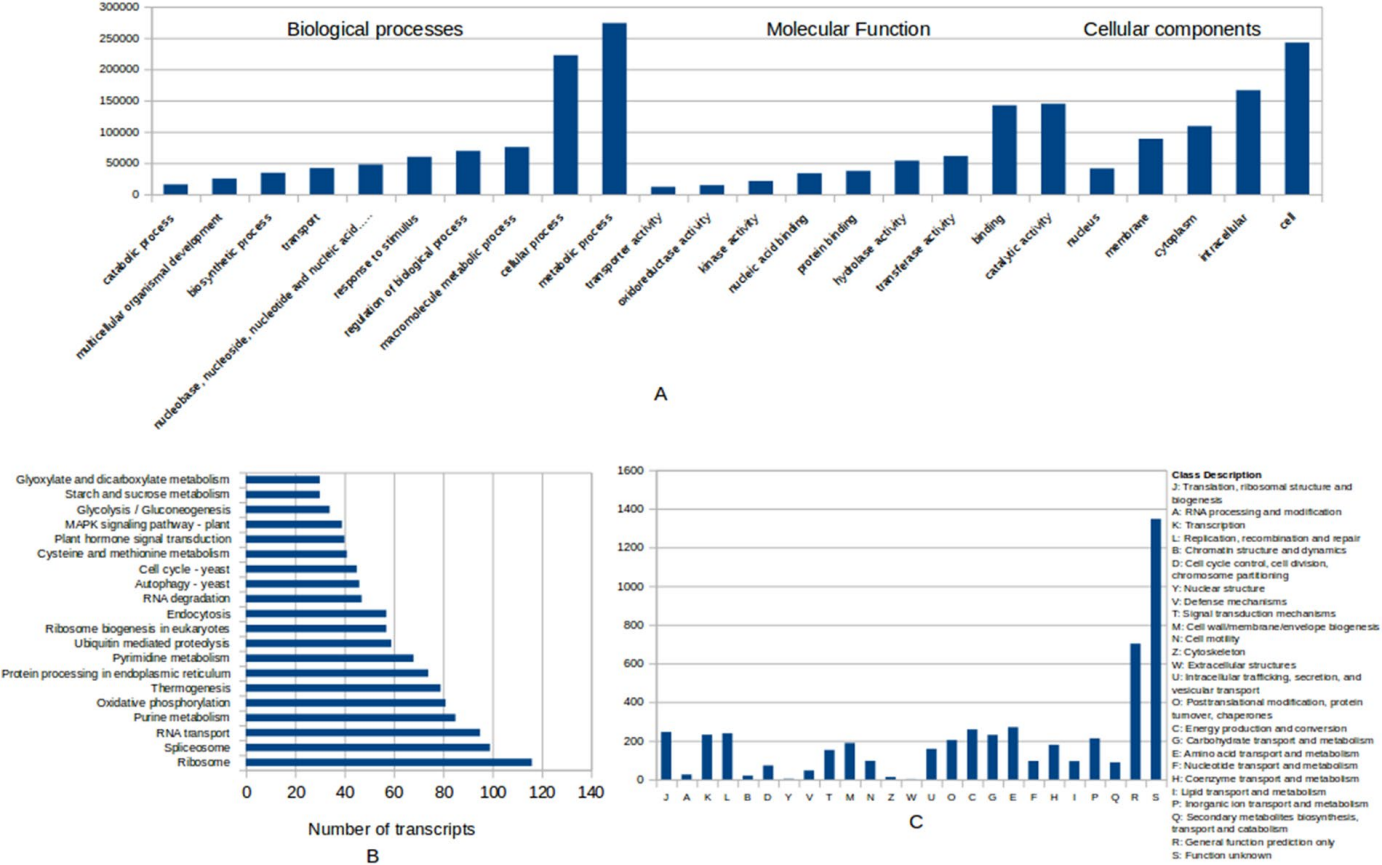
Functional annotation of unigenes: (**A**) Assignment of GO Slim terms to unigenes after comparison with Uniprot-Swiss Prot database (**B**) Distribution of unigenes into biological pathways in KEGG database (**C**) Annotation of unigenes with COG database.

Functional annotation of the unigenes was carried out using three different databases to decipher the general profile related to the biological functions represented in the transcriptome of *P. karka*. Gene Ontology (GO) terms were assigned after BLASTX search against the Uniprot Swissprot database and the unigenes were classified into Biological processes, Molecular functions and Cellular components. It was observed that the categories “metabolic process”, “binding” and “catalytic activity” were over-represented (Fig. 1A). The unigenes were distributed into biological pathways based on their homology to the enzymes in KEGG Automatic Annotation Server (KAAS) database (https://www.genome.jp/kegg/kaas/). Majority of the unigenes were classified into “Ribosome”, “Spliceosome” and “RNA transport” categories. In addition, categories like “Ubiquitin mediated proteolysis”, “MAPK signalling pathway”, “Oxidative phosphorylation” and “Plant hormone signal transduction” were also enriched in the transcriptome (Fig. 1B). A comparison with the COG database (http://weizhong-lab.ucsd.edu/webMGA/server/cog/) showed that the most highly represented categories were “General function prediction only” and “Function unknown” along with unigenes related to “Translation, ribosomal structure and biogenesis”, “Transcription” and “Amino acid transport and metabolism” (Fig. 1C).

### Global analysis of differentially expressed unigenes

**Figure 2:**
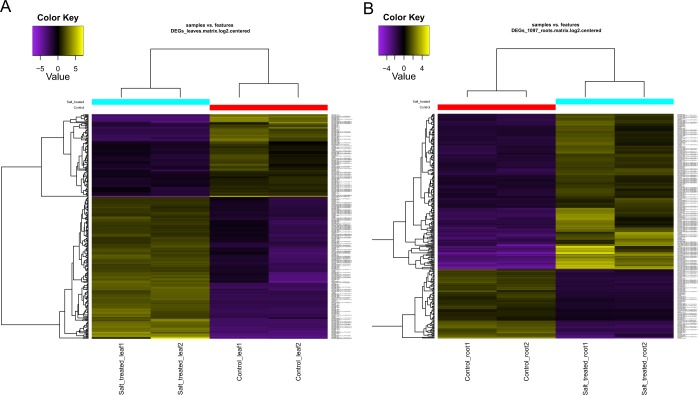
*In silico* analysis of differential expression of unigenes in (**A**) Leaves and (**B**) Root tissues subjected to salinity stresses. The set of perl scripts included in the Trinity v. 2.8.5^58^ was used to generate the respective heat maps. Detailed description for usage of the scripts can be found at https://github.com/trinityrnaseq/trinityrnaseq/wiki/Trinity-Differential-Expression.

Digital gene expression analysis is a convenient method to screen the large number of unigenes for those that are differentially expressed. The process requires mapping of raw reads onto the assembled transcriptome to determine transcript abundance and the data is subsequently normalised to generate a non-biased view of the differentially expressed genes (DEGs). The same was carried out for determining the DEGs in *P. karka* transcriptomes after exposure to high salinity. Leaf and root tissues were analysed separately to determine tissue-specific DEGs as well as those common to both tissues during salinity stress. In leaves, 954 unigenes were differentially regulated and after normalisation using edgeR^16^, 305 DEGs were seen to have significant differential expression with a 4-fold change in levels of expression (Fig. 2A). The majority of unigenes were upregulated in response to salinity stress and included transcription factors like Ethylene responsive transcription factor (ERF), MYB, C2CH Zn finger, FAR1 and AP2/ERF. A number of stress responsive genes such as heat shock proteins, chaperones, glutathione S transferase, genes for the 26 S proteasome pathway were detected. In addition, a number of sugar, calcium and ion transporters and ribosomal proteins were also identified amongst the unigenes with a significant level of differential expression (Table S1).

In roots, 1097 unigenes were significantly differentially expressed and the pattern of gene expression was similar to that observed in leaves i.e. most of the genes were upregulated in response to salinity stress (Fig. 2B). Out of the 1097 DEGs, 289 were expressed at 4 fold level or more and were analysed further for identifying important genes. BLASTx search against the Uniprot SwissProt database showed that TFs like ERF, NAC, WRKY, CCCH and MYB along with genes like ribosomal proteins, kinases, a number of various transporters like polyol transporters, ion transporters and antioxidants are expressed differentially and therefore, could be crucial to salinity stress tolerance (Table S2).

### DEGs common to root and leaf tissue

**Figure 3:**
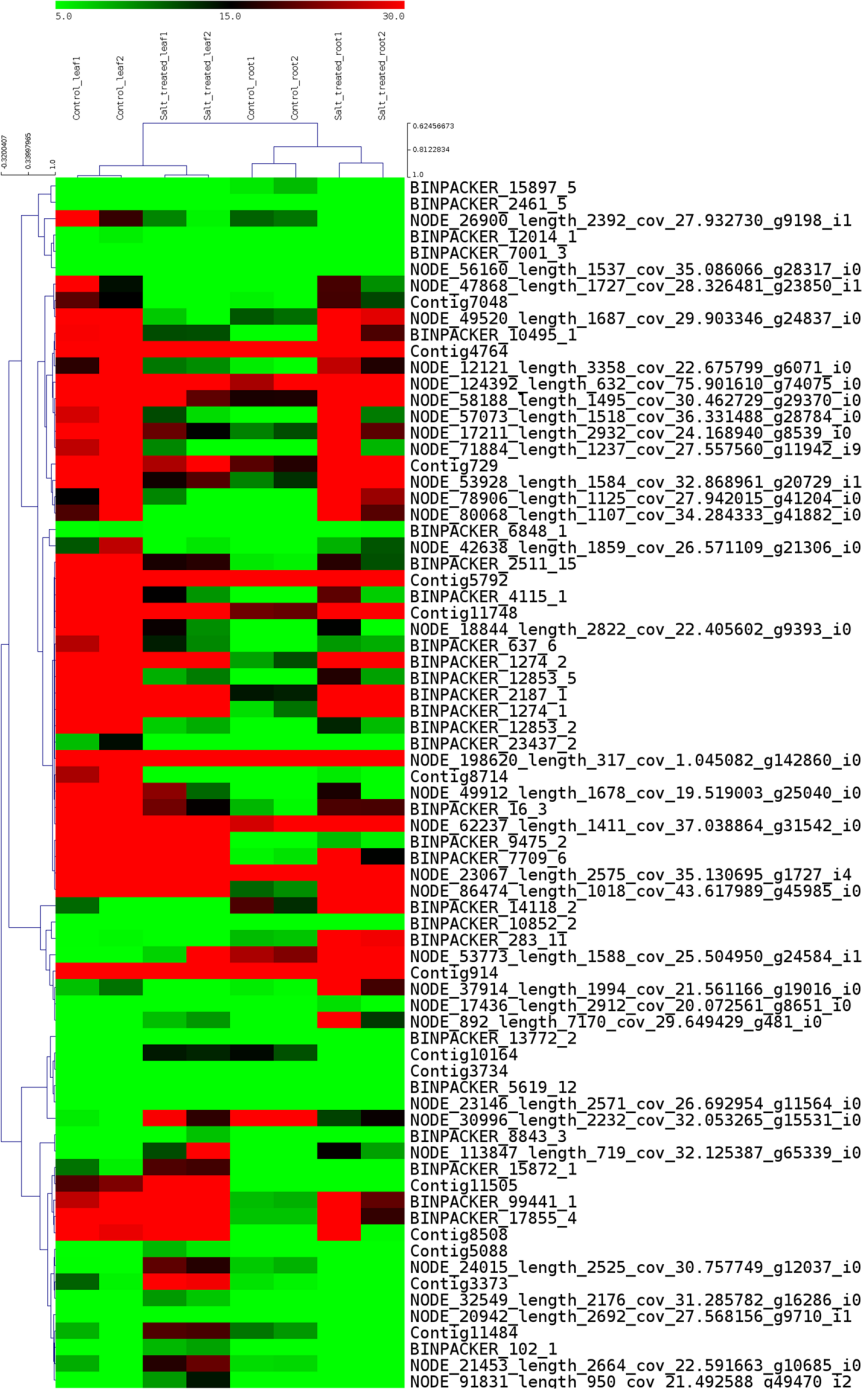
Differential expression of 74 DEGs common to leaf and root tissue in *P. karka* during exposure to salinity stress. Heat map was drawn using MeV v. 4.8.1 (https://sourceforge.net/projects/mev-tm4/files/).

A total of 74 DEGs were found to be common to both leaves and roots (Fig. 3). These included gene encoding 40 S ribosomal protein, CSC1- like protein, MYB related protein, LYR motif, Ethylene responsive factor, Hexokinase, Cysteine rich repeat which were found to be are down regulated in leaf tissue under salinity stress while Cellulose synthase is up regulated (Table S3). Genes encoding MYB related protein, LYR motif containing, Methylene blue sensitivity, Hexokinase-7, EFR, Cysteine rich secretory protein, Caffeolylshikimate esterase, Alanine glyoxylate aminotransferase, Pheophorbide-a-oxygenase are highly upregulated in root tissue during exposure to salinity stress (Table S3).

### Transcription factors in *P. karka* for salinity stress tolerance

**Figure 4:**
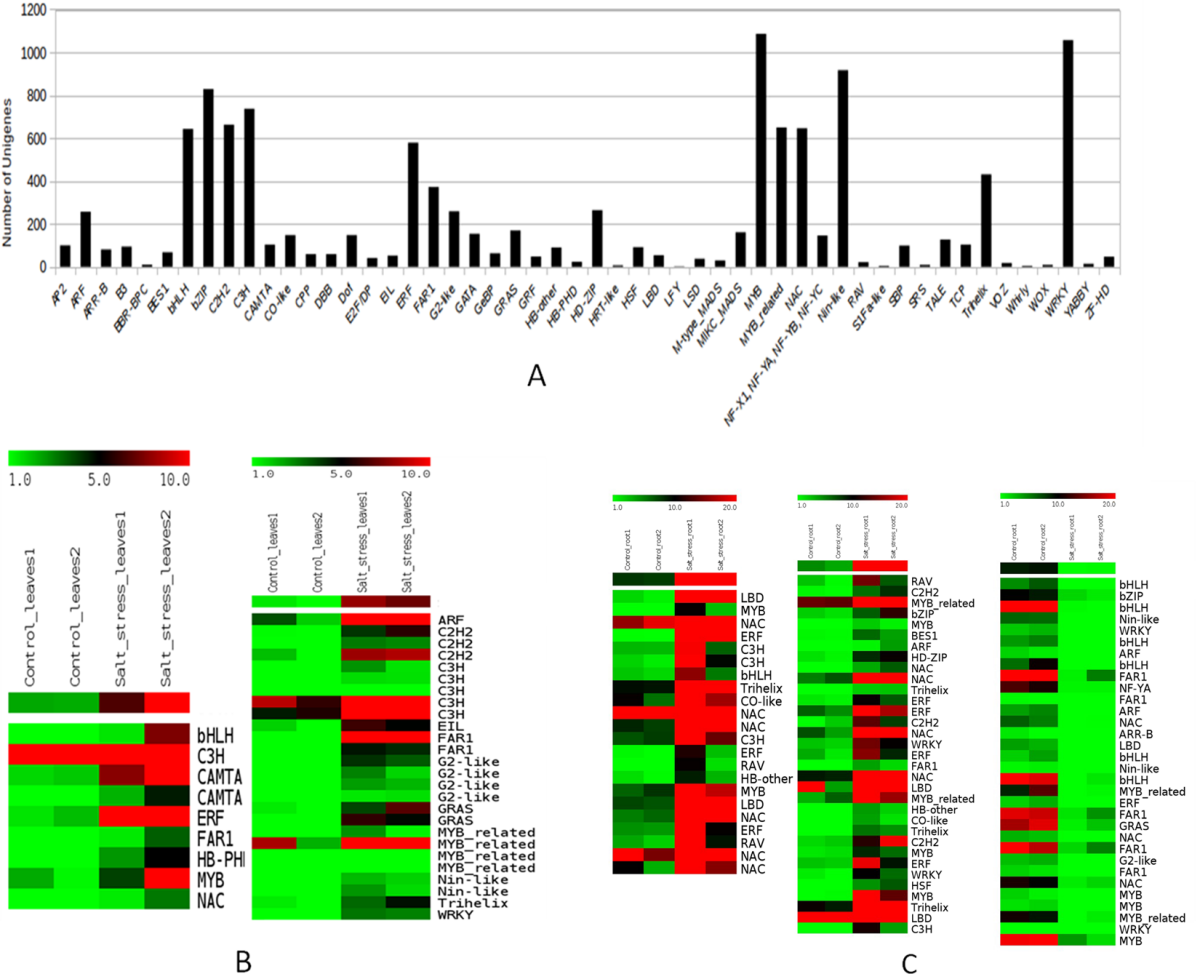
Transcription factors identified in *P. karka* transcriptome. (**A**) Frequency of distribution of TFs in *P. karka* transcriptome. Differential expression of Tfs under salinity stress in (**B**) Leaves and (**C**) Roots. Heat maps were generated using the MeV v.4.8.1 (https://sourceforge.net/projects/mev-tm4/files/).

Transcription factors are crucial regulatory molecules that regulate gene expression in an organism. Numerous studies have reported the role of many transcription factors in response to salt stress^17,18^. Therefore, transcription factors (TFs) were identified in the transcriptome of *P. karka* and a total of 11,242 TFs were predicted. MYB (1086 unigenes) and WRKY (1057 unigenes) transcription factors accounted for the vast majority of predicted TFs followed by Nin-like (917), bZIP (829), C3H (737) and C2H2 (663). Apart from this, a number of NAC, bHLH, FAR1 and Trihelix TFs were also identified (Fig. 4A). *In silico* differential gene expression analysis showed that members of ARF, C3H, MYB, C2H2, and FAR1 were upregulated in leaves during salt stress (Fig. 4B). In the case of roots, members of NAC and Trihelix TF families were seen to be upregulated while a number of bHLH TFs were downregulated (Fig. 4C).

### Validation of differentially expressed genes

**Figure 5:**
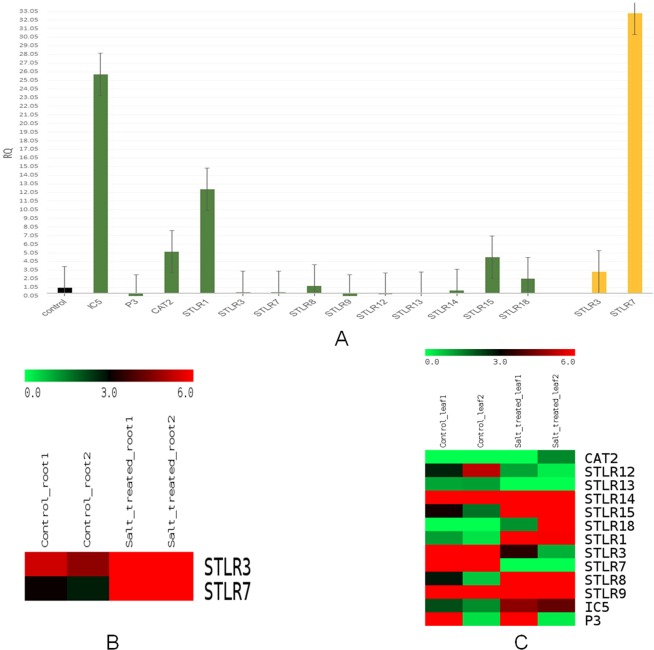
Validation of *in silico* gene expression analysis using qRT PCR (**A**) and Heatmap showing the *in silico* expression data for roots (**B**) and leaves (**C**) subjected to salinity stress (drawn using MeV 4.8.1; https://sourceforge.net/projects/mev-tm4/files/).

Thirteen genes (designated STLR1–18) were selected randomly based on their differential expression in response to salinity stress as observed *in silico*. Their expression was confirmed through qRT-PCR (primer sequences provided in Table S4). It was observed that the expression pattern of most of the candidate genes in the qRT-PCR analysis showed a similar trend to that of *in silico* analysis (Fig. 5), green for salt treated leaves and yellow for salt treated root. A gene encoding ion channel (IC5) was found to be up regulated in leaf tissue after exposure to salt stress while an un-annotated gene, (depicted as P3) was found to be downregulated (Fig. 5). The results are in agreement with the pattern of expression in the matrix generated by edgeR (Fig. 5, Table S5).

### SSR identification

**Figure 6:**
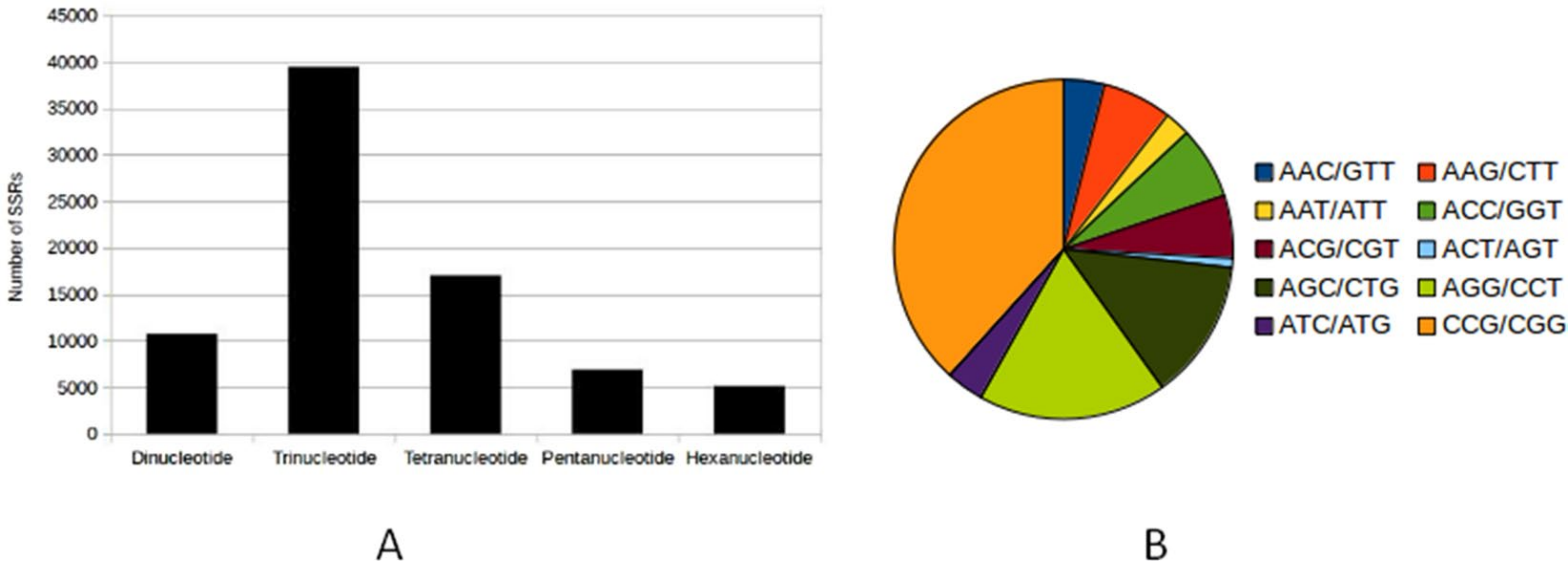
Identification of genic SSRs in *P. karka* transcriptome. (**A**) Distribution of SSRs according to their unit size (**B**) Distribution of trinucleotide repeats containing SSRs into sub categories based on nucleotide composition. Figures were made using the data generated by misa.pl (in Methods, SSR identification) on MS Excel.

**Figure 7:**
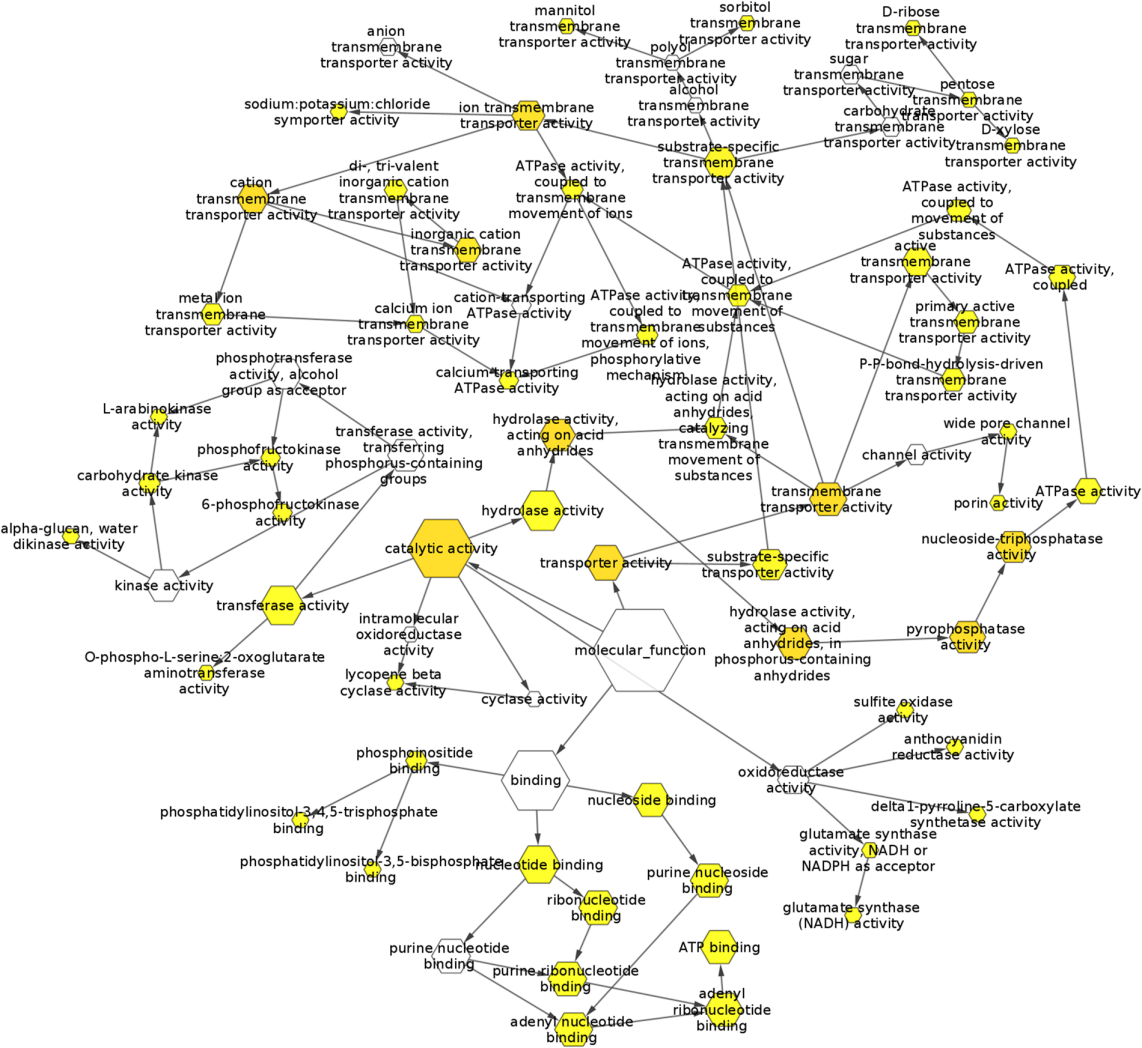
GO term enrichment for SSR containing DEGs of *P. karka* in the Molecular functions category. BiNGO app of Cytoscape v 3.7.2 was used to generate the figure (https://cytoscape.org/).

Simple sequence repeats (SSRs) are important molecular markers and a valuable source of variation in different plants. The *P. karka* transcriptome was mined for genic SSRs using MISA perl script^19^. A total of 79,300 SSRs were identified in 50,456 unigene sequences (Table 2). It was seen that majority of the repeats were trinucleotides (49.74%), followed by tetranucleotide repeats (21.46%) and dinucleotide repeats (13.58%). Amongst the trinucleotide repeats, CCG/CGG type repeats were most abundant (38.29%) followed by AGG/CCT (17.99%) and AGC/CTG (13.37%) (Fig. 6). The sequences containing SSRs were retrieved from the *P. karka* transcriptome and analysed for differential expression during salinity stress. Of the 50,456 unigenes containing SSRs, 442 were found to be differentially expressed in leaf and root tissue (IDs listed in Table S6). The DEGs containing SSRs were enriched for GO terms based on their homology with the peptides reported in *Arabidospsis* and it was observed that GO terms related to ion and sugar transporters, aquaporins, oxidoreductases and ABC transporters were over-represented (Fig. 7).

**Table 2:** SSRs identified in *P. karka* transcriptome.

Results of Microsatellite search	
Total number of sequences examined	161,403
Total size of examined sequences (bp)	218566080
Total number of identified SSRs	79300
Number of SSR containing sequences	50456
Number of sequences containing more than 1 SSR	17933
Number of SSRs present in compound formation	2936
**Distribution to different repeat type classes**	
Unit size	Number of SSRs
Dinucleotide	10771
Trinucleotide	39449
Tetranucleotide	17019
Pentanucleotide	6906
Hexanucleotide	5155

## Discussion

Increased salinity of arable land has a number of detrimental effects on crop yield since it leads to delayed seed germination and slower plant growth, resulting in considerable reduction in grain yield, quality and quantity^20^. Plants employ a number of complex molecular mechanisms to alleviate the effects of such stress conditions. They respond through well defined pathways that involve stress sensing, signal transduction, and the activation of a number of stress-responsive genes and metabolites. However, the ability to tolerate such stresses often comes at the cost of reduced yield and biomass. Therefore, plants that can naturally tolerate higher levels of salinity may serve as a source of genes and metabolites that can improve salinity stress tolerance in susceptible plants.

In this study, we report the transcriptome of *Phragmites karka* in response to salinity stress. A comprehensive transcriptomic analysis was performed in both salt treated and untreated plants through Illumina based paired-end read sequencing. *De novo* assembly of the reads using BinPacker^21^ and rnaSPAdes^22^ assemblers generated a total of 161,403 unigenes. Both software packages have been developed to be more flexible in their assembly parameters and thereby provide a more comprehensive transcriptome assembly compared to others^23^. Quality assessment showed that 84.6% complete BUSCOs were represented in the *P. karka* transcriptome, of which, 51.9% were single copy and 32.7% were duplicated. The percentage of duplicated BUSCOs may be due to the level of polyploidy in the genome of *P. karka* which transcribes into the transcriptome. Also, on average, 74% of the unigenes were found to code for long, complete ORFs, thus verifying the good quality of assembly. Functional annotation with GO terms revealed that categories like “metabolic processes”, “catalytic activity” and “binding” were found to account for the majority of unigenes. Enrichment of GO terms, such as “response to hormone stimulus” and “signaling”, has also been reported in a number of similar studies^24^ in response to early salt stress. The observations suggest that *P. karka* and many other plants share common pathways for regulating gene expression in response to salinity. KEGG enrichment analysis revealed that a number of genes were involved in the pathways related to MAPK signaling pathway, Glycolysis/Gluconeogenesis, plant hormone signal transduction, Ubiquitin-mediated proteolysis, Pyrimidine metabolism and Oxidative Phosphorylation. These pathways are principally involved in cell wall biosynthesis, cell proliferation, nutrient accumulation, primary metabolism and hormone signaling, all processes that regulate abiotic stress tolerance in plants.

Generally, the application of any stress elicits similar complex molecular responses in most plants. These include changes in gene expression, transcriptional regulation and signal transduction networks^18^. In this study, a total of 1342 unigenes were found to be differentially expressed in leaf tissue, 1016 unigenes were differentially expressed in root tissue during exposure to salinity stress and 74 DEGs were common to both leaf and root tissue. The leaf and root tissues of *P. karka* had similar patterns of expression in terms of TFs. Both tissues showed a higher expression of TFs like ERF, NAC, MYB and C2CH Zn finger genes. These TFs have been previously reported in many studies to have significant roles in salinity and drought stress tolerance^17^. Although the tissues had common differentially expressed TFs, they also showed distinct patterns of gene expression. For example, genes encoding heat shock proteins, chaperones, glutathione S transferase, and components of the 26 S proteasome pathway were over-represented in leaf tissue subjected to salinity stress while the root tissue showed a number of various transporters like polyol transporters, ion transporters and antioxidants to be expressed differentially. The data suggest that ion exchange and active transporters are more operational during salinity stress in roots whereas in leaves, salinity stress affects protein turnover and folding. In addition, there was an abundance of F-box proteins in leaf tissue subjected to salinity stress. These have been known to be involved in many plant reproduction, hormone signaling and developmental processes including response to abiotic stress^25^. For example, it was reported that over-expression of the gene *AtPP2-B11*, which encodes an F-box protein in *Arabidopsis*, improves salinity tolerance by expression of annexin1 (*AnnAt1*), a key responsive gene to oxidative stress^26^. A number of low molecular weight organic compounds such as proline, glycine-betaine and sugars are known to act as osmoprotectants in halophytes^27^. These compounds were also detected in the transcriptome of *P. karka*, thereby validating the assembly as well as suggesting that the role of these osmolytes in salinity and drought tolerance is universal. Studies have also reported that in addition to these small molecules, plant secondary metabolites also have an osmoregulatory role and are involved in plant defense and stress acclimation^28^. These compounds are also involved in reactive oxygen species (ROS) scavenging, enzyme activation, photoprotection and signal regulation^29^. The presence of such gene families in *P. karka* points to the role of osmoprotectants in salinity response.

A total of 74 DEGs were found to be common to both leaf and root tissue. Genes encoding 40 S ribosomal protein, MYB -related TFs, LYR motif-containing protein, NADH-ubiquinone oxidoreductase, Ethylene responsive factor, Cysteine rich repeat containing proteins were found to be downregulated in leaf tissue while being upregulated in root tissue during salt stress. Ribosomal proteins are known to regulate crucial processes such as protein synthesis, cell growth, development and apoptosis^30^. The ribosomal proteins identified in this study will make viable candidates to study their involvement in abiotic stress tolerance. The role of MYB and MYB-related TFs in salinity stress tolerance has been studied in numerous plants including grasses like alfalfa^31^ and food crops like maize^32^. LYR motif containing proteins along with NADH-ubiquinone oxidoreductase are components of the mitochondrial complex I, the first complex in the respiratory chain. Increased expression of genes encoding these proteins in root tissue in response to salt stress implies an increase in ATP production, which could, in turn, drive the active transporters to remove excess solutes from the roots. Similar reports have been found in case of *Arabidopsis*^33^. On the other hand, gene encoding cellulose synthase was observed to be up regulated in leaves while being down regulated in roots. Various studies have reported the effects of salinity stress on cellulose synthesis, a key component of cell walls^34,35^. Thus, up regulation of genes encoding cellulose synthase in leaf tissue of *P. karka* in response to salt stress indicates fortification of the cellular structure to alleviate the effects of osmotic stress and to prevent cell damage.

*Phragmites karka* is an invasive species. Over the past decades, studies have contributed to an understanding of the biology and ecology of plant invasion^36^. With the onset of the genomics era, many studies have undertaken to analyse the genomic basis of plant invasion^37^. These studies provide important insights into the genomics of plant invasiveness and support the overall idea that genome plasticity allows an invasive plant species to adapt to its surroundings more efficiently. Genome plasticity is the ability of the genome to rearrange to suit the needs of the plant and is affected by a number of factors. Polyploidy is key factor determining plant genome plasticity^2,38^. The genus *Phragmites* is known to comprise polyploid species^2^ and *P. karka* is tetraploid in nature (2n = 4×=36)^39^. Most polyploids have been reported to contain novel variations, and are superior to their corresponding diploids in terms of tolerance to environmental stresses^40^. This phenomenon could be associated with additive changes due to the level of heterozygosity and the gene dosage^41^.

In an earlier study of transcriptome analysis of the invasive species, *Mikania micrantha*^37^, a number of DEGs involved in processes like photosynthesis, energy metabolism, wound healing, protein modification, asexual reproduction, and biological regulation were identified. Amongst the DEGs found in this study, there were a number of genes regulating protein turnover (26 S proteasome, F-box proteins etc.) and secondary metabolite production (Caffeoylshikimate esterase). Plant hormones, especially abscisic acid (ABA), are known to mediate signaling pathways in plants in response to various abiotic stresses^42^. Transcription factors such as NAC, AP2/ERF, MYB, bHLH, CCCH are associated with the regulation of ABA-mediated response of the plants to salinity stress^43^. In addition, the abundance of WRKY transcription factors reiterates studies that associate these TFs with abiotic stress tolerance in plants^44^. Therefore, the findings in our study are in agreement with previous reports. In addition, SSR markers are a valuable source of genomic variation and many transcriptomes have been screened to identify them in important plant species, including chinese hawthorn^45^ *Glycyrrhiza*^46^, coriander^47^ and *Curcuma alismatifolia*^48^ In this study, about 31% of the *P. karka* unigenes contained SSR loci. Trinucleotide repeats were found to be most abundant, which is in keeping with a number of previous reports in monocots^49^. The most abundant trinucleotide repeat was CCG/CGG followed by AGG/CCT. The results reiterate the observation that these motifs are common in monocots as reported in many previous studies^48^.

In conclusion, the root and leaf transcriptomes of *P. karka* revealed a large number of differentially expressed genes which could contribute to the discovery of potential stress-responsive candidates for functional study and further application in crop improvement. We have also identified a number of genic SSR markers from the transcriptomic dataset. These candidate SSR markers provide valuable resources for future ecological and evolutionary studies in *P. karka*.

## Methods

### Plant Material, growth conditions and salt stress treatment

Young plants of *Phragmites karka* were collected from Chilika Lake, Odisha and transferred to pots. For salinity stress treatment, the plants were removed from soil, washed carefully to remove soil from the leaves and roots and kept overnight in beakers containing distilled water to acclimatize them. The plants were then treated with 150 mM NaCl in distilled water for duration of 48 hrs and 72 hrs and plants treated with only distilled water were taken as control. Two biological replicates were collected for each tissue sample, washed thoroughly with 0.1% DEPC water, frozen in liquid nitrogen and stored at −80 °C until RNA extraction.

### RNA extraction, Illumina sequencing and data quality control

Total RNA was extracted from leaf and root samples of *P. karka* in duplicate (for control, 48hr and 72 hr of treatment) using TRIzol Reagent (Invitrogen)/RNeasy Mini Kit (Qiagen). RNA quantity and quality was determined using a Nanodrop (Thermo Fisher Scientific Inc.) and Agilent 2100 Bioanalyzer (Agilent Technologies, Palo Alto, CA, USA). 1 μg total RNA with RIN value above 7 was used for library preparation. Next generation sequencing library preparations were constructed using NEBNext Ultra RNA Library Prep Kit for Illumina according to the manufacturer’s protocol. The quality of library was checked on Qubit 2.0, Agilent 2100 and Q-PCR. After passing the quality filters the libraries were fed into HiSeq. 2500 sequencer after pooling according to its effective concentration and expected data volume.

### Read processing, assembly and annotation

The high quality (>70% sequences with phred score of Q30), adapter free reads were assembled using BinPacker (http://sourceforge.net/projects/transcriptomeassembly/files/BinPacker_1.0.tar.gz/download) and rnaSPAdes (cab.spbu.ru/software/rnaspades/). For BinPacker the assembly was at kmer k = 25. For rnaSPAdes the assembly was computed at k = 69. In all the cases, the assembly’s minimum lengths for transcript reporting were taken as 200 bp. The individual assemblies were merged and redundant transcripts were removed using CD-HIT-EST^50^ and CAP3^51^ softwares. Quality of the final assembled transcriptome was assessed using these parameters: (i) identifying long ORFs within the transcript sequences using Perl script ORF Predictor^52^ and Transdecoder (https://github.com/TransDecoder/TransDecoder/wiki) (ii) comparing to Benchmarking Universal Single-Copy Orthologs (BUSCO) database. In addition to these, indicators like N50 and contig length distribution were also used to determine assembly quality.

Functional annotation of the transcripts was carried out by assigning GO terms after BLASTx search against the Uniprot-Swissprot database. The standalone version of BLAST was downloaded from the ftp site at NCBI (ftp://ftp.ncbi.nlm.nih.gov/blast/executables/blast+/2.9.0/) and the BLASTx program was used to perform the search against Uniprot-Swissprot database (https://www.uniprot.org/uniprot/?query=reviewed:yes) using a e-value cut off of 10^–5^. GO annotations of these proteins were also downloaded from the FTP site of GO database under Uniprot (ftp://ftp.ebi.ac.uk/pub/databases/GO/goa/UNIPROT/gene_association.goa_uniprot.gz). GO terms and their corresponding GO Slim terms were downloaded from Uniprot GOA database (ftp://ftp.ebi.ac.uk/pub/databases/GO/goa/goslim/goaslim.map). All the plant GOSlim terms were searched and saved from EBI’s QuickGO-Beta server (http://www.ebi.ac.uk/QuickGO-Beta/) which is provided by UniProt-GOA project and incorporates annotations from the GO consortium and other specialist groups. These plant GOSlim terms were assigned to our corresponding transcripts using linux shell commands. The KAAS (KEGG Automatic Annotation Server; https://www.genome.jp/kegg/kaas/) web-server was used to assign biological pathways to the transcripts^53^. Protein function annotation by comparison against COG database was done using the web-server on WebMGA^54^ http://weizhong-lab.ucsd.edu/webMGA/server/).

### Differential gene expression analysis

Bowtie2 (https://sourceforge.net/projects/bowtie-bio/) was used to determine the abundance of each transcript by mapping the raw reads onto the assembled transcriptome. Abundance was calculated by RSEM (RNA-Seq by Expectation-Maximization-http://deweylab.github.io/RSEM/package) for each library^55^. Differentially expressed genes (DEGs) among the salinity stressed and control libraries were calculated by using the Empirical Analysis of Digital Gene Expression (edgeR) (http://biocon-ductor.org/packages/release/bioc/html/edgeR.html) statistical package^16^. The normalization factors were calculated using trimmed mean of M-values (TMM) method. The threshold FDR < 0.05 was adjusted to identify the differentially expressed genes by fold change (≥2).

### Identification of transcription factors

The peptide sequences for transcription factors of *Oryza sativa indica* were downloaded from Plant TFDB (http://planttfdb.cbi.pku.edu.cn/index.php?sp=Osi). BLASTX program of NCBI (ftp://ftp.ncbi.nlm.nih.gov/blast/executables/blast+/2.9.0/) was used to search the *P. karka* transcripts against the *O. sativa indica* transcription factors using an e-value cutoff of 10^–5^. All heat maps were generated after *in silico* gene expression analysis using MeV (v. 4.8.1) software (https://sourceforge.net/projects/mev-tm4/).

### Quantitative real-time PCR (qRT-PCR) analysis

For the validation of *in silico* data, qRT-PCR was done using ∆∆Ct method^56^. To validate the data generated through RNA Seq, 13 genes selected from the DEG’s analysis were subjected to quantitative real-time PCR (q RT PCR). The primer pairs were designed using Unigene sequences obtained in this study. The RNA samples used for sequencing from the biological replicates were used for the validation. The total RNA was reverse transcribed using First-Strand cDNA synthesis kit (Thermo Scientific, USA). After assessing quality control Nanodrop Spectrophotometer, the cDNA samples were diluted. The housekeeping gene elongation factor1α was used as endogenous control. The qRT PCR analysis was performed using Applied Biosystems QuantStudio 3 Real-Time PCR system with SYBR green chemistry (Applied Biosystems, USA) in three technical replicates. The primer sequences for the unigenes are provided in Table S4.

### SSR identification

SSRs were identified in the assembled unigenes of *P. karka* using MISA software (https://github.com/cfljam/SSR_marker_design/blob/master/misa.pl) with the following parameters in the misa.ini file: a minimum of 6 repeats for dinucleotide, 4 repeats for trinucleotide and 3 repeats for tetra, penta and hexanucleotide with a maximum interruption of 10 bases between two SSRs. GO enrichment was carried out using BiNGO app of Cytoscape v 3.7.2^57^ after conducting a homology search against the peptide database of Arabidopsis (https://www.arabidopsis.org/download/index-auto.jsp?dir=/download_files/Proteins).

## Supporting Information

Supporting Information.
Supporting Information2.
Supporting Information3.
Supporting Information4.
Supporting Information5.
Supporting Information6.
Supporting Information7.
