# Supplementary figures and images for: De novo transcriptome assembly and analysis of Phragmites karka, an invasive halophyte, to study the mechanism of salinity stress tolerance
Source: Sci Rep. 2020 Mar 23;10:5192. doi: 10.1038/s41598-020-61857-8 (PMC7089983; doi:10.1038/s41598-020-61857-8)

# BUSCO Assessment Results

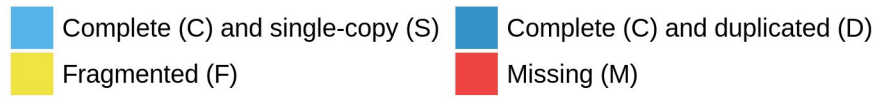

Phragmites\_busco

C:2772 [S:1701, D:1071], F:319, M:187, n:3278

0

20

40

60

80

100

%BUSCOs

Supplement: Supplementary file 1 — Supporting Information. [file 41598_2020_61857_MOESM1_ESM.pdf]
