# Supplementary material for: De novo transcriptome assembly and analysis of Phragmites karka, an invasive halophyte, to study the mechanism of salinity stress tolerance
Source: Sci Rep. 2020 Mar 23;10:5192. doi: 10.1038/s41598-020-61857-8 (PMC7089983; doi:10.1038/s41598-020-61857-8)
Supplement: Supplementary file 2 — Supporting Information2. [file 41598_2020_61857_MOESM2_ESM.pdf]

TableS1a. In silico expression data for DEGs in leaf tissue of *P. karka* during exposure to salinity stress

DEGs in leaf tissue under salinity stress: The data below corresponds to the differential expression of unigenes in P. karka leaves subjected to salinity stress. LogFC denotes the level of expression in sample A as compared to sample B

Unigene ID	sample A	sample B	logFC	logCPM	pvalue	FDR
NODE_875_length_7193_cov_31.008567_g0_i26	Control	Salt_treated	15.6025471	9.49782912	7.351E-42	1.801E-37
BINPACKER_627_5	Control	Salt_treated	10.26847261	4.149767565	1.007E-23	3.525E-20
NODE_235_length_10039_cov_29.675597_g32_i4	Control	Salt_treated	10.02659071	3.893879693	1.327E-25	6.5E-22
NODE_21020_length_2688_cov_33.556405_g6301_i81	Control	Salt_treated	9.861234974	3.646783464	6.181E-06	0.00049
BINPACKER_5433_10	Control	Salt_treated	9.713884764	3.587629874	8.034E-22	2.187E-18
NODE_47868_length_1727_cov_28.326481_g23850_i1	Control	Salt_treated	9.705673132	3.59554103	5.685E-17	7.329E-14
BINPACKER_2578_4	Control	Salt_treated	9.684074528	3.561234534	1.345E-20	2.995E-17
BINPACKER_28145_5	Control	Salt_treated	9.493829216	3.373259955	1.065E-18	1.739E-15
NODE_65062_length_1358_cov_111.919066_g27862_i51	Control	Salt_treated	9.474336316	3.348588375	9.821E-20	1.85E-16
NODE_32616_length_2174_cov_24.599238_g2618_i2	Control	Salt_treated	9.361735525	3.196854845	1.275E-13	9.465E-11
BINPACKER_4117_8	Control	Salt_treated	9.276804297	3.143471014	1.808E-18	2.768E-15
BINPACKER_1736_15	Control	Salt_treated	8.692589529	2.582105568	2.719E-13	1.8E-10
BINPACKER_11298_1	Control	Salt_treated	8.486992648	2.405534008	2.087E-10	7.2E-08
BINPACKER_3224_11	Control	Salt_treated	8.443338161	2.263701749	1.048E-07	1.689E-05
NODE_39365_length_1950_cov_26.508791_g6383_i2	Control	Salt_treated	8.370274341	2.315503382	1.985E-08	4.086E-06
Contig10910	Control	Salt_treated	8.246241911	2.076480463	2.816E-07	3.987E-05
BINPACKER_2161_4	Control	Salt_treated	8.233149271	2.140443545	9.973E-11	3.94E-08
BINPACKER_14225_7	Control	Salt_treated	8.156478549	2.060698397	1.919E-10	6.812E-08
BINPACKER_3574_3	Control	Salt_treated	8.068357244	2.007696663	5.557E-09	1.375E-06
BINPACKER_12014_1	Control	Salt_treated	8.011083262	1.893414893	2.527E-08	4.952E-06
BINPACKER_15274_4	Control	Salt_treated	7.987827199	1.897252782	1.762E-09	4.796E-07
NODE_11190_length_3455_cov_31.498817_g5600_i0	Control	Salt_treated	7.986701568	1.909070093	1.182E-09	3.574E-07
BINPACKER_3980_3	Control	Salt_treated	7.979169686	1.893186727	1.354E-09	3.902E-07
BINPACKER_11299_1	Control	Salt_treated	7.870124266	1.832150257	5.936E-08	1.061E-05
BINPACKER_11333_7	Control	Salt_treated	7.708612998	1.659719606	2.3E-08	4.623E-06
BINPACKER_3622_5	Control	Salt_treated	7.539808457	1.524963238	2.664E-07	3.828E-05
NODE_12690_length_3303_cov_21.344582_g6352_i1	Control	Salt_treated	7.460943826	1.438635196	1.575E-07	2.411E-05
NODE_53485_length_1594_cov_23.708087_g6470_i1	Control	Salt_treated	7.425075011	1.405506283	2.542E-07	3.685E-05
BINPACKER_2511_14	Control	Salt_treated	7.351475935	1.358146005	6.865E-07	8.046E-05
BINPACKER_8747_7	Control	Salt_treated	7.232057606	1.172738368	4.837E-05	0.0022574
BINPACKER_2461_5	Control	Salt_treated	7.177336024	1.208878864	2.188E-06	0.0002144
NODE_61217_length_1432_cov_30.080942_g30970_i0	Control	Salt_treated	7.154197958	1.122765758	3.044E-05	0.0015925
BINPACKER_7001_3	Control	Salt_treated	7.116165727	1.139926943	1.979E-06	0.0001986
BINPACKER_27799_2	Control	Salt_treated	7.045950261	0.998526496	0.0003505	0.0097148
BINPACKER_4195_9	Control	Salt_treated	6.880735313	1.019780514	0.0002499	0.0078182
NODE_48062_length_1722_cov_49.464524_g24101_i0	Control	Salt_treated	6.597436748	0.713931033	0.0001051	0.0041465
NODE_68388_length_1298_cov_30.328980_g10566_i4	Control	Salt_treated	6.557360392	0.66421458	0.0003472	0.0096424
NODE_43661_length_1832_cov_24.445708_g21450_i1	Control	Salt_treated	6.538739308	0.737396272	0.0004121	0.0108431
NODE_68676_length_1293_cov_22.790164_g34668_i1	Control	Salt_treated	6.498242306	0.689377882	0.0002392	0.0075686
NODE_80838_length_1096_cov_19.468231_g29241_i5	Control	Salt_treated	6.498242306	0.689377882	0.0002392	0.0075686
BINPACKER_56091_3	Control	Salt_treated	6.491878458	0.607406002	0.0006546	0.0149984
BINPACKER_598_3	Control	Salt_treated	6.39610168	0.587425862	0.000145	0.0053237
BINPACKER_18216_1	Control	Salt_treated	6.339848102	0.533165317	0.0003052	0.0088584
NODE_37598_length_2003_cov_29.933161_g18863_i0	Control	Salt_treated	6.276063873	0.518162632	0.0004137	0.0108625
BINPACKER_10852_2	Control	Salt_treated	6.250186899	0.513184864	0.0006927	0.0155115
NODE_141871_length_521_cov_4.705357_g89276_i0	Control	Salt_treated	6.192295786	0.456297454	0.0005298	0.0128624
NODE_56160_length_1537_cov_35.086066_g28317_i0	Control	Salt_treated	6.176022439	0.406893638	0.0009804	0.0194286
NODE_45113_length_1793_cov_32.323256_g22541_i1	Control	Salt_treated	6.029305999	0.32915957	0.0010908	0.0208015
BINPACKER_14629_10	Control	Salt_treated	5.981321588	0.318987891	0.0012635	0.0230102
NODE_143050_length_514_cov_6.011338_g90328_i0	Control	Salt_treated	5.927475007	0.258282711	0.0018342	0.0292981
BINPACKER_12041_2	Control	Salt_treated	5.927475007	0.258282711	0.0018342	0.0292981
BINPACKER_12041_4	Control	Salt_treated	5.927475007	0.258282711	0.0018342	0.0292981
NODE_47419_length_1737_cov_14.825120_g23774_i0	Control	Salt_treated	5.87703848	0.248041486	0.0016863	0.027586
NODE_33775_length_2132_cov_16.494900_g16909_i0	Control	Salt_treated	5.87703848	0.248041486	0.0016863	0.027586
NODE_58754_length_1483_cov_24.571631_g26965_i2	Control	Salt_treated	5.836726901	4.514710228	1.233E-15	1.208E-12
BINPACKER_22260_4	Control	Salt_treated	5.818596005	0.183773977	0.0032042	0.0426555
Contig10757	Control	Salt_treated	5.789159487	0.178611507	0.002426	0.0356072
BINPACKER_225_4	Control	Salt_treated	5.763364469	0.17346254	0.0035345	0.0454958
BINPACKER_669_8	Control	Salt_treated	5.667099397	0.100043664	0.0040635	0.0494453
NODE_47644_length_1732_cov_29.517782_g22843_i1	Control	Salt_treated	5.667099397	0.100043664	0.0040635	0.0494453
NODE_62246_length_1411_cov_28.113602_g23187_i1	Control	Salt_treated	5.667099397	0.100043664	0.0040635	0.0494453
BINPACKER_104006_1	Control	Salt_treated	5.667099397	0.100043664	0.0040635	0.0494453
BINPACKER_7553_7	Control	Salt_treated	5.268204538	2.177694561	1.086E-09	3.326E-07
BINPACKER_25794_2	Control	Salt_treated	5.168649811	1.396213684	0.0003254	0.0092392
BINPACKER_3064_3	Control	Salt_treated	5.013360796	1.263264142	0.0001957	0.0065937
Contig4177	Control	Salt_treated	4.751429538	0.972469616	0.0002769	0.0083434
NODE_35893_length_2060_cov_23.523905_g18003_i0	Control	Salt_treated	4.657362992	0.877919731	0.0008171	0.0172635
BINPACKER_1356_3	Control	Salt_treated	4.612004421	0.868219549	5.334E-05	0.0024241
BINPACKER_6475_20	Control	Salt_treated	4.569567667	2.000303644	1.858E-07	2.771E-05
BINPACKER_376_1	Control	Salt_treated	4.417764976	7.618288756	1.064E-08	2.349E-06
NODE_192582_length_333_cov_49.269231_g136943_i0	Control	Salt_treated	4.247693858	0.636112084	0.0014003	0.0244475
BINPACKER_62678_1	Control	Salt_treated	4.127029468	1.08435081	0.0025528	0.0368134
BINPACKER_46_3	Control	Salt_treated	4.092349542	7.472902279	1.093E-25	6.5E-22
NODE_80068_length_1107_cov_34.284333_g41882_i0	Control	Salt_treated	4.089181566	4.296972289	3.608E-06	0.0003145
NODE_46821_length_1751_cov_11.604291_g23489_i0	Control	Salt_treated	4.075609798	1.850282961	0.0005079	0.0124298
BINPACKER_3720_7	Control	Salt_treated	4.026713502	1.126876949	4.896E-05	0.0022757
NODE_34133_length_2120_cov_17.817294_g17104_i0	Control	Salt_treated	4.019667442	0.462908446	0.003461	0.0448212
BINPACKER_6036_8	Control	Salt_treated	4.002556033	2.697449141	0.0001454	0.0053299
BINPACKER_24495_3	Control	Salt_treated	4.002404243	1.529857701	1.399E-05	0.0009186
Contig741	Control	Salt_treated	3.903236897	1.010265414	0.000204	0.0067702
BINPACKER_6893_1	Control	Salt_treated	3.880297622	1.06280897	0.0008525	0.0177723
BINPACKER_13594_2	Control	Salt_treated	3.8534932	0.988990578	0.000184	0.0062762
BINPACKER_2095_11	Control	Salt_treated	3.848633435	1.376533352	0.000146	0.0053461
BINPACKER_996_1	Control	Salt_treated	3.814056966	0.924585557	0.0009862	0.0195029
BINPACKER_61704_1	Control	Salt_treated	3.793953855	1.391324556	5.56E-05	0.0025174
BINPACKER_11582_4	Control	Salt_treated	3.784104048	0.874700817	0.0016312	0.0271303
NODE_56047_length_1540_cov_20.166326_g16600_i3	Control	Salt_treated	3.751889242	1.274287719	0.0009376	0.0188874
BINPACKER_59_5	Control	Salt_treated	3.714503844	4.16114266	1.552E-07	2.391E-05
BINPACKER_4554_4	Control	Salt_treated	3.709936506	0.826851933	0.0019152	0.0300923
NODE_82843_length_1068_cov_32.400000_g43608_i0	Control	Salt_treated	3.692774952	1.244854358	0.0021694	0.032742
NODE_45859_length_1774_cov_21.871252_g22988_i0	Control	Salt_treated	3.657007901	0.85297379	0.000413	0.0108538
NODE_41780_length_1882_cov_32.033167_g20888_i0	Control	Salt_treated	3.64014899	0.769882643	0.0028795	0.0399531
NODE_19277_length_2795_cov_17.316312_g9608_i0	Control	Salt_treated	3.616715931	0.807370069	0.0004639	0.0116583
NODE_21357_length_2669_cov_28.042758_g10636_i0	Control	Salt_treated	3.611643944	2.27231518	8.917E-05	0.0036571
BINPACKER_1516_4	Control	Salt_treated	3.595504064	3.256493674	6.031E-05	0.0026763
BINPACKER_335_7	Control	Salt_treated	3.59310161	13.69787186	2.379E-18	3.428E-15
BINPACKER_592_5	Control	Salt_treated	3.568530218	2.384571344	0.0007777	0.0166806
Contig9190	Control	Salt_treated	3.547024835	1.22069002	0.0006553	0.0150011
BINPACKER_19457_1	Control	Salt_treated	3.544504726	1.140192847	0.0007497	0.0163379
NODE_115423_length_705_cov_25.376582_g66593_i0	Control	Salt_treated	3.530216523	1.141333512	0.0004454	0.0113186
BINPACKER_24996_2	Control	Salt_treated	3.502363319	0.708567398	0.0015389	0.0261223
BINPACKER_6782_10	Control	Salt_treated	3.445586808	0.692577675	0.0010894	0.0208015
NODE_78906_length_1125_cov_27.942015_g41204_i0	Control	Salt_treated	3.42977895	4.675632004	0.0005265	0.012798
NODE_62936_length_1398_cov_30.912453_g29311_i1	Control	Salt_treated	3.426177089	4.107576858	0.0035808	0.0457091

BINPACKER_15897_5	Control	Salt_treated	3.415376113	1.641440614	0.0001485	0.0054061
BINPACKER_14118_2	Control	Salt_treated	3.409212493	2.163149661	0.000855	0.0177929
NODE_6962_length_4036_cov_27.564471_g2862_i2	Control	Salt_treated	3.407154576	1.103130766	0.0016887	0.027586
BINPACKER_55507_2	Control	Salt_treated	3.395989341	1.351243698	0.0012581	0.0229292
NODE_41403_length_1893_cov_23.392857_g20700_i0	Control	Salt_treated	3.395899715	0.650133733	0.0031393	0.0419972
NODE_21655_length_2653_cov_27.673643_g10798_i0	Control	Salt_treated	3.379347699	0.643913787	0.0014566	0.0251618
BINPACKER_2918_5	Control	Salt_treated	3.355795958	1.42270232	0.0020196	0.0311525
NODE_61163_length_1434_cov_5.903012_g30933_i0	Control	Salt_treated	3.311110223	9.64262499	4.237E-14	3.46E-11
NODE_100904_length_846_cov_19.406210_g55631_i0	Control	Salt_treated	3.309821122	1.569830792	0.0001257	0.0047827
BINPACKER_17230_1	Control	Salt_treated	3.30670406	0.630409649	0.002796	0.0391267
BINPACKER_47395_1	Control	Salt_treated	3.298741998	4.887981996	8.229E-15	7.753E-12
Contig7118	Control	Salt_treated	3.258173276	11.50506453	1.103E-12	6.754E-10
BINPACKER_12202_7	Control	Salt_treated	3.245534993	2.039376931	0.0030222	0.0411809
Contig7614	Control	Salt_treated	3.245343694	13.78570494	3.169E-09	8.257E-07
Contig8714	Control	Salt_treated	3.237883182	3.045767436	6E-09	1.465E-06
Contig7314	Control	Salt_treated	3.236316344	1.225757589	0.0017061	0.0277293
NODE_63989_length_1378_cov_23.648276_g32482_i0	Control	Salt_treated	3.235931477	0.945915437	0.0012343	0.0226478
BINPACKER_8292_1	Control	Salt_treated	3.19029647	1.736370911	0.0006338	0.014632
BINPACKER_95431_1	Control	Salt_treated	3.176801827	7.056650281	6.66E-09	1.599E-06
NODE_124150_length_634_cov_21.905526_g73870_i0	Control	Salt_treated	3.156941635	0.518950328	0.0045142	0.0536508
Contig11817	Control	Salt_treated	3.152191577	0.511375749	0.0045494	0.0539384
Contig9449	Control	Salt_treated	3.149743815	1.227923065	0.0006945	0.0155214
BINPACKER_72698_1	Control	Salt_treated	3.149310753	6.110128515	8.928E-09	2.025E-06
NODE_101460_length_840_cov_279.384615_g56022_i0	Control	Salt_treated	3.143543038	10.59075061	2.21E-12	1.23E-09
NODE_38671_length_1969_cov_28.831224_g19395_i0	Control	Salt_treated	3.142951853	8.056048964	2.262E-20	4.617E-17
BINPACKER_16853_1	Control	Salt_treated	3.13084667	2.645648243	0.000138	0.0051152
BINPACKER_14065_3	Control	Salt_treated	3.090283062	0.881947276	0.0032742	0.0433153
NODE_67921_length_1307_cov_35.444084_g30860_i1	Control	Salt_treated	3.085394327	4.342592172	2.156E-10	7.334E-08
NODE_85841_length_1028_cov_6.057592_g45574_i0	Control	Salt_treated	3.084512038	1.133655125	0.0014903	0.0255079
Contig7891	Control	Salt_treated	3.074546888	10.10365151	5.009E-11	2.152E-08
BINPACKER_430_5	Control	Salt_treated	3.053084017	2.213606651	0.0002102	0.0069283
NODE_71784_length_1239_cov_29.917667_g36906_i0	Control	Salt_treated	3.038264296	7.173275141	1.075E-14	9.406E-12
NODE_9780_length_3603_cov_115.428329_g4942_i0	Control	Salt_treated	3.024128971	12.98795178	1.316E-09	3.839E-07
BINPACKER_12549_1	Control	Salt_treated	3.012033618	2.025279163	0.0009761	0.0194068
Contig7048	Control	Salt_treated	3.010081443	3.526621351	8.27E-07	9.378E-05
BINPACKER_577_1	Control	Salt_treated	3.007394969	3.823673221	1.71E-05	0.0010656
NODE_64957_length_1360_cov_27.984460_g32998_i0	Control	Salt_treated	2.994238948	4.858883081	1.362E-13	9.81E-11
BINPACKER_9_33	Control	Salt_treated	2.993940851	7.280006533	7.103E-11	2.9E-08
NODE_80759_length_1097_cov_28.265625_g42299_i0	Control	Salt_treated	2.989270743	4.822343174	1.992E-10	6.971E-08
BINPACKER_75_1	Control	Salt_treated	2.987194072	3.865462645	9.025E-10	2.798E-07
NODE_49520_length_1687_cov_29.903346_g24837_i0	Control	Salt_treated	2.976098101	4.769345914	4.841E-13	3.04E-10
NODE_81577_length_1086_cov_4.307009_g42811_i0	Control	Salt_treated	2.975192806	1.325728595	0.0008105	0.0171586
NODE_134558_length_563_cov_32.051020_g82779_i0	Control	Salt_treated	2.96637523	1.081130445	0.0012865	0.0231886
NODE_37895_length_1994_cov_27.515357_g19008_i0	Control	Salt_treated	2.946279401	1.323379459	0.0033662	0.0440464
NODE_31380_length_2218_cov_24.472727_g15715_i0	Control	Salt_treated	2.935823853	1.035590766	0.0040794	0.0494674
Contig6666	Control	Salt_treated	2.917709061	5.673819691	1.221E-15	1.208E-12
NODE_25650_length_2446_cov_27.918247_g6064_i2	Control	Salt_treated	2.905479704	1.002868346	0.0038827	0.0481566
BINPACKER_14_1	Control	Salt_treated	2.891946068	8.769050005	2.839E-11	1.371E-08
BINPACKER_53118_1	Control	Salt_treated	2.889903181	1.799309239	0.0025216	0.0365097
BINPACKER_9778_1	Control	Salt_treated	2.861460572	2.642273168	4.616E-06	0.0003912
NODE_8206_length_3835_cov_24.580276_g2607_i1	Control	Salt_treated	2.856724282	1.840106816	0.0036965	0.046764
NODE_90211_length_971_cov_3.518931_g48395_i0	Control	Salt_treated	2.839382622	1.603785951	0.0006893	0.0154912
NODE_66965_length_1324_cov_7.711431_g34116_i0	Control	Salt_treated	2.832675337	2.273092767	0.0006158	0.0143515
BINPACKER_11585_5	Control	Salt_treated	2.830568924	0.983979069	0.0026038	0.0372976
NODE_133011_length_573_cov_25.386000_g81401_i0	Control	Salt_treated	2.830052043	2.168282849	0.0019558	0.0304947
BINPACKER_117235_1	Control	Salt_treated	2.829965363	5.546573304	7.903E-09	1.826E-06
NODE_45189_length_1791_cov_24.700815_g22652_i0	Control	Salt_treated	2.82794241	7.663211096	1.525E-16	1.779E-13
NODE_2168_length_5733_cov_24.762191_g1112_i12	Control	Salt_treated	2.819497169	4.283660146	2.351E-05	0.0013266
BINPACKER_55238_2	Control	Salt_treated	2.81587354	1.252845143	0.0038237	0.0477132
NODE_12830_length_3290_cov_21.687908_g6427_i0	Control	Salt_treated	2.809656346	1.205518821	0.0013318	0.0237077
BINPACKER_31213_1	Control	Salt_treated	2.805064741	2.381605108	0.0004095	0.0108095
NODE_37058_length_2021_cov_11.436345_g5583_i2	Control	Salt_treated	2.760977217	0.940417757	0.0027209	0.0383695
NODE_198620_length_317_cov_1.045082_g142860_i0	Control	Salt_treated	2.758442939	4.590129351	1.731E-08	3.624E-06
NODE_248_length_9957_cov_29.699110_g132_i0	Control	Salt_treated	2.74810461	8.1391035	2.44E-12	1.328E-09
NODE_70031_length_1270_cov_28.499582_g35854_i0	Control	Salt_treated	2.744582929	0.9356849	0.0030389	0.0412168
NODE_62701_length_1403_cov_14.715789_g22281_i2	Control	Salt_treated	2.741373301	2.62522519	0.0002887	0.0085613
NODE_133946_length_567_cov_29.457490_g82237_i0	Control	Salt_treated	2.734645542	1.336407301	0.0045637	0.0540298
NODE_8622_length_3766_cov_36.254806_g2948_i1	Control	Salt_treated	2.731393723	11.78696587	4.103E-11	1.827E-08
NODE_74367_length_1196_cov_25.065004_g38413_i0	Control	Salt_treated	2.731265324	0.903818154	0.0045179	0.0536687
NODE_76566_length_1161_cov_16.766544_g39744_i0	Control	Salt_treated	2.711030841	1.509502931	0.0016875	0.027586
NODE_9027_length_3707_cov_32.456522_g456_i4	Control	Salt_treated	2.700710389	2.003919053	0.0002559	0.0079231
NODE_9026_length_3707_cov_32.655751_g456_i3	Control	Salt_treated	2.700710389	2.003919053	0.0002559	0.0079231
NODE_109685_length_758_cov_26.137226_g62011_i0	Control	Salt_treated	2.694063704	2.956998674	7.735E-06	0.0005866
BINPACKER_56085_1	Control	Salt_treated	2.678650899	7.174200388	1.154E-10	4.416E-08
BINPACKER_12853_2	Control	Salt_treated	2.677823671	4.340281706	1.146E-07	1.835E-05
NODE_44155_length_1818_cov_20.106590_g22104_i0	Control	Salt_treated	2.677750192	0.889498499	0.0038559	0.0479196
BINPACKER_9801_3	Control	Salt_treated	2.67320474	1.117859761	0.0022747	0.033996
Contig9811	Control	Salt_treated	2.672493139	5.555792148	0.0002342	0.00749
BINPACKER_2187_1	Control	Salt_treated	2.670007029	7.951301877	3.913E-11	1.775E-08
BINPACKER_30082_1	Control	Salt_treated	2.668167098	3.234421377	0.0003763	0.0101733
BINPACKER_1614_1	Control	Salt_treated	2.665837593	10.73864746	7.601E-09	1.773E-06
NODE_41775_length_1882_cov_33.639027_g20886_i0	Control	Salt_treated	2.656169819	1.284937255	0.0027256	0.0383912
BINPACKER_1935_8	Control	Salt_treated	2.646076868	3.717791948	1.591E-08	3.359E-06
NODE_49912_length_1678_cov_19.519003_g25040_i0	Control	Salt_treated	2.639805126	6.01772411	3.376E-09	8.704E-07
NODE_137161_length_547_cov_27.166667_g85078_i0	Control	Salt_treated	2.638449664	2.021831743	0.0034124	0.0444843
NODE_116334_length_697_cov_38.048077_g67330_i0	Control	Salt_treated	2.63786764	2.619520267	1.093E-05	0.0007674
NODE_72036_length_1235_cov_23.829604_g37045_i0	Control	Salt_treated	2.630609846	2.011315825	0.001091	0.0208015
BINPACKER_10187_2	Control	Salt_treated	2.62511805	2.98879537	5.907E-07	7.127E-05
BINPACKER_401_1	Control	Salt_treated	2.621286395	2.241994291	0.0001643	0.0057982
BINPACKER_2716_8	Control	Salt_treated	2.611591858	9.9361531	4.697E-07	5.962E-05
NODE_119630_length_669_cov_33.956376_g70044_i0	Control	Salt_treated	2.610333667	2.231971605	0.0006987	0.0155728
Contig3716	Control	Salt_treated	2.606709148	4.72235325	2.315E-05	0.0013095
BINPACKER_1274_1	Control	Salt_treated	2.606386565	5.8820359	2.527E-08	4.952E-06
NODE_87128_length_1010_cov_22.696905_g30925_i1	Control	Salt_treated	2.60405705	1.294868746	0.0018258	0.0292743
NODE_19579_length_2775_cov_28.939674_g9337_i2	Control	Salt_treated	2.603414336	2.2517343	0.000118	0.0045774
NODE_77447_length_1147_cov_21.022346_g21017_i2	Control	Salt_treated	2.59727375	4.688059228	3.116E-06	0.0002816
BINPACKER_1935_2	Control	Salt_treated	2.590623319	2.394657093	0.0002683	0.0081828
NODE_92182_length_946_cov_19.521191_g49704_i0	Control	Salt_treated	2.589716447	3.055111698	0.0005843	0.0137621
Contig6996	Control	Salt_treated	2.588521662	10.24703819	2.58E-13	1.8E-10
NODE_71884_length_1237_cov_27.557560_g11942_i9	Control	Salt_treated	2.578233718	4.15024714	1.787E-05	0.0010913
BINPACKER_12853_5	Control	Salt_treated	2.577612109	4.473949617	6.112E-08	1.062E-05
NODE_192613_length_333_cov_26.642308_g136971_i0	Control	Salt_treated	2.577137567	1.98177899	0.000956	0.019162
NODE_74217_length_1199_cov_7.786856_g38319_i0	Control	Salt_treated	2.560646142	2.669178971	0.001065	0.0204768
Contig1430	Control	Salt_treated	2.555322405	2.340208734	0.0037012	0.046764
BINPACKER_23437_2	Control	Salt_treated	2.549634769	3.79124527	6.818E-06	0.0005302
NODE_8056_length_3857_cov_237.693446_g4058_i0	Control	Salt_treated	2.540059737	14.88226933	1.59E-12	9.274E-10
NODE_162819_length_424_cov_33.586895_g108629_i0	Control	Salt_treated	2.535817238	2.438801321	0.002488	0.0361471
BINPACKER_6848_1	Control	Salt_treated	2.521855369	2.84602322	0.000724	0.0158889
NODE_16206_length_3008_cov_22.704940_g8048_i0	Control	Salt_treated	2.515101053	2.114775906	0.0017707	0.0285542

NODE_102768_length_827_cov_30.193634_g56919_i0	Control	Salt_treated	2.501942161	1.563527609	0.0008419	0.0176402
NODE_74558_length_1193_cov_29.506250_g38535_i0	Control	Salt_treated	2.495160372	4.487226073	2.042E-06	0.0002042
BINPACKER_635_1	Control	Salt_treated	2.484832565	1.924509933	0.0012713	0.0230793
BINPACKER_4771_7	Control	Salt_treated	2.482803362	2.413054987	6.307E-05	0.0027887
BINPACKER_10217_1	Control	Salt_treated	2.481845007	5.224747981	5.965E-06	0.0004791
Contig878	Control	Salt_treated	2.479666636	3.561784668	0.0003298	0.0093184
BINPACKER_13630_3	Control	Salt_treated	2.470072266	2.85833058	0.0013784	0.024221
NODE_96383_length_896_cov_5.222357_g52532_i0	Control	Salt_treated	2.465800506	2.294308463	0.00137	0.0241605
NODE_79738_length_1112_cov_28.348412_g41258_i1	Control	Salt_treated	2.462921486	4.922386427	1.806E-09	4.862E-07
NODE_87058_length_1011_cov_21.768657_g46353_i0	Control	Salt_treated	2.461330428	2.102607124	0.0014727	0.0253142
BINPACKER_47497_1	Control	Salt_treated	2.453931616	2.111873376	0.0005329	0.0128869
BINPACKER_631_6	Control	Salt_treated	2.453149123	4.34297458	2.913E-08	5.619E-06
BINPACKER_1274_2	Control	Salt_treated	2.449605035	5.950132728	2.405E-09	6.403E-07
Contig1648	Control	Salt_treated	2.441741804	12.30997217	2.909E-11	1.371E-08
NODE_81901_length_1081_cov_17.897817_g43021_i0	Control	Salt_treated	2.439638082	2.929409028	6.945E-05	0.0029949
BINPACKER_17675_1	Control	Salt_treated	2.438548443	1.325575956	0.0039916	0.0489565
NODE_44104_length_1819_cov_26.987973_g8997_i5	Control	Salt_treated	2.437859817	6.841671557	7.227E-10	2.299E-07
NODE_16889_length_2955_cov_26.873005_g7139_i1	Control	Salt_treated	2.436184225	2.38635398	3.88E-05	0.001901
NODE_101436_length_841_cov_8.735677_g56001_i0	Control	Salt_treated	2.429476	2.06315778	0.0034979	0.0450824
NODE_69941_length_1272_cov_9.020851_g35796_i0	Control	Salt_treated	2.427271867	2.264713224	0.0013545	0.023973
BINPACKER_41932_1	Control	Salt_treated	2.424713467	4.162724553	1.944E-07	2.869E-05
NODE_44783_length_1801_cov_10.914352_g22436_i0	Control	Salt_treated	2.42049633	1.768591999	0.0006609	0.015079
BINPACKER_1208_1	Control	Salt_treated	2.413583826	3.084281423	0.0028902	0.0399977
BINPACKER_1208_10	Control	Salt_treated	2.413583826	3.084281423	0.0028902	0.0399977
NODE_36144_length_2051_cov_22.107685_g9688_i1	Control	Salt_treated	2.408544798	3.604109788	1E-06	0.0001114
NODE_52902_length_1607_cov_29.043025_g26285_i1	Control	Salt_treated	2.406042407	6.907504032	1.685E-05	0.0010568
NODE_67517_length_1314_cov_38.141015_g34411_i0	Control	Salt_treated	2.396229197	3.790055128	8.928E-05	0.0036571
NODE_102488_length_830_cov_29.013210_g56714_i0	Control	Salt_treated	2.39454373	2.95335316	2.804E-05	0.0015028
NODE_44830_length_1800_cov_11.867979_g22468_i0	Control	Salt_treated	2.388353321	1.59083667	0.0029728	0.0407715
Contig2144	Control	Salt_treated	2.386742643	2.247490193	0.0019212	0.0301286
NODE_57469_length_1510_cov_29.632568_g28978_i0	Control	Salt_treated	2.379781379	7.042753302	6.674E-07	7.898E-05
Contig1310	Control	Salt_treated	2.37842381	2.566457754	0.0004453	0.0113186
BINPACKER_7245_1	Control	Salt_treated	2.37377983	2.867170428	0.0017487	0.0282541
NODE_147876_length_489_cov_25.293269_g94746_i0	Control	Salt_treated	2.367376467	1.732244579	0.0041586	0.0502789
NODE_31348_length_2219_cov_23.300093_g15699_i0	Control	Salt_treated	2.36427051	2.878608763	3.131E-05	0.0016148
NODE_69323_length_1282_cov_21.373036_g35438_i0	Control	Salt_treated	2.363440128	1.598696258	0.0012453	0.0227814
NODE_153341_length_463_cov_24.617949_g99772_i0	Control	Salt_treated	2.356964235	2.374899905	0.0017304	0.028033
NODE_41632_length_1886_cov_26.305019_g20813_i0	Control	Salt_treated	2.352990128	2.385656078	0.0004897	0.0121662
NODE_17695_length_2892_cov_29.531394_g8791_i0	Control	Salt_treated	2.349304953	2.954811159	0.0017278	0.0280096
NODE_15365_length_3072_cov_30.894632_g7657_i0	Control	Salt_treated	2.346996918	7.625498057	1.738E-07	2.614E-05
NODE_44498_length_1808_cov_28.997695_g22281_i0	Control	Salt_treated	2.341553599	2.932037778	1.173E-05	0.0008045
Contig9252	Control	Salt_treated	2.339550128	1.705783505	0.0031903	0.0425572
NODE_43056_length_1848_cov_18.432113_g19866_i1	Control	Salt_treated	2.328674092	1.568417287	0.002008	0.0310978
NODE_97642_length_881_cov_25.956683_g53404_i0	Control	Salt_treated	2.32829781	2.790237629	0.0003139	0.0090027
NODE_111386_length_742_cov_29.046338_g60123_i4	Control	Salt_treated	2.325384435	6.478362108	2.314E-05	0.0013095
BINPACKER_18414_1	Control	Salt_treated	2.325210573	5.057880886	0.0004421	0.011288
NODE_109784_length_757_cov_27.314327_g62090_i0	Control	Salt_treated	2.321164221	1.709569439	0.0015361	0.0261119
Contig864	Control	Salt_treated	2.311904404	3.85683991	0.0002688	0.0081843
BINPACKER_8521_1	Control	Salt_treated	2.304547281	2.495459029	0.0024285	0.0356202
BINPACKER_3054_3	Control	Salt_treated	2.301322916	3.843892358	0.001383	0.0242562
NODE_85387_length_1034_cov_4.792924_g45276_i0	Control	Salt_treated	2.301309784	3.401092907	4.586E-07	5.851E-05
BINPACKER_18955_9	Control	Salt_treated	2.298936604	1.262729707	0.0030316	0.0411972
NODE_135177_length_559_cov_30.890947_g83308_i0	Control	Salt_treated	2.297323016	3.555136684	0.0009315	0.0188419
NODE_46315_length_1762_cov_31.595027_g23221_i0	Control	Salt_treated	2.282829211	5.61087203	7.904E-06	0.0005939
NODE_129776_length_593_cov_28.938462_g78719_i0	Control	Salt_treated	2.282109179	1.786257348	0.0039313	0.0484632
NODE_79022_length_1123_cov_30.629524_g41258_i0	Control	Salt_treated	2.278604221	3.386376995	7.92E-07	9.053E-05
NODE_67255_length_1319_cov_26.832263_g34252_i0	Control	Salt_treated	2.27459108	1.234985046	0.0040654	0.0494453
BINPACKER_869_4	Control	Salt_treated	2.272712777	3.421313652	6.975E-07	8.135E-05
NODE_129025_length_598_cov_28.796190_g78065_i0	Control	Salt_treated	2.266207799	1.641703563	0.0028582	0.039779
BINPACKER_14067_2	Control	Salt_treated	2.259579464	1.512301756	0.0031088	0.0417496
NODE_158145_length_443_cov_4.624324_g104246_i0	Control	Salt_treated	2.259325473	9.375968385	1.251E-09	3.737E-07
BINPACKER_31421_1	Control	Salt_treated	2.258760195	5.191084613	2.245E-05	0.0012878
NODE_9199_length_3685_cov_27.445183_g4643_i0	Control	Salt_treated	2.244077612	2.97424903	0.0004218	0.0109808
NODE_108942_length_765_cov_29.833815_g61435_i0	Control	Salt_treated	2.241417132	2.309019571	0.0009064	0.0185023
BINPACKER_21570_1	Control	Salt_treated	2.240999635	1.649725096	0.002052	0.0314937
BINPACKER_233_12	Control	Salt_treated	2.232201374	3.998053787	0.0003005	0.008784
NODE_81707_length_1084_cov_11.248269_g42899_i0	Control	Salt_treated	2.229317478	1.629010834	0.0020248	0.0311624
NODE_11602_length_3411_cov_32.740264_g5806_i0	Control	Salt_treated	2.228989942	4.810099938	5.212E-05	0.0023772
BINPACKER_9778_2	Control	Salt_treated	2.225609308	2.580656632	0.0002919	0.0086237
NODE_61499_length_1427_cov_25.813146_g31131_i0	Control	Salt_treated	2.224779699	2.821266314	0.0003068	0.0088714
BINPACKER_25771_2	Control	Salt_treated	2.216299966	2.482852697	0.0018059	0.029026
BINPACKER_49974_1	Control	Salt_treated	2.212899672	2.300182898	0.0001726	0.005988
BINPACKER_25747_3	Control	Salt_treated	2.212597114	2.112032796	0.0028239	0.0393686
Contig11597	Control	Salt_treated	2.211196228	4.489193276	0.000295	0.0086856
BINPACKER_6301_1	Control	Salt_treated	2.206560448	4.448062965	2.113E-06	0.0002095
NODE_83497_length_1059_cov_32.326572_g44030_i0	Control	Salt_treated	2.198826272	8.387887948	3.962E-06	0.000343
BINPACKER_2093_1	Control	Salt_treated	2.197805763	2.171942833	0.0043758	0.0524137
NODE_110511_length_750_cov_25.568685_g60013_i1	Control	Salt_treated	2.194576296	2.02100175	0.0010373	0.0200859
NODE_81144_length_1091_cov_25.738703_g42540_i0	Control	Salt_treated	2.193708169	3.638977655	0.0005911	0.0138945
NODE_76576_length_1161_cov_7.875919_g36156_i1	Control	Salt_treated	2.191819805	2.735592406	0.0009364	0.0188833
NODE_60280_length_1450_cov_27.898330_g19063_i2	Control	Salt_treated	2.190048547	3.723492771	6.22E-06	0.0004904
NODE_83339_length_1061_cov_32.520243_g43924_i0	Control	Salt_treated	2.189946457	2.33161914	0.0021529	0.032666
NODE_46181_length_1765_cov_32.359929_g8120_i2	Control	Salt_treated	2.188976704	3.732851456	2.132E-05	0.0012343
BINPACKER_875_4	Control	Salt_treated	2.185613061	2.492903308	0.0002719	0.0082311
BINPACKER_71226_4	Control	Salt_treated	2.183646045	2.694796679	4.512E-05	0.0021376
BINPACKER_80976_1	Control	Salt_treated	2.178495058	3.114570847	0.0004191	0.0109501
BINPACKER_6730_1	Control	Salt_treated	2.168371323	1.833855729	0.0013648	0.0241037
BINPACKER_283_11	Control	Salt_treated	2.155482523	3.359643753	3.538E-06	0.0003106
BINPACKER_1269_2	Control	Salt_treated	2.15531025	4.982398438	0.0010907	0.0208015
NODE_11777_length_3394_cov_15.750376_g5897_i0	Control	Salt_treated	2.150338726	1.805174621	0.0043824	0.0524664
NODE_53653_length_1590_cov_33.678312_g24033_i2	Control	Salt_treated	2.146355349	1.991204065	0.0011727	0.0218607
Contig6378	Control	Salt_treated	2.142618404	3.299849136	0.0011285	0.0213353
BINPACKER_24351_2	Control	Salt_treated	2.140410967	1.441540078	0.0040644	0.0494453
NODE_54692_length_1568_cov_24.355853_g27562_i0	Control	Salt_treated	2.136662389	4.626484621	9.3E-08	1.519E-05
NODE_93651_length_928_cov_29.556725_g50708_i0	Control	Salt_treated	2.131618298	3.773464856	0.0003224	0.0091936
NODE_89_length_12429_cov_28.028488_g43_i0	Control	Salt_treated	2.126935426	8.099982507	7.348E-07	8.49E-05
BINPACKER_55137_1	Control	Salt_treated	2.125034917	1.669105236	0.0046329	0.054603
NODE_44773_length_1801_cov_20.358796_g22428_i0	Control	Salt_treated	2.121453439	2.060068437	0.0041847	0.0504958
NODE_42632_length_1859_cov_27.138298_g21304_i0	Control	Salt_treated	2.117945494	2.301710233	0.0004238	0.0110086
NODE_173170_length_388_cov_23.101587_g118380_i0	Control	Salt_treated	2.114603636	1.958827687	0.0024647	0.0359398
NODE_37534_length_2006_cov_22.611485_g18823_i0	Control	Salt_treated	2.107164842	5.255114548	4.576E-07	5.851E-05
NODE_152431_length_467_cov_28.167513_g98932_i0	Control	Salt_treated	2.102175341	2.359514536	0.0011622	0.021731
NODE_47440_length_1736_cov_29.565845_g23787_i0	Control	Salt_treated	2.10152276	6.022740772	1.386E-05	0.0009153
NODE_95343_length_908_cov_23.638323_g51816_i0	Control	Salt_treated	2.088233011	3.30070402	0.0003291	0.0093082
NODE_65297_length_1354_cov_27.562061_g33181_i0	Control	Salt_treated	2.084039422	2.530061473	0.0030332	0.0411972
NODE_33825_length_2130_cov_33.540593_g16932_i0	Control	Salt_treated	2.077182939	3.351259082	0.0002814	0.0084412
NODE_46653_length_1755_cov_13.122473_g23394_i1	Control	Salt_treated	2.076805794	2.588989295	0.0013079	0.0234528
Contig7730	Control	Salt_treated	2.074885573	1.741608366	0.0026542	0.0377773
NODE_35567_length_2070_cov_22.103655_g17830_i0	Control	Salt_treated	2.073636486	2.42030472	0.0003111	0.0089341

NODE_101713_length_838_cov_24.220915_g56202_i0	Control	Salt_treated	2.072647424	1.852479889	0.0026319	0.0376151
NODE_80949_length_1094_cov_25.319295_g42408_i0	Control	Salt_treated	2.05754387	3.222384012	0.000664	0.015103
Contig12297	Control	Salt_treated	2.056806152	4.294141802	1.296E-07	2.035E-05
NODE_10112_length_3563_cov_34.381375_g5108_i0	Control	Salt_treated	2.055205845	10.8611747	1.726E-09	4.75E-07
NODE_56107_length_1539_cov_9.309686_g28292_i0	Control	Salt_treated	2.044594164	8.740920914	2.3E-08	4.623E-06
BINPACKER_6689_2	Control	Salt_treated	2.041004139	2.468284117	0.0019677	0.0306395
BINPACKER_50446_1	Control	Salt_treated	2.037327856	1.730178289	0.002697	0.0381614
Contig7942	Control	Salt_treated	2.035559296	15.97610414	9.547E-09	2.126E-06
NODE_14691_length_3122_cov_27.849787_g7360_i0	Control	Salt_treated	2.033259451	2.409107684	0.0016258	0.0270914
BINPACKER_19293_3	Control	Salt_treated	2.02720431	2.364614662	0.0028625	0.0398172
NODE_72117_length_1234_cov_18.043066_g37095_i0	Control	Salt_treated	2.017880012	1.712781237	0.002659	0.037801
NODE_89851_length_975_cov_16.662971_g39216_i1	Control	Salt_treated	2.015488191	3.075592101	0.0009873	0.0195029
NODE_19611_length_2773_cov_21.503704_g9780_i0	Control	Salt_treated	2.012924742	2.007434753	0.003016	0.0411574
NODE_19050_length_2809_cov_29.687500_g6389_i1	Control	Salt_treated	-2.010088407	5.53470392	5.586E-06	0.0004561
BINPACKER_1847_7	Control	Salt_treated	-2.014514172	3.168641979	5.153E-05	0.0023581
BINPACKER_2393_7	Control	Salt_treated	-2.016795892	2.943412282	0.0004347	0.0112006
Contig10511	Control	Salt_treated	-2.020428946	3.338886411	0.0023332	0.0346369
NODE_75996_length_1170_cov_39.439380_g39402_i0	Control	Salt_treated	-2.020632602	3.540016512	0.0005986	0.0140324
NODE_72630_length_1225_cov_30.328125_g1176_i7	Control	Salt_treated	-2.023990129	3.121260236	0.0002047	0.006784
Contig9304	Control	Salt_treated	-2.024958339	3.181987822	0.000226	0.0072738
NODE_30133_length_2265_cov_28.268704_g15118_i0	Control	Salt_treated	-2.02684093	3.566626693	0.0014102	0.0245268
NODE_38225_length_1984_cov_21.733647_g11558_i4	Control	Salt_treated	-2.027449798	2.464294901	0.0024556	0.0359245
BINPACKER_29097_3	Control	Salt_treated	-2.029422979	3.353548168	9.33E-05	0.0037525
Contig11613	Control	Salt_treated	-2.029917056	3.338452331	0.0002079	0.0068719
NODE_15339_length_3074_cov_31.451516_g7647_i0	Control	Salt_treated	-2.030550535	3.069308639	0.0001726	0.005988
Contig5314	Control	Salt_treated	-2.030822535	2.119290031	0.0026828	0.0380076
NODE_61833_length_1420_cov_22.317743_g31317_i0	Control	Salt_treated	-2.034932909	2.08116913	0.0039267	0.0484632
NODE_24430_length_2504_cov_11.279309_g9543_i2	Control	Salt_treated	-2.037048164	2.643828381	0.0026391	0.0376498
Contig8287	Control	Salt_treated	-2.037612905	3.818381143	3.099E-05	0.0016083
NODE_10756_length_3495_cov_34.616014_g5428_i0	Control	Salt_treated	-2.038441974	4.264562085	2.594E-05	0.0014087
NODE_24015_length_2525_cov_30.757749_g12037_i0	Control	Salt_treated	-2.03897922	4.372820106	3.271E-07	4.501E-05
NODE_31028_length_2231_cov_29.982391_g1851_i3	Control	Salt_treated	-2.039069682	4.101780049	1.088E-06	0.00012
NODE_32979_length_2161_cov_20.391284_g10641_i2	Control	Salt_treated	-2.03997468	1.984852362	0.0030572	0.0413057
NODE_59306_length_1470_cov_28.391553_g1750_i1	Control	Salt_treated	-2.040971213	2.813912304	0.0009456	0.0190171
NODE_4177_length_4756_cov_27.926543_g2112_i0	Control	Salt_treated	-2.043884611	3.477121806	0.0043395	0.0520801
NODE_66357_length_1335_cov_25.379556_g14548_i3	Control	Salt_treated	-2.046878606	2.423360904	0.0010366	0.0200859
NODE_20920_length_2693_cov_29.906489_g3138_i1	Control	Salt_treated	-2.047048025	3.977976076	2.375E-05	0.0013271
NODE_2329_length_5635_cov_27.150306_g1177_i0	Control	Salt_treated	-2.048722455	4.471728426	0.0001271	0.0048158
BINPACKER_26263_1	Control	Salt_treated	-2.049382172	2.721408425	0.0010779	0.020692
Contig11873	Control	Salt_treated	-2.050088153	2.516728796	0.0008716	0.0180015
NODE_14497_length_3138_cov_22.875367_g1757_i4	Control	Salt_treated	-2.052088362	2.812438991	0.0003853	0.0103039
NODE_36257_length_2047_cov_28.080041_g18187_i0	Control	Salt_treated	-2.053654402	5.349180692	1.149E-05	0.0007903
BINPACKER_16462_2	Control	Salt_treated	-2.056816395	3.17929426	4.838E-05	0.0022574
Contig11187	Control	Salt_treated	-2.063819517	2.159841433	0.0018802	0.0297516
BINPACKER_241_11	Control	Salt_treated	-2.065782904	3.970474753	0.0003054	0.0088584
BINPACKER_10087_2	Control	Salt_treated	-2.067105848	2.916694191	0.0003913	0.0104289
NODE_29796_length_2277_cov_29.278131_g9351_i2	Control	Salt_treated	-2.067706915	2.504569285	0.000917	0.0186708
BINPACKER_33793_1	Control	Salt_treated	-2.068283197	3.988699186	7.709E-06	0.0005864
NODE_43838_length_1827_cov_9.171608_g21943_i0	Control	Salt_treated	-2.07026334	3.223173326	7.741E-05	0.0032694
Contig10797	Control	Salt_treated	-2.07087325	3.562277966	0.0009239	0.0187272
BINPACKER_14076_1	Control	Salt_treated	-2.072139994	2.132026781	0.0018905	0.0298757
NODE_2042_length_5807_cov_34.179456_g807_i2	Control	Salt_treated	-2.07484035	5.371859235	1.571E-05	0.0010019
BINPACKER_17742_5	Control	Salt_treated	-2.078498925	2.544391784	0.0039933	0.0489565
NODE_5566_length_4348_cov_24.456608_g2829_i0	Control	Salt_treated	-2.080152445	2.350909719	0.001335	0.0237127
BINPACKER_3598_2	Control	Salt_treated	-2.081764218	3.082175634	8.087E-05	0.0033815
NODE_6437_length_4146_cov_25.404861_g3262_i0	Control	Salt_treated	-2.08378774	2.052608931	0.003132	0.0419686
NODE_5112_length_4470_cov_30.201046_g2607_i0	Control	Salt_treated	-2.101415823	3.168608065	2.913E-05	0.0015348
NODE_2212_length_5721_cov_33.823831_g1122_i1	Control	Salt_treated	-2.105679387	2.557863927	0.0005622	0.0134342
NODE_56287_length_1535_cov_24.850889_g23078_i1	Control	Salt_treated	-2.10844485	3.067026901	0.001454	0.0251344
BINPACKER_3603_18	Control	Salt_treated	-2.11092875	5.803194063	5.765E-05	0.0026007
NODE_68424_length_1298_cov_16.053878_g34932_i0	Control	Salt_treated	-2.114243971	3.809145948	2.495E-05	0.0013702
BINPACKER_5704_2	Control	Salt_treated	-2.115377123	2.629210544	0.0024158	0.0355185
BINPACKER_1177_8	Control	Salt_treated	-2.115951773	8.685250525	8.249E-10	2.59E-07
BINPACKER_1807_8	Control	Salt_treated	-2.117390771	3.616915119	3.404E-06	0.0003032
BINPACKER_4169_6	Control	Salt_treated	-2.119417798	2.586285477	0.0003425	0.009569
NODE_11280_length_3445_cov_19.795077_g5647_i0	Control	Salt_treated	-2.121999779	2.434099111	0.001303	0.0233816
NODE_4444_length_4669_cov_36.006527_g2256_i0	Control	Salt_treated	-2.125364532	3.317775558	9.148E-05	0.0037101
NODE_85657_length_1030_cov_26.378265_g45451_i0	Control	Salt_treated	-2.125916574	1.929129756	0.0025376	0.0366712
BINPACKER_20230_2	Control	Salt_treated	-2.12941498	3.26794201	1.296E-05	0.0008604
BINPACKER_9342_7	Control	Salt_treated	-2.13051259	3.948675382	0.0022609	0.0338926
NODE_56065_length_1539_cov_38.900409_g28278_i0	Control	Salt_treated	-2.132817688	3.70763824	1.651E-05	0.001042
NODE_4725_length_4590_cov_24.807173_g1300_i1	Control	Salt_treated	-2.135285944	3.896378302	1.293E-05	0.0008604
NODE_12756_length_3296_cov_23.668321_g5923_i1	Control	Salt_treated	-2.140649446	4.165332983	3.111E-05	0.0016112
Contig8874	Control	Salt_treated	-2.140720663	2.51052696	0.0006238	0.0145103
Contig541	Control	Salt_treated	-2.142649829	2.351303685	0.001281	0.0231402
BINPACKER_2253_1	Control	Salt_treated	-2.144463995	5.360257307	1.578E-09	4.393E-07
Contig8313	Control	Salt_treated	-2.146640723	2.495001227	0.0009366	0.0188833
NODE_27011_length_2387_cov_35.974503_g13547_i0	Control	Salt_treated	-2.15335487	1.775822755	0.0032411	0.0430306
NODE_43266_length_1842_cov_21.886942_g1993_i2	Control	Salt_treated	-2.154009767	2.537972028	0.0002943	0.0086758
Contig9002	Control	Salt_treated	-2.156108976	3.38093968	6.551E-06	0.000511
BINPACKER_36669_1	Control	Salt_treated	-2.15945619	2.831143146	0.0004951	0.0122533
NODE_107542_length_779_cov_24.379603_g60355_i0	Control	Salt_treated	-2.165588586	2.293412848	0.0017375	0.0281113
NODE_6137_length_4206_cov_32.436245_g3109_i0	Control	Salt_treated	-2.169342963	2.400236021	0.0012735	0.0230793
BINPACKER_7449_11	Control	Salt_treated	-2.171359265	3.943307324	6.735E-05	0.0029201
Contig6136	Control	Salt_treated	-2.174392512	2.710133077	0.0002862	0.0085181
NODE_89116_length_984_cov_31.419319_g47672_i0	Control	Salt_treated	-2.174397973	4.781206757	4.122E-09	1.039E-06
NODE_10119_length_3563_cov_27.283381_g5113_i0	Control	Salt_treated	-2.177195402	2.726622288	0.0002837	0.0084643
NODE_38368_length_1979_cov_31.081322_g19236_i0	Control	Salt_treated	-2.177733971	3.21869467	0.0005449	0.0131233
BINPACKER_3264_2	Control	Salt_treated	-2.178542883	5.344019556	3.9E-08	7.349E-06
NODE_14640_length_3126_cov_31.782509_g7337_i0	Control	Salt_treated	-2.187156776	1.822323046	0.0033122	0.0435964
NODE_145609_length_500_cov_52.327869_g92656_i0	Control	Salt_treated	-2.187311575	3.673460839	5.476E-06	0.0004516
NODE_14142_length_3167_cov_22.212670_g7082_i0	Control	Salt_treated	-2.188679183	1.800383129	0.0040022	0.0489735
NODE_26806_length_2396_cov_29.857942_g13442_i0	Control	Salt_treated	-2.198399281	2.266890025	0.0024846	0.0361408
BINPACKER_13766_3	Control	Salt_treated	-2.199317217	5.721503591	5.215E-08	9.463E-06
NODE_2945_length_5256_cov_31.076211_g257_i4	Control	Salt_treated	-2.199437721	3.187715229	1.782E-05	0.0010911
NODE_107160_length_783_cov_28.885915_g60065_i0	Control	Salt_treated	-2.199761725	5.281513265	1.354E-07	2.113E-05
NODE_99489_length_860_cov_26.058450_g43621_i2	Control	Salt_treated	-2.203831929	3.676414748	2.676E-06	0.0002503
Contig8243	Control	Salt_treated	-2.207821435	6.175665496	0.0004281	0.0110852
BINPACKER_6041_11	Control	Salt_treated	-2.209652364	2.110494033	0.0020984	0.0320448
NODE_57088_length_1518_cov_26.829066_g28498_i1	Control	Salt_treated	-2.210141658	2.851247639	0.0010213	0.0199187
BINPACKER_1906_12	Control	Salt_treated	-2.212026609	2.126500813	0.002268	0.0339361
BINPACKER_10372_13	Control	Salt_treated	-2.213163398	2.216115777	0.001161	0.0217246
Contig6526	Control	Salt_treated	-2.216742063	3.588266436	1.824E-05	0.0011112
Contig2101	Control	Salt_treated	-2.220760138	4.819634009	4E-08	7.478E-06
Contig3420	Control	Salt_treated	-2.220994208	2.827908292	8.99E-05	0.0036701
NODE_14272_length_3157_cov_23.680934_g7150_i0	Control	Salt_treated	-2.223120745	2.995681362	0.000435	0.0112006
NODE_61672_length_1423_cov_27.291111_g13011_i3	Control	Salt_treated	-2.22546075	2.649570978	0.0004011	0.0106329
Contig4276	Control	Salt_treated	-2.226616642	3.712619015	7.946E-07	9.053E-05
BINPACKER_7853_4	Control	Salt_treated	-2.23210989	4.078834537	1.776E-06	0.0001812

BINPACKER_10786_4	Control	Salt_treated	-2.23400745	2.839461271	0.0031249	0.0418964
BINPACKER_11849_1	Control	Salt_treated	-2.236971196	1.790873472	0.0037495	0.0470514
NODE_17376_length_2917_cov_26.476793_g8621_i0	Control	Salt_treated	-2.237008441	3.055038901	0.0021257	0.0323415
BINPACKER_17773_3	Control	Salt_treated	-2.237165427	4.861966356	8.586E-08	1.431E-05
NODE_42509_length_1862_cov_27.155953_g21236_i0	Control	Salt_treated	-2.237808748	3.028041055	3.266E-05	0.0016701
NODE_89823_length_975_cov_29.166297_g48136_i0	Control	Salt_treated	-2.238510972	4.351650972	8.924E-07	9.981E-05
NODE_64849_length_1362_cov_37.215671_g2133_i1	Control	Salt_treated	-2.238576845	3.371573009	9.08E-06	0.0006679
Contig8298	Control	Salt_treated	-2.240957447	2.551010671	0.0007019	0.0156159
BINPACKER_9581_2	Control	Salt_treated	-2.242829814	2.194331055	0.0018261	0.0292743
BINPACKER_224_2	Control	Salt_treated	-2.24595328	6.368745349	5.878E-10	1.894E-07
BINPACKER_26810_1	Control	Salt_treated	-2.24602831	6.103489606	8.879E-05	0.0036492
Contig10106	Control	Salt_treated	-2.24621139	2.698476052	8.006E-05	0.003364
NODE_38017_length_1990_cov_32.582160_g14991_i1	Control	Salt_treated	-2.248861229	4.717583403	3.587E-05	0.0018002
NODE_39723_length_1939_cov_21.869775_g19908_i0	Control	Salt_treated	-2.249524339	4.218739133	6.675E-08	1.135E-05
BINPACKER_8140_1	Control	Salt_treated	-2.250892667	2.479401988	0.000447	0.0113469
BINPACKER_8134_4	Control	Salt_treated	-2.250969545	2.369024208	0.0018355	0.0292981
NODE_4602_length_4628_cov_29.942481_g2334_i0	Control	Salt_treated	-2.257066725	5.587525751	3.46E-05	0.0017474
NODE_10502_length_3522_cov_30.229052_g5311_i0	Control	Salt_treated	-2.259836055	3.854514513	3.917E-05	0.0019076
BINPACKER_8565_1	Control	Salt_treated	-2.26031903	2.329070388	0.0031793	0.0424632
BINPACKER_16243_1	Control	Salt_treated	-2.26185998	1.8358619	0.0024617	0.0359398
Contig3017	Control	Salt_treated	-2.266527693	2.812698272	4.213E-05	0.0020273
NODE_30607_length_2247_cov_26.385005_g15344_i0	Control	Salt_treated	-2.267630346	2.311754228	0.0012082	0.0223692
NODE_11246_length_3448_cov_25.122074_g5628_i0	Control	Salt_treated	-2.273331459	4.810682821	0.0002732	0.0082516
BINPACKER_5679_4	Control	Salt_treated	-2.274962161	2.22356522	0.0023405	0.0346828
BINPACKER_375_7	Control	Salt_treated	-2.288290653	4.468337119	8.635E-06	0.000641
Contig9474	Control	Salt_treated	-2.288651366	3.86248288	2.672E-07	3.828E-05
Contig8570	Control	Salt_treated	-2.295051604	4.471954017	6E-08	1.062E-05
BINPACKER_20472_1	Control	Salt_treated	-2.29846948	4.289884259	2.114E-05	0.0012343
Contig3707	Control	Salt_treated	-2.300637734	2.173398481	0.0010136	0.0198127
NODE_38022_length_1990_cov_29.664580_g19069_i0	Control	Salt_treated	-2.301587934	2.519039605	0.0002895	0.0085761
NODE_18601_length_2837_cov_33.042330_g9265_i0	Control	Salt_treated	-2.302182214	2.849698688	0.0005052	0.0124035
NODE_29650_length_2282_cov_33.137619_g14886_i0	Control	Salt_treated	-2.306191166	3.80825294	8.447E-07	9.52E-05
NODE_27962_length_2347_cov_23.562005_g14038_i0	Control	Salt_treated	-2.30962619	4.441834787	1.422E-08	3.083E-06
NODE_4125_length_4781_cov_24.155055_g2082_i0	Control	Salt_treated	-2.315048594	3.825900833	5.274E-07	6.592E-05
NODE_53142_length_1602_cov_15.655984_g26729_i0	Control	Salt_treated	-2.3160668	3.310884564	1.109E-05	0.000771
BINPACKER_182_10	Control	Salt_treated	-2.317734131	3.405189062	5.82E-06	0.0004721
NODE_37260_length_2014_cov_25.878928_g18688_i0	Control	Salt_treated	-2.319420136	3.45283968	9.095E-05	0.003697
BINPACKER_8095_7	Control	Salt_treated	-2.329342188	2.675675368	0.0015726	0.0265064
NODE_63174_length_1393_cov_38.174242_g32035_i0	Control	Salt_treated	-2.33150369	2.14815532	0.0020131	0.031111
NODE_8149_length_3844_cov_22.826836_g4104_i0	Control	Salt_treated	-2.332314155	3.971777502	1.27E-07	2.007E-05
NODE_6290_length_4175_cov_30.597513_g3185_i0	Control	Salt_treated	-2.335440713	4.108913888	4.365E-06	0.0003754
NODE_50465_length_1665_cov_11.616206_g25319_i0	Control	Salt_treated	-2.337243745	1.885032194	0.0033445	0.0439
NODE_63566_length_1386_cov_24.827113_g28957_i1	Control	Salt_treated	-2.338551198	3.16949746	0.0001694	0.005911
Contig5232	Control	Salt_treated	-2.34497133	2.283723773	0.003647	0.0463582
BINPACKER_17910_1	Control	Salt_treated	-2.347355818	3.153183713	1.879E-05	0.0011336
NODE_17897_length_2879_cov_32.516393_g8889_i0	Control	Salt_treated	-2.350374345	3.697201075	3.321E-06	0.000298
BINPACKER_21293_3	Control	Salt_treated	-2.350423824	4.564168063	7.57E-06	0.0005795
NODE_46843_length_1750_cov_26.189028_g23498_i0	Control	Salt_treated	-2.351664461	2.027845068	0.0038828	0.0481566
NODE_8474_length_3789_cov_28.317815_g3752_i1	Control	Salt_treated	-2.352068729	2.057525853	0.0009146	0.0186377
NODE_77102_length_1152_cov_26.779425_g40066_i0	Control	Salt_treated	-2.352546743	2.859075172	0.0013462	0.023878
NODE_16015_length_3023_cov_29.319322_g7962_i0	Control	Salt_treated	-2.369906582	2.523469486	0.0009873	0.0195029
NODE_119756_length_668_cov_35.492437_g65142_i8	Control	Salt_treated	-2.37022282	2.231214393	0.0003078	0.0088795
BINPACKER_240_2	Control	Salt_treated	-2.372940548	3.800548031	0.0016893	0.027586
BINPACKER_12352_1	Control	Salt_treated	-2.373077709	2.472900666	0.000122	0.00469
BINPACKER_475_15	Control	Salt_treated	-2.37711826	1.744540109	0.0025929	0.037202
NODE_12866_length_3286_cov_28.122316_g6446_i0	Control	Salt_treated	-2.377692665	1.921019559	0.0012724	0.0230793
BINPACKER_47578_1	Control	Salt_treated	-2.381235686	2.053894639	0.0011801	0.0219496
BINPACKER_15159_4	Control	Salt_treated	-2.385083004	5.109322114	8.584E-06	0.0006391
NODE_41412_length_1893_cov_16.317033_g20661_i1	Control	Salt_treated	-2.386133953	1.722352694	0.002654	0.0377773
NODE_94058_length_923_cov_29.911765_g50973_i0	Control	Salt_treated	-2.387621359	2.419889296	0.002793	0.0391164
NODE_24292_length_2510_cov_32.297907_g2138_i4	Control	Salt_treated	-2.388536693	2.518382402	9.101E-05	0.003697
NODE_130819_length_586_cov_36.072125_g79623_i0	Control	Salt_treated	-2.401482009	2.070141928	0.0010265	0.0199716
NODE_136328_length_552_cov_31.597077_g84333_i0	Control	Salt_treated	-2.40358236	2.722456879	0.0025505	0.0368134
Contig6487	Control	Salt_treated	-2.406373146	3.088721063	6.017E-06	0.0004816
NODE_83902_length_1053_cov_37.536735_g44298_i0	Control	Salt_treated	-2.408130063	3.871550334	6.089E-08	1.062E-05
Contig8679	Control	Salt_treated	-2.409926608	2.421593727	0.0001563	0.0055828
NODE_8467_length_3791_cov_24.601399_g1835_i1	Control	Salt_treated	-2.415657472	2.940241321	0.0001686	0.005892
NODE_5619_length_4331_cov_31.106858_g2847_i0	Control	Salt_treated	-2.418705153	2.857682919	0.0003054	0.0088584
BINPACKER_21498_1	Control	Salt_treated	-2.432937975	5.078070225	0.0002378	0.0075535
NODE_71890_length_1237_cov_26.051546_g22842_i2	Control	Salt_treated	-2.438453652	5.127599731	0.000801	0.0169868
BINPACKER_27363_1	Control	Salt_treated	-2.444600655	2.732181176	3.414E-05	0.0017348
Contig590	Control	Salt_treated	-2.450587495	2.380818705	0.0014931	0.0255156
BINPACKER_31763_1	Control	Salt_treated	-2.458818371	2.642477751	0.0003745	0.0101374
NODE_3056_length_5194_cov_33.111502_g1567_i0	Control	Salt_treated	-2.46385886	3.914536534	6.605E-05	0.0028736
NODE_19703_length_2767_cov_29.785820_g9823_i1	Control	Salt_treated	-2.470042339	4.407650343	2.6E-08	5.064E-06
BINPACKER_20985_1	Control	Salt_treated	-2.470623583	4.75023754	4.483E-06	0.000384
BINPACKER_21140_1	Control	Salt_treated	-2.475508003	2.672446163	6.33E-05	0.0027939
BINPACKER_8732_6	Control	Salt_treated	-2.477731967	3.581677377	1.449E-05	0.0009396
NODE_92990_length_936_cov_20.132097_g12169_i1	Control	Salt_treated	-2.477777905	2.416518587	0.0002429	0.0076485
NODE_69398_length_1280_cov_32.965203_g35485_i0	Control	Salt_treated	-2.478676665	3.031626341	5.527E-06	0.0004528
Contig2363	Control	Salt_treated	-2.480206211	2.243313997	0.0010308	0.0200073
NODE_32601_length_2174_cov_39.153260_g16309_i0	Control	Salt_treated	-2.480772763	6.202043603	0.0007065	0.0156757
NODE_43251_length_1842_cov_30.750141_g21629_i0	Control	Salt_treated	-2.484128291	3.423501153	1.335E-06	0.0001441
NODE_18736_length_2829_cov_25.564224_g9340_i0	Control	Salt_treated	-2.487142435	3.996908906	2.297E-06	0.0002207
Contig8766	Control	Salt_treated	-2.498115602	2.975586278	0.0005267	0.012798
NODE_23528_length_2551_cov_24.234867_g1022_i1	Control	Salt_treated	-2.500311383	2.460838811	0.0039976	0.0489735
BINPACKER_10190_2	Control	Salt_treated	-2.504725068	5.287196901	2.146E-08	4.381E-06
BINPACKER_9115_2	Control	Salt_treated	-2.506970275	2.79617823	1.973E-05	0.0011759
NODE_58244_length_1494_cov_28.108374_g29402_i0	Control	Salt_treated	-2.509704935	1.862581287	0.0009641	0.0192782
Contig3130	Control	Salt_treated	-2.514182161	5.512908026	6.48E-08	1.11E-05
NODE_32819_length_2166_cov_29.662685_g16414_i0	Control	Salt_treated	-2.524054154	2.346545783	0.0001379	0.0051152
NODE_62150_length_1413_cov_28.052239_g31494_i0	Control	Salt_treated	-2.525130583	1.830569011	0.0008698	0.0179802
BINPACKER_22412_2	Control	Salt_treated	-2.526561029	2.191490709	0.0004203	0.0109635
BINPACKER_2797_12	Control	Salt_treated	-2.527153463	1.648702685	0.002049	0.0314672
NODE_48344_length_1715_cov_26.291108_g24060_i1	Control	Salt_treated	-2.529188328	2.574787285	8.125E-05	0.0033847
NODE_19622_length_2772_cov_22.017414_g1560_i5	Control	Salt_treated	-2.538331461	3.430447524	2.44E-06	0.0002326
NODE_37339_length_2012_cov_25.046416_g1990_i2	Control	Salt_treated	-2.539470821	2.486898233	4.735E-05	0.0022264
Contig1844	Control	Salt_treated	-2.544807688	2.625343055	4.309E-05	0.0020574
NODE_48763_length_1705_cov_30.152574_g24438_i0	Control	Salt_treated	-2.545878481	3.554625657	8.819E-08	1.45E-05
NODE_25509_length_2453_cov_25.831092_g12807_i0	Control	Salt_treated	-2.548598229	2.512734218	4.864E-05	0.002265
BINPACKER_618_1	Control	Salt_treated	-2.55222466	2.517201153	0.0029487	0.0405784
NODE_13398_length_3233_cov_24.319620_g6735_i0	Control	Salt_treated	-2.55431092	3.115615983	0.0002164	0.0070486
NODE_77034_length_1153_cov_25.269444_g30003_i2	Control	Salt_treated	-2.554777104	2.631577303	2.125E-05	0.0012343
BINPACKER_4244_3	Control	Salt_treated	-2.555673756	2.124325735	0.0014879	0.0254866
BINPACKER_5641_6	Control	Salt_treated	-2.55784633	4.880748511	1.838E-08	3.816E-06
NODE_56911_length_1522_cov_28.762595_g28691_i0	Control	Salt_treated	-2.560690312	3.137726476	1.411E-06	0.0001509
NODE_30313_length_2258_cov_29.632037_g15205_i0	Control	Salt_treated	-2.562940211	5.208121308	0.0015864	0.0266154
NODE_52986_length_1605_cov_27.009138_g26648_i0	Control	Salt_treated	-2.567374751	1.968205082	0.0033234	0.0436969
BINPACKER_16949_2	Control	Salt_treated	-2.569694972	1.617174906	0.0030623	0.041329

NODE_218_length_10247_cov_31.183900_g113_i0	Control	Salt_treated	-2.574659549	4.088536724	4.482E-07	5.806E-05
NODE_91831_length_950_cov_21.492588_g49470_i2	Control	Salt_treated	-2.574737127	2.038463071	0.0008843	0.0181878
NODE_79675_length_1113_cov_24.308654_g41646_i0	Control	Salt_treated	-2.577231856	2.114526539	0.0004691	0.0117723
NODE_98249_length_874_cov_26.580524_g53840_i0	Control	Salt_treated	-2.579692428	2.408775163	0.0001574	0.0056109
NODE_56700_length_1526_cov_30.163111_g28586_i0	Control	Salt_treated	-2.582104561	2.541817298	0.0024261	0.0356072
BINPACKER_7271_3	Control	Salt_treated	-2.585472723	2.053334187	0.0004419	0.011288
NODE_16201_length_3008_cov_30.939693_g3770_i4	Control	Salt_treated	-2.588192959	2.160745489	0.0032067	0.0426662
NODE_7064_length_4021_cov_32.446302_g3576_i0	Control	Salt_treated	-2.588520656	1.695335593	0.001525	0.0259772
NODE_40653_length_1912_cov_31.511147_g1560_i6	Control	Salt_treated	-2.597990982	1.429824115	0.0025219	0.0365097
Contig5140	Control	Salt_treated	-2.599857616	2.063285656	0.0011309	0.0213418
NODE_4611_length_4625_cov_26.815466_g959_i2	Control	Salt_treated	-2.601947802	3.364664278	5.731E-05	0.00259
NODE_131582_length_581_cov_40.011811_g80282_i0	Control	Salt_treated	-2.602856694	3.510088027	8.349E-08	1.401E-05
NODE_1721_length_6111_cov_29.891189_g321_i3	Control	Salt_treated	-2.60429224	1.889116909	0.0012324	0.0226478
BINPACKER_55596_1	Control	Salt_treated	-2.606092453	2.447367325	0.0019418	0.030315
NODE_8769_length_3743_cov_30.690191_g4422_i0	Control	Salt_treated	-2.606503398	1.432787413	0.0031916	0.0425572
BINPACKER_11220_1	Control	Salt_treated	-2.612231013	2.258446974	0.0001464	0.0053498
Contig10002	Control	Salt_treated	-2.621128227	1.719391372	0.0014038	0.0244911
BINPACKER_22448_1	Control	Salt_treated	-2.622706576	1.646227283	0.0043536	0.0521726
BINPACKER_12411_2	Control	Salt_treated	-2.638015565	2.690727951	0.0020241	0.0311624
NODE_30996_length_2232_cov_32.053265_g15531_i0	Control	Salt_treated	-2.639754209	4.404233112	7.094E-06	0.0005464
BINPACKER_6343_9	Control	Salt_treated	-2.642095544	2.760451824	0.0001417	0.0052443
NODE_106553_length_789_cov_31.800279_g18826_i2	Control	Salt_treated	-2.647025513	2.593500795	0.0017371	0.0281113
NODE_58907_length_1480_cov_22.266525_g26573_i1	Control	Salt_treated	-2.65344282	2.065974287	0.0012489	0.0228048
BINPACKER_6432_12	Control	Salt_treated	-2.659429704	2.481229549	0.0023998	0.0353558
BINPACKER_28941_2	Control	Salt_treated	-2.660493131	1.746600185	0.001172	0.0218607
BINPACKER_11033_2	Control	Salt_treated	-2.668574063	2.482320617	5.913E-05	0.0026382
NODE_2409_length_5571_cov_28.455075_g779_i1	Control	Salt_treated	-2.676134604	4.865877033	0.0002199	0.007125
NODE_26060_length_2428_cov_29.766030_g9710_i3	Control	Salt_treated	-2.68038294	1.909584742	0.0012922	0.0232393
BINPACKER_15835_4	Control	Salt_treated	-2.680873128	2.91769994	6.119E-06	0.000488
BINPACKER_9400_1	Control	Salt_treated	-2.692994181	2.496928933	0.0003255	0.0092392
NODE_23493_length_2553_cov_27.755242_g11735_i0	Control	Salt_treated	-2.695540312	3.414987054	1.193E-05	0.0008079
NODE_17924_length_2878_cov_31.997861_g8902_i0	Control	Salt_treated	-2.699200097	1.45863358	0.0040026	0.0489735
NODE_98777_length_868_cov_21.090566_g54202_i0	Control	Salt_treated	-2.704967426	3.257401302	7.393E-07	8.502E-05
NODE_29480_length_2289_cov_26.551444_g10541_i1	Control	Salt_treated	-2.727243529	4.465401692	5.89E-05	0.002633
Contig12375	Control	Salt_treated	-2.728751376	5.10697455	1.552E-06	0.0001638
NODE_190309_length_339_cov_34.932331_g134729_i0	Control	Salt_treated	-2.730630321	1.947127017	0.0014362	0.0248935
NODE_3954_length_4848_cov_26.911623_g1991_i0	Control	Salt_treated	-2.743749965	6.421304841	1.141E-05	0.0007875
NODE_98518_length_871_cov_29.195489_g54030_i0	Control	Salt_treated	-2.744677698	3.626601357	0.0029378	0.0404733
NODE_24319_length_2509_cov_28.167077_g12200_i0	Control	Salt_treated	-2.74572374	2.357834832	0.0001083	0.0042427
BINPACKER_7226_16	Control	Salt_treated	-2.74863634	2.297914605	0.0008214	0.0172992
Contig8808	Control	Salt_treated	-2.749343084	2.010420082	0.0031077	0.0417496
Contig1151	Control	Salt_treated	-2.749643165	3.449814158	2.044E-05	0.0012036
NODE_5798_length_4289_cov_26.403463_g2737_i2	Control	Salt_treated	-2.750981273	5.738023356	4.686E-12	2.392E-09
NODE_20237_length_2736_cov_30.128051_g5547_i2	Control	Salt_treated	-2.75535703	3.283344046	0.0025942	0.037202
BINPACKER_7417_8	Control	Salt_treated	-2.757145814	2.568061972	0.000786	0.0167616
NODE_15263_length_3080_cov_36.650482_g7612_i0	Control	Salt_treated	-2.758954716	2.88617361	0.0012025	0.0222817
BINPACKER_1885_9	Control	Salt_treated	-2.768137471	3.070499815	0.0016355	0.0271606
BINPACKER_10038_4	Control	Salt_treated	-2.76885719	2.604243569	9.243E-05	0.0037266
BINPACKER_43327_1	Control	Salt_treated	-2.783984239	1.99008877	0.0006868	0.0154767
BINPACKER_4972_1	Control	Salt_treated	-2.795978709	1.538470839	0.0013757	0.0242028
NODE_30616_length_2246_cov_29.046940_g11643_i2	Control	Salt_treated	-2.797023988	1.599715908	0.0018105	0.0290617
NODE_2063_length_5793_cov_25.525699_g456_i2	Control	Salt_treated	-2.800713189	3.466908381	6.357E-06	0.0004991
BINPACKER_2379_3	Control	Salt_treated	-2.80133773	1.236532296	0.0028487	0.0396694
NODE_10464_length_3526_cov_23.673328_g5292_i0	Control	Salt_treated	-2.805415373	1.880861963	0.0004563	0.0114931
BINPACKER_9419_2	Control	Salt_treated	-2.807229206	3.133485289	1.866E-07	2.771E-05
Contig3784	Control	Salt_treated	-2.813154554	3.401885544	0.0004756	0.0118988
BINPACKER_9271_1	Control	Salt_treated	-2.820780321	2.829661398	1.872E-06	0.0001895
Contig3905	Control	Salt_treated	-2.832459315	4.910971172	3.534E-13	2.278E-10
NODE_32240_length_2188_cov_26.717730_g16133_i0	Control	Salt_treated	-2.838342337	3.272646234	6.058E-08	1.062E-05
BINPACKER_27106_2	Control	Salt_treated	-2.847297059	1.867331342	0.0004193	0.0109501
NODE_8627_length_3765_cov_30.060943_g2711_i1	Control	Salt_treated	-2.848023775	1.868179593	0.0003389	0.0095148
Contig3039	Control	Salt_treated	-2.852782069	2.590562984	2.356E-05	0.0013266
BINPACKER_5018_7	Control	Salt_treated	-2.852852439	2.08759337	0.0040759	0.0494674
NODE_6028_length_4232_cov_28.273623_g3052_i0	Control	Salt_treated	-2.85838972	2.509327824	0.00133	0.0236934
BINPACKER_4574_2	Control	Salt_treated	-2.86054312	3.301611047	0.0012685	0.0230667
NODE_79123_length_1122_cov_21.135367_g121_i19	Control	Salt_treated	-2.860927922	1.273899543	0.0025186	0.0365097
BINPACKER_1958_17	Control	Salt_treated	-2.865694221	2.235498315	0.0003277	0.0092892
NODE_41761_length_1883_cov_18.060221_g14596_i3	Control	Salt_treated	-2.871980911	1.346505097	0.0027302	0.0384347
NODE_69162_length_1285_cov_22.133663_g35349_i0	Control	Salt_treated	-2.877039711	3.555696096	3.386E-07	4.583E-05
NODE_38541_length_1973_cov_35.915789_g19319_i0	Control	Salt_treated	-2.87720459	4.160581681	0.0011578	0.0216878
NODE_8376_length_3806_cov_28.022770_g4212_i0	Control	Salt_treated	-2.884118918	1.594440107	0.0021537	0.032666
NODE_43807_length_1827_cov_33.095211_g21923_i0	Control	Salt_treated	-2.885603922	2.536865376	0.0001324	0.0049583
NODE_60712_length_1442_cov_25.894083_g30689_i0	Control	Salt_treated	-2.888504099	3.542108598	0.0037641	0.0471859
NODE_84217_length_1049_cov_19.094262_g44489_i0	Control	Salt_treated	-2.889576408	1.300171925	0.002506	0.0363873
BINPACKER_555_10	Control	Salt_treated	-2.893780534	5.500432962	2.071E-06	0.0002062
BINPACKER_2019_5	Control	Salt_treated	-2.900472072	2.547311902	0.0008175	0.0172635
Contig11111	Control	Salt_treated	-2.917520316	1.873716662	0.0008672	0.0179802
NODE_74070_length_1201_cov_23.326241_g34390_i1	Control	Salt_treated	-2.919984963	1.771713261	0.0029187	0.0402785
BINPACKER_4729_14	Control	Salt_treated	-2.924087169	1.696527666	0.000572	0.0136032
BINPACKER_12252_2	Control	Salt_treated	-2.929430596	3.755158844	1.159E-08	2.536E-06
NODE_93582_length_929_cov_19.250000_g50657_i0	Control	Salt_treated	-2.930051859	2.511294032	0.0034944	0.0450824
NODE_11233_length_3450_cov_26.435001_g5621_i0	Control	Salt_treated	-2.937444951	2.713899097	0.0002967	0.0087083
NODE_56259_length_1535_cov_35.404925_g28362_i0	Control	Salt_treated	-2.938527414	1.959744538	0.0001869	0.0063249
NODE_11587_length_3413_cov_30.869461_g5800_i0	Control	Salt_treated	-2.940823469	3.113855075	2.208E-06	0.0002155
BINPACKER_8571_2	Control	Salt_treated	-2.94426384	2.584887489	0.0005313	0.0128864
NODE_39004_length_1961_cov_11.068326_g19549_i0	Control	Salt_treated	-2.952041002	1.337999229	0.0019005	0.029956
Contig11043	Control	Salt_treated	-2.953253998	5.105659622	1.487E-09	4.187E-07
NODE_39773_length_1937_cov_28.239807_g19934_i0	Control	Salt_treated	-2.964262484	2.472946349	0.0001511	0.0054683
BINPACKER_15934_2	Control	Salt_treated	-2.966971311	0.942665484	0.0039168	0.0484384
BINPACKER_830_5	Control	Salt_treated	-2.97649576	2.726573185	4.987E-06	0.000418
BINPACKER_1967_5	Control	Salt_treated	-2.977660702	2.61832965	0.0005438	0.0131107
NODE_1801_length_6022_cov_30.817112_g945_i0	Control	Salt_treated	-2.983465193	2.019133503	0.0001222	0.0046914
BINPACKER_555_18	Control	Salt_treated	-2.984935008	2.151303251	0.0001581	0.0056109
NODE_57384_length_1512_cov_25.659486_g28943_i0	Control	Salt_treated	-2.994427763	2.567874911	0.0021083	0.032167
BINPACKER_7641_1	Control	Salt_treated	-2.998979911	3.739875927	8.747E-09	2.002E-06
NODE_161583_length_429_cov_49.112360_g107469_i0	Control	Salt_treated	-3.006185544	1.422255978	0.0022572	0.0338586
BINPACKER_1405_14	Control	Salt_treated	-3.007329408	1.723815573	0.0014292	0.0248108
BINPACKER_14353_1	Control	Salt_treated	-3.01447648	1.70912115	0.0004276	0.0110846
BINPACKER_18589_1	Control	Salt_treated	-3.015302077	2.419878917	9.884E-06	0.00071
NODE_52630_length_1613_cov_24.931818_g26444_i0	Control	Salt_treated	-3.024101886	5.361200666	0.0019309	0.0302171
BINPACKER_6171_10	Control	Salt_treated	-3.028369721	1.753848916	0.001002	0.0197243
NODE_3061_length_5189_cov_26.893276_g1569_i0	Control	Salt_treated	-3.028956377	4.47756408	5.444E-11	2.26E-08
NODE_22129_length_2626_cov_26.583627_g11047_i0	Control	Salt_treated	-3.036207054	2.888770368	5.204E-06	0.0004336
Contig9624	Control	Salt_treated	-3.03914358	2.911390319	5.76E-06	0.0004688
Contig107	Control	Salt_treated	-3.051307752	2.901385484	3.361E-07	4.574E-05
NODE_73882_length_1204_cov_27.220159_g4104_i3	Control	Salt_treated	-3.056451564	1.424005726	0.0006884	0.015488
NODE_115388_length_705_cov_68.462025_g15333_i3	Control	Salt_treated	-3.05814736	3.779847197	0.0037649	0.0471859
BINPACKER_4195_2	Control	Salt_treated	-3.058909321	2.796414041	0.0012292	0.0226217
NODE_14303_length_3154_cov_27.895813_g6730_i1	Control	Salt_treated	-3.064453717	1.739052752	0.0005324	0.0128869

BINPACKER_1164_1	Control	Salt_treated	-3.06577596	2.928216313	8.472E-07	9.52E-05
BINPACKER_11694_2	Control	Salt_treated	-3.074437375	1.017909423	0.003843	0.0478328
NODE_11297_length_3442_cov_25.326210_g5656_i0	Control	Salt_treated	-3.079638833	1.42893957	0.001634	0.027154
BINPACKER_3594_3	Control	Salt_treated	-3.08049275	1.022345808	0.0021387	0.0324979
Contig4460	Control	Salt_treated	-3.083299162	1.759667022	0.000269	0.0081843
NODE_116792_length_693_cov_25.180645_g67690_i0	Control	Salt_treated	-3.085076278	1.023455477	0.0024018	0.0353558
Contig3874	Control	Salt_treated	-3.088984533	3.792418164	3.232E-08	6.186E-06
NODE_29914_length_2273_cov_30.569545_g15021_i0	Control	Salt_treated	-3.089069173	1.062222479	0.0036883	0.0467442
BINPACKER_5972_11	Control	Salt_treated	-3.091380237	1.451136268	0.0005706	0.0135956
NODE_99239_length_863_cov_13.473418_g54517_i0	Control	Salt_treated	-3.096320272	2.3199196	0.0003843	0.010292
NODE_58944_length_1479_cov_22.510669_g29760_i0	Control	Salt_treated	-3.098252243	1.065520144	0.0032386	0.0430208
BINPACKER_36691_1	Control	Salt_treated	-3.105225046	1.779942187	0.0001799	0.0061814
NODE_71269_length_1248_cov_23.266383_g36583_i0	Control	Salt_treated	-3.113966417	2.038279878	0.000299	0.008751
Contig9054	Control	Salt_treated	-3.119121739	3.842471396	0.0013211	0.0236141
NODE_47517_length_1735_cov_17.433213_g23815_i0	Control	Salt_treated	-3.119697109	1.825068747	0.0001247	0.0047595
Contig5088	Control	Salt_treated	-3.125676881	4.239546783	4.159E-09	1.039E-06
NODE_8889_length_3728_cov_20.694938_g4491_i0	Control	Salt_treated	-3.128102358	1.829376637	8.986E-05	0.0036701
BINPACKER_9491_2	Control	Salt_treated	-3.134156967	3.953849544	3.299E-05	0.0016836
NODE_3096_length_5171_cov_30.269714_g1045_i1	Control	Salt_treated	-3.134927576	4.125291349	5.213E-11	2.2E-08
NODE_50788_length_1657_cov_25.315657_g20672_i2	Control	Salt_treated	-3.1352642	2.059350642	0.0001181	0.0045774
NODE_2057_length_5796_cov_30.540800_g1080_i0	Control	Salt_treated	-3.142921527	4.633864271	5.098E-07	6.404E-05
NODE_68238_length_1301_cov_27.320033_g34813_i0	Control	Salt_treated	-3.150306825	1.141000209	0.0043921	0.0525574
NODE_42298_length_1868_cov_30.396657_g21142_i0	Control	Salt_treated	-3.154637165	3.888337467	1.342E-10	4.979E-08
BINPACKER_8995_6	Control	Salt_treated	-3.176143885	1.573739064	0.0007372	0.0161069
BINPACKER_29068_4	Control	Salt_treated	-3.176500223	1.919686882	0.0001777	0.0061235
BINPACKER_5680_4	Control	Salt_treated	-3.180643779	1.095479624	0.001682	0.0275776
Contig8947	Control	Salt_treated	-3.18767736	3.839244047	0.0009199	0.0186967
Contig8875	Control	Salt_treated	-3.191677045	1.135518344	0.0026725	0.0379113
NODE_145372_length_501_cov_52.018692_g92433_i0	Control	Salt_treated	-3.194868377	1.136595	0.0023479	0.0347502
NODE_38061_length_1989_cov_22.081420_g19083_i0	Control	Salt_treated	-3.198784384	1.528315127	0.0011239	0.0212759
BINPACKER_2061_8	Control	Salt_treated	-3.20847238	4.040303917	0.0019099	0.0300644
NODE_64909_length_1361_cov_30.470497_g4442_i4	Control	Salt_treated	-3.214315551	1.86634548	8.858E-05	0.0036466
BINPACKER_14318_1	Control	Salt_treated	-3.22337972	2.378496084	5.929E-06	0.0004778
NODE_61979_length_1417_cov_12.124256_g31404_i0	Control	Salt_treated	-3.231777703	1.556179579	0.0005591	0.0133871
BINPACKER_2512_4	Control	Salt_treated	-3.233244288	2.697708041	1.569E-05	0.0010019
NODE_13015_length_3269_cov_27.933667_g6524_i0	Control	Salt_treated	-3.245339149	3.831927187	0.0003718	0.0100956
NODE_44374_length_1812_cov_26.252444_g7991_i2	Control	Salt_treated	-3.248881465	1.564669014	0.0003963	0.0105399
BINPACKER_8441_9	Control	Salt_treated	-3.25756472	3.640388151	0.000143	0.0052755
NODE_53773_length_1588_cov_25.504950_g24584_i1	Control	Salt_treated	-3.267317863	3.698638388	0.0003899	0.0104137
NODE_85011_length_1038_cov_24.607254_g20507_i3	Control	Salt_treated	-3.272560742	2.490154081	3.981E-05	0.001927
NODE_12679_length_3304_cov_20.062210_g6352_i0	Control	Salt_treated	-3.278506123	2.234718922	1.98E-05	0.0011771
NODE_92923_length_937_cov_19.097222_g43749_i2	Control	Salt_treated	-3.312065897	2.396128827	0.0001733	0.005997
BINPACKER_3476_10	Control	Salt_treated	-3.312651266	1.595678295	0.0014912	0.0255079
NODE_79165_length_1121_cov_29.895038_g40019_i7	Control	Salt_treated	-3.332114579	1.203913459	0.0006718	0.0152657
NODE_1794_length_6029_cov_30.733546_g780_i1	Control	Salt_treated	-3.335668645	2.440011968	2.433E-05	0.0013455
NODE_19074_length_2808_cov_26.791225_g9507_i0	Control	Salt_treated	-3.337301051	1.240168561	0.0009752	0.0194068
NODE_6692_length_4092_cov_31.196317_g3064_i1	Control	Salt_treated	-3.341029956	1.670199526	0.000215	0.0070297
BINPACKER_6344_6	Control	Salt_treated	-3.343422081	2.095533094	0.000271	0.0082161
NODE_71726_length_1240_cov_26.986290_g7216_i3	Control	Salt_treated	-3.359711129	2.679386487	1.642E-06	0.0001697
NODE_14152_length_3166_cov_31.508244_g7089_i0	Control	Salt_treated	-3.366978377	1.268204957	0.0022641	0.0339205
BINPACKER_7867_2	Control	Salt_treated	-3.369518777	1.723548166	0.0002965	0.0087083
NODE_4706_length_4596_cov_23.638072_g2395_i0	Control	Salt_treated	-3.371266461	2.91132881	4.346E-08	8.064E-06
NODE_20942_length_2692_cov_27.568156_g9710_i1	Control	Salt_treated	-3.372788727	1.664403273	0.0001171	0.00456
NODE_35274_length_2080_cov_32.264076_g14785_i1	Control	Salt_treated	-3.408790347	2.987175508	0.0019698	0.0306395
NODE_37488_length_2007_cov_29.026887_g15922_i2	Control	Salt_treated	-3.412775947	1.262883215	0.001656	0.0273342
NODE_81603_length_1085_cov_24.329051_g19383_i1	Control	Salt_treated	-3.412930363	1.265982913	0.0005234	0.012744
NODE_97620_length_881_cov_44.339109_g53389_i0	Control	Salt_treated	-3.426569781	3.316044865	2.38E-05	0.0013271
BINPACKER_4945_5	Control	Salt_treated	-3.429286492	5.537380434	6.643E-09	1.599E-06
BINPACKER_8092_5	Control	Salt_treated	-3.440382316	2.516097616	1.64E-05	0.0010406
BINPACKER_29842_1	Control	Salt_treated	-3.444159918	4.631307279	2.851E-10	9.438E-08
NODE_17430_length_2912_cov_32.158507_g8648_i0	Control	Salt_treated	-3.454907439	1.295404182	0.0010461	0.0202088
NODE_29945_length_2272_cov_28.192815_g15035_i0	Control	Salt_treated	-3.455154135	1.297450927	0.0005666	0.0135129
NODE_19174_length_2801_cov_22.208944_g9557_i0	Control	Salt_treated	-3.463663406	1.75125189	0.0035906	0.0457787
NODE_2939_length_5259_cov_21.655226_g1501_i0	Control	Salt_treated	-3.478548235	1.751123094	0.0001024	0.0040567
BINPACKER_20015_1	Control	Salt_treated	-3.489521814	0.668291511	0.0037817	0.0473101
NODE_20823_length_2698_cov_32.540952_g8501_i1	Control	Salt_treated	-3.497830496	1.330228014	0.0003075	0.0088795
BINPACKER_21195_1	Control	Salt_treated	-3.499359982	2.123149546	1.34E-05	0.0008874
NODE_5029_length_4498_cov_28.343955_g2563_i0	Control	Salt_treated	-3.509014081	3.621225648	3.368E-11	1.557E-08
BINPACKER_15812_10	Control	Salt_treated	-3.509845196	1.768841402	0.0004565	0.0114931
NODE_18389_length_2850_cov_24.696075_g9153_i0	Control	Salt_treated	-3.518176924	1.33631424	0.0004424	0.011288
NODE_174407_length_384_cov_29.173633_g119523_i0	Control	Salt_treated	-3.530751651	2.426324931	0.0009746	0.0194068
NODE_39113_length_1957_cov_27.431529_g17930_i2	Control	Salt_treated	-3.533660605	2.439279767	2.853E-06	0.0002627
NODE_111697_length_739_cov_30.312312_g63633_i0	Control	Salt_treated	-3.560618977	0.716804429	0.002702	0.0381775
BINPACKER_22054_1	Control	Salt_treated	-3.560829995	1.873338698	6.624E-05	0.0028766
BINPACKER_4812_1	Control	Salt_treated	-3.561492815	1.806234235	9.449E-05	0.0037943
NODE_2130_length_5751_cov_29.685981_g1105_i0	Control	Salt_treated	-3.566003073	4.618698732	8.299E-16	8.838E-13
NODE_125836_length_621_cov_23.624088_g75322_i0	Control	Salt_treated	-3.569139334	0.719199104	0.0030277	0.0411972
NODE_10114_length_3563_cov_30.214900_g5109_i0	Control	Salt_treated	-3.574632592	1.824951829	2.867E-05	0.0015235
BINPACKER_9620_2	Control	Salt_treated	-3.575499301	0.720396982	0.0038425	0.0478328
NODE_116469_length_696_cov_28.492777_g67443_i0	Control	Salt_treated	-3.575499301	0.720396982	0.0038425	0.0478328
BINPACKER_6890_3	Control	Salt_treated	-3.57607839	1.389658944	0.0002159	0.0070412
NODE_138991_length_536_cov_51.438445_g86718_i0	Control	Salt_treated	-3.580830179	1.865305208	2.379E-05	0.0013271
NODE_28705_length_2317_cov_32.089127_g14407_i0	Control	Salt_treated	-3.587573932	2.686618165	0.0003805	0.0102301
NODE_62676_length_1403_cov_26.886466_g24634_i2	Control	Salt_treated	-3.606234265	1.847648532	3.91E-05	0.0019076
NODE_6291_length_4175_cov_27.213067_g3186_i0	Control	Salt_treated	-3.622095066	3.34890265	2.745E-09	7.231E-07
NODE_89248_length_983_cov_9.305495_g47753_i0	Control	Salt_treated	-3.624614931	0.761273876	0.0044651	0.0532157
NODE_103458_length_820_cov_53.353414_g57407_i0	Control	Salt_treated	-3.635626567	0.766007026	0.0019701	0.0306395
BINPACKER_22565_2	Control	Salt_treated	-3.641002849	0.76719116	0.0023873	0.0352272
BINPACKER_8269_4	Control	Salt_treated	-3.645748663	3.321538813	2.276E-06	0.0002203
BINPACKER_16543_5	Control	Salt_treated	-3.647692616	0.768375633	0.0031793	0.0424632
NODE_59065_length_1476_cov_29.938703_g29827_i0	Control	Salt_treated	-3.668345841	1.967087641	7.207E-05	0.0030916
NODE_635_length_7772_cov_30.879465_g344_i0	Control	Salt_treated	-3.674775236	3.355204447	4.401E-07	5.734E-05
NODE_90320_length_969_cov_25.392857_g48467_i0	Control	Salt_treated	-3.685695257	1.539640047	0.0002612	0.0080492
NODE_15299_length_3078_cov_21.868552_g7166_i1	Control	Salt_treated	-3.690079405	0.80656968	0.0037175	0.0468175
Contig11882	Control	Salt_treated	-3.694988673	4.58672441	2.128E-17	2.896E-14
NODE_54112_length_1580_cov_27.221632_g16257_i6	Control	Salt_treated	-3.70976003	0.813591254	0.0019485	0.030401
NODE_94055_length_923_cov_30.422353_g50970_i0	Control	Salt_treated	-3.711438644	3.304477203	0.0035635	0.045676
BINPACKER_1525_4	Control	Salt_treated	-3.722345642	1.933384311	5.282E-05	0.0024049
BINPACKER_20082_3	Control	Salt_treated	-3.722929671	3.097990569	1.665E-07	2.534E-05
NODE_16027_length_3022_cov_30.863344_g7970_i0	Control	Salt_treated	-3.741956853	2.901787041	0.0016116	0.0269272
NODE_27625_length_2361_cov_24.315122_g13853_i0	Control	Salt_treated	-3.755765358	0.852712622	0.001664	0.0273737
NODE_23202_length_2568_cov_28.131463_g11588_i0	Control	Salt_treated	-3.758937891	1.531480611	5.461E-05	0.0024771
NODE_60014_length_1455_cov_30.783647_g30320_i0	Control	Salt_treated	-3.763909947	1.983519832	1.465E-05	0.0009446
BINPACKER_833_3	Control	Salt_treated	-3.788056582	2.338576249	1.601E-06	0.0001662
NODE_53686_length_1590_cov_17.008570_g18273_i2	Control	Salt_treated	-3.791623729	2.063091701	1.464E-05	0.0009446
NODE_67749_length_1310_cov_33.109135_g34532_i0	Control	Salt_treated	-3.808707206	3.873049971	0.0003961	0.0105399
BINPACKER_5491_12	Control	Salt_treated	-3.815494334	0.895142207	0.0013509	0.0239443
NODE_71642_length_1241_cov_32.800514_g36817_i0	Control	Salt_treated	-3.815991498	2.057119526	8.467E-06	0.0006323
BINPACKER_11104_5	Control	Salt_treated	-3.816623791	2.849109571	4.269E-07	5.592E-05

NODE_3386_length_5048_cov_33.671156_g1737_i0	Control	Salt_treated	-3.824038766	2.682908957	3.503E-07	4.715E-05
NODE_1550_length_6254_cov_21.148843_g837_i0	Control	Salt_treated	-3.831040165	2.86569421	6.407E-08	1.105E-05
BINPACKER_27267_2	Control	Salt_treated	-3.842318691	0.945347366	0.0027914	0.0391164
NODE_138847_length_537_cov_18.465517_g86584_i0	Control	Salt_treated	-3.843819748	0.946481996	0.0033026	0.0435461
BINPACKER_12996_3	Control	Salt_treated	-3.844369597	2.8706806	2.115E-07	3.084E-05
NODE_19991_length_2752_cov_27.438223_g9945_i0	Control	Salt_treated	-3.881322144	3.69738358	1.084E-10	4.213E-08
NODE_4327_length_4705_cov_30.112478_g353_i2	Control	Salt_treated	-3.885395296	1.631482483	3.962E-05	0.0019216
NODE_40614_length_1913_cov_27.597283_g1959_i2	Control	Salt_treated	-3.887191036	1.633320504	2.529E-05	0.0013826
Contig950	Control	Salt_treated	-3.889995991	0.982948715	0.0021982	0.0330951
NODE_13858_length_3189_cov_24.645379_g1896_i1	Control	Salt_treated	-3.945709934	2.233201376	9.069E-05	0.0036964
BINPACKER_5175_2	Control	Salt_treated	-3.955611185	1.685579517	1.448E-05	0.0009396
NODE_30823_length_2238_cov_32.937182_g15453_i0	Control	Salt_treated	-3.982358818	2.599469884	0.0002302	0.0073908
BINPACKER_24385_2	Control	Salt_treated	-3.996736167	1.021633915	0.0004399	0.011259
NODE_26369_length_2414_cov_29.865869_g5216_i1	Control	Salt_treated	-3.996810496	2.174317474	2.66E-06	0.0002503
NODE_2281_length_5671_cov_33.391568_g780_i3	Control	Salt_treated	-4.004456519	3.57418316	2.615E-12	1.392E-09
NODE_66300_length_1336_cov_26.726049_g33760_i0	Control	Salt_treated	-4.018636266	3.818647687	0.0007586	0.0164304
NODE_111785_length_738_cov_27.527820_g63709_i0	Control	Salt_treated	-4.050623076	1.060357921	0.0003652	0.0099836
NODE_8877_length_3729_cov_32.091904_g4483_i0	Control	Salt_treated	-4.09426814	2.872889747	7.53E-09	1.773E-06
NODE_20777_length_2701_cov_26.247336_g10351_i0	Control	Salt_treated	-4.106935222	1.803031314	9.433E-06	0.0006877
BINPACKER_9236_10	Control	Salt_treated	-4.133840201	1.128275464	0.0002388	0.0075686
BINPACKER_3259_13	Control	Salt_treated	-4.140817317	1.131500416	0.0001743	0.0060127
Contig11185	Control	Salt_treated	-4.175767768	4.138488423	7.039E-17	8.62E-14
BINPACKER_25672_1	Control	Salt_treated	-4.189174763	1.167282668	0.0001347	0.005023
BINPACKER_4016_2	Control	Salt_treated	-4.220945908	1.193743654	0.0006784	0.0153293
NODE_73394_length_1212_cov_40.580334_g37844_i0	Control	Salt_treated	-4.227487912	1.906421818	1.668E-06	0.000171
BINPACKER_2145_12	Control	Salt_treated	-4.230176954	1.200063576	9.193E-05	0.0037221
NODE_49562_length_1686_cov_30.755115_g22094_i1	Control	Salt_treated	-4.230581399	2.33833697	4.24E-05	0.0020327
BINPACKER_7391_11	Control	Salt_treated	-4.236682928	3.286345764	1.382E-09	3.937E-07
NODE_14542_length_3134_cov_23.969944_g3441_i1	Control	Salt_treated	-4.303556012	5.266523606	2.45E-05	0.0013518
BINPACKER_337_5	Control	Salt_treated	-4.309478248	1.97484707	7.57E-06	0.0005795
NODE_96083_length_899_cov_28.601695_g52331_i0	Control	Salt_treated	-4.311176631	1.97889926	1.11E-06	0.0001208
BINPACKER_2663_4	Control	Salt_treated	-4.319426605	1.267371916	5.16E-05	0.0023581
NODE_5498_length_4366_cov_32.645935_g2799_i0	Control	Salt_treated	-4.320551758	2.057807515	2.939E-05	0.0015449
NODE_1442_length_6375_cov_26.719930_g780_i0	Control	Salt_treated	-4.355497177	2.01523353	1.48E-06	0.000157
BINPACKER_4614_6	Control	Salt_treated	-4.365161308	2.828772012	1.56E-08	3.323E-06
NODE_6259_length_4181_cov_31.654820_g2241_i2	Control	Salt_treated	-4.436714667	1.359606966	2.833E-05	0.0015151
Contig8	Control	Salt_treated	-4.464454736	2.573182468	2.46E-08	4.898E-06
NODE_20716_length_2705_cov_16.951368_g10315_i0	Control	Salt_treated	-4.472120973	1.388003747	2.674E-05	0.0014459
NODE_50738_length_1658_cov_33.369716_g25454_i0	Control	Salt_treated	-4.484301657	1.431800956	0.0006886	0.015488
BINPACKER_5740_6	Control	Salt_treated	-4.514675171	2.952747521	3.292E-08	6.252E-06
BINPACKER_7938_2	Control	Salt_treated	-4.541472315	1.442993482	4.819E-05	0.0022574
NODE_17825_length_2884_cov_22.620064_g8859_i0	Control	Salt_treated	-4.564253348	3.311465995	3.283E-12	1.711E-09
BINPACKER_1205_5	Control	Salt_treated	-4.682800128	2.817098083	8.766E-08	1.45E-05
NODE_209_length_10332_cov_31.315820_g108_i0	Control	Salt_treated	-4.755720861	3.889085043	4.414E-14	3.488E-11
NODE_113847_length_719_cov_32.125387_g65339_i0	Control	Salt_treated	-4.765162128	3.237406791	2.419E-05	0.0013403
NODE_16780_length_2964_cov_30.533034_g8325_i0	Control	Salt_treated	-4.78420942	1.671692915	0.0001972	0.0066273
NODE_31098_length_2228_cov_28.062181_g15590_i0	Control	Salt_treated	-5.128174433	3.760673492	0.0016919	0.0275924
NODE_136518_length_551_cov_19.205021_g84500_i0	Control	Salt_treated	-5.360943539	2.076791542	0.0002908	0.0086014
NODE_74807_length_1189_cov_29.235663_g38693_i0	Control	Salt_treated	-5.593988935	0.32663782	0.0037095	0.0468132
NODE_94741_length_915_cov_25.200713_g44353_i1	Control	Salt_treated	-5.598809416	0.327938706	0.003581	0.0457091
NODE_95625_length_905_cov_15.747596_g52007_i0	Control	Salt_treated	-5.598809416	0.327938706	0.003581	0.0457091
Contig5509	Control	Salt_treated	-5.603838622	0.329240149	0.0040732	0.0494664
NODE_61582_length_1425_cov_30.119822_g28445_i1	Control	Salt_treated	-5.603838622	0.329240149	0.0040732	0.0494664
Contig5894	Control	Salt_treated	-5.683954731	0.387197877	0.0034602	0.0448212
NODE_161270_length_431_cov_2.826816_g107176_i0	Control	Salt_treated	-5.683954731	0.387197877	0.0034602	0.0448212
NODE_51284_length_1645_cov_21.924936_g25736_i0	Control	Salt_treated	-5.689369521	0.388481841	0.0026351	0.0376151
BINPACKER_4209_2	Control	Salt_treated	-5.689369521	0.388481841	0.0026351	0.0376151
NODE_46665_length_1754_cov_29.191553_g23407_i0	Control	Salt_treated	-5.689369521	0.388481841	0.0026351	0.0376151
NODE_59149_length_1474_cov_36.484654_g29874_i0	Control	Salt_treated	-5.694107724	0.389766329	0.0023399	0.0346828
NODE_62510_length_1406_cov_30.139535_g31679_i0	Control	Salt_treated	-5.703813987	0.392336877	0.0030564	0.0413057
BINPACKER_15393_1	Control	Salt_treated	-5.703813987	0.392336877	0.0030564	0.0413057
NODE_42111_length_1873_cov_27.469444_g21045_i0	Control	Salt_treated	-5.724573278	2.433505245	1.306E-09	3.839E-07
NODE_74415_length_1195_cov_35.800357_g38444_i0	Control	Salt_treated	-5.778638144	0.447641029	0.0028292	0.0394207
NODE_46505_length_1758_cov_28.263501_g23314_i0	Control	Salt_treated	-5.787788066	0.450178617	0.0015605	0.0263974
NODE_21685_length_2652_cov_6.679721_g7033_i1	Control	Salt_treated	-5.787788066	0.450178617	0.0015605	0.0263974
BINPACKER_8269_5	Control	Salt_treated	-5.845023878	2.536947344	3.655E-07	4.866E-05
NODE_116468_length_696_cov_28.495987_g67442_i0	Control	Salt_treated	-5.862526962	0.504336834	0.0021612	0.0326878
NODE_43695_length_1831_cov_22.903868_g21872_i0	Control	Salt_treated	-5.867337734	0.505589879	0.0016752	0.027539
NODE_94720_length_915_cov_33.425178_g51414_i0	Control	Salt_treated	-5.885734806	0.510606691	0.0027194	0.0383695
BINPACKER_40947_1	Control	Salt_treated	-5.950822812	0.561240892	0.0010286	0.0199801
BINPACKER_18216_5	Control	Salt_treated	-5.950822812	0.561240892	0.0010286	0.0199801
BINPACKER_1588_13	Control	Salt_treated	-5.954857197	0.562479725	0.0010065	0.0197699
NODE_2315_length_5642_cov_32.165918_g1171_i0	Control	Salt_treated	-5.954857197	0.562479725	0.0010065	0.0197699
BINPACKER_15667_1	Control	Salt_treated	-6.025779097	0.613544164	0.0007378	0.0161069
NODE_120170_length_665_cov_24.891892_g40117_i2	Control	Salt_treated	-6.037832317	0.617217684	0.0008881	0.0182193
Contig11434	Control	Salt_treated	-6.037832317	0.617217684	0.0008881	0.0182193
NODE_137621_length_544_cov_28.787686_g85497_i0	Control	Salt_treated	-6.047761417	0.619668741	0.0018737	0.0296866
NODE_109771_length_757_cov_35.872807_g62083_i2	Control	Salt_treated	-6.087695866	0.661488921	0.0011496	0.0216005
NODE_109770_length_757_cov_35.976608_g62083_i1	Control	Salt_treated	-6.087695866	0.661488921	0.0011496	0.0216005
BINPACKER_4325_13	Control	Salt_treated	-6.096919611	0.663907261	0.0005537	0.0132831
BINPACKER_8376_4	Control	Salt_treated	-6.096919611	0.663907261	0.0005537	0.0132831
NODE_4345_length_4700_cov_30.559542_g2201_i0	Control	Salt_treated	-6.104574813	0.666327144	0.0004546	0.0114672
BINPACKER_43030_1	Control	Salt_treated	-6.104574813	0.666327144	0.0004546	0.0114672
Contig2791	Control	Salt_treated	-6.160233333	0.711272532	0.0006265	0.0145259
BINPACKER_11862_1	Control	Salt_treated	-6.172187837	0.714859846	0.0003043	0.0088584
BINPACKER_2629_6	Control	Salt_treated	-6.175814383	0.716056342	0.0003281	0.0092909
BINPACKER_5721_10	Control	Salt_treated	-6.188655785	0.719647995	0.0008894	0.0182314
NODE_23146_length_2571_cov_26.692954_g11564_i0	Control	Salt_treated	-6.22478287	0.758168065	0.0007051	0.0156722
NODE_45586_length_1780_cov_31.975981_g22858_i0	Control	Salt_treated	-6.23311245	0.760532024	0.0003563	0.0098172
BINPACKER_47208_1	Control	Salt_treated	-6.243801948	0.764080517	0.0002493	0.0078177
BINPACKER_4357_1	Control	Salt_treated	-6.24770185	0.765264027	0.0003361	0.0094509
BINPACKER_21501_1	Control	Salt_treated	-6.24770185	0.765264027	0.0003361	0.0094509
BINPACKER_7186_1	Control	Salt_treated	-6.251982633	0.766447876	0.0004967	0.0122646
NODE_1318_length_6520_cov_29.671785_g682_i1	Control	Salt_treated	-6.281783913	0.802327719	0.0009586	0.0191994
NODE_39372_length_1950_cov_23.713905_g1408_i4	Control	Salt_treated	-6.297597005	3.74278536	1.217E-19	2.128E-16
BINPACKER_14485_2	Control	Salt_treated	-6.301846613	0.808172069	0.0002018	0.0067361
NODE_17436_length_2912_cov_20.072561_g8651_i0	Control	Salt_treated	-6.308834191	0.810512048	0.0002729	0.0082516
NODE_9889_length_3590_cov_26.065397_g5001_i0	Control	Salt_treated	-6.312755256	0.811682515	0.0002823	0.0084437
BINPACKER_14629_1	Control	Salt_treated	-6.331474691	0.816367554	0.0018358	0.0292981
NODE_62253_length_1411_cov_24.207025_g31547_i0	Control	Salt_treated	-6.340998478	0.846201637	0.0008452	0.0176502
Contig12431	Control	Salt_treated	-6.414265532	0.893272347	0.0001319	0.0049474
NODE_22046_length_2631_cov_29.685301_g11001_i0	Control	Salt_treated	-6.424001742	0.896704737	0.0001053	0.0041485
BINPACKER_5195_7	Control	Salt_treated	-6.452525064	0.929905336	0.0008383	0.0175959
NODE_43576_length_1834_cov_31.222033_g21802_i0	Control	Salt_treated	-6.461003583	3.115306432	2.668E-13	1.8E-10
NODE_12888_length_3283_cov_32.258567_g6454_i0	Control	Salt_treated	-6.461169073	0.932166441	0.0002497	0.0078182
BINPACKER_14585_1	Control	Salt_treated	-6.492402228	0.942354516	0.0002486	0.0078082
BINPACKER_11299_2	Control	Salt_treated	-6.500825991	0.944621419	0.0006406	0.0147471
BINPACKER_8774_5	Control	Salt_treated	-6.505186001	0.969894215	0.0007533	0.0163813
NODE_32434_length_2181_cov_26.444972_g10712_i1	Control	Salt_treated	-6.509830755	0.946889363	0.0016594	0.027371

BINPACKER_43526_1	Control	Salt_treated	-6.59365743	1.020922207	8.103E-05	0.0033815
BINPACKER_1516_3	Control	Salt_treated	-6.597253146	1.022031443	0.0001254	0.0047753
BINPACKER_15521_1	Control	Salt_treated	-6.613293578	1.026470669	0.000859	0.0178471
BINPACKER_5245_4	Control	Salt_treated	-6.667722177	1.08756451	7.53E-05	0.0031968
NODE_58430_length_1490_cov_34.649965_g28317_i2	Control	Salt_treated	-6.674200387	1.089734409	3.448E-05	0.0017448
BINPACKER_13486_9	Control	Salt_treated	-6.682697723	1.092990781	2.384E-05	0.0013271
NODE_15097_length_3094_cov_31.203906_g7507_i1	Control	Salt_treated	-6.719806776	1.125430717	2.873E-05	0.0015235
Contig22	Control	Salt_treated	-6.733734992	1.130803744	2.275E-05	0.0012989
BINPACKER_2302_3	Control	Salt_treated	-6.750532965	1.155955354	0.0001944	0.0065573
NODE_12052_length_3365_cov_30.170109_g6037_i0	Control	Salt_treated	-6.761535596	1.139410319	0.0007584	0.0164304
BINPACKER_14815_5	Control	Salt_treated	-6.766904717	1.161270797	1.73E-05	0.0010727
NODE_113199_length_725_cov_34.136503_g64816_i0	Control	Salt_treated	-6.777636322	1.165526344	1.445E-05	0.0009396
BINPACKER_13772_2	Control	Salt_treated	-6.81247677	1.196216352	1.082E-05	0.0007616
Contig10215	Control	Salt_treated	-6.81247677	1.196216352	1.082E-05	0.0007616
BINPACKER_3916_2	Control	Salt_treated	-6.831774843	1.203592397	3.716E-05	0.0018427
BINPACKER_28172_4	Control	Salt_treated	-6.872355194	1.254303196	0.0002413	0.0076159
NODE_80828_length_1096_cov_23.658847_g32405_i1	Control	Salt_treated	-6.91236338	1.268760999	1.279E-05	0.0008562
BINPACKER_11327_4	Control	Salt_treated	-6.941063932	1.296104488	4.534E-06	0.0003856
Contig7977	Control	Salt_treated	-6.983874613	1.32888999	3.682E-06	0.0003198
BINPACKER_4_4	Control	Salt_treated	-7.020872532	1.358942459	2.571E-06	0.0002441
Contig4402	Control	Salt_treated	-7.033489324	1.363964814	1.216E-05	0.0008181
NODE_57846_length_1502_cov_23.522043_g22056_i1	Control	Salt_treated	-7.039535452	1.381377102	1.752E-05	0.0010812
BINPACKER_7553_1	Control	Salt_treated	-7.052394788	1.38634904	2.184E-06	0.0002144
Contig3190	Control	Salt_treated	-7.071747109	1.394309516	1.121E-05	0.0007754
BINPACKER_2838_12	Control	Salt_treated	-7.074864217	1.410208707	1.604E-05	0.0010207
Contig214	Control	Salt_treated	-7.077614183	1.41119379	1.016E-05	0.0007255
NODE_86346_length_1020_cov_31.897571_g22918_i1	Control	Salt_treated	-7.145393348	1.453088776	9.714E-06	0.0006999
BINPACKER_3965_6	Control	Salt_treated	-7.159973471	1.472860565	1.099E-06	0.0001202
BINPACKER_11212_2	Control	Salt_treated	-7.181093014	1.495099162	5E-06	0.000418
BINPACKER_11299_3	Control	Salt_treated	-7.190550932	1.498935661	1.094E-06	0.0001201
BINPACKER_8072_7	Control	Salt_treated	-7.202623974	1.51784178	3.687E-05	0.0018395
NODE_71321_length_1247_cov_24.484668_g33464_i1	Control	Salt_treated	-7.205653339	1.505652315	1.901E-06	0.0001917
NODE_42937_length_1851_cov_23.165354_g21450_i0	Control	Salt_treated	-7.21564802	1.522594095	2.874E-06	0.0002637
BINPACKER_11666_3	Control	Salt_treated	-7.237316731	1.532103702	1.165E-06	0.0001262
BINPACKER_15128_2	Control	Salt_treated	-7.2601115	1.554273063	4.163E-07	5.483E-05
NODE_113_length_11949_cov_23.605760_g53_i0	Control	Salt_treated	-7.266203629	1.557101513	5.514E-07	6.719E-05
BINPACKER_13620_1	Control	Salt_treated	-7.332559737	1.610324166	5.543E-07	6.722E-05
NODE_16394_length_2994_cov_32.117083_g8141_i0	Control	Salt_treated	-7.359563544	1.633910404	3.206E-07	4.437E-05
BINPACKER_495_1	Control	Salt_treated	-7.38055458	1.654339707	1.74E-07	2.614E-05
BINPACKER_3639_12	Control	Salt_treated	-7.387116436	1.644940668	4.704E-05	0.0022159
BINPACKER_13876_1	Control	Salt_treated	-7.487971445	1.745779777	1.416E-07	2.195E-05
Contig8351	Control	Salt_treated	-7.526389468	1.77403376	5.695E-08	1.026E-05
NODE_105282_length_801_cov_29.458791_g11442_i4	Control	Salt_treated	-7.564761437	1.801833478	3.609E-07	4.83E-05
Contig3734	Control	Salt_treated	-7.571821252	1.814555953	3.293E-07	4.507E-05
BINPACKER_3980_1	Control	Salt_treated	-7.573504781	1.815416898	2.957E-07	4.115E-05
NODE_42009_length_1876_cov_30.190238_g7261_i1	Control	Salt_treated	-7.581658936	1.828787393	7.03E-07	8.161E-05
BINPACKER_208_14	Control	Salt_treated	-7.615398391	1.864170152	2.106E-05	0.0012342
BINPACKER_7879_6	Control	Salt_treated	-7.620690433	1.856362411	2.864E-07	4.009E-05
NODE_100983_length_845_cov_30.231865_g49217_i2	Control	Salt_treated	-7.638088887	1.854414334	3.343E-05	0.0017024
BINPACKER_8504_1	Control	Salt_treated	-7.678509483	1.910935248	4.108E-07	5.439E-05
BINPACKER_15122_6	Control	Salt_treated	-7.685431166	1.923935541	2.009E-05	0.0011858
BINPACKER_4692_2	Control	Salt_treated	-7.741998667	1.967225743	1.056E-06	0.0001171
BINPACKER_370_1	Control	Salt_treated	-7.762589077	1.976951442	7.416E-08	1.253E-05
BINPACKER_5619_12	Control	Salt_treated	-7.787036242	2.005321299	5.995E-07	7.198E-05
BINPACKER_15611_7	Control	Salt_treated	-7.805103327	1.997202527	1.765E-05	0.0010861
BINPACKER_4611_8	Control	Salt_treated	-7.821785076	2.029930814	1.248E-07	1.986E-05
BINPACKER_7774_3	Control	Salt_treated	-7.886171611	2.078153674	9.484E-08	1.539E-05
BINPACKER_7486_1	Control	Salt_treated	-7.897958604	2.0981281	4.419E-08	8.139E-06
BINPACKER_18532_2	Control	Salt_treated	-7.936837241	2.13208131	4.735E-08	8.655E-06
NODE_98684_length_869_cov_25.770101_g54129_i0	Control	Salt_treated	-7.962380112	2.145770037	1.488E-08	3.197E-06
BINPACKER_2723_1	Control	Salt_treated	-8.011658854	2.191155932	3.525E-09	8.994E-07
BINPACKER_14225_1	Control	Salt_treated	-8.079540038	2.246646854	9.337E-09	2.098E-06
BINPACKER_3574_6	Control	Salt_treated	-8.254180712	2.403715932	2.487E-10	8.347E-08
NODE_41225_length_1897_cov_30.594298_g12887_i1	Control	Salt_treated	-8.290221798	2.434358773	1.549E-10	5.662E-08
NODE_74820_length_1189_cov_20.601254_g19063_i3	Control	Salt_treated	-8.306311424	2.448442562	1.316E-10	4.96E-08
BINPACKER_9064_5	Control	Salt_treated	-8.339665303	2.481313391	4.569E-10	1.492E-07
NODE_39385_length_1949_cov_33.895522_g6691_i2	Control	Salt_treated	-8.419986205	2.550556969	2.862E-11	1.371E-08
BINPACKER_8843_3	Control	Salt_treated	-8.448962243	2.562512419	1.405E-06	0.0001509
NODE_55018_length_1561_cov_27.586022_g27740_i0	Control	Salt_treated	-8.455697465	2.584031655	8.154E-11	3.274E-08
NODE_12371_length_3334_cov_31.317694_g5600_i1	Control	Salt_treated	-8.476025653	2.600418216	1.069E-11	5.342E-09
Contig3654	Control	Salt_treated	-8.639163782	2.74475057	1.669E-12	1E-09
NODE_9547_length_3636_cov_27.134718_g4676_i2	Control	Salt_treated	-8.701698598	2.793344272	6.865E-09	1.633E-06
BINPACKER_17821_3	Control	Salt_treated	-8.803719705	2.889893562	4.864E-11	2.127E-08
BINPACKER_1743_1	Control	Salt_treated	-8.93202128	3.008870939	7.324E-14	5.607E-11
BINPACKER_1516_15	Control	Salt_treated	-8.948214252	3.024466318	1.051E-14	9.406E-12
BINPACKER_11298_4	Control	Salt_treated	-8.971562763	3.044952645	3.641E-14	3.075E-11
BINPACKER_1736_23	Control	Salt_treated	-9.084111238	3.149657103	2.196E-16	2.445E-13
BINPACKER_3203_11	Control	Salt_treated	-9.233303422	3.290882606	1.324E-12	7.91E-10
NODE_27809_length_2352_cov_31.843352_g13955_i0	Control	Salt_treated	-9.358777381	3.388447771	4.077E-05	0.001966
Contig4882	Control	Salt_treated	-9.694696498	3.714011183	1.204E-21	2.95E-18
BINPACKER_6268_11	Control	Salt_treated	-9.717948217	3.743328621	1.656E-10	5.964E-08
NODE_35179_length_2083_cov_30.670149_g17626_i0	Control	Salt_treated	-9.8948048	6.465089765	5.845E-06	0.0004725
NODE_43689_length_1831_cov_28.917520_g4080_i2	Control	Salt_treated	-10.05750502	4.055825262	1.481E-23	4.533E-20
BINPACKER_9470_2	Control	Salt_treated	-10.08467747	4.079983934	2.004E-24	8.181E-21
BINPACKER_2578_1	Control	Salt_treated	-10.62893175	4.598120172	6.603E-31	8.087E-27
BINPACKER_627_2	Control	Salt_treated	-10.7231777	4.687589375	1.018E-28	8.312E-25
NODE_27886_length_2349_cov_50.690685_g13995_i0	Control	Salt_treated	-11.28257266	5.222607011	1.391E-05	0.0009156

TableS1b.Matrix for DEGs (after edgeR) w

	Control_leaf1	Control_leaf2	Salt_treated_leaf1	Salt_treated_leaf2
NODE_43576_length_1834_cov_31.222033_g21802_i0	0	1	91	64
NODE_42937_length_1851_cov_23.165354_g21450_i0	0	0	29	15
NODE_30996_length_2232_cov_32.053265_g15531_i0	35	7	231	126
NODE_131582_length_581_cov_40.011811_g80282_i0	14	7	89	94
NODE_19622_length_2772_cov_22.017414_g1560_i5	16	5	106	67
BINPACKER_3980_1	0	0	30	26
NODE_5798_length_4289_cov_26.403463_g2737_i2	72	27	472	453
Contig9002	17	8	78	81
NODE_6290_length_4175_cov_30.597513_g3185_i0	32	8	154	124
BINPACKER_9064_5	0	0	59	37
BINPACKER_182_10	15	8	102	64
BINPACKER_1274_1	278	392	76	93
BINPACKER_4614_6	4	0	50	70
NODE_65062_length_1358_cov_111.919066_g27862_i51	96	52	0	0
NODE_96083_length_899_cov_28.601695_g52331_i0	2	0	37	25
Contig3654	0	0	55	62
NODE_69162_length_1285_cov_22.133663_g35349_i0	10	8	73	119
Contig1648	40343	21454	9607	6531
BINPACKER_8504_1	0	0	43	18
BINPACKER_11298_4	0	0	64	83
NODE_79022_length_1123_cov_30.629524_g41258_i0	78	45	21	15
BINPACKER_11327_4	0	0	19	17
NODE_15365_length_3072_cov_30.894632_g7657_i0	1905	587	360	300
BINPACKER_11298_1	57	20	0	0
BINPACKER_9778_1	38	37	6	9
NODE_39113_length_1957_cov_27.431529_g17930_i2	3	2	36	50
BINPACKER_24495_3	21	15	3	0
BINPACKER_14_1	4073	1617	670	392
NODE_105282_length_801_cov_29.458791_g11442_i4	0	0	20	35
NODE_89116_length_984_cov_31.419319_g47672_i0	42	26	236	206
NODE_5029_length_4498_cov_28.343955_g2563_i0	10	3	110	102
NODE_22129_length_2626_cov_26.583627_g11047_i0	7	3	81	39
NODE_39365_length_1950_cov_26.508791_g6383_i2	59	14	0	0
NODE_107160_length_783_cov_28.885915_g60065_i0	71	28	383	254
NODE_12690_length_3303_cov_21.344582_g6352_i1	22	14	0	0
BINPACKER_21195_1	3	1	28	39
Contig8570	37	15	201	159
NODE_209_length_10332_cov_31.315820_g108_i0	7	0	155	116
BINPACKER_10190_2	59	24	260	391
BINPACKER_2161_4	39	23	0	0
BINPACKER_14318_1	5	1	37	44
NODE_4706_length_4596_cov_23.638072_g2395_i0	4	4	60	63
BINPACKER_41932_1	156	65	33	24
NODE_17825_length_2884_cov_22.620064_g8859_i0	4	1	78	96
BINPACKER_14225_7	34	24	0	0
NODE_113_length_11949_cov_23.605760_g53_i0	0	0	21	24
NODE_31028_length_2231_cov_29.982391_g1851_i3	28	17	150	117
NODE_8056_length_3857_cov_237.693446_g4058_i0	233247	135082	52492	37978
NODE_19050_length_2809_cov_29.687500_g6389_i1	104	31	381	358
BINPACKER_10187_2	63	34	10	12
NODE_101460_length_840_cov_279.384615_g56022_i0	14908	5671	2006	1207
BINPACKER_9419_2	10	4	75	66
NODE_19991_length_2752_cov_27.438223_g9945_i0	10	1	130	99
NODE_74820_length_1189_cov_20.601254_g19063_i3	0	0	46	47
NODE_109685_length_758_cov_26.137226_g62011_i0	41	50	10	11
BINPACKER_2716_8	10329	2591	1592	1217
Contig7891	11038	3720	1474	917
NODE_42009_length_1876_cov_30.190238_g7261_i1	0	0	40	17
BINPACKER_17773_3	53	19	248	224
NODE_69398_length_1280_cov_32.965203_g35485_i0	12	4	67	60
BINPACKER_6301_1	132	116	55	25
BINPACKER_11104_5	6	0	74	47
BINPACKER_14225_1	0	0	33	46
Contig3734	0	0	31	25
NODE_635_length_7772_cov_30.879465_g344_i0	10	0	101	75
BINPACKER_28145_5	104	48	0	0
NODE_116334_length_697_cov_38.048077_g67330_i0	36	36	10	7
BINPACKER_2723_1	0	0	41	35
BINPACKER_13876_1	0	0	32	21
NODE_53686_length_1590_cov_17.008570_g18273_i2	1	2	38	27
BINPACKER_13620_1	0	0	20	27

BINPACKER_21293_3	34	19	123	257
BINPACKER_2578_1	0	0	240	227
NODE_145609_length_500_cov_52.327869_g92656_i0	23	8	111	88
BINPACKER_1935_8	86	72	23	14
Contig11185	7	5	162	156
BINPACKER_1736_15	56	30	0	0
BINPACKER_72698_1	768	185	77	65
BINPACKER_9_33	1476	572	234	122
NODE_10112_length_3563_cov_34.381375_g5108_i0	10885	9667	3842	3490
NODE_11587_length_3413_cov_30.869461_g5800_i0	11	2	85	56
BINPACKER_224_2	144	63	752	621
BINPACKER_7486_1	0	0	49	22
NODE_80828_length_1096_cov_23.658847_g32405_i1	0	0	14	21
Contig8351	0	0	26	28
NODE_26369_length_2414_cov_29.865869_g5216_i1	3	0	46	26
BINPACKER_1205_5	1	2	41	78
Contig4276	17	13	110	95
BINPACKER_11299_3	0	0	25	18
BINPACKER_12014_1	22	28	0	0
Contig10215	0	0	19	14
NODE_61163_length_1434_cov_5.903012_g30933_i0	7830	2962	907	590
BINPACKER_11333_7	27	16	0	0
BINPACKER_11212_2	0	0	29	14
BINPACKER_56085_1	1215	599	265	136
BINPACKER_2253_1	70	36	349	316
NODE_64957_length_1360_cov_27.984460_g32998_i0	248	126	38	28
NODE_64849_length_1362_cov_37.215671_g2133_i1	18	6	87	73
BINPACKER_1743_1	0	0	62	81
BINPACKER_13772_2	0	0	19	14
NODE_3386_length_5048_cov_33.671156_g1737_i0	3	2	41	64
BINPACKER_5433_10	114	61	0	0
NODE_89823_length_975_cov_29.166297_g48136_i0	38	12	168	159
NODE_42298_length_1868_cov_30.396657_g21142_i0	15	5	132	121
NODE_79738_length_1112_cov_28.348412_g41258_i1	255	119	57	38
NODE_83497_length_1059_cov_32.326572_g44030_i0	1933	1819	886	352
NODE_47440_length_1736_cov_29.565845_g23787_i0	260	427	134	117
BINPACKER_1807_8	18	12	89	99
BINPACKER_627_2	0	0	210	285
NODE_39372_length_1950_cov_23.713905_g1408_i4	2	0	124	123
NODE_43251_length_1842_cov_30.750141_g21629_i0	13	8	72	97
NODE_20777_length_2701_cov_26.247336_g10351_i0	2	0	22	31
NODE_32616_length_2174_cov_24.599238_g2618_i2	49	76	0	0
BINPACKER_495_1	0	0	27	22
BINPACKER_8269_5	1	0	78	22
BINPACKER_20082_3	1	6	74	70
NODE_99489_length_860_cov_26.058450_g43621_i2	19	11	118	82
BINPACKER_370_1	0	0	36	28
BINPACKER_7641_1	16	4	124	101
NODE_49912_length_1678_cov_19.519003_g25040_i0	426	349	130	55
BINPACKER_3980_3	29	22	0	0
Contig9474	22	11	113	117
NODE_38671_length_1969_cov_28.831224_g19395_i0	2085	1309	257	291
BINPACKER_7774_3	0	0	28	41
NODE_67921_length_1307_cov_35.444084_g30860_i1	118	130	27	17
BINPACKER_23437_2	68	91	15	26
NODE_98684_length_869_cov_25.770101_g54129_i0	0	0	33	40
NODE_39723_length_1939_cov_21.869775_g19908_i0	25	18	140	156
NODE_46315_length_1762_cov_31.595027_g23221_i0	205	326	71	98
BINPACKER_627_5	186	77	0	0
NODE_54692_length_1568_cov_24.355853_g27562_i0	141	135	46	47
NODE_35179_length_2083_cov_30.670149_g17626_i0	0	1	26	1670
BINPACKER_75_1	131	58	18	15
NODE_4725_length_4590_cov_24.807173_g1300_i1	29	9	132	101
NODE_71784_length_1239_cov_29.917667_g36906_i0	1346	553	175	144
NODE_71726_length_1240_cov_26.986290_g7216_i3	6	1	65	39
Contig11882	14	9	233	200
Contig7118	28286	10792	3571	2070
NODE_158145_length_443_cov_4.624324_g104246_i0	5687	2402	1235	1099
BINPACKER_376_1	515	1792	92	87
NODE_12371_length_3334_cov_31.317694_g5600_i1	0	0	57	48
BINPACKER_1516_15	0	0	68	77
BINPACKER_18532_2	0	0	51	22
Contig5088	22	5	194	134
NODE_89_length_12429_cov_28.028488_g43_i0	2523	831	502	533

BINPACKER_12996_3	6	0	54	68
BINPACKER_4611_8	0	0	42	25
BINPACKER_2578_4	117	56	0	0
BINPACKER_10217_1	202	227	88	30
Contig10910	17	39	0	0
NODE_24015_length_2525_cov_30.757749_g12037_i0	35	20	184	141
NODE_53485_length_1594_cov_23.708087_g6470_i1	21	14	0	0
Contig107	7	3	58	62
NODE_86346_length_1020_cov_31.897571_g22918_i1	0	0	14	27
BINPACKER_4692_2	0	0	48	16
NODE_21020_length_2688_cov_33.556405_g6301_i81	8	148	0	0
BINPACKER_283_11	68	49	23	15
Contig2101	50	20	226	230
NODE_71642_length_1241_cov_32.800514_g36817_i0	2	1	41	24
BINPACKER_1274_2	330	374	87	107
BINPACKER_3264_2	76	29	328	331
BINPACKER_9470_2	0	0	149	170
BINPACKER_47395_1	191	180	33	23
NODE_37534_length_2006_cov_22.611485_g18823_i0	192	225	81	66
Contig11043	46	11	313	288
NODE_60280_length_1450_cov_27.898330_g19063_i2	109	48	30	18
BINPACKER_3224_11	19	45	0	0
NODE_1550_length_6254_cov_21.148843_g837_i0	6	0	63	59
NODE_56107_length_1539_cov_9.309686_g28292_i0	3469	1568	986	718
NODE_11190_length_3455_cov_31.498817_g5600_i0	32	20	0	0
Contig7977	0	0	18	19
Contig9624	6	4	82	40
BINPACKER_95431_1	1489	355	154	116
NODE_8149_length_3844_cov_22.826836_g4104_i0	24	11	133	118
NODE_39385_length_1949_cov_33.895522_g6691_i2	0	0	55	46
BINPACKER_4_4	0	0	19	19
NODE_4125_length_4781_cov_24.155055_g2082_i0	23	9	115	110
BINPACKER_33793_1	31	11	122	124
Contig8	3	0	45	54
NODE_55018_length_1561_cov_27.586022_g27740_i0	0	0	63	41
NODE_73394_length_1212_cov_40.580334_g37844_i0	2	0	27	31
NODE_875_length_7193_cov_31.008567_g0_i26	8954	2170	0	0
Contig12297	126	95	38	39
BINPACKER_11299_1	37	13	0	0
NODE_19703_length_2767_cov_29.785820_g9823_i1	33	12	165	183
BINPACKER_20230_2	14	9	78	68
NODE_83902_length_1053_cov_37.536735_g44298_i0	21	10	123	112
BINPACKER_3203_11	0	0	127	53
BINPACKER_5740_6	4	0	48	84
BINPACKER_9271_1	8	3	57	55
NODE_57469_length_1510_cov_29.632568_g28978_i0	715	776	308	131
BINPACKER_3574_6	0	0	49	41
BINPACKER_6475_20	25	27	2	1
BINPACKER_7001_3	16	12	0	0
BINPACKER_11666_3	0	0	19	25
NODE_47868_length_1727_cov_28.326481_g23850_i1	136	45	0	0
BINPACKER_7391_11	3	3	61	107
BINPACKER_631_6	113	121	34	30
BINPACKER_7553_1	0	0	22	17
NODE_198620_length_317_cov_1.045082_g142860_i0	130	154	25	38
NODE_44498_length_1808_cov_28.997695_g22281_i0	49	39	10	15
NODE_49520_length_1687_cov_29.903346_g24837_i0	215	131	40	23
NODE_18736_length_2829_cov_25.564224_g9340_i0	24	9	98	159
BINPACKER_20985_1	50	10	239	208
BINPACKER_5619_12	0	0	49	17
BINPACKER_3622_5	27	12	0	0
BINPACKER_4945_5	51	8	415	426
NODE_218_length_10247_cov_31.183900_g113_i0	27	7	132	147
NODE_56911_length_1522_cov_28.762595_g28691_i0	9	7	68	70
Contig12375	57	10	317	274
NODE_58754_length_1483_cov_24.571631_g26965_i2	133	172	8	0
Contig3905	34	17	274	244
BINPACKER_555_10	70	11	460	335
BINPACKER_13766_3	95	40	370	488
BINPACKER_8269_4	10	0	110	62
BINPACKER_337_5	2	0	42	20
BINPACKER_8843_3	0	0	16	84
NODE_80759_length_1097_cov_28.265625_g42299_i0	152	187	37	28
NODE_113199_length_725_cov_34.136503_g64816_i0	0	0	15	17

Contig6487	10	7	62	69
BINPACKER_17821_3	0	0	46	84
BINPACKER_46_3	1150	1134	102	98
NODE_3096_length_5171_cov_30.269714_g1045_i1	17	7	137	162
NODE_17897_length_2879_cov_32.516393_g8889_i0	18	10	132	75
NODE_27962_length_2347_cov_23.562005_g14038_i0	34	16	172	179
NODE_3954_length_4848_cov_26.911623_g1991_i0	149	23	760	734
BINPACKER_2461_5	20	10	0	0
BINPACKER_12853_5	116	141	30	35
BINPACKER_59_5	207	47	8	17
BINPACKER_830_5	5	4	44	60
Contig6996	9807	5211	2046	1491
BINPACKER_15835_4	10	3	52	66
NODE_29650_length_2282_cov_33.137619_g14886_i0	19	12	135	88
NODE_2057_length_5796_cov_30.540800_g1080_i0	33	4	236	199
NODE_77447_length_1147_cov_21.022346_g21017_i2	144	157	57	19
BINPACKER_12252_2	16	5	104	121
BINPACKER_3965_6	0	0	23	19
NODE_74558_length_1193_cov_29.506250_g38535_i0	123	136	50	20
BINPACKER_335_7	79830	85326	9883	10706
NODE_2063_length_5793_cov_25.525699_g456_i2	17	2	98	83
BINPACKER_12853_2	100	135	27	29
Contig6666	345	282	70	52
BINPACKER_3574_3	41	16	0	0
BINPACKER_833_3	4	0	43	38
BINPACKER_1614_1	7742	11519	2415	2308
NODE_2281_length_5671_cov_33.391568_g780_i3	7	2	123	87
BINPACKER_15159_4	68	14	273	297
NODE_71321_length_1247_cov_24.484668_g33464_i1	0	0	18	25
NODE_43689_length_1831_cov_28.917520_g4080_i2	0	0	186	130
NODE_23493_length_2553_cov_27.755242_g11735_i0	15	4	117	57
Contig7614	156907	39230	16338	11200
NODE_16394_length_2994_cov_32.117083_g8141_i0	0	0	22	26
NODE_53142_length_1602_cov_15.655984_g26729_i0	17	5	80	74
NODE_48763_length_1705_cov_30.152574_g24438_i0	12	10	94	94
NODE_60014_length_1455_cov_30.783647_g30320_i0	3	0	33	28
BINPACKER_1177_8	745	370	3233	3522
BINPACKER_117235_1	477	142	64	53
BINPACKER_869_4	80	46	21	16
NODE_98777_length_868_cov_21.090566_g54202_i0	9	7	95	59
Contig3190	0	0	14	25
Contig7048	98	50	21	5
NODE_8622_length_3766_cov_36.254806_g2948_i1	30968	13970	5949	3532
Contig7942	459779	277855	150687	107475
NODE_3061_length_5189_cov_26.893276_g1569_i0	22	11	161	221
BINPACKER_2187_1	1316	1543	322	359
NODE_41225_length_1897_cov_30.594298_g12887_i1	0	0	46	46
BINPACKER_4117_8	73	53	0	0
NODE_9780_length_3603_cov_115.428329_g4942_i0	83676	25696	11308	6913
Contig3130	76	23	335	432
BINPACKER_1736_23	0	0	84	76
BINPACKER_8732_6	21	4	111	81
Contig4402	0	0	14	24
BINPACKER_6268_11	0	0	214	41
BINPACKER_375_7	44	10	190	168
BINPACKER_18589_1	5	2	47	36
BINPACKER_15128_2	0	0	24	21
NODE_85387_length_1034_cov_4.792924_g45276_i0	72	51	21	15
NODE_235_length_10039_cov_29.675597_g32_i4	123	89	0	0
BINPACKER_7853_4	31	10	135	133
NODE_42111_length_1873_cov_27.469444_g21045_i0	1	0	49	42
NODE_27886_length_2349_cov_50.690685_g13995_i0	0	0	8	697
BINPACKER_5641_6	47	14	275	221
BINPACKER_15274_4	28	23	0	0
NODE_248_length_9957_cov_29.699110_g132_i0	2586	1041	359	381
NODE_2130_length_5751_cov_29.685981_g1105_i0	18	8	212	227
NODE_8877_length_3729_cov_32.091904_g4483_i0	5	0	67	57
NODE_6291_length_4175_cov_27.213067_g3186_i0	9	1	87	87
NODE_80068_length_1107_cov_34.284333_g41882_i0	51	185	16	7
Contig4882	0	0	122	122
BINPACKER_29842_1	17	11	146	289
NODE_1442_length_6375_cov_26.719930_g780_i0	2	0	40	24
BINPACKER_7879_6	0	0	33	25
BINPACKER_2511_14	23	11	0	0

Contig8714	56	48	10	6
Contig214	0	0	27	13
NODE_44104_length_1819_cov_26.987973_g8997_i5	604	695	205	160
BINPACKER_1164_1	6	4	51	71
Contig3874	6	12	126	108
NODE_45189_length_1791_cov_24.700815_g22652_i0	1691	877	256	252
NODE_36257_length_2047_cov_28.080041_g18187_i0	91	25	366	288
NODE_9547_length_3636_cov_27.134718_g4676_i2	0	0	29	91
BINPACKER_5175_2	2	0	23	25
NODE_32240_length_2188_cov_26.717730_g16133_i0	9	6	89	68
NODE_36144_length_2051_cov_22.107685_g9688_i1	69	71	17	22
BINPACKER_7553_7	39	23	0	2

TableS1c.Blastx results for DEGs

NODE_61163_length_1434_cov_5.903012_g30933_i0	sp_A1E9Q2_RR12_SORBI_30S_ribosomal_protein_S12,_chloroplastic_OS=Sorghum_bicolor_OX=4558_GN=rps12-A_PE=3_SV=1	81.967	61	11	0	1432	1250	56	116	1.33E-25	104
NODE_875_length_7193_cov_31.008567_g0_i26	sp_A1E9S3_PSAB_SORBI_Photosystem_I_P700_chlorophyll_a_apoprotein_A2_OS=Sorghum_bicolor_OX=4558_GN=psaB_PE=3_SV=1	99.863	729	1	0	1789	3975	1	729	0	1425
NODE_89_length_12429_cov_28.028488_g43_i0	sp_A1E9T7_CYF_SORBI_Cytochrome_f_OS=Sorghum_bicolor_OX=4558_GN=petA_PE=3_SV=1	99.062	320	3	0	3197	4156	1	320	0	638
NODE_38671_length_1969_cov_28.831224_g19395_i0	sp_A1E9V0_PSBB_SORBI_Photosystem_II_CP47_reaction_center_protein_OS=Sorghum_bicolor_OX=4558_GN=psbB_PE=3_SV=1	99.467	375	2	0	1969	845	133	507	0	731
BINPACKER_376_1	sp_A1E9X3_CCSA_SORBI_Cytochrome_c_biogenesis_protein_CcsA_OS=Sorghum_bicolor_OX=4558_GN=ccsA_PE=3_SV=1	92.593	324	21	1	1553	582	1	321	5.4E-163	479
BINPACKER_370_1	sp_A2XFB7_PRR73_ORYSI_Two-component_response_regulator-like_PRR73_OS=Oryza_sativa_subsp._indica_OX=39946_GN=PRR73_PE=2_SV=2	77.5	160	35	1	645	1121	1	160	6.12E-96	253
Contig5088	sp_A4GSN8_NUA_ARATH_Nuclear-pore_anchor_OS=Arabidopsis_thaliana_OX=3702_GN=NUA_PE=1_SV=1	44.79	1833	880	30	6348	1153	1	1802	0	1234
NODE_1550_length_6254_cov_21.148843_g837_i0	sp_A4GSN8_NUA_ARATH_Nuclear-pore_anchor_OS=Arabidopsis_thaliana_OX=3702_GN=NUA_PE=1_SV=1	48.189	1436	707	13	6190	1943	1	1419	0	1090
BINPACKER_8269_4	sp_B5BT18_BTAF1_ARATH_TATA-binding_protein-associated_factor_BTAF1_OS=Arabidopsis_thaliana_OX=3702_GN=BTAF1_PE=1_SV=1	50.419	597	249	12	317	2029	4	579	1.5E-151	494
BINPACKER_8269_5	sp_B5BT18_BTAF1_ARATH_TATA-binding_protein-associated_factor_BTAF1_OS=Arabidopsis_thaliana_OX=3702_GN=BTAF1_PE=1_SV=1	53.416	805	330	11	332	2683	4	784	0	698
BINPACKER_4_4	sp_B9FK36_ACC2_ORYSJ_Acetyl-CoA_carboxylase_2_OS=Oryza_sativa_subsp._japonica_OX=39947_GN=ACC2_PE=3_SV=2	84.865	2339	326	9	361	7341	4	2326	0	3989
NODE_635_length_7772_cov_30.879465_g344_i0	sp_B9G2A8_BIG_ORYSJ_Auxin_transport_protein_BIG_OS=Oryza_sativa_subsp._japonica_OX=39947_GN=Os09g0247700_PE=2_SV=1	80.339	1770	331	8	2492	7771	673	2435	0	2699
NODE_8149_length_3844_cov_22.826836_g4104_i0	sp_F4HRS2_SWTIE_ARATH_Protein_SWEETIE_OS=Arabidopsis_thaliana_OX=3702_GN=SWEETIE_PE=1_SV=2	61.94	402	152	1	1206	1	637	1037	1.9E-141	479
BINPACKER_375_7	sp_F4HRS2_SWTIE_ARATH_Protein_SWEETIE_OS=Arabidopsis_thaliana_OX=3702_GN=SWEETIE_PE=1_SV=2	48.416	1673	782	21	6339	1462	407	2045	0	1361
Contig12375	sp_F4HZB2_BCHA1_ARATH_Protein_SPIRRIG_OS=Arabidopsis_thaliana_OX=3702_GN=SPI_PE=1_SV=1	65.488	3564	1141	30	11223	679	62	3585	0	4474
NODE_3386_length_5048_cov_33.671156_g1737_i0	sp_F4HZB5_NET1D_ARATH_Protein_NETWORKED_1D_OS=Arabidopsis_thaliana_OX=3702_GN=NET1D_PE=3_SV=1	48.814	295	106	7	5048	4173	13	265	7.02E-70	264
BINPACKER_2723_1	sp_F4I4P3_MED23_ARATH_Mediator_of_RNA_polymerase_II_transcription_subunit_23_OS=Arabidopsis_thaliana_OX=3702_GN=MED23_PE=1_SV=1	51.282	390	174	5	407	1567	21	397	1.9E-116	393
NODE_43576_length_1834_cov_31.222033_g21802_i0	sp_F4I893_ILA_ARATH_Protein_ILITYHIA_OS=Arabidopsis_thaliana_OX=3702_GN=ILA_PE=1_SV=1	54.111	523	237	3	1576	14	142	663	3.2E-180	568
BINPACKER_6268_11	sp_F4I893_ILA_ARATH_Protein_ILITYHIA_OS=Arabidopsis_thaliana_OX=3702_GN=ILA_PE=1_SV=1	65.646	2253	703	9	805	7518	413	2609	0	2709
NODE_209_length_10332_cov_31.315820_g108_i0	sp_F4I9T0_BCHB_ARATH_BEACH_domain-containing_protein_B_OS=Arabidopsis_thaliana_OX=3702_GN=BCHB_PE=4_SV=1	73.367	199	53	0	2836	2240	1867	2065	1.54E-77	292
NODE_113_length_11949_cov_23.605760_g53_i0	sp_F4IAT2_THOC2_ARATH_THO_complex_subunit_2_OS=Arabidopsis_thaliana_OX=3702_GN=THO2_PE=1_SV=1	64.217	517	167	4	10449	11948	88	603	0	668
BINPACKER_2161_4	sp_F4ICK8_IWS1_ARATH_Protein_IWS1_homolog_1_OS=Arabidopsis_thaliana_OX=3702_GN=IWS1_PE=1_SV=1	69.315	365	101	4	1570	503	140	502	1.2E-138	418
NODE_218_length_10247_cov_31.183900_g113_i0	sp_F4IG73_BCHC2_ARATH_BEACH_domain-containing_protein_C2_OS=Arabidopsis_thaliana_OX=3702_GN=BCHC2_PE=1_SV=1	51.603	312	130	5	8644	9576	2698	2989	1.62E-68	263
BINPACKER_555_10	sp_F4IHS2_SYD_ARATH_Chromatin_structure-remodeling_complex_protein_SYD_OS=Arabidopsis_thaliana_OX=3702_GN=SYD_PE=1_SV=1	76.034	943	212	5	13958	11166	453	1393	0	1271
NODE_30996_length_2232_cov_32.053265_g15531_i0	sp_F4IIM1_CSI1_ARATH_Protein_CELLULOSE_SYNTHASE_INTERACTIVE_1_OS=Arabidopsis_thaliana_OX=3702_GN=CSI1_PE=1_SV=1	76.682	639	144	5	1906	2	1	638	0	905
Contig8351	sp_F4IN58_PIEZO_ARATH_Piezo-type_mechanosensitive_ion_channel_homolog_OS=Arabidopsis_thaliana_OX=3702_GN=At2g48060/At2g48040/At2g48050_PE=2_SV=	59.494	316	127	1	1521	2468	483	797	7.7E-107	365
NODE_4725_length_4590_cov_24.807173_g1300_i1	sp_F4IS91_RSA1_ARATH_Protein_SHORT_ROOT_IN_SALT_MEDIUM_1_OS=Arabidopsis_thaliana_OX=3702_GN=RSA1_PE=1_SV=1	43.195	338	124	6	3156	2155	441	714	7.2E-72	269
NODE_3096_length_5171_cov_30.269714_g1045_i1	sp_F4IVL6_GRV2_ARATH_DnaJ_homolog_subfamily_C_GRV2_OS=Arabidopsis_thaliana_OX=3702_GN=GRV2_PE=1_SV=1	67.728	1664	450	17	248	5170	22	1621	0	2077
BINPACKER_7774_3	sp_F4IXW2_BIG5_ARATH_Brefeldin_A-inhibited_guanine_nucleotide-exchange_protein_5_OS=Arabidopsis_thaliana_OX=3702_GN=BIG5_PE=1_SV=2	69.79	1526	417	12	5221	707	235	1737	0	2091
NODE_56911_length_1522_cov_28.762595_g28691_i0	sp_F4J6F6_IREH1_ARATH_Probable_serine/threonine_protein_kinase_IREH1_OS=Arabidopsis_thaliana_OX=3702_GN=IREH1_PE=1_SV=1	58.708	511	197	6	1503	1	266	772	0	585
Contig3734	sp_F4J8G7_VIR_ARATH_Protein_virilizer_homolog_OS=Arabidopsis_thaliana_OX=3702_GN=VIR_PE=1_SV=1	43.002	1779	928	34	6901	1637	62	1778	0	1154
NODE_23493_length_2553_cov_27.755242_g11735_i0	sp_F4J9M5_CHR12_ARATH_Probable_ATP-dependent_DNA_helicase_CHR12_OS=Arabidopsis_thaliana_OX=3702_GN=CHR12_PE=2_SV=1	61.777	484	175	4	2045	606	73	550	2.1E-155	486
NODE_73394_length_1212_cov_40.580334_g37844_i0	sp_F4JFN3_HS906_ARATH_Heat_shock_protein_90-6,_mitochondrial_OS=Arabidopsis_thaliana_OX=3702_GN=HSP90-6_PE=1_SV=1	71.246	313	87	2	934	2	44	355	7.1E-121	372
BINPACKER_1743_1	sp_F4JGZ1_MED16_ARATH_Mediator_of_RNA_polymerase_II_transcription_subunit_16_OS=Arabidopsis_thaliana_OX=3702_GN=MED16_PE=1_SV=1	68.351	1207	343	14	565	4113	83	1274	0	1535
NODE_5798_length_4289_cov_26.403463_g2737_i2	sp_F4JKH6_TSS_ARATH_Protein_TSS_OS=Arabidopsis_thaliana_OX=3702_GN=TSS_PE=1_SV=1	55.54	704	256	7	504	2489	24	712	0	659
NODE_3954_length_4848_cov_26.911623_g1991_i0	sp_F4JKH6_TSS_ARATH_Protein_TSS_OS=Arabidopsis_thaliana_OX=3702_GN=TSS_PE=1_SV=1	55.733	750	303	6	1520	3760	284	1007	0	776
Contig4402	sp_F4JSZ5_BIG1_ARATH_Brefeldin_A-inhibited_guanine_nucleotide-exchange_protein_1_OS=Arabidopsis_thaliana_OX=3702_GN=BIG1_PE=2_SV=1	60.45	1555	575	15	821	5419	149	1685	0	1890
NODE_3061_length_5189_cov_26.893276_g1569_i0	sp_F4K5J1_MYO17_ARATH_Myosin-17_OS=Arabidopsis_thaliana_OX=3702_GN=XI-K_PE=1_SV=2	83.019	689	116	1	245	2308	1	689	0	1233
NODE_6290_length_4175_cov_30.597513_g3185_i0	sp_F4KBP5_CHR4_ARATH_Protein_CHROMATIN_REMODELING_4_OS=Arabidopsis_thaliana_OX=3702_GN=CHR4_PE=2_SV=1	73.399	812	206	4	2700	289	535	1344	0	1186
NODE_4125_length_4781_cov_24.155055_g2082_i0	sp_F4KCC2_PRT6_ARATH_E3_ubiquitin-protein_ligase_PRT6_OS=Arabidopsis_thaliana_OX=3702_GN=PRT6_PE=2_SV=1	50.725	483	215	3	1794	3233	676	1138	3E-128	446
BINPACKER_2578_1	sp_H3K2Y6_MED12_ARATH_Mediator_of_RNA_polymerase_II_transcription_subunit_12_OS=Arabidopsis_thaliana_OX=3702_GN=MED12_PE=1_SV=1	45.494	2297	1097	52	541	7152	1	2235	0	1630
BINPACKER_2578_4	sp_H3K2Y6_MED12_ARATH_Mediator_of_RNA_polymerase_II_transcription_subunit_12_OS=Arabidopsis_thaliana_OX=3702_GN=MED12_PE=1_SV=1	45.451	2297	1098	52	494	7105	1	2235	0	1627
NODE_17825_length_2884_cov_22.620064_g8859_i0	sp_K7U9N8_OP1_MAIZE_Protein_OPAQUE1_OS=Zea_mays_OX=4577_GN=O1_PE=1_SV=1	93.253	415	27	1	358	1599	28	442	0	777
NODE_39385_length_1949_cov_33.895522_g6691_i2	sp_O04647_PP399_ARATH_Pentatricopeptide_repeat-containing_protein_At5g27270_OS=Arabidopsis_thaliana_OX=3702_GN=EMB976_PE=2_SV=2	19.62	316	219	2	1573	626	751	1031	1.08E-06	55.8
NODE_47868_length_1727_cov_28.326481_g23850_i1	sp_O22174_ERF08_ARATH_Ethylene-responsive_transcription_factor_ERF008_OS=Arabidopsis_thaliana_OX=3702_GN=ERF008_PE=2_SV=1	58.519	135	44	3	1121	744	33	164	2.69E-25	106
BINPACKER_29842_1	sp_O22941_IDE1_ARATH_Insulin-degrading_enzyme-like_1,_peroxisomal_OS=Arabidopsis_thaliana_OX=3702_GN=PXM16_PE=2_SV=1	62.269	432	162	1	3270	1978	13	444	0	570
BINPACKER_17773_3	sp_O23461_ARAK_ARATH_L-arabinokinase_OS=Arabidopsis_thaliana_OX=3702_GN=ARA1_PE=1_SV=1	82.883	333	51	1	2038	1040	46	372	0	579
Contig3905	sp_O48847_LUH_ARATH_Transcriptional_corepressor_LEUNIG_HOMOLOG_OS=Arabidopsis_thaliana_OX=3702_GN=LUH_PE=1_SV=1	61.994	692	230	12	293	2341	1	668	0	756
BINPACKER_3224_11	sp_O65717_CNGC1_ARATH_Cyclic_nucleotide-gated_ion_channel_1_OS=Arabidopsis_thaliana_OX=3702_GN=CNGC1_PE=1_SV=1	59.613	723	265	10	2423	306	4	716	0	813
NODE_17897_length_2879_cov_32.516393_g8889_i0	sp_O80738_TPS10_ARATH_Probable_alpha,alpha-trehalose-phosphate_synthase_[UDP-forming]_10_OS=Arabidopsis_thaliana_OX=3702_GN=TPS10_PE=2_SV=1	62.636	827	308	1	2879	402	35	861	0	1114
NODE_74558_length_1193_cov_29.506250_g38535_i0	sp_O80920_PYL4_ARATH_Abscisic_acid_receptor_PYL4_OS=Arabidopsis_thaliana_OX=3702_GN=PYL4_PE=1_SV=1	65.663	166	52	3	225	719	40	201	2.44E-51	174
BINPACKER_10217_1	sp_O80920_PYL4_ARATH_Abscisic_acid_receptor_PYL4_OS=Arabidopsis_thaliana_OX=3702_GN=PYL4_PE=1_SV=1	69.032	155	44	2	1162	701	50	201	1.31E-49	172
NODE_46315_length_1762_cov_31.595027_g23221_i0	sp_P03905_NU4M_HUMAN_NADH-ubiquinone_oxidoreductase_chain_4_OS=Homo_sapiens_OX=9606_GN=MT-ND4_PE=1_SV=1	92.421	409	31	0	1285	59	51	459	1.6E-175	508
NODE_45189_length_1791_cov_24.700815_g22652_i0	sp_P05642_CYB6_MAIZE_Cytochrome_b6_OS=Zea_mays_OX=4577_GN=petB_PE=2_SV=2	99.07	215	2	0	910	266	1	215	8.3E-151	435
BINPACKER_21195_1	sp_P08926_RUBA_PEA_RuBisCO_large_subunit-binding_protein_subunit_alpha,_chloroplastic_OS=Pisum_sativum_OX=3888_PE=1_SV=2	72.727	176	37	3	495	1	1	176	1.29E-76	243
NODE_10112_length_3563_cov_34.381375_g5108_i0	sp_P0C314_CLPP_ORYSJ_ATP-dependent_Clp_protease_proteolytic_subunit_OS=Oryza_sativa_subsp._japonica_OX=39947_GN=clpP_PE=3_SV=1	98.611	216	3	0	980	1627	1	216	1.5E-146	442
BINPACKER_41932_1	sp_P0C319_PETD_ORYSJ_Cytochrome_b6-f_complex_subunit_4_OS=Oryza_sativa_subsp._japonica_OX=39947_GN=petD_PE=3_SV=1	100	119	0	0	359	3	1	119	3.03E-80	239
BINPACKER_335_7	sp_P0C355_PSAA_ORYSJ_Photosystem_I_P700_chlorophyll_a_apoprotein_A1_OS=Oryza_sativa_subsp._japonica_OX=39947_GN=psaA_PE=3_SV=1	96.277	188	7	0	2733	3296	1	188	1.2E-112	375
BINPACKER_9419_2	sp_P0DM58_DI3L2_ARATH_DIS3-like_exonuclease_2_OS=Arabidopsis_thaliana_OX=3702_GN=SOV_PE=1_SV=1	47.269	952	454	12	3314	471	148	1055	0	794
BINPACKER_9778_1	sp_P11734_CU08_LOCMI_Cuticle_protein_8_OS=Locusta_migratoria_OX=7004_PE=1_SV=1	65.517	58	19	1	230	403	63	119	1.67E-13	70.5
NODE_65062_length_1358_cov_111.919066_g27862_i51	sp_P12329_CB21_MAIZE_Chlorophyll_a-b_binding_protein_1,_chloroplastic_OS=Zea_mays_OX=4577_GN=CAB1_PE=1_SV=1	91.571	261	22	0	1334	552	2	262	1.1E-173	490
NODE_64957_length_1360_cov_27.984460_g32998_i0	sp_P17703_RR15_MAIZE_30S_ribosomal_protein_S15,_chloroplastic_OS=Zea_mays_OX=4577_GN=rps15-A_PE=3_SV=1	97.778	90	2	0	406	137	1	90	4.15E-39	139
NODE_109685_length_758_cov_26.137226_g62011_i0	sp_P18124_RL7_HUMAN_60S_ribosomal_protein_L7_OS=Homo_sapiens_OX=9606_GN=RPL7_PE=1_SV=1	97.845	232	5	0	58	753	17	248	3.4E-154	431
Contig7048	sp_P20026_MYB1_HORVU_Myb-related_protein_Hv1_OS=Hordeum_vulgare_OX=4513_GN=MYB1_PE=2_SV=1	67.524	311	32	9	893	1750	1	267	2.5E-125	373
NODE_49520_length_1687_cov_29.903346_g24837_i0	sp_P20026_MYB1_HORVU_Myb-related_protein_Hv1_OS=Hordeum_vulgare_OX=4513_GN=MYB1_PE=2_SV=1	75	268	40	6	1109	384	1	267	9.9E-132	387
NODE_27962_length_2347_cov_23.562005_g14038_i0	sp_P23225_GLTB_MAIZE_Ferredoxin-dependent_glutamate_synthase,_chloroplastic_OS=Zea_mays_OX=4577_GN=GLSF_PE=1_SV=1	100	33	0	0	3	101	439	471	8.77E-13	76.3
NODE_15365_length_3072_cov_30.894632_g7657_i0	sp_P24794_COX1_BETVU_Cytochrome_c_oxidase_subunit_1_OS=Beta_vulgaris_OX=161934_GN=COX1_PE=3_SV=2	98.844	519	6	0	1809	253	1	519	0	955
BINPACKER_283_11	sp_P26517_G3PC1_HORVU_Glyceraldehyde-3-phosphate_dehydrogenase_1,_cytosolic_OS=Hordeum_vulgare_OX=4513_GN=GAPC_PE=2_SV=1	94.362	337	19	0	1498	488	1	337	0	623
BINPACKER_9470_2	sp_P32456_GBP2_HUMAN_Guanylate-binding_protein_2_OS=Homo_sapiens_OX=9606_GN=GBP2_PE=1_SV=3	36.088	363	218	7	2748	1684	10	366	2.56E-65	234
BINPACKER_631_6	sp_P33679_ZEAM_MAIZE_Zeamatin_OS=Zea_mays_OX=4577_GN=Zlp_PE=1_SV=2	55.714	210	76	6	3016	3603	21	227	1.05E-66	228
Contig12297	sp_P35684_RL3_ORYSJ_60S_ribosomal_protein_L3_OS=Oryza_sativa_subsp._japonica_OX=39947_GN=RPL3_PE=2_SV=2	71.429	98	17	1	643	383	236	333	3.93E-40	144
BINPACKER_20985_1	sp_P40340_TBP7_YEAST_Tat-binding_homolog_7_OS=Saccharomyces_cerevisiae_(strain_ATCC_204508_/_S288c)_OX=559292_GN=YTA7_PE=1_SV=2	38.163	490	245	9	4527	3058	410	841	4.05E-91	331
BINPACKER_12014_1	sp_P40468_TAO3_YEAST_Cell_morphogenesis_protein_PAG1_OS=Saccharomyces_cerevisiae_(strain_ATCC_204508_/_S288c)_OX=559292_GN=TAO3_PE=1_SV=1	23.481	362	229	5	2473	1391	1775	2089	4.73E-19	97.1
NODE_248_length_9957_cov_29.699110_g132_i0	sp_P46620_NU5C_MAIZE_NAD(P)H-quinone_oxidoreductase_subunit_5,_chloroplastic_OS=Zea_mays_OX=4577_GN=ndhF_PE=3_SV=1	95.222	586	28	0	1846	89	146	731	0	1016
NODE_18736_length_2829_cov_25.564224_g9340_i0	sp_P53393_SUT3_STYHA_Low_affinity_sulfate_transporter_3_OS=Stylosanthes_hamata_OX=37660_GN=ST3_PE=2_SV=1	58.171	667	238	4	2470	491	11	643	0	719
NODE_107160_length_783_cov_28.885915_g60065_i0	sp_P54873_HMCS_ARATH_Hydroxymethylglutaryl-CoA_synthase_OS=Arabidopsis_thaliana_OX=3702_GN=HMGS_PE=1_SV=2	73.152	257	35	1	781	11	5	227	2.1E-132	385
NODE_47440_length_1736_cov_29.565845_g23787_i0	sp_P68105_EF1A1_RABIT_Elongation_factor_1-alpha_1_OS=Oryctolagus_cuniculus_OX=9986_GN=EEF1A1_PE=1_SV=1	100	432	0	0	54	1349	1	432	0	895
NODE_49912_length_1678_cov_19.519003_g25040_i0	sp_P81713_IBB3_WHEAT_Bowman-Birk_type_trypsin_inhibitor_OS=Triticum_aestivum_OX=4565_PE=1_SV=1	55.357	56	24	1	1510	1343	8	62	4.05E-15	73.6
NODE_80759_length_1097_cov_28.265625_g42299_i0	sp_P81713_IBB3_WHEAT_Bowman-Birk_type_trypsin_inhibitor_OS=Triticum_aestivum_OX=4565_PE=1_SV=1	56.923	65	27	1	146	340	7	70	9.85E-20	85.5
BINPACKER_4611_8	sp_P85193_PK01_HELAN_Putative_serine/threonine-protein_kinase_(Fragment)_OS=Helianthus_annuus_OX=4232_PE=1_SV=1	40.625	160	91	2	907	437	35	193	2.35E-33	132
NODE_79022_length_1123_cov_30.629524_g41258_i0	sp_P93176_TBB_HORVU_Tubulin_beta_chain_OS=Hordeum_vulgare_OX=4513_GN=TUBB_PE=2_SV=1	100	347	0	0	82	1122	1	347	0	702
NODE_79738_length_1112_cov_28.348412_g41258_i1	sp_P93176_TBB_HORVU_Tubulin_beta_chain_OS=Hordeum_vulgare_OX=4513_GN=TUBB_PE=2_SV=1	100	347	0	0	1042	2	1	347	0	703
BINPACKER_224_2	sp_P93647_LONP2_MAIZE_Lon_protease_homolog_2,_peroxisomal_OS=Zea_mays_OX=4577_GN=LON1_PE=2_SV=1	95.329	471	22	0	265	1677	1	471	0	848
NODE_101460_length_840_cov_279.384615_g56022_i0	sp_Q03663_GSTX2_TOBAC_Probable_glutathione_S-transferase_OS=Nicotiana_tabacum_OX=4097_PE=2_SV=1	60	50	20	0	691	840	26	75	7.98E-12	66.6
BINPACKER_7001_3	sp_Q09263_EFR3_CAEEL_Protein_EFR3_homolog_OS=Caenorhabditis_elegans_OX=6239_GN=efr-3_PE=3_SV=1	20.896	469	312	15	3948	2635	13	453	2.65E-06	56.6
NODE_19991_length_2752_cov_27.438223_g9945_i0	sp_Q0D3F2_NAS3_ORYSJ_Nicotianamine_synthase_3_OS=Oryza_sativa_subsp._japonica_OX=39947_GN=NAS3_PE=2_SV=1	82.132	319	46	4	2674	1748	25	342	2E-162	481
Contig11185	sp_Q0DJS1_TOR_ORYSJ_Serine/threonine-protein_kinase_TOR_OS=Oryza_sativa_subsp._japonica_OX=39947_GN=TOR_PE=1_SV=3	91.176	34	3	0	4062	3961	1	34	3.28E-11	72.4
BINPACKER_15159_4	sp_Q0DJS1_TOR_ORYSJ_Serine/threonine-protein_kinase_TOR_OS=Oryza_sativa_subsp._japonica_OX=39947_GN=TOR_PE=1_SV=3	93.007	1716	116	2	5593	458	154	1869	0	3169
BINPACKER_18589_1	sp_Q0E3C8_CLPB3_ORYSJ_Chaperone_protein_ClpB3,_mitochondrial_OS=Oryza_sativa_subsp._japonica_OX=39947_GN=CLPB3_PE=2_SV=3	93.75	608	35	1	700	2523	79	683	0	1094
BINPACKER_3264_2	sp_Q0IQN5_RTOR2_ORYSJ_Regulatory-associated_protein_of_TOR_2_OS=Oryza_sativa_subsp._japonica_OX=39947_GN=RAPTOR2_PE=1_SV=2	89.864	1105	104	5	414	3710	27	1129	0	1830
BINPACKER_33793_1	sp_Q0J6P7_KAD5_ORYSJ_Probable_adenylate_kinase_5,_chloroplastic_OS=Oryza_sativa_subsp._japonica_OX=39947_GN=Os08g0288200_PE=2_SV=2	84.122	296	46	1	194	1078	63	358	2E-171	493
BINPACKER_11298_1	sp_Q0JDM0_C3H27_ORYSJ_Zinc_finger_CCCH_domain-containing_protein_27_OS=Oryza_sativa_subsp._japonica_OX=39947_GN=Os04g0394300_PE=2_SV=2	74.75	899	202	11	3383	726	1	887	0	1195
BINPACKER_11298_4	sp_Q0JDM0_C3H27_ORYSJ_Zinc_finger_CCCH_domain-containing_protein_27_OS=Oryza_sativa_subsp._japonica_OX=39947_GN=Os04g0394300_PE=2_SV=2	74.861	899	202	10	3386	726	1	887	0	1200
NODE_19050_length_2809_cov_29.687500_g6389_i1	sp_Q0JKD0_GLT1_ORYSJ_Glutamate_synthase_1_[NADH],_chloroplastic_OS=Oryza_sativa_subsp._japonica_OX=39947_GN=Os01g0681900_PE=2_SV=1	94.225	935	54	0	3	2807	266	1200	0	1754
NODE_96083_length_899_cov_28.601695_g52331_i0	sp_Q0JKD0_GLT1_ORYSJ_Glutamate_synthase_1_[NADH],_chloroplastic_OS=Oryza_sativa_subsp._japonica_OX=39947_GN=Os01g0681900_PE=2_SV=1	78.893	289	55	3	854	3	1	288	4.9E-123	390
NODE_71642_length_1241_cov_32.800514_g36817_i0	sp_Q0WL80_UGGG_ARATH_UDP-glucose:glycoprotein_glucosyltransferase_OS=Arabidopsis_thaliana_OX=3702_GN=UGGT_PE=1_SV=1	60.714	336	110	4	1007	3	28	342	4.6E-130	412
Contig214	sp_Q0WPK4_PAT1H_ARATH_Protein_PAT1_homolog_OS=Arabidopsis_thaliana_OX=3702_GN=PAT1_PE=1_SV=1	44.72	767	342	23	3117	892	73	782	6.6E-145	464
BINPACKER_1164_1	sp_Q0WVE9_PLST1_ARATH_Probable_plastidic_glucose_transporter_1_OS=Arabidopsis_thaliana_OX=3702_GN=At1g05030_PE=2_SV=2	70.196	255	70	3	841	1593	165	417	7.1E-111	354
NODE_98684_length_869_cov_25.770101_g54129_i0	sp_Q10064_YAMB_SCHPO_Uncharacterized_PI3/PI4-kinase_family_protein_C1F5.11c_OS=Schizosaccharomyces_pombe_(strain_972_/_ATCC_24843)_OX=284812_GN	36.134	119	68	5	2	346	817	931	1.03E-11	68.9
NODE_99489_length_860_cov_26.058450_g43621_i2	sp_Q10716_CYSP1_MAIZE_Cysteine_proteinase_1_OS=Zea_mays_OX=4577_GN=CCP1_PE=2_SV=1	78.824	170	29	2	371	859	23	192	1.74E-89	273
BINPACKER_182_10	sp_Q10L71_VLN2_ORYSJ_Villin-2_OS=Oryza_sativa_subsp._japonica_OX=39947_GN=VLN2_PE=2_SV=1	86.313	358	47	2	421	1491	7	363	0	652
Contig8570	sp_Q10LP5_SUS4_ORYSJ_Sucrose_synthase_4_OS=Oryza_sativa_subsp._japonica_OX=39947_GN=SUS4_PE=2_SV=1	93.043	230	16	0	7099	6410	1	230	4.4E-133	442
NODE_43251_length_1842_cov_30.750141_g21629_i0	sp_Q10R54_SUT1_ORYSJ_Sucrose_transport_protein_SUT1_OS=Oryza_sativa_subsp._japonica_OX=39947_GN=SUT1_PE=1_SV=1	87.526	481	60	0	2	1444	56	536	0	848
BINPACKER_1205_5	sp_Q14692_BMS1_HUMAN_Ribosome_biogenesis_protein_BMS1_homolog_OS=Homo_sapiens_OX=9606_GN=BMS1_PE=1_SV=1	53.264	383	164	3	306	1451	37	405	3E-118	382
BINPACKER_3622_5	sp_Q1JPX3_SYFA_DANRE_Phenylalanine--tRNA_ligase_alpha_subunit_OS=Danio_rerio_OX=7955_GN=farsa_PE=2_SV=2	42.612	291	160	5	786	1643	22	310	1.8E-148	234
NODE_20777_length_2701_cov_26.247336_g10351_i0	sp_Q2H102_BST1_CHAGB_GPI_inositol-deacylase_OS=Chaetomium_globosum_(strain_ATCC_6205_/_CBS_148.51_/_DSM_1962_/_NBRC_6347_/_NRRL_1970)_OX=30	33.75	160	102	2	1344	865	343	498	1.26E-14	82.4
NODE_116334_length_697_cov_38.048077_g67330_i0	sp_Q2MHE4_HT1_ARATH_Serine/threonine-protein_kinase_HT1_OS=Arabidopsis_thaliana_OX=3702_GN=HT1_PE=1_SV=1	83.406	229	38	0	10	696	122	350	8.1E-143	407
NODE_19703_length_2767_cov_29.785820_g9823_i1	sp_Q2QMX9_ACA10_ORYSJ_Calcium-transporting_ATPase_10,_plasma_membrane-type_OS=Oryza_sativa_subsp._japonica_OX=39947_GN=ACA10_PE=2_SV=1	93.657	804	51	0	351	2762	1	804	0	1523
BINPACKER_7486_1	sp_Q2QNU0_TR130_ORYSJ_Trafficking_protein_particle_complex_II-specific_subunit_130_homolog_OS=Oryza_sativa_subsp._japonica_OX=39947_GN=TRS130_PE=2_	91.152	712	60	2	159	2288	1	711	0	1329
BINPACKER_28145_5	sp_Q2QTC2_PWD_ORYSJ_Phosphoglucan,_water_dikinase,_chloroplastic_OS=Oryza_sativa_subsp._japonica_OX=39947_GN=GWD3_PE=3_SV=2	78.5	400	86	0	183	1382	807	1206	0	567
NODE_9547_length_3636_cov_27.134718_g4676_i2	sp_Q2R2B4_RFC1_ORYSJ_Replication_factor_C_subunit_1_OS=Oryza_sativa_subsp._japonica_OX=39947_GN=RFC1_PE=2_SV=2	88.67	812	84	3	886	3312	192	998	0	1308
BINPACKER_4945_5	sp_Q2RBN7_CLH1_ORYSJ_Clathrin_heavy_chain_1_OS=Oryza_sativa_subsp._japonica_OX=39947_GN=Os11g0104900_PE=3_SV=1	98.026	1013	20	0	196	3234	10	1022	0	2007
NODE_19622_length_2772_cov_22.017414_g1560_i5	sp_Q2RBN7_CLH1_ORYSJ_Clathrin_heavy_chain_1_OS=Oryza_sativa_subsp._japonica_OX=39947_GN=Os11g0104900_PE=3_SV=1	97.719	263	6	0	1787	2575	252	514	1.1E-149	486
BINPACKER_11299_1	sp_Q2TBM9_SRAC1_BOVIN_Protein_SERAC1_OS=Bos_taurus_OX=9913_GN=SERAC1_PE=2_SV=1	44.286	140	66	4	2704	3105	395	528	5.86E-39	117
BINPACKER_11299_3	sp_Q2TBM9_SRAC1_BOVIN_Protein_SERAC1_OS=Bos_taurus_OX=9913_GN=SERAC1_PE=2_SV=1	44.286	140	66	4	2707	3108	395	528	5.86E-39	117
BINPACKER_15835_4	sp_Q336N8_JM706_ORYSJ_Lysine-specific_demethylase_JMJ706_OS=Oryza_sativa_subsp._japonica_OX=39947_GN=JMJ706_PE=2_SV=1	49.153	826	388	10	3035	618	16	829	0	778
NODE_36144_length_2051_cov_22.107685_g9688_i1	sp_Q38933_LCYB_ARATH_Lycopene_beta_cyclase,_chloroplastic_OS=Arabidopsis_thaliana_OX=3702_GN=LCY1_PE=1_SV=1	76.43	437	102	1	285	1595	66	501	0	680
Contig7614	sp_Q3BAI2_YCX91_PHAAO_Uncharacterized_protein_ORF91_OS=Phalaenopsis_aphrodite_subsp._formosana_OX=308872_PE=4_SV=1	96.226	53	2	0	1957	1799	39	91	3.18E-27	108
BINPACKER_14_1	sp_Q3BAI2_YCX91_PHAAO_Uncharacterized_protein_ORF91_OS=Phalaenopsis_aphrodite_subsp._formosana_OX=308872_PE=4_SV=1	87.931	58	7	0	848	1021	1	58	1.58E-26	107
NODE_8622_length_3766_cov_36.254806_g2948_i1	sp_Q3BAI2_YCX91_PHAAO_Uncharacterized_protein_ORF91_OS=Phalaenopsis_aphrodite_subsp._formosana_OX=308872_PE=4_SV=1	81.69	71	11	1	1493	1287	1	71	6.44E-29	114
BINPACKER_56085_1	sp_Q3BAI2_YCX91_PHAAO_Uncharacterized_protein_ORF91_OS=Phalaenopsis_aphrodite_subsp._formosana_OX=308872_PE=4_SV=1	78.161	87	19	0	71	331	5	91	3.27E-43	139
NODE_235_length_10039_cov_29.675597_g32_i4	sp_Q41365_PRS7_SPIOL_26S_proteasome_regulatory_subunit_7_OS=Spinacia_oleracea_OX=3562_GN=RPT1_PE=2_SV=1	100	292	0	0	8758	7883	135	426	0	602
NODE_71784_length_1239_cov_29.917667_g36906_i0	sp_Q41542_XTH_WHEAT_Probable_xyloglucan_endotransglucosylase/hydrolase_OS=Triticum_aestivum_OX=4565_GN=XTH_PE=2_SV=1	89.668	271	27	1	1080	268	24	293	0	518
Contig3874	sp_Q42456_ASPR1_ORYSJ_Aspartic_proteinase_oryzasin-1_OS=Oryza_sativa_subsp._japonica_OX=39947_GN=Os05g0567100_PE=2_SV=2	88.998	509	55	1	1831	308	1	509	0	883
BINPACKER_869_4	sp_Q43697_TBB5_MAIZE_Tubulin_beta-5_chain_OS=Zea_mays_OX=4577_GN=TUBB5_PE=2_SV=1	99.534	429	2	0	4879	3593	1	429	0	872
NODE_105282_length_801_cov_29.458791_g11442_i4	sp_Q53LQ0_PDI11_ORYSJ_Protein_disulfide_isomerase-like_1-1_OS=Oryza_sativa_subsp._japonica_OX=39947_GN=PDIL1-1_PE=2_SV=1	41.346	104	56	3	588	277	405	503	5.76E-15	77.8
BINPACKER_2187_1	sp_Q54IK2_TM120_DICDI_Transmembrane_protein_120_homolog_OS=Dictyostelium_discoideum_OX=44689_GN=tmem120_PE=3_SV=1	34.241	257	140	7	1744	995	106	340	2.83E-22	102
NODE_80828_length_1096_cov_23.658847_g32405_i1	sp_Q54R62_UCKC_DICDI_Uridine-cytidine_kinase_C_OS=Dictyostelium_discoideum_OX=44689_GN=udkC_PE=3_SV=1	35.075	268	156	6	341	1096	27	292	1.87E-44	162
NODE_5029_length_4498_cov_28.343955_g2563_i0	sp_Q54T85_TRA1_DICDI_Probable_transcription-associated_protein_1_OS=Dictyostelium_discoideum_OX=44689_GN=tra1_PE=3_SV=2	36.568	845	454	16	312	2780	1645	2429	2.4E-129	450
BINPACKER_3574_3	sp_Q56YP2_PI5K1_ARATH_Phosphatidylinositol_4-phosphate_5-kinase_1_OS=Arabidopsis_thaliana_OX=3702_GN=PIP5K1_PE=1_SV=1	66.667	537	153	5	510	2105	71	586	0	711
BINPACKER_3574_6	sp_Q56YP2_PI5K1_ARATH_Phosphatidylinositol_4-phosphate_5-kinase_1_OS=Arabidopsis_thaliana_OX=3702_GN=PIP5K1_PE=1_SV=1	66.357	538	155	5	504	2102	70	586	0	710
NODE_31028_length_2231_cov_29.982391_g1851_i3	sp_Q5F3V3_PDS5A_CHICK_Sister_chromatid_cohesion_protein_PDS5_homolog_A_OS=Gallus_gallus_OX=9031_GN=PDS5A_PE=2_SV=2	28.736	174	122	2	782	1297	62	235	2.11E-17	90.9
BINPACKER_15128_2	sp_Q5HZ38_GRIK2_ARATH_Serine/threonine-protein_kinase_GRIK2_OS=Arabidopsis_thaliana_OX=3702_GN=GRIK2_PE=1_SV=1	66.5	200	62	1	806	222	65	264	9.12E-87	274
NODE_39372_length_1950_cov_23.713905_g1408_i4	sp_Q5NBT9_TPR1_ORYSJ_Protein_TPR1_OS=Oryza_sativa_subsp._japonica_OX=39947_GN=TPR1_PE=1_SV=1	94.982	279	13	1	1709	876	1	279	2.1E-171	521
BINPACKER_10190_2	sp_Q5NBT9_TPR1_ORYSJ_Protein_TPR1_OS=Oryza_sativa_subsp._japonica_OX=39947_GN=TPR1_PE=1_SV=1	78.003	741	93	4	251	2464	1	674	0	1155
BINPACKER_9064_5	sp_Q5U241_PD5BB_XENLA_Sister_chromatid_cohesion_protein_PDS5_homolog_B-B_OS=Xenopus_laevis_OX=8355_GN=pds5b-b_PE=2_SV=2	25.057	882	629	14	2577	16	68	945	3E-50	197
NODE_24015_length_2525_cov_30.757749_g12037_i0	sp_Q5VQ78_COB21_ORYSJ_Coatomer_subunit_beta'-1_OS=Oryza_sativa_subsp._japonica_OX=39947_GN=Os06g0143900_PE=2_SV=1	91.204	648	52	1	32	1960	254	901	0	1189
NODE_83902_length_1053_cov_37.536735_g44298_i0	sp_Q5VQ78_COB21_ORYSJ_Coatomer_subunit_beta'-1_OS=Oryza_sativa_subsp._japonica_OX=39947_GN=Os06g0143900_PE=2_SV=1	98.701	308	4	0	925	2	1	308	0	652
Contig4882	sp_Q5VQG4_RFS_ORYSJ_Galactinol--sucrose_galactosyltransferase_OS=Oryza_sativa_subsp._japonica_OX=39947_GN=RFS_PE=1_SV=1	82.976	793	113	9	2493	151	1	783	0	1303
NODE_42937_length_1851_cov_23.165354_g21450_i0	sp_Q5ZLW3_DYM_CHICK_Dymeclin_OS=Gallus_gallus_OX=9031_GN=DYM_PE=2_SV=1	30.019	513	312	12	1589	90	23	501	1.21E-50	189
BINPACKER_4692_2	sp_Q620W3_EFR3_CAEBR_Protein_EFR3_homolog_OS=Caenorhabditis_briggsae_OX=6238_GN=CBG02625_PE=3_SV=1	21.415	523	338	16	418	1908	6	481	2.84E-18	94.7
NODE_53142_length_1602_cov_15.655984_g26729_i0	sp_Q65X71_ACA6_ORYSJ_Probable_calcium-transporting_ATPase_6,_plasma_membrane-type_OS=Oryza_sativa_subsp._japonica_OX=39947_GN=ACA6_PE=3_SV=1	72.283	184	34	1	553	2	9	175	4.4E-68	240
BINPACKER_1614_1	sp_Q67ZB3_FRL3_ARATH_FRIGIDA-like_protein_3_OS=Arabidopsis_thaliana_OX=3702_GN=FRL3_PE=1_SV=1	51.142	569	218	11	2793	1207	1	549	1.5E-158	481
NODE_36257_length_2047_cov_28.080041_g18187_i0	sp_Q69TY5_SPP_ORYSJ_Stromal_processing_peptidase,_chloroplastic_OS=Oryza_sativa_subsp._japonica_OX=39947_GN=SPP_PE=2_SV=2	88.095	546	62	2	417	2045	42	587	0	980
Contig8714	sp_Q69UI2_RS131_ORYSJ_40S_ribosomal_protein_S13-1_OS=Oryza_sativa_subsp._japonica_OX=39947_GN=Os08g0117200_PE=3_SV=2	69.412	85	24	1	694	446	67	151	1.09E-44	119
BINPACKER_59_5	sp_Q6AT10_IAA15_ORYSJ_Auxin-responsive_protein_IAA15_OS=Oryza_sativa_subsp._japonica_OX=39947_GN=IAA15_PE=2_SV=1	63.226	155	41	3	424	2	17	169	2.94E-38	139
BINPACKER_2511_14	sp_Q6AUG0_U2AFB_ORYSJ_Splicing_factor_U2af_small_subunit_B_OS=Oryza_sativa_subsp._japonica_OX=39947_GN=U2AF35B_PE=2_SV=1	95.109	184	9	0	1076	1627	1	184	1.3E-120	375
BINPACKER_3980_1	sp_Q6AVI0_RSLE2_ORYSJ_Zinc_finger_BED_domain-containing_protein_RICESLEEPER_2_OS=Oryza_sativa_subsp._japonica_OX=39947_GN=Os03g0733400_PE=2_SV=	83.242	728	114	2	2451	268	1	720	0	1206

BINPACKER_3980_3	sp_Q6AVI0_RSLE2_ORYSJ_Zinc_finger_BED_domain-containing_protein_RICESLEEPER_2_OS=Oryza_sativa_subsp._japonica_OX=39947_GN=Os03g0733400_PE=2_SV=	82.167	729	121	3	2454	268	1	720	0	1190
Contig6666	sp_Q6CQE5_TAR1_KLULA_Protein_TAR1_OS=Kluyveromyces_lactis_(strain_ATCC_8585_/_CBS_2359_/_DSM_70799_/_NBRC_1267_/_NRRL_Y-1140_/_WM37)_OX=28	77.358	53	12	0	1677	1835	7	59	1.6E-16	79.3
Contig7942	sp_Q6CQE5_TAR1_KLULA_Protein_TAR1_OS=Kluyveromyces_lactis_(strain_ATCC_8585_/_CBS_2359_/_DSM_70799_/_NBRC_1267_/_NRRL_Y-1140_/_WM37)_OX=28	55.682	88	39	0	4102	3839	7	94	2.47E-14	73.9
NODE_158145_length_443_cov_4.624324_g104246_i0	sp_Q6CQE5_TAR1_KLULA_Protein_TAR1_OS=Kluyveromyces_lactis_(strain_ATCC_8585_/_CBS_2359_/_DSM_70799_/_NBRC_1267_/_NRRL_Y-1140_/_WM37)_OX=28	64.815	54	19	0	12	173	16	69	6.62E-11	58.9
NODE_9780_length_3603_cov_115.428329_g4942_i0	sp_Q6ENQ5_YCF68_SACOF_Uncharacterized_protein_ycf68_OS=Saccharum_officinarum_OX=4547_GN=ycf68-1_PE=3_SV=1	94.262	122	7	0	1152	1517	1	122	5.61E-44	159
NODE_21020_length_2688_cov_33.556405_g6301_i81	sp_Q6ER94_BAS1_ORYSJ_2-Cys_peroxiredoxin_BAS1,_chloroplastic_OS=Oryza_sativa_subsp._japonica_OX=39947_GN=BAS1_PE=1_SV=1	95.808	167	7	0	159	659	58	224	9.8E-107	333
BINPACKER_5641_6	sp_Q6EVK6_BRM_ARATH_ATP-dependent_helicase_BRM_OS=Arabidopsis_thaliana_OX=3702_GN=BRM_PE=1_SV=1	55.214	2148	802	43	697	6921	133	2193	0	1905
BINPACKER_7853_4	sp_Q6GQT9_NOMO1_MOUSE_Nodal_modulator_1_OS=Mus_musculus_OX=10090_GN=Nomo1_PE=1_SV=1	32.685	1080	615	34	3928	875	26	1055	4.8E-160	517
BINPACKER_21293_3	sp_Q6H795_CLPD1_ORYSJ_Chaperone_protein_ClpD1,_chloroplastic_OS=Oryza_sativa_subsp._japonica_OX=39947_GN=CLPD1_PE=2_SV=1	83.962	212	29	2	439	1074	546	752	5E-159	358
NODE_98777_length_868_cov_21.090566_g54202_i0	sp_Q6H8D5_COB22_ORYSJ_Coatomer_subunit_beta'-2_OS=Oryza_sativa_subsp._japonica_OX=39947_GN=Os02g0209100_PE=2_SV=1	96.244	213	8	0	228	866	2	214	5.2E-150	445
NODE_8877_length_3729_cov_32.091904_g4483_i0	sp_Q6K3T2_KAT1_ORYSJ_Potassium_channel_KAT1_OS=Oryza_sativa_subsp._japonica_OX=39947_GN=Os02g0245800_PE=2_SV=1	75.78	673	144	3	607	2601	57	718	0	1043
NODE_113199_length_725_cov_34.136503_g64816_i0	sp_Q6K4V2_SWET4_ORYSJ_Bidirectional_sugar_transporter_SWEET4_OS=Oryza_sativa_subsp._japonica_OX=39947_GN=SWEET4_PE=2_SV=1	88.701	177	20	0	531	1	1	177	9.64E-93	276
BINPACKER_14318_1	sp_Q6K8Z4_FH7_ORYSJ_Formin-like_protein_7_OS=Oryza_sativa_subsp._japonica_OX=39947_GN=FH7_PE=3_SV=2	70.304	559	94	6	465	2051	1	517	0	768
NODE_39723_length_1939_cov_21.869775_g19908_i0	sp_Q6L4L4_SIZ1_ORYSJ_E3_SUMO-protein_ligase_SIZ1_OS=Oryza_sativa_subsp._japonica_OX=39947_GN=SIZ1_PE=1_SV=1	80.365	219	40	2	1131	475	36	251	3.5E-120	381
BINPACKER_12252_2	sp_Q6WWW4_UPL3_ARATH_E3_ubiquitin-protein_ligase_UPL3_OS=Arabidopsis_thaliana_OX=3702_GN=UPL3_PE=1_SV=1	64.558	996	325	12	613	3570	155	1132	0	1111
BINPACKER_337_5	sp_Q6YSF3_AN321_ORYSJ_Acidic_leucine-rich_nuclear_phosphoprotein_32-related_protein_1_OS=Oryza_sativa_subsp._japonica_OX=39947_GN=Os07g0607800_PE=	85.973	221	29	1	1256	600	1	221	1.61E-93	294
BINPACKER_627_2	sp_Q6YWQ4_HAK25_ORYSJ_Potassium_transporter_25_OS=Oryza_sativa_subsp._japonica_OX=39947_GN=HAK25_PE=2_SV=1	86.089	762	99	4	471	2753	15	770	0	1259
BINPACKER_627_5	sp_Q6YWQ4_HAK25_ORYSJ_Potassium_transporter_25_OS=Oryza_sativa_subsp._japonica_OX=39947_GN=HAK25_PE=2_SV=1	85.827	762	101	4	463	2745	15	770	0	1255
NODE_145609_length_500_cov_52.327869_g92656_i0	sp_Q6YZX6_ACOC_ORYSJ_Putative_aconitate_hydratase,_cytoplasmic_OS=Oryza_sativa_subsp._japonica_OX=39947_GN=Os08g0191100_PE=3_SV=1	96.988	166	5	0	500	3	88	253	4.96E-97	302
BINPACKER_5175_2	sp_Q6Z0E2_CCC1_ORYSJ_Cation-chloride_cotransporter_1_OS=Oryza_sativa_subsp._japonica_OX=39947_GN=CCC1_PE=2_SV=1	88.542	384	28	4	1742	600	1	371	0	635
NODE_4706_length_4596_cov_23.638072_g2395_i0	sp_Q6ZFZ4_DEK1_ORYSJ_Calpain-type_cysteine_protease_ADL1_OS=Oryza_sativa_subsp._japonica_OX=39947_GN=ADL1_PE=1_SV=1	91.174	1031	89	1	4199	1113	1	1031	0	1617
Contig11043	sp_Q709C8_VP13C_HUMAN_Vacuolar_protein_sorting-associated_protein_13C_OS=Homo_sapiens_OX=9606_GN=VPS13C_PE=1_SV=1	25.494	506	327	13	410	1870	2	476	1.48E-28	131
BINPACKER_7641_1	sp_Q70PP2_SMG1_DROME_Serine/threonine-protein_kinase_Smg1_OS=Drosophila_melanogaster_OX=7227_GN=nonC_PE=1_SV=2	24.203	533	307	20	2596	4104	850	1315	2.55E-21	107
NODE_16394_length_2994_cov_32.117083_g8141_i0	sp_Q70PP2_SMG1_DROME_Serine/threonine-protein_kinase_Smg1_OS=Drosophila_melanogaster_OX=7227_GN=nonC_PE=1_SV=2	40	100	54	2	2699	2980	850	949	2.69E-08	62.4
BINPACKER_13876_1	sp_Q75W16_AROG_ORYSJ_Phospho-2-dehydro-3-deoxyheptonate_aldolase_2,_chloroplastic_OS=Oryza_sativa_subsp._japonica_OX=39947_GN=DAHPS2_PE=2_SV=1	84.579	214	30	2	286	918	23	236	5.6E-115	345
Contig9002	sp_Q7LHG5_YI31B_YEAST_Transposon_Ty3-I_Gag-Pol_polyprotein_OS=Saccharomyces_cerevisiae_(strain_ATCC_204508_/_S288c)_OX=559292_GN=TY3B-I_PE=3_SV=	34.043	705	421	10	2006	4006	599	1297	4.9E-110	391
BINPACKER_17821_3	sp_Q7TPQ3_SHPRH_MOUSE_E3_ubiquitin-protein_ligase_SHPRH_OS=Mus_musculus_OX=10090_GN=Shprh_PE=1_SV=1	36.364	484	242	9	4975	3536	649	1070	1.15E-67	258
BINPACKER_17821_3	sp_Q7TPQ3_SHPRH_MOUSE_E3_ubiquitin-protein_ligase_SHPRH_OS=Mus_musculus_OX=10090_GN=Shprh_PE=1_SV=1	38.075	239	114	4	1256	552	1458	1666	3.4E-36	155
BINPACKER_17821_3	sp_Q7TPQ3_SHPRH_MOUSE_E3_ubiquitin-protein_ligase_SHPRH_OS=Mus_musculus_OX=10090_GN=Shprh_PE=1_SV=1	36.364	132	59	5	5401	5048	267	387	1.75E-11	73.9
NODE_37534_length_2006_cov_22.611485_g18823_i0	sp_Q7XCL0_EXLA2_ORYSJ_Expansin-like_A2_OS=Oryza_sativa_subsp._japonica_OX=39947_GN=EXLA2_PE=2_SV=1	85.938	128	17	1	542	162	148	275	5.65E-70	232
BINPACKER_6475_20	sp_Q7XCL0_EXLA2_ORYSJ_Expansin-like_A2_OS=Oryza_sativa_subsp._japonica_OX=39947_GN=EXLA2_PE=2_SV=1	83.333	84	11	2	2331	2089	192	275	1.63E-37	145
NODE_42009_length_1876_cov_30.190238_g7261_i1	sp_Q7XD99_EXEC1_ORYSJ_Protein_EXECUTER_1,_chloroplastic_OS=Oryza_sativa_subsp._japonica_OX=39947_GN=EX1_PE=2_SV=1	75.605	537	114	3	280	1875	81	605	0	710
BINPACKER_14225_1	sp_Q7XHL3_TDC2_ORYSJ_Tryptophan_decarboxylase_2_OS=Oryza_sativa_subsp._japonica_OX=39947_GN=TDC2_PE=1_SV=1	88.406	483	56	0	2053	605	14	496	0	890
BINPACKER_14225_7	sp_Q7XHL3_TDC2_ORYSJ_Tryptophan_decarboxylase_2_OS=Oryza_sativa_subsp._japonica_OX=39947_GN=TDC2_PE=1_SV=1	89.195	435	47	0	2027	723	14	448	0	811
BINPACKER_7391_11	sp_Q7XJE6_MCA1_ARATH_Metacaspase-1_OS=Arabidopsis_thaliana_OX=3702_GN=AMC1_PE=1_SV=1	50.385	260	107	4	2734	1955	30	267	2.29E-62	218
Contig9474	sp_Q7XLC6_HAK11_ORYSJ_Probable_potassium_transporter_11_OS=Oryza_sativa_subsp._japonica_OX=39947_GN=HAK11_PE=2_SV=4	91.878	197	16	0	760	1350	1	197	1E-122	378
BINPACKER_1177_8	sp_Q7XPY2_PMA1_ORYSJ_Plasma_membrane_ATPase_OS=Oryza_sativa_subsp._japonica_OX=39947_GN=Os04g0656100_PE=2_SV=1	90.058	694	33	1	2281	200	1	658	0	1134
BINPACKER_11666_3	sp_Q7XQ12_AMT11_ORYSJ_Ammonium_transporter_1_member_1_OS=Oryza_sativa_subsp._japonica_OX=39947_GN=AMT1-1_PE=2_SV=1	93.621	486	31	0	1984	527	1	486	0	852
NODE_42111_length_1873_cov_27.469444_g21045_i0	sp_Q7XZU0_SAC9_ARATH_Probable_phosphoinositide_phosphatase_SAC9_OS=Arabidopsis_thaliana_OX=3702_GN=SAC9_PE=1_SV=1	68.24	466	145	3	1393	2	9	473	0	676
BINPACKER_1516_15	sp_Q7ZWL6_MAF1_XENLA_Repressor_of_RNA_polymerase_III_transcription_MAF1_homolog_OS=Xenopus_laevis_OX=8355_GN=maf1_PE=2_SV=1	47.959	98	46	1	2293	2000	110	202	1.19E-18	90.1
BINPACKER_13620_1	sp_Q84LK0_MSH1_ARATH_DNA_mismatch_repair_protein_MSH1,_mitochondrial_OS=Arabidopsis_thaliana_OX=3702_GN=MSH1_PE=1_SV=1	61.124	872	316	6	2996	390	255	1106	0	1077
Contig4276	sp_Q84WU2_UBP13_ARATH_Ubiquitin_carboxyl-terminal_hydrolase_13_OS=Arabidopsis_thaliana_OX=3702_GN=UBP13_PE=1_SV=1	72.099	362	99	2	2855	1773	651	1011	4.5E-157	511
NODE_39113_length_1957_cov_27.431529_g17930_i2	sp_Q84WU2_UBP13_ARATH_Ubiquitin_carboxyl-terminal_hydrolase_13_OS=Arabidopsis_thaliana_OX=3702_GN=UBP13_PE=1_SV=1	37.037	135	74	3	789	385	55	178	8.1E-16	85.5
BINPACKER_12996_3	sp_Q84ZL0_FH5_ORYSJ_Formin-like_protein_5_OS=Oryza_sativa_subsp._japonica_OX=39947_GN=FH5_PE=2_SV=2	75.676	814	181	5	266	2662	1	812	0	1230
NODE_74820_length_1189_cov_20.601254_g19063_i3	sp_Q8BHB4_WDR3_MOUSE_WD_repeat-containing_protein_3_OS=Mus_musculus_OX=10090_GN=Wdr3_PE=1_SV=1	23.871	155	107	1	24	455	463	617	5.43E-06	52.4
Contig11882	sp_Q8GY23_UPL1_ARATH_E3_ubiquitin-protein_ligase_UPL1_OS=Arabidopsis_thaliana_OX=3702_GN=UPL1_PE=1_SV=3	37.527	922	506	17	6526	3839	9	886	2.5E-136	483
NODE_2057_length_5796_cov_30.540800_g1080_i0	sp_Q8GY23_UPL1_ARATH_E3_ubiquitin-protein_ligase_UPL1_OS=Arabidopsis_thaliana_OX=3702_GN=UPL1_PE=1_SV=3	35.721	1089	599	24	3688	578	990	2029	1.1E-163	563
NODE_2130_length_5751_cov_29.685981_g1105_i0	sp_Q8GY23_UPL1_ARATH_E3_ubiquitin-protein_ligase_UPL1_OS=Arabidopsis_thaliana_OX=3702_GN=UPL1_PE=1_SV=3	35.842	1063	574	24	3637	638	992	2009	6.2E-158	545
NODE_32616_length_2174_cov_24.599238_g2618_i2	sp_Q8H3P9_HAK7_ORYSJ_Potassium_transporter_7_OS=Oryza_sativa_subsp._japonica_OX=39947_GN=HAK7_PE=2_SV=3	85.753	365	49	3	644	1738	450	811	0	600
BINPACKER_11212_2	sp_Q8L7S3_C3H24_ARATH_Zinc_finger_CCCH_domain-containing_protein_24_OS=Arabidopsis_thaliana_OX=3702_GN=At2g28450_PE=1_SV=1	48.87	841	292	12	233	2668	78	809	0	752
NODE_67921_length_1307_cov_35.444084_g30860_i1	sp_Q8LGG8_USPAL_ARATH_Universal_stress_protein_A-like_protein_OS=Arabidopsis_thaliana_OX=3702_GN=At3g01520_PE=1_SV=2	47.727	44	23	0	766	897	104	147	4.8E-06	50.4
Contig10910	sp_Q8LLD0_NUP96_ARATH_Nuclear_pore_complex_protein_NUP96_OS=Arabidopsis_thaliana_OX=3702_GN=NUP96_PE=1_SV=1	53.808	1011	430	9	542	3526	46	1035	0	1062
NODE_22129_length_2626_cov_26.583627_g11047_i0	sp_Q8LPQ5_BTS_ARATH_Zinc_finger_protein_BRUTUS_OS=Arabidopsis_thaliana_OX=3702_GN=BTS_PE=1_SV=1	50.428	817	374	12	249	2624	1	811	0	738
BINPACKER_8732_6	sp_Q8N766_EMC1_HUMAN_ER_membrane_protein_complex_subunit_1_OS=Homo_sapiens_OX=9606_GN=EMC1_PE=1_SV=1	28.63	971	578	30	3392	666	22	939	8.83E-91	318
NODE_64849_length_1362_cov_37.215671_g2133_i1	sp_Q8R3Q2_PP6R2_MOUSE_Serine/threonine-protein_phosphatase_6_regulatory_subunit_2_OS=Mus_musculus_OX=10090_GN=Ppp6r2_PE=1_SV=1	37.056	197	122	2	249	839	1	195	2.25E-30	128
NODE_6291_length_4175_cov_27.213067_g3186_i0	sp_Q8RVL1_DEK1_MAIZE_Calpain-type_cysteine_protease_DEK1_OS=Zea_mays_OX=4577_GN=DEK1_PE=1_SV=2	88.739	1039	82	1	3783	667	1	1004	0	1601
BINPACKER_5433_10	sp_Q8RWW0_ALE2_ARATH_Receptor-like_serine/threonine-protein_kinase_ALE2_OS=Arabidopsis_thaliana_OX=3702_GN=ALE2_PE=1_SV=1	78.723	188	39	1	3877	4440	439	625	2.83E-87	306
BINPACKER_3965_6	sp_Q8RWY6_CLASP_ARATH_CLIP-associated_protein_OS=Arabidopsis_thaliana_OX=3702_GN=CLASP_PE=1_SV=1	60.475	1222	458	12	178	3786	1	1216	0	1341
NODE_43689_length_1831_cov_28.917520_g4080_i2	sp_Q8RX56_UNC13_ARATH_Protein_unc-13_homolog_OS=Arabidopsis_thaliana_OX=3702_GN=PATROL1_PE=2_SV=1	33.333	486	287	12	1492	104	260	731	2.57E-61	223
NODE_77447_length_1147_cov_21.022346_g21017_i2	sp_Q8RY89_PI5K8_ARATH_Phosphatidylinositol_4-phosphate_5-kinase_8_OS=Arabidopsis_thaliana_OX=3702_GN=PIP5K8_PE=1_SV=1	34.066	182	97	1	548	3	3	161	2.09E-19	93.6
BINPACKER_1935_8	sp_Q8RY89_PI5K8_ARATH_Phosphatidylinositol_4-phosphate_5-kinase_8_OS=Arabidopsis_thaliana_OX=3702_GN=PIP5K8_PE=1_SV=1	33.88	183	98	1	764	1312	3	162	4.21E-18	92.8
BINPACKER_495_1	sp_Q8S9J0_TAUE4_ARATH_Sulfite_exporter_TauE/SafE_family_protein_4_OS=Arabidopsis_thaliana_OX=3702_GN=At2g36630_PE=2_SV=1	52.083	336	115	4	454	1449	30	323	9.36E-93	310
NODE_56107_length_1539_cov_9.309686_g28292_i0	sp_Q8TGM5_ART3_YEAST_Putative_uncharacterized_protein_ART3_OS=Saccharomyces_cerevisiae_(strain_ATCC_204508_/_S288c)_OX=559292_GN=ART3_PE=5_SV=	72.973	37	10	0	389	279	10	46	3E-09	56.6
NODE_8056_length_3857_cov_237.693446_g4058_i0	sp_Q8TGM7_ART2_YEAST_Putative_uncharacterized_protein_ART2_OS=Saccharomyces_cerevisiae_(strain_ATCC_204508_/_S288c)_OX=559292_GN=ART2_PE=5_SV=	76.786	56	13	0	2532	2699	2	57	1.51E-17	81.3
BINPACKER_11327_4	sp_Q8VCY6_UTP6_MOUSE_U3_small_nucleolar_RNA-associated_protein_6_homolog_OS=Mus_musculus_OX=10090_GN=Utp6_PE=2_SV=1	25.743	404	223	11	2372	1188	1	336	4.87E-24	111
NODE_86346_length_1020_cov_31.897571_g22918_i1	sp_Q8VYD6_PP374_ARATH_Pentatricopeptide_repeat-containing_protein_At5g10690_OS=Arabidopsis_thaliana_OX=3702_GN=CBSPPR1_PE=2_SV=1	58.525	217	83	2	284	928	41	252	3.3E-70	232
BINPACKER_2253_1	sp_Q8VZG7_TSN1_ARATH_Ribonuclease_TUDOR_1_OS=Arabidopsis_thaliana_OX=3702_GN=TSN1_PE=1_SV=1	66.388	717	222	11	2637	520	3	711	0	924
BINPACKER_6301_1	sp_Q8VZT0_NLAL1_ARATH_Putative_H/ACA_ribonucleoprotein_complex_subunit_1-like_protein_1_OS=Arabidopsis_thaliana_OX=3702_GN=At3g03920_PE=2_SV=1	84.158	101	16	0	667	365	51	151	2.88E-56	184
Contig6487	sp_Q8W1L6_MFP_ORYSJ_Peroxisomal_fatty_acid_beta-oxidation_multifunctional_protein_OS=Oryza_sativa_subsp._japonica_OX=39947_GN=MFP_PE=1_SV=2	94.048	84	5	0	256	5	120	203	2.52E-85	165
NODE_53485_length_1594_cov_23.708087_g6470_i1	sp_Q8W1Y0_SSC14_ARATH_Sister_chromatid_cohesion_1_protein_4_OS=Arabidopsis_thaliana_OX=3702_GN=SYN4_PE=2_SV=1	61.111	54	21	0	1057	896	574	627	7.26E-13	75.5
BINPACKER_11104_5	sp_Q93VQ0_VCL1_ARATH_Protein_VACUOLELESS1_OS=Arabidopsis_thaliana_OX=3702_GN=VCL1_PE=1_SV=1	53.247	539	210	8	257	1819	8	522	1.7E-170	513
BINPACKER_8843_3	sp_Q93YS6_GTE9_ARATH_Transcription_factor_GTE9_OS=Arabidopsis_thaliana_OX=3702_GN=GTE9_PE=1_SV=1	38.314	783	354	24	496	2760	7	688	7.17E-84	290
NODE_12690_length_3303_cov_21.344582_g6352_i1	sp_Q93YU8_NRG2_ARATH_Nitrate_regulatory_gene2_protein_OS=Arabidopsis_thaliana_OX=3702_GN=NRG2_PE=1_SV=1	32.639	144	81	4	931	548	649	792	4.85E-13	77.4
BINPACKER_2461_5	sp_Q93YV6_2A5K_ARATH_Serine/threonine_protein_phosphatase_2A_57_kDa_regulatory_subunit_B'_kappa_isoform_OS=Arabidopsis_thaliana_OX=3702_GN=B'KAP	60.817	416	162	1	1526	279	85	499	0	537
Contig3130	sp_Q941T1_P5CS2_ORYSJ_Delta-1-pyrroline-5-carboxylate_synthase_2_OS=Oryza_sativa_subsp._japonica_OX=39947_GN=P5CS2_PE=2_SV=1	86.966	468	61	0	324	1727	19	486	0	771
BINPACKER_13766_3	sp_Q941T1_P5CS2_ORYSJ_Delta-1-pyrroline-5-carboxylate_synthase_2_OS=Oryza_sativa_subsp._japonica_OX=39947_GN=P5CS2_PE=2_SV=1	86.538	468	63	0	297	1700	19	486	0	769
NODE_71321_length_1247_cov_24.484668_g33464_i1	sp_Q94AH3_NIPA4_ARATH_Probable_magnesium_transporter_NIPA4_OS=Arabidopsis_thaliana_OX=3702_GN=At1g71900_PE=2_SV=1	81.013	237	45	0	537	1247	6	242	2.3E-86	269
BINPACKER_11333_7	sp_Q94CC0_Y5994_ARATH_Uncharacterized_protein_At5g49945_OS=Arabidopsis_thaliana_OX=3702_GN=At5g49945_PE=2_SV=1	56.79	243	103	2	378	1103	96	337	1.43E-81	263
BINPACKER_20082_3	sp_Q94FB9_AB1D_ARATH_ABC_transporter_D_family_member_1_OS=Arabidopsis_thaliana_OX=3702_GN=ABCD1_PE=1_SV=1	68.052	385	71	2	1000	2	453	837	1.8E-160	501
NODE_27886_length_2349_cov_50.690685_g13995_i0	sp_Q94K49_ALP1_ARATH_Protein_ANTAGONIST_OF_LIKE_HETEROCHROMATIN_PROTEIN_1_OS=Arabidopsis_thaliana_OX=3702_GN=ALP1_PE=1_SV=1	30.183	328	186	9	1857	964	43	357	1.71E-36	145
NODE_35179_length_2083_cov_30.670149_g17626_i0	sp_Q94K49_ALP1_ARATH_Protein_ANTAGONIST_OF_LIKE_HETEROCHROMATIN_PROTEIN_1_OS=Arabidopsis_thaliana_OX=3702_GN=ALP1_PE=1_SV=1	28.613	346	204	9	258	1205	41	373	2.66E-33	135
BINPACKER_7553_7	sp_Q9ASS2_FREE1_ARATH_Protein_FREE1_OS=Arabidopsis_thaliana_OX=3702_GN=FREE1_PE=1_SV=1	67.898	352	99	6	1719	673	261	601	1.2E-150	456
BINPACKER_7553_1	sp_Q9ASS2_FREE1_ARATH_Protein_FREE1_OS=Arabidopsis_thaliana_OX=3702_GN=FREE1_PE=1_SV=1	68.085	376	104	7	1788	673	238	601	1.6E-159	479
NODE_89823_length_975_cov_29.166297_g48136_i0	sp_Q9AWL7_GIGAN_ORYSJ_Protein_GIGANTEA_OS=Oryza_sativa_subsp._japonica_OX=39947_GN=GI_PE=2_SV=2	88.372	215	25	0	922	278	1	215	4.7E-124	385
NODE_57469_length_1510_cov_29.632568_g28978_i0	sp_Q9AWS0_Y1410_ORYSJ_AP2/ERF_and_B3_domain-containing_protein_Os01g0141000_OS=Oryza_sativa_subsp._japonica_OX=39947_GN=Os01g0141000_PE=2_SV	78.193	321	57	4	354	1283	47	365	8.6E-122	364
NODE_53686_length_1590_cov_17.008570_g18273_i2	sp_Q9C8G9_AB1C_ARATH_ABC_transporter_C_family_member_1_OS=Arabidopsis_thaliana_OX=3702_GN=ABCC1_PE=1_SV=1	51.452	241	110	4	1095	379	1	236	6.73E-69	244
BINPACKER_15274_4	sp_Q9FF80_SUVH1_ARATH_Histone-lysine_N-methyltransferase,_H3_lysine-9_specific_SUVH1_OS=Arabidopsis_thaliana_OX=3702_GN=SUVH1_PE=2_SV=1	52.747	546	232	11	2007	418	135	670	0	564
NODE_83497_length_1059_cov_32.326572_g44030_i0	sp_Q9FF86_DCR_ARATH_BAHD_acyltransferase_DCR_OS=Arabidopsis_thaliana_OX=3702_GN=DCR_PE=2_SV=1	35.312	337	187	8	64	1041	132	448	3.99E-49	174
NODE_44498_length_1808_cov_28.997695_g22281_i0	sp_Q9FJ45_GDL83_ARATH_GDSL_esterase/lipase_At5g45910_OS=Arabidopsis_thaliana_OX=3702_GN=At5g45910_PE=2_SV=1	41.86	344	193	3	308	1318	27	370	5.31E-90	286
NODE_131582_length_581_cov_40.011811_g80282_i0	sp_Q9FPT1_UBP12_ARATH_Ubiquitin_carboxyl-terminal_hydrolase_12_OS=Arabidopsis_thaliana_OX=3702_GN=UBP12_PE=1_SV=2	90.155	193	19	0	581	3	124	316	5.7E-123	376
BINPACKER_9271_1	sp_Q9FPT1_UBP12_ARATH_Ubiquitin_carboxyl-terminal_hydrolase_12_OS=Arabidopsis_thaliana_OX=3702_GN=UBP12_PE=1_SV=2	82.222	270	47	1	1087	281	29	298	0	477
BINPACKER_13772_2	sp_Q9FXI5_IQD32_ARATH_Protein_IQ-DOMAIN_32_OS=Arabidopsis_thaliana_OX=3702_GN=IQD32_PE=1_SV=3	32.012	656	328	26	934	2778	215	793	4.37E-60	224
Contig2101	sp_Q9LKW9_NHX7_ARATH_Sodium/hydrogen_exchanger_7_OS=Arabidopsis_thaliana_OX=3702_GN=NHX7_PE=1_SV=1	79.859	283	57	0	799	1647	33	315	3.5E-118	392
NODE_26369_length_2414_cov_29.865869_g5216_i1	sp_Q9LMI0_TPS7_ARATH_Probable_alpha,alpha-trehalose-phosphate_synthase_[UDP-forming]_7_OS=Arabidopsis_thaliana_OX=3702_GN=TPS7_PE=1_SV=1	67.208	677	206	3	2177	147	1	661	0	969
NODE_48763_length_1705_cov_30.152574_g24438_i0	sp_Q9LNC5_CLO_ARATH_110_kDa_U5_small_nuclear_ribonucleoprotein_component_CLO_OS=Arabidopsis_thaliana_OX=3702_GN=CLO_PE=1_SV=1	75	460	113	1	1381	2	64	521	0	699
Contig3654	sp_Q9LPL4_SKP2A_ARATH_F-box_protein_SKP2A_OS=Arabidopsis_thaliana_OX=3702_GN=SKP2A_PE=1_SV=1	70.552	163	48	0	2145	1657	74	236	1.09E-64	228
NODE_2063_length_5793_cov_25.525699_g456_i2	sp_Q9LUM0_FAB1B_ARATH_1-phosphatidylinositol-3-phosphate_5-kinase_FAB1B_OS=Arabidopsis_thaliana_OX=3702_GN=FAB1B_PE=2_SV=1	51.746	1575	615	38	4675	155	205	1702	0	1399
NODE_80068_length_1107_cov_34.284333_g41882_i0	sp_Q9LV60_CRR55_ARATH_Cysteine-rich_repeat_secretory_protein_55_OS=Arabidopsis_thaliana_OX=3702_GN=CRRSP55_PE=2_SV=1	45.887	231	120	3	120	797	20	250	5.18E-51	174
NODE_55018_length_1561_cov_27.586022_g27740_i0	sp_Q9LYS2_AB10C_ARATH_ABC_transporter_C_family_member_10_OS=Arabidopsis_thaliana_OX=3702_GN=ABCC10_PE=2_SV=2	63.706	394	132	3	6	1181	659	1043	3.7E-161	498
NODE_41225_length_1897_cov_30.594298_g12887_i1	sp_Q9LZA6_SDP1_ARATH_Triacylglycerol_lipase_SDP1_OS=Arabidopsis_thaliana_OX=3702_GN=SDP1_PE=1_SV=1	72.421	504	123	7	411	1895	1	497	0	679
NODE_89116_length_984_cov_31.419319_g47672_i0	sp_Q9LZF6_CD48E_ARATH_Cell_division_control_protein_48_homolog_E_OS=Arabidopsis_thaliana_OX=3702_GN=CDC48E_PE=2_SV=2	92.657	286	21	0	859	2	4	289	0	556
BINPACKER_10187_2	sp_Q9LZI2_UXS2_ARATH_UDP-glucuronic_acid_decarboxylase_2_OS=Arabidopsis_thaliana_OX=3702_GN=UXS2_PE=1_SV=1	91.111	180	16	0	1	540	252	431	2E-115	350
NODE_71726_length_1240_cov_26.986290_g7216_i3	sp_Q9M0E5_VPS15_ARATH_Serine/threonine-protein_kinase_VPS15_OS=Arabidopsis_thaliana_OX=3702_GN=VPS15_PE=1_SV=1	71.47	347	98	1	179	1219	1	346	3.1E-161	494
BINPACKER_4614_6	sp_Q9M0E5_VPS15_ARATH_Serine/threonine-protein_kinase_VPS15_OS=Arabidopsis_thaliana_OX=3702_GN=VPS15_PE=1_SV=1	52.987	1557	581	27	5741	1335	1	1494	0	1475
Contig8	sp_Q9M658_MOM1_ARATH_Helicase_protein_MOM1_OS=Arabidopsis_thaliana_OX=3702_GN=MOM1_PE=1_SV=1	35.284	564	308	12	3070	4641	460	1006	1.4E-74	281
BINPACKER_18532_2	sp_Q9M876_SYYM_ARATH_Tyrosine--tRNA_ligase,_chloroplastic/mitochondrial_OS=Arabidopsis_thaliana_OX=3702_GN=EMB2768_PE=2_SV=1	64.924	459	114	4	1612	290	82	511	0	575
NODE_69162_length_1285_cov_22.133663_g35349_i0	sp_Q9MBC2_MMT1_HORVU_Methionine_S-methyltransferase_OS=Hordeum_vulgare_OX=4513_GN=MMT1_PE=1_SV=1	75.918	245	52	1	209	943	11	248	6E-120	377
BINPACKER_4117_8	sp_Q9S834_CLPP5_ARATH_ATP-dependent_Clp_protease_proteolytic_subunit_5,_chloroplastic_OS=Arabidopsis_thaliana_OX=3702_GN=CLPP5_PE=1_SV=1	73.741	278	57	5	180	974	24	298	1.1E-141	410
BINPACKER_7879_6	sp_Q9SDQ3_SCL1_ARATH_Scarecrow-like_protein_1_OS=Arabidopsis_thaliana_OX=3702_GN=SCL1_PE=2_SV=1	63.536	181	66	0	1134	1676	223	403	1.6E-72	245
NODE_29650_length_2282_cov_33.137619_g14886_i0	sp_Q9SF47_PAH1_ARATH_Phosphatidate_phosphatase_PAH1_OS=Arabidopsis_thaliana_OX=3702_GN=PAH1_PE=1_SV=1	47.012	251	103	6	2179	1505	1	247	2.46E-54	205
BINPACKER_23437_2	sp_Q9SFV2_FHA2_ARATH_FHA_domain-containing_protein_FHA2_OS=Arabidopsis_thaliana_OX=3702_GN=FHA2_PE=1_SV=1	46.667	345	152	7	1435	419	2	320	1.66E-79	266
BINPACKER_833_3	sp_Q9SIV5_C3H19_ARATH_Zinc_finger_CCCH_domain-containing_protein_19_OS=Arabidopsis_thaliana_OX=3702_GN=NERD_PE=1_SV=3	50.635	551	236	8	2887	4521	598	1118	2.3E-160	548
NODE_39365_length_1950_cov_26.508791_g6383_i2	sp_Q9SIW1_BLH7_ARATH_BEL1-like_homeodomain_protein_7_OS=Arabidopsis_thaliana_OX=3702_GN=BLH7_PE=1_SV=1	69.737	152	45	1	1701	1249	181	332	3.21E-66	228
BINPACKER_8504_1	sp_Q9SK66_NDUA9_ARATH_NADH_dehydrogenase_[ubiquinone]_1_alpha_subcomplex_subunit_9,_mitochondrial_OS=Arabidopsis_thaliana_OX=3702_GN=At2g2036	75.272	368	88	1	1406	303	35	399	0	573
NODE_58754_length_1483_cov_24.571631_g26965_i2	sp_Q9SLX0_IMA1B_ORYSJ_Importin_subunit_alpha-1b_OS=Oryza_sativa_subsp._japonica_OX=39947_GN=Os05g0155601_PE=1_SV=2	87.634	372	43	1	1438	332	1	372	0	652
BINPACKER_1807_8	sp_Q9SMV6_XPO1A_ARATH_Protein_EXPORTIN_1A_OS=Arabidopsis_thaliana_OX=3702_GN=XPO1_PE=1_SV=1	83.83	235	38	0	300	1004	4	238	9.4E-130	416
Contig9624	sp_Q9SMZ4_AASS_ARATH_Alpha-aminoadipic_semialdehyde_synthase_OS=Arabidopsis_thaliana_OX=3702_GN=LKR/SDH_PE=1_SV=1	80.952	84	15	1	1334	1083	12	94	2.24E-70	150
NODE_44104_length_1819_cov_26.987973_g8997_i5	sp_Q9SND9_Y3028_ARATH_Uncharacterized_acetyltransferase_At3g50280_OS=Arabidopsis_thaliana_OX=3702_GN=At3g50280_PE=3_SV=1	33.833	467	258	17	251	1588	4	440	6.27E-52	187
BINPACKER_20230_2	sp_Q9STN3_SPT51_ARATH_Putative_transcription_elongation_factor_SPT5_homolog_1_OS=Arabidopsis_thaliana_OX=3702_GN=At4g08350_PE=1_SV=2	72.021	193	47	3	569	3	113	302	6.81E-65	224
NODE_11190_length_3455_cov_31.498817_g5600_i0	sp_Q9SZL8_FRS5_ARATH_Protein_FAR1-RELATED_SEQUENCE_5_OS=Arabidopsis_thaliana_OX=3702_GN=FRS5_PE=1_SV=1	43.451	649	339	5	2837	906	71	696	3.7E-162	504
NODE_12371_length_3334_cov_31.317694_g5600_i1	sp_Q9SZL8_FRS5_ARATH_Protein_FAR1-RELATED_SEQUENCE_5_OS=Arabidopsis_thaliana_OX=3702_GN=FRS5_PE=1_SV=1	43.451	649	339	5	2716	785	71	696	2.1E-162	503
BINPACKER_2716_8	sp_Q9TIC1_MATK_PHRAU_Maturase_K_OS=Phragmites_australis_OX=29695_GN=matK_PE=3_SV=1	99.61	513	2	0	3180	1642	1	513	0	1048
NODE_60280_length_1450_cov_27.898330_g19063_i2	sp_Q9UNX4_WDR3_HUMAN_WD_repeat-containing_protein_3_OS=Homo_sapiens_OX=9606_GN=WDR3_PE=1_SV=1	23.871	155	107	1	24	455	464	618	8.1E-06	52.4
Contig10215	sp_Q9UXR9_DNAJ_METTE_Chaperone_protein_DnaJ_OS=Methanosarcina_thermophila_OX=2210_GN=dnaJ_PE=3_SV=1	55.556	72	30	1	654	869	6	75	1.76E-14	80.5
BINPACKER_3203_11	sp_Q9ZPS7_TMN3_ARATH_Transmembrane_9_superfamily_member_3_OS=Arabidopsis_thaliana_OX=3702_GN=TMN3_PE=2_SV=1	85.211	568	84	0	2027	324	25	592	0	904
BINPACKER_24495_3	sp_Q9ZQ31_STY13_ARATH_Serine/threonine-protein_kinase_STY13_OS=Arabidopsis_thaliana_OX=3702_GN=STY13_PE=1_SV=2	44.751	181	99	1	1441	899	209	388	4.68E-52	184
