# Supplementary material for: De novo transcriptome assembly and analysis of Phragmites karka, an invasive halophyte, to study the mechanism of salinity stress tolerance
Source: Sci Rep. 2020 Mar 23;10:5192. doi: 10.1038/s41598-020-61857-8 (PMC7089983; doi:10.1038/s41598-020-61857-8)
Supplement: Supplementary file 3 — Supporting Information3. [file 41598_2020_61857_MOESM3_ESM.pdf]

TableS2a. In silico expression data for DEGs in root tissue of *P. karka* during exposure to salinity stress

DEGs in root tissue under salinity stress: The data below corresponds to the differential expression of unigenes in P. karka leaves subjected to salinity stress. LogFC denotes the level of expression in sample A as compared to sample B

	sampleA	sampleB	logFC	logCPM	PValue	FDR
NODE_46908_length_1749_cov_17.919451_g23527_i0	Control	Salt_treated	9.967014746	3.077603475	1.17758E-15	1.42244E-12
NODE_26900_length_2392_cov_27.932730_g9198_i1	Control	Salt_treated	9.788423502	2.915068837	6.48365E-15	7.39676E-12
BINPACKER_2191_3	Control	Salt_treated	9.442731075	2.597851645	1.64354E-09	4.96326E-07
BINPACKER_584_15	Control	Salt_treated	9.404822814	2.568288843	3.77975E-13	3.37466E-10
BINPACKER_7519_3	Control	Salt_treated	9.308887531	2.481537437	5.32577E-12	3.52789E-09
BINPACKER_14303_1	Control	Salt_treated	9.302790581	2.480054109	1.04619E-12	7.95683E-10
BINPACKER_11352_4	Control	Salt_treated	9.220281577	2.399847912	0.000000001	3.49219E-07
BINPACKER_25624_2	Control	Salt_treated	9.085876481	2.283431235	1.96029E-11	1.15013E-08
BINPACKER_1841_16	Control	Salt_treated	8.960294733	2.1699236	1.28637E-09	4.12742E-07
BINPACKER_11034_1	Control	Salt_treated	8.927979211	2.147386065	3.56327E-11	1.82929E-08
NODE_22954_length_2581_cov_18.747209_g11467_i0	Control	Salt_treated	8.870683991	2.10411766	8.87383E-08	1.59846E-05
BINPACKER_23604_2	Control	Salt_treated	8.788068836	2.016193098	3.55691E-08	7.45318E-06
NODE_7349_length_3974_cov_15.034863_g3367_i1	Control	Salt_treated	8.768065173	1.998764521	3.24088E-08	7.00541E-06
NODE_88857_length_988_cov_5.486339_g47499_i0	Control	Salt_treated	8.748287278	1.989440773	2.37185E-10	9.0196E-08
BINPACKER_5619_12	Control	Salt_treated	8.64782689	1.904199732	1.14642E-08	3.0168E-06
BINPACKER_3082_1	Control	Salt_treated	8.584698136	1.844671809	5.41761E-10	1.98662E-07
BINPACKER_1138_6	Control	Salt_treated	8.563487471	1.836762083	1.16692E-06	0.00013693
BINPACKER_39419_2	Control	Salt_treated	8.525779012	1.791881621	1.31474E-09	4.15357E-07
BINPACKER_5851_2	Control	Salt_treated	8.449428676	1.724414035	6.13542E-09	1.68915E-06
NODE_56108_length_1539_cov_9.210778_g28293_i0	Control	Salt_treated	8.24918665	1.549955023	7.68983E-08	1.42262E-05
BINPACKER_8747_1	Control	Salt_treated	8.225765215	1.53522529	4.15535E-08	0.000008533
Contig9283	Control	Salt_treated	8.116997814	5.054394268	3.9369E-19	1.61689E-15
BINPACKER_2461_5	Control	Salt_treated	8.046461497	1.369844844	2.65385E-06	0.000262004
BINPACKER_22535_1	Control	Salt_treated	8.033469518	1.362785864	2.35227E-07	3.52582E-05
NODE_80300_length_1104_cov_19.317168_g29241_i3	Control	Salt_treated	8.028053324	1.36375345	1.20134E-07	0.000020558
NODE_2758_length_5351_cov_32.404320_g1400_i0	Control	Salt_treated	8.012934781	1.350659905	1.46154E-07	2.37666E-05
NODE_79618_length_1114_cov_18.319885_g41603_i0	Control	Salt_treated	7.990959095	1.333356074	2.00428E-07	3.09458E-05
BINPACKER_2296_5	Control	Salt_treated	7.989250492	1.332336703	2.90418E-07	4.19981E-05
NODE_29978_length_2271_cov_26.885805_g15050_i1	Control	Salt_treated	7.978850397	1.322014499	1.88076E-07	2.92586E-05
BINPACKER_13772_2	Control	Salt_treated	7.732704069	1.107063107	2.09355E-06	0.000218651
NODE_84398_length_1046_cov_30.177801_g12630_i2	Control	Salt_treated	7.728304377	1.11605474	3.59344E-06	0.000330903
NODE_47476_length_1735_cov_30.148014_g23807_i0	Control	Salt_treated	7.65509119	1.052805474	2.25733E-06	0.000231771
BINPACKER_4386_3	Control	Salt_treated	7.640091372	1.038531482	1.26511E-06	0.00014595
NODE_3214_length_5115_cov_36.923840_g0_i32	Control	Salt_treated	7.628478768	1.032689581	7.36441E-06	0.000592458
BINPACKER_16487_2	Control	Salt_treated	7.534339799	0.957874341	5.36524E-05	0.002861693
NODE_9712_length_3615_cov_22.990966_g4909_i0	Control	Salt_treated	7.49186268	0.907404078	3.80077E-06	0.000346883
NODE_11039_length_3470_cov_28.317044_g5534_i0	Control	Salt_treated	7.473474194	0.897455176	1.52141E-06	0.000168877
BINPACKER_2440_4	Control	Salt_treated	7.43324589	0.854302895	3.20835E-05	0.001937749
Contig1468	Control	Salt_treated	7.426169382	0.858356771	2.57748E-06	0.000256934
BINPACKER_34152_1	Control	Salt_treated	7.414845243	0.845380282	3.21031E-06	0.000306622
BINPACKER_7850_20	Control	Salt_treated	7.346679674	0.786976408	0.000011108	0.000811755
Contig645	Control	Salt_treated	7.3388603	0.783050517	0.00000491	0.000432736
BINPACKER_2914_2	Control	Salt_treated	7.336384872	0.781741625	4.16719E-06	0.000376975
BINPACKER_9160_1	Control	Salt_treated	7.324121665	0.775195261	0.000005585	0.00047987
NODE_19709_length_2767_cov_25.770973_g799_i2	Control	Salt_treated	7.314320355	0.762487649	5.43485E-06	0.000468927
NODE_88501_length_992_cov_23.681175_g47272_i0	Control	Salt_treated	7.274198242	0.733594297	5.91606E-06	0.000497924
Contig2960	Control	Salt_treated	7.253318737	0.705520999	8.58374E-05	0.003972195
Contig183	Control	Salt_treated	7.252768179	0.734734383	0.002063449	0.035939433
BINPACKER_16981_1	Control	Salt_treated	7.251088799	0.713621958	6.34716E-06	0.000523661
BINPACKER_9981_2	Control	Salt_treated	7.234538073	0.705552061	3.92727E-05	0.002252695
NODE_28600_length_2321_cov_27.559164_g8179_i4	Control	Salt_treated	7.230072217	0.694670764	6.76008E-06	0.000553061
BINPACKER_13653_2	Control	Salt_treated	7.134195353	0.630908776	0.000437956	0.012684662
NODE_24787_length_2486_cov_27.701616_g12421_i0	Control	Salt_treated	7.078266552	0.573516531	1.88019E-05	0.001291292
NODE_56702_length_1526_cov_29.034412_g28586_i1	Control	Salt_treated	7.075466645	0.572094617	2.17438E-05	0.001440352
BINPACKER_23749_3	Control	Salt_treated	7.063266084	0.556546279	4.11733E-05	0.002329186
BINPACKER_40706_1	Control	Salt_treated	7.063011142	0.566406529	0.000076186	0.003655324
BINPACKER_795_3	Control	Salt_treated	7.057165772	0.553675269	2.31977E-05	0.001498
Contig4362	Control	Salt_treated	7.052350736	0.56214005	0.000314456	0.009995906
BINPACKER_5750_9	Control	Salt_treated	7.052350736	0.56214005	0.000314456	0.009995906
BINPACKER_6459_6	Control	Salt_treated	7.033578489	0.532006256	6.49034E-05	0.003249915
NODE_25430_length_2456_cov_30.826269_g12763_i0	Control	Salt_treated	7.012846328	0.521858489	2.91996E-05	0.001800645
BINPACKER_2980_1	Control	Salt_treated	7.010467649	0.509915323	0.00017558	0.006718551
NODE_78502_length_1131_cov_27.205104_g40952_i0	Control	Salt_treated	7.003304981	0.506987334	7.99523E-05	0.00378168
NODE_20942_length_2692_cov_27.568156_g9710_i1	Control	Salt_treated	6.972445913	0.4923465	8.32594E-05	0.003908728
Contig11157	Control	Salt_treated	6.972423385	0.481470477	0.000128917	0.005413716
BINPACKER_13552_1	Control	Salt_treated	6.940903251	0.455435497	0.00020786	0.007409526
NODE_41340_length_1895_cov_17.052141_g20661_i0	Control	Salt_treated	6.884224271	0.404774291	0.0009465	0.021473737
NODE_12706_length_3301_cov_28.345105_g6366_i0	Control	Salt_treated	6.881962519	0.416787635	6.07901E-05	0.003110432
NODE_40703_length_1911_cov_29.692601_g20378_i0	Control	Salt_treated	6.87584278	0.401724105	0.000427109	0.012493841
BINPACKER_29795_6	Control	Salt_treated	6.872020484	0.412260687	0.000134301	0.00557961
Contig11629	Control	Salt_treated	6.871808575	0.400199068	0.000287367	0.009351961
BINPACKER_1287_10	Control	Salt_treated	6.860319145	0.407734111	0.00044555	0.012850226
NODE_41031_length_1902_cov_33.442318_g20531_i0	Control	Salt_treated	6.856109665	0.406225344	0.000678596	0.017182449
BINPACKER_11306_11	Control	Salt_treated	6.851051225	0.391049741	5.47473E-05	0.002871236
BINPACKER_47657_1	Control	Salt_treated	6.833954327	0.370918374	0.000315789	0.010001227
BINPACKER_11937_1	Control	Salt_treated	6.826276225	0.367836302	0.000150522	0.006021586
BINPACKER_17938_1	Control	Salt_treated	6.81323278	0.361672913	7.47662E-05	0.003604046
NODE_13540_length_3219_cov_31.952956_g6800_i0	Control	Salt_treated	6.81013033	0.360132234	8.20521E-05	0.00386454
Contig652	Control	Salt_treated	6.803515291	0.344107152	0.000601546	0.015857183
BINPACKER_8852_3	Control	Salt_treated	6.791581528	0.352429941	0.000433315	0.012603585
BINPACKER_12014_1	Control	Salt_treated	6.791162708	0.339434484	0.000181233	0.006778908
NODE_96359_length_896_cov_11.773998_g52515_i0	Control	Salt_treated	6.791162708	0.339434484	0.000181233	0.006778908
BINPACKER_12468_3	Control	Salt_treated	6.782769769	0.349349573	0.001255209	0.025803437
BINPACKER_1065_7	Control	Salt_treated	6.782174949	0.319838619	0.003184146	0.04748471
BINPACKER_4939_7	Control	Salt_treated	6.782174949	0.319838619	0.003184146	0.04748471
BINPACKER_8665_1	Control	Salt_treated	6.772787374	0.31668909	0.001470282	0.028483254
NODE_39020_length_1960_cov_27.996820_g18901_i1	Control	Salt_treated	6.751287365	0.308816887	0.000205236	0.007379218
BINPACKER_12996_4	Control	Salt_treated	6.740909501	0.304094755	0.000102056	0.004555897
BINPACKER_8348_13	Control	Salt_treated	6.734547204	0.300947195	0.000111584	0.004917134
BINPACKER_577_8	Control	Salt_treated	6.727490694	0.297800076	0.000190076	0.0070328
BINPACKER_11804_3	Control	Salt_treated	6.718456472	0.280676751	0.000372885	0.011401747
Contig10738	Control	Salt_treated	6.71489013	0.293080258	0.000824259	0.019590449
BINPACKER_12408_1	Control	Salt_treated	6.706702408	0.275902605	0.000179423	0.006772887
BINPACKER_19701_1	Control	Salt_treated	6.696873708	0.271129581	0.000144536	0.005865709
NODE_116468_length_696_cov_28.495987_g67442_i0	Control	Salt_treated	6.676361139	0.248650581	0.000414172	0.012206726
BINPACKER_7001_3	Control	Salt_treated	6.668296971	0.245432645	0.000202245	0.007311807
NODE_23146_length_2571_cov_26.692954_g11564_i0	Control	Salt_treated	6.651240559	0.237390398	0.000194833	0.00719584
BINPACKER_3926_14	Control	Salt_treated	6.647357022	0.23578241	0.000373673	0.011401747
NODE_43584_length_1834_cov_28.304940_g21802_i1	Control	Salt_treated	6.633863481	0.230959406	0.001240342	0.025668223
BINPACKER_8735_1	Control	Salt_treated	6.625081215	0.212598134	0.000243713	0.008341077
BINPACKER_23634_1	Control	Salt_treated	6.621567571	0.210971504	0.000200309	0.007286064
BINPACKER_52169_1	Control	Salt_treated	6.61493487	0.207718768	0.000197864	0.007252861
NODE_57764_length_1504_cov_9.523410_g29131_i0	Control	Salt_treated	6.603414641	0.202841	0.000465875	0.013232016
Contig5570	Control	Salt_treated	6.584380609	0.180597806	0.000276107	0.009100901
BINPACKER_8843_3	Control	Salt_treated	6.584380609	0.180597806	0.000276107	0.009100901
BINPACKER_5016_1	Control	Salt_treated	6.573855982	0.175663075	0.000265318	0.008873449
BINPACKER_9529_1	Control	Salt_treated	6.573855982	0.175663075	0.000265318	0.008873449
BINPACKER_16001_9	Control	Salt_treated	6.566654787	0.172374261	0.000388352	0.011662037
BINPACKER_29664_1	Control	Salt_treated	6.534924272	0.144455905	0.000277617	0.00913599

BINPACKER_6377_6	Control	Salt_treated	6.528066047	0.141128916	0.000279824	0.009175756
BINPACKER_5680_4	Control	Salt_treated	6.504406231	0.115797736	0.001133738	0.024175816
Contig10299	Control	Salt_treated	6.484355045	0.107381515	0.000389019	0.011662037
Contig11883	Control	Salt_treated	6.484355045	0.107381515	0.000389019	0.011662037
BINPACKER_37985_1	Control	Salt_treated	6.446596161	0.076136309	0.000494519	0.013776514
BINPACKER_6130_2	Control	Salt_treated	6.435614984	0.071028524	0.000608514	0.015979323
BINPACKER_4089_1	Control	Salt_treated	6.431716207	0.069326507	0.000746018	0.018457194
Contig2810	Control	Salt_treated	6.41554319	0.045745701	0.002472945	0.040560648
NODE_79026_length_1123_cov_29.888571_g34668_i3	Control	Salt_treated	6.400429543	0.040573922	0.000821382	0.019562883
Contig11053	Control	Salt_treated	6.400429543	0.040573922	0.000821382	0.019562883
BINPACKER_15788_4	Control	Salt_treated	6.396306323	0.038850624	0.000645551	0.016549789
BINPACKER_2296_14	Control	Salt_treated	6.388940587	0.035404983	0.000581141	0.01543819
BINPACKER_8668_1	Control	Salt_treated	6.385118994	0.033682645	0.000678566	0.017182449
BINPACKER_23022_1	Control	Salt_treated	6.376053844	0.030238947	0.001274767	0.02602122
BINPACKER_5106_10	Control	Salt_treated	6.370755176	0.028517592	0.002573135	0.041777781
NODE_9499_length_3642_cov_25.698515_g2480_i1	Control	Salt_treated	6.357867745	0.005751426	0.001951663	0.034484696
NODE_58031_length_1499_cov_8.133240_g29273_i0	Control	Salt_treated	6.344346034	0.000518634	0.000882541	0.020570929
Contig8771	Control	Salt_treated	6.344346034	0.000518634	0.000882541	0.020570929
NODE_56160_length_1537_cov_35.086066_g28317_i0	Control	Salt_treated	6.332689683	-0.004710971	0.001137603	0.024207954
BINPACKER_13620_2	Control	Salt_treated	6.32798023	-0.006453453	0.001553147	0.029558728
NODE_75172_length_1183_cov_27.541441_g38909_i0	Control	Salt_treated	6.29884489	-0.035388658	0.00121324	0.02539641
Contig4962	Control	Salt_treated	6.29884489	-0.035388658	0.00121324	0.02539641
NODE_6451_length_4141_cov_28.174287_g3268_i0	Control	Salt_treated	6.28677998	-0.040683735	0.000874547	0.020500936
Contig11505	Control	Salt_treated	6.282726115	-0.042447974	0.001505798	0.029007098
NODE_155129_length_456_cov_2.879896_g101423_i0	Control	Salt_treated	6.278134959	-0.044211815	0.001950831	0.034484696
NODE_20875_length_2695_cov_26.006102_g10403_i0	Control	Salt_treated	6.234813835	-0.079527201	0.001268236	0.025965327
BINPACKER_153_4	Control	Salt_treated	6.23082592	-0.081313423	0.001398524	0.027429501
NODE_130601_length_588_cov_6.646602_g79425_i0	Control	Salt_treated	6.23082592	-0.081313423	0.001398524	0.027429501
BINPACKER_17291_2	Control	Salt_treated	6.221249625	-0.084884557	0.002406803	0.039922213
NODE_14267_length_3157_cov_30.187743_g7147_i0	Control	Salt_treated	6.184942848	-0.11775796	0.002108743	0.036389104
NODE_42987_length_1850_cov_23.106922_g21477_i0	Control	Salt_treated	6.134304723	-0.15721337	0.003074834	0.046739783
NODE_60884_length_1439_cov_17.707174_g30780_i0	Control	Salt_treated	6.124734732	-0.16087905	0.001994847	0.035042075
BINPACKER_16212_1	Control	Salt_treated	6.124734732	-0.16087905	0.001994847	0.035042075
NODE_5958_length_4249_cov_27.417625_g3023_i0	Control	Salt_treated	6.120641156	-0.162711113	0.001933204	0.034281823
BINPACKER_4779_3	Control	Salt_treated	6.120641156	-0.162711113	0.001933204	0.034281823
NODE_20176_length_2740_cov_26.490814_g10041_i0	Control	Salt_treated	6.120641156	-0.162711113	0.001933204	0.034281823
NODE_40615_length_1913_cov_27.505978_g1959_i3	Control	Salt_treated	6.066406073	-0.203544968	0.003215123	0.047773186
BINPACKER_34626_1	Control	Salt_treated	6.066406073	-0.203544968	0.003215123	0.047773186
BINPACKER_15709_1	Control	Salt_treated	6.057846834	-0.207256352	0.003059752	0.046632708
BINPACKER_115089_1	Control	Salt_treated	5.509563862	1.717495048	0.000000032	7.00541E-06
BINPACKER_20431_1	Control	Salt_treated	5.367370111	2.379222946	6.61931E-11	3.08926E-08
NODE_14157_length_3166_cov_29.543485_g7091_i0	Control	Salt_treated	5.23479732	2.435709239	2.60774E-07	3.85252E-05
BINPACKER_21821_1	Control	Salt_treated	4.951194038	1.244458043	2.10289E-06	0.000218651
BINPACKER_44392_1	Control	Salt_treated	4.848716939	1.155533363	2.22464E-06	0.000229563
Contig10907	Control	Salt_treated	4.741730031	1.150296128	7.35434E-05	0.003561825
NODE_103985_length_815_cov_4.334232_g57781_i0	Control	Salt_treated	4.716348052	1.955376412	1.94662E-06	0.000208197
NODE_65631_length_1348_cov_18.066667_g14569_i2	Control	Salt_treated	4.637759911	0.984252308	0.00006741	0.003319577
BINPACKER_35519_1	Control	Salt_treated	4.620502009	2.90038608	3.30864E-06	0.000312153
NODE_3307_length_5078_cov_28.543257_g1703_i0	Control	Salt_treated	4.585043022	1.762667609	2.76335E-07	4.05324E-05
NODE_12856_length_3287_cov_24.713752_g6441_i0	Control	Salt_treated	4.566400396	0.926231569	7.50173E-06	0.00059478
BINPACKER_20779_7	Control	Salt_treated	4.537022499	0.904071979	0.000008692	0.000661077
NODE_118502_length_679_cov_4.914191_g69090_i0	Control	Salt_treated	4.41278122	0.883453768	0.000570905	0.015225373
NODE_21396_length_2667_cov_28.594063_g10656_i0	Control	Salt_treated	4.381263553	0.778183097	5.04743E-05	0.002746118
BINPACKER_33_2	Control	Salt_treated	4.370016384	1.517827696	1.41369E-06	0.000157772
BINPACKER_11579_11	Control	Salt_treated	4.364406449	3.149497462	1.3894E-10	5.59438E-08
Contig11901	Control	Salt_treated	4.320967353	0.72473322	4.77287E-05	0.0026347
NODE_16737_length_2967_cov_29.620594_g8302_i0	Control	Salt_treated	4.313878504	0.722044368	3.31217E-05	0.001982955
NODE_72790_length_1222_cov_32.975631_g37477_i0	Control	Salt_treated	4.312107353	1.468727743	2.69445E-06	0.00026474
NODE_6835_length_4061_cov_15.106068_g3451_i0	Control	Salt_treated	4.295480527	0.807488967	0.002679207	0.042881931
BINPACKER_16485_2	Control	Salt_treated	4.286591115	1.521539849	2.02329E-05	0.001364801
NODE_9448_length_3650_cov_15.866648_g3508_i3	Control	Salt_treated	4.281625915	0.697733753	3.88592E-05	0.00224057
BINPACKER_21348_2	Control	Salt_treated	4.22808461	0.621484321	0.001686332	0.03133831
Contig946	Control	Salt_treated	4.175542384	0.682819479	0.000701356	0.017606789
NODE_7365_length_3971_cov_32.382247_g3718_i0	Control	Salt_treated	4.123232273	1.310445827	3.40875E-06	0.000318176
NODE_6566_length_4117_cov_31.886251_g3327_i0	Control	Salt_treated	4.120258087	1.474066189	0.000188247	0.006990326
BINPACKER_34092_11	Control	Salt_treated	4.099735121	2.684660157	6.25153E-09	1.68915E-06
Contig8450	Control	Salt_treated	4.098957634	0.539560907	0.000450314	0.012915092
BINPACKER_2733_1	Control	Salt_treated	4.090070544	0.54249524	0.001509977	0.029041839
NODE_114169_length_716_cov_29.589425_g54900_i1	Control	Salt_treated	4.089771399	1.438801692	0.000122227	0.00526192
NODE_115456_length_705_cov_18.408228_g66616_i0	Control	Salt_treated	4.06354923	0.526509493	0.000196979	0.007236075
NODE_99993_length_855_cov_27.966752_g55030_i0	Control	Salt_treated	4.06354923	0.526509493	0.000196979	0.007236075
NODE_47156_length_1743_cov_29.588623_g23647_i0	Control	Salt_treated	4.0549827	2.251612558	2.36845E-06	0.000238413
Contig96	Control	Salt_treated	4.047997537	1.23910453	8.78986E-06	0.000666051
NODE_26086_length_2427_cov_25.486831_g12875_i1	Control	Salt_treated	4.024471429	1.222439599	7.4189E-06	0.000592458
BINPACKER_9432_2	Control	Salt_treated	4.022516291	0.562543979	0.00145871	0.028339264
Contig4168	Control	Salt_treated	4.007946556	2.131857896	5.86172E-07	7.71605E-05
NODE_9551_length_3635_cov_31.888827_g4825_i0	Control	Salt_treated	4.000987427	2.069350954	0.000000147	2.37666E-05
BINPACKER_8827_4	Control	Salt_treated	3.998189768	2.065905927	0.000000146	2.37666E-05
NODE_146520_length_496_cov_7.626478_g93504_i0	Control	Salt_treated	3.997253066	0.540900109	0.001896307	0.033867101
BINPACKER_7513_1	Control	Salt_treated	3.99393828	0.471403804	0.000473738	0.013362934
BINPACKER_9545_4	Control	Salt_treated	3.989942597	0.458705511	0.000478184	0.013465012
NODE_49890_length_1678_cov_29.505296_g25025_i0	Control	Salt_treated	3.987450519	2.928360549	1.68066E-09	5.00178E-07
NODE_146604_length_496_cov_2.359338_g93579_i0	Control	Salt_treated	3.97434019	0.454222072	0.000218074	0.007628867
NODE_8696_length_3756_cov_32.238393_g157_i3	Control	Salt_treated	3.930512169	0.423094211	0.000258016	0.008660958
NODE_20143_length_2743_cov_19.122472_g9653_i1	Control	Salt_treated	3.929212468	0.487433438	0.002272676	0.038379444
NODE_91831_length_950_cov_21.492588_g49470_i2	Control	Salt_treated	3.859558684	0.368088646	0.000330045	0.010331499
NODE_75174_length_1183_cov_27.322523_g38910_i0	Control	Salt_treated	3.845578114	1.797350583	0.000179034	0.006770634
NODE_8241_length_3829_cov_31.342918_g3327_i1	Control	Salt_treated	3.833702337	4.438308288	3.8189E-12	2.61404E-09
NODE_128498_length_602_cov_17.124764_g77610_i0	Control	Salt_treated	3.747287307	0.280899582	0.000678586	0.017182449
BINPACKER_10846_2	Control	Salt_treated	3.737196045	0.276123339	0.000662645	0.016903608
NODE_83981_length_1052_cov_31.492339_g44342_i0	Control	Salt_treated	3.723754993	1.149731984	0.00129632	0.02622653
BINPACKER_35722_5	Control	Salt_treated	3.721310547	0.253692139	0.001747741	0.032246047
BINPACKER_807_2	Control	Salt_treated	3.711956716	1.693730928	0.000416002	0.012238688
BINPACKER_86140_1	Control	Salt_treated	3.698829901	4.072548042	1.37985E-08	3.49817E-06
NODE_93219_length_933_cov_42.606977_g50412_i0	Control	Salt_treated	3.691813732	0.22419245	0.002993591	0.046047485
BINPACKER_2864_4	Control	Salt_treated	3.674228882	2.488394137	0.001026403	0.022688037
Contig294	Control	Salt_treated	3.651417369	2.300055916	6.56544E-07	8.37399E-05
NODE_70548_length_1261_cov_7.674242_g36155_i0	Control	Salt_treated	3.627908339	0.187369674	0.001759048	0.03232046
NODE_93964_length_924_cov_34.935370_g50904_i0	Control	Salt_treated	3.607452342	0.179138529	0.001379175	0.027232072
NODE_53066_length_1603_cov_31.833333_g1978_i5	Control	Salt_treated	3.570197999	1.045787994	0.002220304	0.03761876
NODE_43361_length_1840_cov_14.741935_g21686_i0	Control	Salt_treated	3.567797008	1.442217355	0.000113967	0.004979374
NODE_40583_length_1914_cov_25.437806_g16499_i4	Control	Salt_treated	3.55814981	0.927416445	0.000876293	0.020518451
NODE_43823_length_1827_cov_27.478335_g12605_i2	Control	Salt_treated	3.529509398	0.117645082	0.001912463	0.034090657
BINPACKER_14737_3	Control	Salt_treated	3.521977544	0.815719916	0.00016979	0.006574468
NODE_3386_length_5048_cov_33.671156_g1737_i0	Control	Salt_treated	3.520093523	0.114275832	0.002204367	0.037421252
NODE_6323_length_4169_cov_30.266357_g3202_i0	Control	Salt_treated	3.513764498	0.878784713	0.000357516	0.011039991
NODE_33186_length_2153_cov_12.391827_g16461_i1	Control	Salt_treated	3.503984988	0.796883502	0.000249098	0.00845493
BINPACKER_8895_7	Control	Salt_treated	3.501063049	0.805338039	0.000159351	0.006280747
NODE_101193_length_843_cov_17.431169_g55836_i0	Control	Salt_treated	3.489930177	0.859541142	0.000357195	0.011039991
NODE_46419_length_1760_cov_23.429164_g19909_i1	Control	Salt_treated	3.489674261	2.819532469	1.72973E-05	0.001204067
BINPACKER_51132_1	Control	Salt_treated	3.467693926	0.076283471	0.002581758	0.041778094
BINPACKER_14715_1	Control	Salt_treated	3.467693926	0.076283471	0.002581758	0.041778094
NODE_46139_length_1766_cov_28.563497_g12230_i4	Control	Salt_treated	3.466012962	0.074579827	0.003245451	0.048015369

NODE_84715_length_1042_cov_22.172343_g44827_i0	Control	Salt_treated	3.464304965	1.222891675	2.18481E-05	0.001442604
BINPACKER_24505_4	Control	Salt_treated	3.445979877	2.357507756	8.87661E-07	0.000105978
NODE_30410_length_2254_cov_25.692801_g13876_i1	Control	Salt_treated	3.423274719	0.911050011	0.003050815	0.046578805
NODE_44208_length_1816_cov_21.724613_g7732_i1	Control	Salt_treated	3.404420091	1.308420672	0.000311201	0.00993858
BINPACKER_7299_3	Control	Salt_treated	3.400224367	0.889621071	0.003188932	0.047508009
NODE_59615_length_1464_cov_13.142344_g30121_i0	Control	Salt_treated	3.399742316	0.888352441	0.00304452	0.046578805
NODE_31281_length_2221_cov_25.865456_g15662_i0	Control	Salt_treated	3.395855119	1.167253104	5.48101E-05	0.002871236
BINPACKER_33045_15	Control	Salt_treated	3.390868309	1.299831069	0.00043288	0.012603585
NODE_43594_length_1834_cov_24.242476_g18773_i1	Control	Salt_treated	3.388220902	1.160873983	3.36513E-05	0.002002983
NODE_23312_length_2562_cov_27.504219_g11643_i1	Control	Salt_treated	3.376675054	1.150844419	7.57693E-05	0.003643849
BINPACKER_37566_1	Control	Salt_treated	3.371691555	0.005871293	0.003237813	0.047936901
NODE_41240_length_1897_cov_23.583333_g20558_i1	Control	Salt_treated	3.371691555	0.005871293	0.003237813	0.047936901
BINPACKER_8183_1	Control	Salt_treated	3.371691555	0.005871293	0.003237813	0.047936901
NODE_804_length_7342_cov_33.630210_g438_i0	Control	Salt_treated	3.370575445	1.473908227	0.000026506	0.001664526
NODE_21064_length_2685_cov_34.379786_g9149_i1	Control	Salt_treated	3.361159672	1.468155451	0.000031896	0.00193211
BINPACKER_5148_9	Control	Salt_treated	3.347531179	0.682934542	0.000243332	0.008341077
NODE_32549_length_2176_cov_31.285782_g16286_i0	Control	Salt_treated	3.32525606	0.661998046	0.000404567	0.012022839
Contig11604	Control	Salt_treated	3.31334736	4.153980359	1.07383E-09	3.61492E-07
NODE_41426_length_1892_cov_28.278725_g20709_i0	Control	Salt_treated	3.305853333	0.652320114	0.000413658	0.012206726
NODE_39964_length_1932_cov_29.710059_g20022_i0	Control	Salt_treated	3.293750653	0.707875175	0.001321184	0.026520548
BINPACKER_15074_1	Control	Salt_treated	3.293169195	0.62040767	0.001537604	0.029391653
NODE_46601_length_1756_cov_25.348782_g23367_i0	Control	Salt_treated	3.251652926	1.820764493	9.91491E-05	0.004445456
BINPACKER_11257_3	Control	Salt_treated	3.225228502	0.588375202	0.000511211	0.014034378
BINPACKER_10286_2	Control	Salt_treated	3.221100977	0.586952676	0.000462079	0.013142377
NODE_60554_length_1445_cov_27.451895_g19299_i3	Control	Salt_treated	3.221100977	0.586952676	0.000462079	0.013142377
NODE_145256_length_502_cov_7.375291_g92330_i0	Control	Salt_treated	3.217904873	0.585530089	0.000448946	0.012893855
Contig11757	Control	Salt_treated	3.20943926	1.477494321	0.000249695	0.008461205
BINPACKER_10650_1	Control	Salt_treated	3.193698232	3.182442651	8.13181E-08	1.47776E-05
NODE_140741_length_527_cov_5.568282_g88263_i0	Control	Salt_treated	3.188981156	1.00301105	0.000129887	0.005443321
BINPACKER_9733_2	Control	Salt_treated	3.184062144	0.619079383	0.001770665	0.03249384
NODE_65977_length_1341_cov_38.010252_g33593_i0	Control	Salt_treated	3.182415854	0.552859091	0.001600719	0.030156667
BINPACKER_13539_1	Control	Salt_treated	3.181126608	0.555732515	0.000932405	0.021203698
NODE_65949_length_1342_cov_12.263199_g33571_i0	Control	Salt_treated	3.172085426	1.90465335	3.64853E-05	0.00212848
BINPACKER_14080_3	Control	Salt_treated	3.149656115	0.517837143	0.001861061	0.033582503
NODE_43243_length_1842_cov_36.015828_g7508_i3	Control	Salt_treated	3.148112791	0.592658473	0.002328738	0.039045956
NODE_42294_length_1868_cov_32.047911_g21139_i0	Control	Salt_treated	3.14538993	3.722216393	0.002242826	0.03796902
BINPACKER_81_8	Control	Salt_treated	3.133969715	3.35055848	8.76975E-05	0.004028788
NODE_35536_length_2071_cov_26.419920_g17817_i0	Control	Salt_treated	3.132451951	3.942259245	3.90904E-09	1.11489E-06
NODE_27431_length_2369_cov_31.353223_g13755_i0	Control	Salt_treated	3.117533211	6.371681513	2.9706E-06	0.000285053
BINPACKER_38139_1	Control	Salt_treated	3.117254187	0.509046342	0.000769393	0.01882823
BINPACKER_3981_1	Control	Salt_treated	3.11456801	0.504650637	0.001252459	0.025796643
BINPACKER_12784_3	Control	Salt_treated	3.110162735	3.004297765	7.93238E-07	9.81274E-05
NODE_730_length_7518_cov_27.059503_g359_i1	Control	Salt_treated	3.103009649	0.934438323	0.000204365	0.00736252
BINPACKER_35343_2	Control	Salt_treated	3.08440522	2.163838454	8.95511E-06	0.000676078
NODE_28113_length_2341_cov_28.254850_g10685_i1	Control	Salt_treated	3.082997129	1.483152423	0.002349017	0.039249038
BINPACKER_38802_1	Control	Salt_treated	3.075547779	3.222506139	4.88725E-05	0.002676257
BINPACKER_102450_1	Control	Salt_treated	3.068019225	1.216276271	0.000200824	0.007286064
NODE_56709_length_1526_cov_24.059876_g28592_i0	Control	Salt_treated	3.06537716	1.795972023	0.001855044	0.033537966
NODE_45180_length_1791_cov_29.323050_g22645_i0	Control	Salt_treated	3.059290467	2.700246907	7.18563E-07	9.05257E-05
Contig7873	Control	Salt_treated	3.056237416	1.036404596	0.002773223	0.04376033
NODE_43495_length_1837_cov_11.702381_g21760_i0	Control	Salt_treated	3.046531768	0.951291478	0.00126048	0.025858091
NODE_86745_length_1015_cov_26.973461_g21458_i2	Control	Salt_treated	3.04530957	0.454502726	0.001113343	0.023843137
NODE_155914_length_452_cov_16.163588_g102166_i0	Control	Salt_treated	3.040353729	1.020486107	0.002467744	0.040540105
Contig1457	Control	Salt_treated	3.040148068	1.329868505	0.000388412	0.011662037
Contig1282	Control	Salt_treated	3.035759419	1.326752659	0.000359098	0.011072188
NODE_68223_length_1302_cov_5.013019_g34806_i0	Control	Salt_treated	3.030903407	1.516164632	0.000118628	0.005139291
NODE_23273_length_2564_cov_32.279004_g11624_i0	Control	Salt_treated	3.024737293	3.515419467	0.002336584	0.039073087
NODE_64148_length_1375_cov_25.809524_g8680_i1	Control	Salt_treated	3.011706242	0.429406612	0.001288774	0.02612424
BINPACKER_30651_1	Control	Salt_treated	3.002051574	1.625198421	0.000767218	0.018800509
BINPACKER_27329_1	Control	Salt_treated	2.99607213	1.545999161	0.0001473	0.005930985
NODE_12132_length_3357_cov_22.911998_g6077_i0	Control	Salt_treated	2.985891843	1.752327026	0.000125963	0.005377631
NODE_57402_length_1512_cov_9.475330_g14628_i5	Control	Salt_treated	2.983531885	1.154631833	0.00037026	0.01134818
BINPACKER_27905_2	Control	Salt_treated	2.980773769	2.634465257	1.39679E-06	0.000157068
BINPACKER_23949_1	Control	Salt_treated	2.980336949	1.139334943	0.00049864	0.013835903
BINPACKER_3826_4	Control	Salt_treated	2.9486603	3.878339538	1.34021E-05	0.000955596
BINPACKER_25200_2	Control	Salt_treated	2.936151048	3.336610629	0.00026677	0.008893046
NODE_37624_length_2003_cov_15.051295_g18872_i0	Control	Salt_treated	2.933070371	1.10082167	0.001543596	0.029431518
BINPACKER_36694_1	Control	Salt_treated	2.927867365	1.4957287	0.000240166	0.008274854
NODE_79270_length_1120_cov_7.931232_g41403_i0	Control	Salt_treated	2.925767637	1.361108816	0.000169182	0.006574468
NODE_21860_length_2642_cov_22.668353_g10903_i0	Control	Salt_treated	2.919585031	0.790713422	0.000634242	0.016362018
BINPACKER_19181_1	Control	Salt_treated	2.917828552	1.173729337	0.000458234	0.013095078
BINPACKER_40575_1	Control	Salt_treated	2.913585412	0.84812171	0.001287094	0.026117061
NODE_2216_length_5718_cov_29.516918_g1128_i0	Control	Salt_treated	2.898499416	1.853894576	2.30893E-05	0.001495707
NODE_17595_length_2901_cov_15.734088_g8733_i0	Control	Salt_treated	2.893155789	1.218442086	0.001696881	0.031505836
BINPACKER_95997_1	Control	Salt_treated	2.89035413	0.829641828	0.001636482	0.030689632
NODE_83814_length_1055_cov_13.148676_g44236_i0	Control	Salt_treated	2.880942068	1.8368348	0.000128401	0.005403113
Contig757	Control	Salt_treated	2.871379979	1.514746445	0.00115243	0.024447469
BINPACKER_1205_7	Control	Salt_treated	2.870564851	1.194658158	0.000896792	0.020714978
NODE_60231_length_1451_cov_27.664006_g8802_i3	Control	Salt_treated	2.864987185	0.749339571	0.000885389	0.020613908
BINPACKER_18135_7	Control	Salt_treated	2.864745671	0.746673278	0.001194717	0.025136806
BINPACKER_10248_1	Control	Salt_treated	2.864000354	3.696564389	2.70431E-05	0.00169308
BINPACKER_42458_1	Control	Salt_treated	2.859436314	2.534029522	0.001277894	0.02604158
Contig8752	Control	Salt_treated	2.855756962	0.314009012	0.002206824	0.037421252
NODE_53749_length_1589_cov_6.121372_g27063_i0	Control	Salt_treated	2.853798803	0.312433193	0.003236144	0.047936901
BINPACKER_27836_6	Control	Salt_treated	2.851386198	4.138187411	0.000316572	0.010001227
NODE_64229_length_1374_cov_15.089931_g32617_i0	Control	Salt_treated	2.844892946	3.450097865	5.61901E-07	7.49262E-05
BINPACKER_9186_1	Control	Salt_treated	2.84432427	1.048102445	0.00040884	0.012114773
BINPACKER_4357_2	Control	Salt_treated	2.837671406	1.034800751	0.000621175	0.016167088
NODE_32339_length_2185_cov_10.960701_g16179_i0	Control	Salt_treated	2.829651485	3.58247383	1.12222E-05	0.000817195
BINPACKER_947_2	Control	Salt_treated	2.82947252	1.287232976	0.00024445	0.008346787
BINPACKER_1461_7	Control	Salt_treated	2.812871138	0.282715627	0.003048047	0.046578805
BINPACKER_9218_2	Control	Salt_treated	2.811340991	3.493053696	0.000000109	1.89683E-05
BINPACKER_13439_10	Control	Salt_treated	2.807732678	1.015366172	0.00060255	0.015863276
NODE_136181_length_553_cov_24.610417_g84192_i1	Control	Salt_treated	2.780072122	0.684651905	0.001006307	0.022410059
NODE_136180_length_553_cov_24.633333_g84192_i0	Control	Salt_treated	2.780072122	0.684651905	0.001006307	0.022410059
BINPACKER_4644_11	Control	Salt_treated	2.768957973	0.737914053	0.002046392	0.035689399
NODE_22774_length_2591_cov_26.875695_g11370_i0	Control	Salt_treated	2.761260829	1.69493761	5.41649E-05	0.002871236
NODE_23202_length_2568_cov_28.131463_g11588_i0	Control	Salt_treated	2.755194714	1.108853833	0.002332284	0.039064797
NODE_18274_length_2857_cov_27.122845_g9087_i0	Control	Salt_treated	2.729321554	2.284554773	1.21414E-05	0.000874819
BINPACKER_9794_1	Control	Salt_treated	2.727479229	1.023509245	0.001591913	0.030063616
BINPACKER_97815_1	Control	Salt_treated	2.722858878	0.954184548	0.000796842	0.019296174
NODE_123699_length_638_cov_4.272566_g73482_i0	Control	Salt_treated	2.715013036	1.192048684	0.0007552	0.018594756
BINPACKER_6513_4	Control	Salt_treated	2.711401226	2.465137603	8.50512E-06	0.00065413
NODE_3131_length_5157_cov_25.003934_g1609_i0	Control	Salt_treated	2.701322971	1.692066342	0.000321482	0.010094239
Contig9709	Control	Salt_treated	2.694639917	2.586741724	0.002735788	0.043415306
Contig9766	Control	Salt_treated	2.685014568	2.573438336	0.002317258	0.038940177
BINPACKER_3840_1	Control	Salt_treated	2.679383889	1.952433382	0.001335584	0.026691478
BINPACKER_39251_2	Control	Salt_treated	2.670965067	3.334766601	0.000152578	0.006083847
NODE_48810_length_1704_cov_29.347026_g24468_i0	Control	Salt_treated	2.661759301	0.592965672	0.002139867	0.036802482
NODE_126826_length_614_cov_29.730129_g76167_i0	Control	Salt_treated	2.659913337	1.598433243	0.000535094	0.014534595
NODE_51607_length_1637_cov_34.050512_g25894_i0	Control	Salt_treated	2.653764174	0.900326054	0.001095248	0.023739656
Contig2851	Control	Salt_treated	2.645045955	2.490149084	2.36262E-05	0.001512061
BINPACKER_15897_5	Control	Salt_treated	2.644444447	2.407069964	0.000021436	0.001424559

Contig3734	Control	Salt_treated	2.632400442	1.503935266	0.000748934	0.018484805
NODE_40665_length_1912_cov_28.119630_g19011_i1	Control	Salt_treated	2.632143192	2.755154238	0.000037701	0.002193173
BINPACKER_14331_1	Control	Salt_treated	2.614780058	1.740377704	0.001032189	0.022766919
NODE_40225_length_1925_cov_22.261879_g20145_i0	Control	Salt_treated	2.613628561	1.314206112	0.001111673	0.023843137
Contig5132	Control	Salt_treated	2.6114877	1.744399528	0.001318584	0.026494238
NODE_25805_length_2440_cov_15.113646_g12530_i1	Control	Salt_treated	2.60170384	1.505693583	0.002303404	0.03880263
NODE_78776_length_1127_cov_24.803605_g10836_i1	Control	Salt_treated	2.600919989	1.30676069	0.000809793	0.019472015
Contig389	Control	Salt_treated	2.590784939	0.541627144	0.002578611	0.041778094
NODE_18399_length_2849_cov_25.683718_g9159_i0	Control	Salt_treated	2.590165633	4.937620712	0.000331192	0.010335921
NODE_42655_length_1859_cov_18.964726_g21315_i0	Control	Salt_treated	2.589832126	2.359058258	8.68496E-05	0.00400777
NODE_31159_length_2225_cov_26.401487_g15611_i0	Control	Salt_treated	2.586555885	0.537273519	0.003253165	0.048060241
Contig1202	Control	Salt_treated	2.583885081	2.135366397	0.001969168	0.034679995
Contig9690	Control	Salt_treated	2.567678641	2.905569619	0.000206019	0.007383232
BINPACKER_836_2	Control	Salt_treated	2.560230219	3.028349944	0.001815754	0.033026136
NODE_77736_length_1143_cov_6.393458_g40468_i0	Control	Salt_treated	2.560091264	0.8935102	0.002043978	0.035689399
BINPACKER_14096_2	Control	Salt_treated	2.558149346	1.069824765	0.001336199	0.026691478
NODE_149838_length_480_cov_3.275184_g96544_i0	Control	Salt_treated	2.545490558	3.595080313	0.000874436	0.020500936
BINPACKER_5696_5	Control	Salt_treated	2.54116316	1.616159501	0.000678514	0.017182449
BINPACKER_8095_7	Control	Salt_treated	2.538278619	1.129674393	0.00341381	0.049841995
Contig9799	Control	Salt_treated	2.524354063	0.800254463	0.002103084	0.036352552
NODE_87888_length_1000_cov_20.591154_g46876_i0	Control	Salt_treated	2.51649111	2.635889614	0.000140162	0.005722125
NODE_39733_length_1938_cov_33.428954_g19915_i0	Control	Salt_treated	2.514544529	1.317684059	0.000817035	0.019562883
BINPACKER_6268_1	Control	Salt_treated	2.512569957	4.085885739	8.68119E-07	0.000104864
BINPACKER_218_24	Control	Salt_treated	2.503435802	1.997132044	0.002716504	0.04324296
NODE_3823_length_4900_cov_22.443547_g1920_i0	Control	Salt_treated	2.498503889	0.779811702	0.002162239	0.037063078
BINPACKER_23155_2	Control	Salt_treated	2.497341917	1.150161864	0.002101358	0.036352552
NODE_21927_length_2638_cov_26.318129_g10938_i0	Control	Salt_treated	2.496554824	1.087234399	0.002899033	0.045105868
BINPACKER_10113_2	Control	Salt_treated	2.496462804	2.153576962	0.000118061	0.005128747
NODE_48911_length_1702_cov_27.023327_g24517_i0	Control	Salt_treated	2.491775628	2.367771962	0.000143946	0.005853343
BINPACKER_2386_14	Control	Salt_treated	2.488595833	4.118408428	0.000451823	0.012940296
Contig9010	Control	Salt_treated	2.486426769	3.132117956	0.000209979	0.007421562
NODE_36974_length_2023_cov_31.542051_g17647_i2	Control	Salt_treated	2.485596736	1.347416911	0.000908092	0.020812133
NODE_60273_length_1450_cov_31.752360_g30471_i0	Control	Salt_treated	2.480418974	1.293396845	0.001187209	0.025030121
NODE_21034_length_2687_cov_24.735654_g10470_i0	Control	Salt_treated	2.478513376	5.380761435	6.44546E-05	0.003247832
NODE_19094_length_2806_cov_32.031833_g9521_i0	Control	Salt_treated	2.477743708	1.083848825	0.00261063	0.04211255
BINPACKER_662_4	Control	Salt_treated	2.472237554	5.442618614	0.000122835	0.005277007
Contig3087	Control	Salt_treated	2.463241702	1.280460221	0.001010553	0.022410059
BINPACKER_18504_1	Control	Salt_treated	2.459390106	3.32828051	3.62575E-06	0.000332388
NODE_45252_length_1790_cov_16.047758_g19651_i1	Control	Salt_treated	2.45861149	2.387103941	0.000225823	0.007873122
BINPACKER_6635_2	Control	Salt_treated	2.451335613	1.647966989	0.000461272	0.013142377
Contig2800	Control	Salt_treated	2.444486235	2.454819321	0.000148549	0.00595792
NODE_97862_length_879_cov_9.691067_g53563_i0	Control	Salt_treated	2.443486518	0.807320563	0.003061159	0.046632708
NODE_96941_length_889_cov_17.376225_g52922_i0	Control	Salt_treated	2.421234321	2.392272537	6.48112E-05	0.003249915
Contig540	Control	Salt_treated	2.421214055	5.412111252	0.000615929	0.016091376
NODE_82126_length_1078_cov_12.124378_g43160_i2	Control	Salt_treated	2.416021495	3.839700005	0.000822146	0.019562883
NODE_82125_length_1078_cov_12.124378_g43160_i1	Control	Salt_treated	2.416021495	3.839700005	0.000822146	0.019562883
BINPACKER_3075_1	Control	Salt_treated	2.413856605	0.715079751	0.003356677	0.049340995
BINPACKER_14813_1	Control	Salt_treated	2.408288486	0.947579057	0.003423579	0.049895814
BINPACKER_30149_2	Control	Salt_treated	2.40407385	1.927740755	0.00025385	0.008587825
NODE_32452_length_2180_cov_29.209302_g16240_i0	Control	Salt_treated	2.392988949	5.467442114	0.001329736	0.026640126
BINPACKER_6281_1	Control	Salt_treated	2.390625773	1.651802728	0.002461899	0.040477557
Contig919	Control	Salt_treated	2.38658493	3.364194012	0.00012733	0.005380735
NODE_44395_length_1811_cov_28.897008_g16708_i2	Control	Salt_treated	2.386550733	2.531968741	0.000102783	0.004568501
BINPACKER_12742_1	Control	Salt_treated	2.377457296	1.006653476	0.002912173	0.045105868
NODE_11559_length_3416_cov_28.718516_g5783_i0	Control	Salt_treated	2.375523639	1.695916195	0.001799732	0.032851109
Contig4587	Control	Salt_treated	2.375441597	2.916676508	0.000044345	0.002467819
BINPACKER_15816_1	Control	Salt_treated	2.37351964	1.634767902	0.001215789	0.025423847
NODE_101283_length_842_cov_20.478544_g55906_i0	Control	Salt_treated	2.363031328	1.664006187	0.000770183	0.01882823
NODE_80338_length_1104_cov_3.675073_g42052_i0	Control	Salt_treated	2.36218541	2.129844206	0.000244693	0.008346787
BINPACKER_15186_1	Control	Salt_treated	2.353214923	2.939834562	0.001131988	0.024163586
NODE_10556_length_3517_cov_21.878920_g5338_i0	Control	Salt_treated	2.34901874	2.336783735	7.86859E-05	0.003740312
NODE_14194_length_3164_cov_19.043999_g7109_i0	Control	Salt_treated	2.347979053	2.713458706	3.64797E-05	0.00212848
BINPACKER_14746_1	Control	Salt_treated	2.343894219	1.692283966	0.001174541	0.024820621
Contig373	Control	Salt_treated	2.334099706	3.576222599	4.07371E-05	0.002310874
NODE_665_length_7665_cov_23.333641_g364_i0	Control	Salt_treated	2.334081261	1.454090976	0.002412678	0.039953714
NODE_25967_length_2432_cov_18.269606_g13020_i0	Control	Salt_treated	2.322787727	2.717922342	3.26619E-05	0.001966899
NODE_20921_length_2693_cov_28.570992_g10421_i0	Control	Salt_treated	2.320173998	1.677520988	0.001064961	0.023250736
NODE_23201_length_2568_cov_28.422846_g10938_i1	Control	Salt_treated	2.318486535	2.668346903	0.000127911	0.005393523
NODE_21113_length_2683_cov_23.903831_g10507_i0	Control	Salt_treated	2.313669992	2.091367724	0.000214636	0.007521409
BINPACKER_19152_3	Control	Salt_treated	2.311158962	6.419981344	0.001793643	0.032798273
Contig12684	Control	Salt_treated	2.309824989	1.490079565	0.000919237	0.020963343
NODE_53281_length_1598_cov_35.158033_g26811_i0	Control	Salt_treated	2.308206609	4.670638733	4.56488E-06	0.000407564
BINPACKER_14746_4	Control	Salt_treated	2.304684557	2.004093879	0.000164784	0.006470061
Contig8814	Control	Salt_treated	2.303117659	1.740221004	0.000497226	0.013816695
NODE_15240_length_3082_cov_31.338983_g7599_i0	Control	Salt_treated	2.298215057	2.080170902	0.000269906	0.008953981
BINPACKER_6732_1	Control	Salt_treated	2.297419032	2.45723296	0.001106338	0.023822069
NODE_47562_length_1734_cov_19.794100_g23836_i0	Control	Salt_treated	2.296339634	2.089024074	0.001834255	0.033244862
NODE_19357_length_2790_cov_13.847994_g9647_i0	Control	Salt_treated	2.29519319	1.706444798	0.001369957	0.027089654
Contig9841	Control	Salt_treated	2.287208	1.319194621	0.001339186	0.026699205
NODE_56201_length_1537_cov_12.060109_g28333_i0	Control	Salt_treated	2.287206687	2.630571733	0.001930616	0.034281823
BINPACKER_1949_12	Control	Salt_treated	2.283743609	2.675312596	0.000306219	0.009856128
BINPACKER_71406_1	Control	Salt_treated	2.278873659	4.109489007	0.000176819	0.006718551
Contig2995	Control	Salt_treated	2.269236477	2.806812052	0.000519718	0.014192043
NODE_22421_length_2610_cov_28.136776_g9151_i4	Control	Salt_treated	2.268083029	3.974523726	0.000817623	0.019562883
NODE_51277_length_1645_cov_28.403944_g25733_i0	Control	Salt_treated	2.261368647	1.955694822	0.000809354	0.019472015
BINPACKER_17868_1	Control	Salt_treated	2.258355813	1.347888358	0.002200004	0.037398239
BINPACKER_2962_4	Control	Salt_treated	2.257776493	2.536139104	0.000102425	0.004562474
NODE_55528_length_1551_cov_19.123816_g28005_i0	Control	Salt_treated	2.252928474	5.258082253	0.001447027	0.028192314
Contig5069	Control	Salt_treated	2.250645489	5.430876901	0.000958924	0.02161483
NODE_95839_length_902_cov_25.814234_g38871_i2	Control	Salt_treated	2.249186481	2.137621056	0.000213797	0.007510353
NODE_49154_length_1696_cov_26.436229_g24648_i0	Control	Salt_treated	2.249185054	2.550307803	0.000274335	0.009086254
NODE_29123_length_2302_cov_20.581427_g14609_i0	Control	Salt_treated	2.246475708	1.887653433	0.002394182	0.039809341
NODE_8586_length_3771_cov_31.194429_g4318_i0	Control	Salt_treated	2.246283502	1.786518442	0.00310179	0.046818373
BINPACKER_101503_1	Control	Salt_treated	2.244779333	1.488527657	0.001450698	0.028210305
BINPACKER_6981_1	Control	Salt_treated	2.241611397	2.399787964	0.000137013	0.005654591
BINPACKER_3649_7	Control	Salt_treated	2.240020906	1.852324132	0.000485001	0.013605857
BINPACKER_28988_2	Control	Salt_treated	2.238863382	2.170697649	0.000174588	0.00671377
BINPACKER_97842_1	Control	Salt_treated	2.234964979	1.525722209	0.00219042	0.037328021
NODE_32629_length_2173_cov_30.887619_g16322_i0	Control	Salt_treated	2.22967072	4.292116739	0.000888675	0.020620282
NODE_154676_length_458_cov_4.012987_g101002_i0	Control	Salt_treated	2.227585151	2.400773734	0.002901122	0.045105868
NODE_97855_length_879_cov_12.767990_g53559_i0	Control	Salt_treated	2.221239917	3.939951621	0.000196331	0.007236075
NODE_82161_length_1077_cov_28.113546_g43182_i0	Control	Salt_treated	2.215288629	2.575155635	0.003111426	0.046818373
NODE_133746_length_569_cov_4.618952_g82057_i0	Control	Salt_treated	2.199731133	4.27704948	0.001856154	0.033537966
BINPACKER_48328_1	Control	Salt_treated	2.196803541	2.983420908	0.000633354	0.016359646
NODE_35478_length_2074_cov_7.373313_g17788_i0	Control	Salt_treated	2.18596198	3.068410217	5.09994E-05	0.00274938
BINPACKER_20310_1	Control	Salt_treated	2.178029124	2.036221486	0.000439492	0.012711212
BINPACKER_64202_1	Control	Salt_treated	2.176187624	2.315514712	0.000613965	0.016060864
BINPACKER_15872_1	Control	Salt_treated	2.173801152	1.899127229	0.000881209	0.020570929
NODE_90994_length_961_cov_14.326577_g48926_i0	Control	Salt_treated	2.173752024	3.088797566	8.56078E-05	0.003972195
NODE_37862_length_1995_cov_24.855359_g18990_i0	Control	Salt_treated	2.172417952	1.941343385	0.000458503	0.013095078
NODE_17675_length_2894_cov_9.354484_g7553_i1	Control	Salt_treated	2.158171239	1.831000429	0.000709331	0.01778524
NODE_68022_length_1305_cov_29.756494_g34691_i0	Control	Salt_treated	2.15057607	1.460472229	0.003093399	0.046818373
BINPACKER_13992_1	Control	Salt_treated	2.149543283	2.520623185	0.000315994	0.010001227

NODE_5581_length_4345_cov_23.545646_g2833_i0	Control	Salt_treated	2.148425416	2.95599179	0.000134498	0.00557961
BINPACKER_5392_1	Control	Salt_treated	2.137623982	2.125952022	0.000772947	0.018850904
NODE_77926_length_1140_cov_17.153702_g40590_i0	Control	Salt_treated	2.136977424	3.200943157	9.28204E-05	0.004235706
BINPACKER_3564_1	Control	Salt_treated	2.126998722	5.517435337	0.000616699	0.016091376
NODE_60654_length_1443_cov_23.424818_g11756_i2	Control	Salt_treated	2.12312788	2.191680306	0.002568758	0.041777781
NODE_26090_length_2427_cov_24.331351_g13075_i0	Control	Salt_treated	2.119669545	1.841366386	0.001466481	0.028436432
NODE_2121_length_5759_cov_27.088111_g1100_i0	Control	Salt_treated	2.117480881	1.802560288	0.001511966	0.029044175
NODE_119386_length_671_cov_26.901338_g48662_i2	Control	Salt_treated	2.113990583	1.943267146	0.000814232	0.019533005
BINPACKER_3965_2	Control	Salt_treated	2.110813533	2.865305589	9.53975E-05	0.004315575
NODE_19890_length_2758_cov_12.524767_g9892_i0	Control	Salt_treated	2.110706441	1.385097142	0.002118584	0.036528231
NODE_45115_length_1793_cov_30.851744_g22615_i0	Control	Salt_treated	2.110701519	3.522951048	0.001390948	0.02738554
NODE_32949_length_2162_cov_23.864050_g16488_i0	Control	Salt_treated	2.107736565	1.513448998	0.002177859	0.037175664
BINPACKER_69116_2	Control	Salt_treated	2.104962812	3.157113384	4.83742E-05	0.00265983
NODE_53766_length_1588_cov_31.258086_g27073_i0	Control	Salt_treated	2.104085193	1.381033559	0.00249175	0.040771378
BINPACKER_11200_1	Control	Salt_treated	2.102309998	4.71244406	0.000564355	0.015084268
NODE_22938_length_2581_cov_31.678628_g11460_i0	Control	Salt_treated	2.100053025	2.255502332	0.000855967	0.020203779
BINPACKER_11785_1	Control	Salt_treated	2.093375216	1.506760381	0.001960703	0.034589833
NODE_27344_length_2374_cov_15.872229_g13712_i0	Control	Salt_treated	2.088964981	4.619100417	0.000274797	0.009086884
BINPACKER_7472_2	Control	Salt_treated	2.088064557	2.130576486	0.000765955	0.018791986
Contig3313	Control	Salt_treated	2.087153902	2.161725346	0.000808436	0.019472015
Contig3373	Control	Salt_treated	2.082832194	2.419255443	0.000906444	0.020812133
NODE_9012_length_3710_cov_28.254056_g4167_i1	Control	Salt_treated	2.081845594	5.640519996	7.37653E-05	0.003564164
BINPACKER_9352_9	Control	Salt_treated	2.076924734	3.315814297	0.001040867	0.022933689
NODE_39460_length_1947_cov_26.621665_g9151_i11	Control	Salt_treated	2.074484559	3.574808258	0.001540961	0.02940858
NODE_1741_length_6092_cov_29.497923_g896_i2	Control	Salt_treated	2.072941126	2.742615405	0.000139396	0.00570217
NODE_88870_length_987_cov_35.447484_g47511_i0	Control	Salt_treated	2.071725221	2.080430865	0.001107868	0.023822069
NODE_49799_length_1680_cov_30.622900_g24980_i0	Control	Salt_treated	2.065873229	2.300175214	0.001008679	0.022410059
BINPACKER_1112_17	Control	Salt_treated	2.064765135	2.765769692	0.002816748	0.044177632
NODE_14292_length_3155_cov_24.577547_g6369_i1	Control	Salt_treated	2.051430828	2.073960596	0.002716224	0.04324296
NODE_49819_length_1680_cov_19.071562_g24988_i0	Control	Salt_treated	2.047499891	2.327569739	0.001359142	0.026914146
Contig9213	Control	Salt_treated	2.047047269	1.74210094	0.00156454	0.029690381
BINPACKER_43587_1	Control	Salt_treated	2.044998544	4.550601573	0.000515543	0.014115554
NODE_21453_length_2664_cov_22.591663_g10685_i0	Control	Salt_treated	2.044935957	2.618772847	0.000294235	0.009530597
Contig5890	Control	Salt_treated	2.042275134	3.764738651	0.001704941	0.031579352
NODE_96227_length_898_cov_6.135758_g52424_i0	Control	Salt_treated	2.035342894	3.826014792	0.000209652	0.007421562
BINPACKER_5696_7	Control	Salt_treated	2.035315421	5.021834216	0.001671054	0.031138926
NODE_17332_length_2921_cov_24.325140_g8512_i1	Control	Salt_treated	2.034691037	2.679587908	0.000519002	0.014191351
NODE_12430_length_3328_cov_27.815054_g6227_i0	Control	Salt_treated	2.033154569	4.147555812	5.71477E-05	0.002970958
BINPACKER_28899_1	Control	Salt_treated	2.032443548	3.473485464	0.000382715	0.01155744
NODE_21203_length_2678_cov_20.276008_g10552_i0	Control	Salt_treated	2.029853331	3.690802063	0.000063413	0.003215272
BINPACKER_564_16	Control	Salt_treated	2.02415041	3.921428076	0.001346899	0.026763574
NODE_5750_length_4300_cov_16.023657_g2916_i0	Control	Salt_treated	2.017367733	1.951081731	0.001069115	0.023281307
Contig10332	Control	Salt_treated	2.01564936	3.586301055	0.000106438	0.004720729
BINPACKER_17283_4	Control	Salt_treated	2.01408624	2.174733703	0.001128584	0.024115997
BINPACKER_1077_1	Control	Salt_treated	2.01400688	2.186703324	0.000495109	0.013776514
BINPACKER_14106_5	Control	Salt_treated	2.01389692	1.895977205	0.002818247	0.044177632
NODE_18395_length_2849_cov_30.739553_g9158_i0	Control	Salt_treated	2.013837232	2.476213241	0.000444903	0.01284962
BINPACKER_3609_3	Control	Salt_treated	2.013653999	2.369308265	0.001479852	0.02861465
BINPACKER_10091_1	Control	Salt_treated	2.012647202	2.603110805	0.000900888	0.020762885
Contig11653	Control	Salt_treated	2.012081693	6.565071161	0.002372509	0.03957715
NODE_41785_length_1882_cov_29.715865_g20890_i0	Control	Salt_treated	2.009823639	3.652281369	0.000279445	0.009175756
Contig5124	Control	Salt_treated	2.005940214	7.606884184	0.001136089	0.024200811
NODE_47518_length_1735_cov_17.422383_g23816_i0	Control	Salt_treated	-2.0013601	2.458832204	0.000549431	0.014806508
NODE_95072_length_911_cov_28.324582_g25541_i2	Control	Salt_treated	-2.002951903	0.464241531	0.002984858	0.045947576
NODE_232_length_10069_cov_14.862645_g123_i0	Control	Salt_treated	-2.004817035	2.223411398	0.000390665	0.011694335
Contig729	Control	Salt_treated	-2.00631305	5.202043364	0.000696339	0.017502236
BINPACKER_4983_2	Control	Salt_treated	-2.011049681	1.5221275	0.000736234	0.018303357
BINPACKER_30082_1	Control	Salt_treated	-2.011767292	4.898799852	0.00185696	0.033537966
BINPACKER_10852_2	Control	Salt_treated	-2.014576772	1.169867117	0.001009247	0.022410059
NODE_62053_length_1415_cov_27.519374_g31441_i0	Control	Salt_treated	-2.015186148	8.473007089	0.003050134	0.046578805
NODE_84786_length_1041_cov_28.052686_g15277_i3	Control	Salt_treated	-2.017586768	2.970527299	0.000632347	0.016359646
Contig394	Control	Salt_treated	-2.01772728	6.311043939	0.000513359	0.014074544
BINPACKER_8153_2	Control	Salt_treated	-2.018721328	1.396281848	0.000959957	0.02161483
BINPACKER_6962_12	Control	Salt_treated	-2.018866949	1.182086692	0.001750008	0.032258902
Contig2844	Control	Salt_treated	-2.025778206	4.280674346	2.07186E-05	0.001381401
NODE_35722_length_2065_cov_24.295181_g17915_i0	Control	Salt_treated	-2.02911779	2.115783631	0.000126767	0.005380735
NODE_10274_length_3546_cov_18.461273_g5193_i0	Control	Salt_treated	-2.031495586	1.570072148	0.000632718	0.016359646
Contig5706	Control	Salt_treated	-2.033164364	2.02237875	0.000647513	0.016569457
NODE_83045_length_1065_cov_27.475806_g43739_i0	Control	Salt_treated	-2.03415485	3.701784822	2.40332E-05	0.001532679
NODE_110283_length_752_cov_35.063328_g62491_i0	Control	Salt_treated	-2.03684824	5.002875202	4.32597E-05	0.0024338
BINPACKER_54987_2	Control	Salt_treated	-2.036913655	2.489137329	9.54113E-05	0.004315575
NODE_64058_length_1377_cov_16.664110_g32521_i0	Control	Salt_treated	-2.040901147	2.779373392	9.78728E-05	0.004407498
NODE_14997_length_3102_cov_29.744470_g5360_i1	Control	Salt_treated	-2.040965741	5.192763941	8.58853E-05	0.003972195
NODE_63703_length_1384_cov_8.706331_g32337_i0	Control	Salt_treated	-2.04258411	5.456003628	0.002654727	0.042589695
NODE_52430_length_1618_cov_24.841424_g3707_i2	Control	Salt_treated	-2.042697513	1.703017985	0.003190336	0.047508009
BINPACKER_417_7	Control	Salt_treated	-2.045243198	3.286977915	0.000949946	0.021507322
BINPACKER_5508_2	Control	Salt_treated	-2.055994787	4.815969841	0.003379838	0.049488876
BINPACKER_4737_2	Control	Salt_treated	-2.062719911	2.517169977	0.002094942	0.036303482
BINPACKER_597_1	Control	Salt_treated	-2.063478127	0.48491794	0.003000993	0.04609262
NODE_45924_length_1772_cov_28.754562_g23029_i0	Control	Salt_treated	-2.067785951	2.045618529	0.000112438	0.004927954
Contig7606	Control	Salt_treated	-2.073785144	2.080823068	0.000170005	0.006574468
BINPACKER_1135_1	Control	Salt_treated	-2.074424613	7.008588264	0.002436152	0.040181836
BINPACKER_21590_1	Control	Salt_treated	-2.075780696	1.594112849	0.000435927	0.012661624
NODE_110842_length_747_cov_54.991098_g62934_i0	Control	Salt_treated	-2.078161023	2.047399112	0.000125385	0.005364124
NODE_70197_length_1267_cov_8.751256_g35957_i0	Control	Salt_treated	-2.078505296	2.834089799	0.002846057	0.044443932
BINPACKER_38074_1	Control	Salt_treated	-2.078560293	2.013574628	0.003112105	0.046818373
BINPACKER_12173_1	Control	Salt_treated	-2.081822402	3.977139858	0.000176507	0.006718551
NODE_31340_length_2219_cov_28.171948_g15694_i0	Control	Salt_treated	-2.086998261	2.942933833	5.33692E-05	0.002854
BINPACKER_398_2	Control	Salt_treated	-2.087175034	5.25274034	3.82514E-05	0.002218907
NODE_47440_length_1736_cov_29.565845_g23787_i0	Control	Salt_treated	-2.090643472	4.801143019	0.00317994	0.04748471
NODE_45089_length_1794_cov_23.948867_g22600_i0	Control	Salt_treated	-2.092297179	1.456112732	0.003176106	0.04748471
NODE_64559_length_1368_cov_36.368340_g30472_i1	Control	Salt_treated	-2.100482475	1.788921841	0.000285236	0.009297338
NODE_119770_length_668_cov_29.134454_g70162_i0	Control	Salt_treated	-2.10207434	3.85291754	3.28655E-05	0.001973371
NODE_109565_length_759_cov_29.957726_g61917_i0	Control	Salt_treated	-2.103159025	1.947227428	0.00031196	0.009947371
NODE_50397_length_1666_cov_27.022599_g25280_i0	Control	Salt_treated	-2.107816574	1.5158001	0.000591683	0.015637343
NODE_41353_length_1894_cov_32.115321_g6178_i1	Control	Salt_treated	-2.10961368	1.948794156	0.001576695	0.029868474
BINPACKER_7614_2	Control	Salt_treated	-2.109739464	3.460782415	0.000331942	0.010343601
Contig6097	Control	Salt_treated	-2.110100595	1.774863535	0.000414321	0.012206726
NODE_79481_length_1116_cov_21.637584_g36586_i1	Control	Salt_treated	-2.112766799	5.096771415	0.000695594	0.017502236
Contig8863	Control	Salt_treated	-2.119538467	1.657616999	0.001256559	0.025803437
NODE_34608_length_2103_cov_21.693103_g17335_i0	Control	Salt_treated	-2.120349167	1.30092178	0.000799466	0.019336914
BINPACKER_12924_2	Control	Salt_treated	-2.120367128	3.446711241	1.19997E-05	0.000867655
NODE_58188_length_1495_cov_30.462729_g29370_i0	Control	Salt_treated	-2.127611199	4.938093162	0.000537934	0.014573187
NODE_38654_length_1970_cov_23.955720_g2841_i2	Control	Salt_treated	-2.129530276	3.35492553	0.002796776	0.044009045
NODE_107352_length_781_cov_24.276836_g60212_i0	Control	Salt_treated	-2.129783593	1.606752981	0.000158931	0.006280747
NODE_27657_length_2360_cov_17.018802_g13869_i0	Control	Salt_treated	-2.13269609	0.633645031	0.001315161	0.026477279
Contig8767	Control	Salt_treated	-2.142375745	1.66380875	0.002740892	0.043429177
NODE_61114_length_1434_cov_32.070536_g20412_i1	Control	Salt_treated	-2.142657685	2.955586234	3.89522E-05	0.00224057
BINPACKER_7228_2	Control	Salt_treated	-2.144861129	2.142627323	0.000776408	0.018890447
NODE_7279_length_3984_cov_30.917924_g3679_i0	Control	Salt_treated	-2.146518301	2.970344097	0.00002291	0.001489307
Contig828	Control	Salt_treated	-2.149981845	2.266937446	3.96599E-05	0.002268569
NODE_32181_length_2190_cov_26.419934_g16107_i0	Control	Salt_treated	-2.154119358	5.018124212	0.001592852	0.030063616
Contig11086	Control	Salt_treated	-2.154429329	5.828960233	0.00064344	0.01651631

BINPACKER_4115_1	Control	Salt_treated	-2.15797237	3.123448739	0.000663829	0.016912801
Contig8570	Control	Salt_treated	-2.159062138	4.127622998	0.002497205	0.040801195
NODE_66698_length_1329_cov_7.877389_g33977_i0	Control	Salt_treated	-2.161482035	2.630368644	0.000436555	0.012661954
BINPACKER_10546_1	Control	Salt_treated	-2.166461975	2.700560426	5.75261E-05	0.002983079
BINPACKER_1245_1	Control	Salt_treated	-2.168742139	7.173399674	0.000166597	0.006516307
NODE_18569_length_2839_cov_30.597252_g9251_i0	Control	Salt_treated	-2.169302917	5.025479641	8.00822E-05	0.00378168
NODE_46020_length_1769_cov_28.435731_g9659_i2	Control	Salt_treated	-2.169404362	4.077470678	0.000127035	0.005380735
NODE_8657_length_3761_cov_32.234273_g4356_i0	Control	Salt_treated	-2.170715926	6.030734345	0.002809574	0.044109027
NODE_25889_length_2436_cov_23.068134_g10358_i1	Control	Salt_treated	-2.176730844	5.668921945	3.55119E-05	0.002083537
NODE_52859_length_1608_cov_24.477524_g26563_i0	Control	Salt_treated	-2.177224901	2.181062247	0.000148209	0.00595593
BINPACKER_46087_1	Control	Salt_treated	-2.179426512	2.760521614	0.000538794	0.014577239
NODE_61670_length_1423_cov_27.660741_g31220_i0	Control	Salt_treated	-2.182410854	3.609666342	0.000006136	0.000514295
NODE_94765_length_915_cov_17.394299_g51446_i0	Control	Salt_treated	-2.18434266	2.301061568	0.00160017	0.030156667
NODE_16537_length_2985_cov_28.642170_g8200_i0	Control	Salt_treated	-2.184840794	0.603267769	0.002461966	0.040477557
NODE_76562_length_1161_cov_20.198529_g39741_i0	Control	Salt_treated	-2.185809178	1.598042574	0.000308148	0.00988014
NODE_28627_length_2320_cov_28.482866_g12058_i3	Control	Salt_treated	-2.190103579	4.39215063	0.000308409	0.00988014
Contig9974	Control	Salt_treated	-2.192895496	1.860079116	0.003365559	0.049436157
NODE_72412_length_1229_cov_23.654844_g2407_i2	Control	Salt_treated	-2.196054943	4.008873904	0.001064868	0.023250736
NODE_113402_length_723_cov_34.736923_g64977_i0	Control	Salt_treated	-2.196828445	4.676011866	5.08789E-06	0.000446495
Contig5791	Control	Salt_treated	-2.197304787	5.827212607	0.002411914	0.039953714
NODE_71485_length_1244_cov_20.910333_g36718_i0	Control	Salt_treated	-2.198562223	1.414182954	0.00269253	0.043061606
BINPACKER_1545_5	Control	Salt_treated	-2.203663452	1.350942158	0.000256559	0.00863677
NODE_25033_length_2475_cov_34.869276_g12557_i0	Control	Salt_treated	-2.204349888	0.751256638	0.001076899	0.023425985
NODE_26422_length_2412_cov_21.501069_g13243_i0	Control	Salt_treated	-2.204746045	3.256647044	2.24496E-05	0.001472854
NODE_37103_length_2019_cov_30.955293_g18608_i0	Control	Salt_treated	-2.205532227	2.080838113	0.002970396	0.045802583
BINPACKER_3408_25	Control	Salt_treated	-2.205774005	1.900658945	0.001758141	0.03232046
Contig3451	Control	Salt_treated	-2.20756489	3.293576907	0.003381191	0.049488876
NODE_191087_length_337_cov_35.143939_g135484_i0	Control	Salt_treated	-2.210672622	2.387591564	0.000747687	0.018476229
NODE_12121_length_3358_cov_22.675799_g6071_i0	Control	Salt_treated	-2.212104976	4.660171254	6.3265E-06	0.000523661
BINPACKER_10726_3	Control	Salt_treated	-2.215284198	3.205513982	3.06614E-05	0.001873904
NODE_17211_length_2932_cov_24.168940_g8539_i0	Control	Salt_treated	-2.218254426	5.542592636	0.001351465	0.026787963
BINPACKER_4667_6	Control	Salt_treated	-2.22123603	3.991217687	0.000499265	0.013835903
BINPACKER_3187_13	Control	Salt_treated	-2.223746503	1.870470953	0.000425559	0.012484085
Contig3846	Control	Salt_treated	-2.227682239	2.845537962	0.001055215	0.023101102
BINPACKER_14118_2	Control	Salt_treated	-2.233414022	5.349221751	0.00060663	0.015950253
NODE_31555_length_2211_cov_24.538354_g8207_i1	Control	Salt_treated	-2.236271106	7.202689732	0.000619919	0.016154858
NODE_34873_length_2094_cov_24.661059_g17471_i0	Control	Salt_treated	-2.237188881	4.889865624	0.000348488	0.010809982
BINPACKER_3944_2	Control	Salt_treated	-2.237429437	3.416047617	0.002129255	0.036650667
NODE_34935_length_2092_cov_37.271421_g17499_i0	Control	Salt_treated	-2.241913257	2.251824183	2.79177E-05	0.001737242
Contig667	Control	Salt_treated	-2.242378007	1.015172052	0.00122538	0.02552047
BINPACKER_3720_10	Control	Salt_treated	-2.246750231	1.53256301	0.000294429	0.009530597
BINPACKER_8161_2	Control	Salt_treated	-2.247547244	5.846982853	0.00259517	0.041962065
NODE_22104_length_2627_cov_32.435395_g8026_i1	Control	Salt_treated	-2.253420234	4.846756132	0.000855334	0.020203779
NODE_48183_length_1719_cov_26.074119_g21717_i1	Control	Salt_treated	-2.257256061	0.98063342	0.002920457	0.04515932
NODE_29826_length_2276_cov_33.517022_g14969_i0	Control	Salt_treated	-2.258520006	1.884656331	0.000317478	0.010014439
BINPACKER_9277_1	Control	Salt_treated	-2.260092471	0.889641062	0.000531297	0.01445058
NODE_62048_length_1415_cov_28.945604_g31439_i0	Control	Salt_treated	-2.262614228	0.415106893	0.001655123	0.03099936
NODE_63983_length_1378_cov_25.455172_g32479_i0	Control	Salt_treated	-2.264592243	7.243085456	8.89626E-05	0.004077783
BINPACKER_3366_5	Control	Salt_treated	-2.266943996	1.914829271	0.002667194	0.042756299
BINPACKER_156_4	Control	Salt_treated	-2.268367508	3.522912292	2.91122E-06	0.00028199
NODE_113847_length_719_cov_32.125387_g65339_i0	Control	Salt_treated	-2.269234947	0.992540177	0.001221041	0.025455912
NODE_83049_length_1065_cov_24.558468_g12028_i3	Control	Salt_treated	-2.26986088	7.035640701	0.001871975	0.03364745
BINPACKER_6138_3	Control	Salt_treated	-2.286340976	2.950865458	0.003108891	0.046818373
NODE_54432_length_1573_cov_25.584000_g27431_i0	Control	Salt_treated	-2.288715018	2.599266097	0.002187033	0.037301265
NODE_92624_length_941_cov_10.624424_g38815_i1	Control	Salt_treated	-2.290055538	5.85589683	0.000593984	0.015677957
NODE_110851_length_747_cov_33.384273_g62943_i0	Control	Salt_treated	-2.295215939	1.082634188	0.000309724	0.009906838
NODE_174726_length_383_cov_52.003226_g119827_i0	Control	Salt_treated	-2.297779558	1.778911712	6.81195E-05	0.003338505
BINPACKER_5048_1	Control	Salt_treated	-2.300316432	1.365108664	0.000127345	0.005380735
NODE_24161_length_2518_cov_28.650716_g11508_i1	Control	Salt_treated	-2.304266915	4.269422637	5.47661E-05	0.002871236
NODE_37368_length_2011_cov_26.773478_g15748_i1	Control	Salt_treated	-2.304397588	1.140997763	0.000213955	0.007510353
NODE_147656_length_490_cov_27.218225_g94538_i0	Control	Salt_treated	-2.308104022	0.820964456	0.000637722	0.016390003
NODE_87058_length_1011_cov_21.768657_g46353_i0	Control	Salt_treated	-2.309995026	0.453371846	0.00142799	0.027901456
NODE_72889_length_1221_cov_18.693380_g37535_i0	Control	Salt_treated	-2.311884121	1.361675174	0.000269897	0.008953981
NODE_44776_length_1801_cov_19.599537_g22431_i0	Control	Salt_treated	-2.31355499	0.367198742	0.002580593	0.041778094
BINPACKER_6061_8	Control	Salt_treated	-2.317653106	5.658426399	0.000710904	0.01780294
NODE_47086_length_1745_cov_24.009569_g23611_i0	Control	Salt_treated	-2.320573025	3.405591106	1.74912E-06	0.000191054
Contig8396	Control	Salt_treated	-2.326593664	4.096751018	0.0005545	0.014873328
NODE_71918_length_1237_cov_15.542955_g15548_i2	Control	Salt_treated	-2.333277501	3.692617833	5.10111E-05	0.00274938
BINPACKER_11561_2	Control	Salt_treated	-2.33489967	0.477941515	0.001104286	0.023819866
NODE_56919_length_1522_cov_26.035197_g28692_i0	Control	Salt_treated	-2.336134778	3.420981373	9.08386E-06	0.000683286
NODE_21257_length_2674_cov_25.630911_g10579_i1	Control	Salt_treated	-2.341554202	3.136186813	0.00020642	0.007384731
NODE_143677_length_510_cov_37.386728_g90905_i0	Control	Salt_treated	-2.344740955	1.571381693	0.002036	0.035643023
BINPACKER_36717_1	Control	Salt_treated	-2.349818451	4.566198213	4.38923E-05	0.002449262
NODE_60562_length_1445_cov_26.011662_g11698_i2	Control	Salt_treated	-2.352587547	5.421296079	0.001230451	0.025548333
NODE_41834_length_1881_cov_25.023783_g20892_i1	Control	Salt_treated	-2.35418356	2.588098834	6.50805E-05	0.003249915
BINPACKER_23437_2	Control	Salt_treated	-2.355187341	1.573649275	5.07011E-05	0.002747091
BINPACKER_42718_2	Control	Salt_treated	-2.35753187	1.580921364	0.000491295	0.013726182
BINPACKER_3944_5	Control	Salt_treated	-2.357614117	2.88865902	0.001201794	0.025222508
NODE_65343_length_1353_cov_23.025000_g17471_i2	Control	Salt_treated	-2.364343513	3.786485896	6.34972E-06	0.000523661
Contig7584	Control	Salt_treated	-2.368075977	2.590705016	0.00056488	0.015084268
BINPACKER_643_1	Control	Salt_treated	-2.371048914	3.572953161	1.9063E-06	0.000204952
BINPACKER_30762_1	Control	Salt_treated	-2.374229292	3.255373432	0.001534754	0.029372016
NODE_134364_length_565_cov_3.617886_g46378_i1	Control	Salt_treated	-2.377758681	0.402970642	0.001338847	0.026699205
BINPACKER_10961_4	Control	Salt_treated	-2.378186305	3.799071725	5.47955E-05	0.002871236
BINPACKER_7722_2	Control	Salt_treated	-2.385018659	6.858383334	0.00150136	0.028948764
Contig11748	Control	Salt_treated	-2.394473839	7.113290549	0.000502478	0.013871656
BINPACKER_7709_6	Control	Salt_treated	-2.395639207	3.168200419	6.45296E-05	0.003247832
BINPACKER_3925_1	Control	Salt_treated	-2.395677225	3.348795362	9.40712E-05	0.004283266
NODE_21110_length_2683_cov_27.647126_g8590_i1	Control	Salt_treated	-2.400656808	2.066554674	0.001202476	0.025222508
BINPACKER_283_11	Control	Salt_treated	-2.403408583	5.846922078	6.27048E-06	0.000523433
Contig914	Control	Salt_treated	-2.408400057	6.857095791	0.000431056	0.012573491
BINPACKER_18276_3	Control	Salt_treated	-2.408636609	4.436343938	0.000254402	0.008592333
NODE_102423_length_831_cov_26.457784_g56668_i0	Control	Salt_treated	-2.410349227	2.629506603	0.00011255	0.004927954
NODE_7947_length_3871_cov_27.219063_g4014_i0	Control	Salt_treated	-2.418941114	1.26792336	0.000447036	0.012875007
BINPACKER_20522_2	Control	Salt_treated	-2.42416541	2.415425865	0.000174996	0.006716882
NODE_77619_length_1144_cov_32.563959_g40399_i0	Control	Salt_treated	-2.425226492	2.768799975	6.68758E-05	0.003309143
NODE_30235_length_2261_cov_25.842322_g15169_i0	Control	Salt_treated	-2.430467891	2.342157541	0.000137957	0.005654591
NODE_87937_length_1000_cov_3.678533_g46913_i0	Control	Salt_treated	-2.4335643	5.724869291	4.73182E-06	0.000418827
BINPACKER_18746_1	Control	Salt_treated	-2.434085702	1.363965737	6.64253E-05	0.003302768
NODE_63816_length_1381_cov_30.732416_g14061_i3	Control	Salt_treated	-2.434651475	5.990279565	9.93652E-05	0.004445456
NODE_81378_length_1088_cov_16.057143_g42694_i0	Control	Salt_treated	-2.438013381	4.691420744	7.06137E-05	0.00342802
NODE_9216_length_3683_cov_28.312188_g4652_i0	Control	Salt_treated	-2.439328736	5.6906123	1.08957E-05	0.000799085
NODE_57007_length_1520_cov_24.126469_g28749_i0	Control	Salt_treated	-2.44235605	0.869890262	0.000448568	0.012893855
Contig5792	Control	Salt_treated	-2.443077596	6.879548872	5.46115E-05	0.002871236
BINPACKER_17855_4	Control	Salt_treated	-2.443498995	4.636787005	0.000083371	0.003908728
BINPACKER_25977_8	Control	Salt_treated	-2.446677329	5.568032063	0.003432621	0.049967142
Contig4536	Control	Salt_treated	-2.452405925	2.611154071	9.43342E-06	0.000701867
NODE_47868_length_1727_cov_28.326481_g23850_i1	Control	Salt_treated	-2.456707018	2.890731278	7.62847E-06	0.00060239
BINPACKER_16086_2	Control	Salt_treated	-2.458323096	4.344797863	0.00247599	0.040578183
BINPACKER_21805_1	Control	Salt_treated	-2.459588826	1.694588943	1.93404E-05	0.001313086
BINPACKER_2187_1	Control	Salt_treated	-2.462004863	5.522451051	3.3805E-06	0.000316979
NODE_53601_length_1591_cov_41.193676_g26984_i0	Control	Salt_treated	-2.46223749	2.251799037	0.000209361	0.007421562
NODE_19220_length_2798_cov_26.666789_g9581_i0	Control	Salt_treated	-2.463707878	0.813979712	0.000886587	0.020618419

BINPACKER_5868_3	Control	Salt_treated	-2.469992864	2.042600062	0.000165212	0.00647449
BINPACKER_5163_10	Control	Salt_treated	-2.470674717	2.320935089	2.89928E-05	0.001798695
BINPACKER_15166_1	Control	Salt_treated	-2.471582256	0.603937672	0.001705454	0.031579352
BINPACKER_11342_2	Control	Salt_treated	-2.474298876	3.842823775	8.6512E-06	0.000661077
NODE_99571_length_859_cov_50.895674_g54753_i0	Control	Salt_treated	-2.475933985	3.649672127	0.001803323	0.032887422
BINPACKER_1285_2	Control	Salt_treated	-2.476942813	5.344796633	0.000331016	0.010335921
NODE_27441_length_2369_cov_25.359756_g13759_i0	Control	Salt_treated	-2.48200383	1.255604648	0.000228477	0.007938721
BINPACKER_156_2	Control	Salt_treated	-2.482858697	2.194437122	3.55835E-06	0.000329147
NODE_17649_length_2896_cov_26.366631_g8767_i0	Control	Salt_treated	-2.492565347	0.902904181	0.000745243	0.018457194
BINPACKER_1095_3	Control	Salt_treated	-2.49303303	2.724304051	4.42584E-06	0.000396876
Contig8714	Control	Salt_treated	-2.495555021	0.458658568	0.000488796	0.013693627
BINPACKER_25028_2	Control	Salt_treated	-2.496790172	1.006838712	0.000246031	0.008378532
Contig9801	Control	Salt_treated	-2.49821471	4.742999108	0.001113493	0.023843137
NODE_61165_length_1433_cov_46.559559_g30935_i0	Control	Salt_treated	-2.49897023	3.656177496	0.000198738	0.007261703
NODE_28538_length_2324_cov_25.371390_g14317_i0	Control	Salt_treated	-2.499702598	2.298655093	2.47393E-05	0.001572824
BINPACKER_25775_1	Control	Salt_treated	-2.508972666	0.863713539	0.000200757	0.007286064
BINPACKER_2375_2	Control	Salt_treated	-2.513927732	1.117054809	0.001962365	0.034589833
BINPACKER_11501_1	Control	Salt_treated	-2.515285753	2.366274975	0.000226635	0.007888049
NODE_134867_length_561_cov_27.514344_g83041_i0	Control	Salt_treated	-2.515323834	2.938173155	0.00151043	0.029041839
BINPACKER_14105_1	Control	Salt_treated	-2.516564257	1.534472851	2.21699E-05	0.001459161
Contig7628	Control	Salt_treated	-2.521526264	7.644438591	0.0013078	0.026393506
BINPACKER_11502_1	Control	Salt_treated	-2.52179107	4.629183407	0.001413146	0.027663439
NODE_16405_length_2994_cov_13.841835_g8146_i0	Control	Salt_treated	-2.525175763	2.712552025	1.88279E-06	0.00020349
NODE_57469_length_1510_cov_29.632568_g28978_i0	Control	Salt_treated	-2.525309597	5.551909573	0.002043573	0.035689399
BINPACKER_37631_1	Control	Salt_treated	-2.526205084	3.587883428	0.000159278	0.006280747
BINPACKER_9475_2	Control	Salt_treated	-2.526523988	1.261920848	0.000065204	0.003249915
NODE_50039_length_1675_cov_27.751561_g25105_i0	Control	Salt_treated	-2.527176791	1.286672954	0.000137517	0.005654591
NODE_72156_length_1233_cov_28.299138_g20111_i2	Control	Salt_treated	-2.528855313	1.074241139	0.000182756	0.006823451
NODE_11471_length_3426_cov_22.505219_g5737_i0	Control	Salt_treated	-2.534081924	2.274252298	9.46751E-05	0.004301224
NODE_11932_length_3378_cov_28.220272_g5979_i0	Control	Salt_treated	-2.536544818	5.150364913	0.000577953	0.015373396
NODE_58362_length_1492_cov_23.357294_g29464_i0	Control	Salt_treated	-2.538889051	2.92623143	2.36351E-05	0.001512061
Contig12498	Control	Salt_treated	-2.542514294	7.556497843	0.000180552	0.006778908
NODE_82843_length_1068_cov_32.400000_g43608_i0	Control	Salt_treated	-2.54812652	1.725075703	0.000198142	0.007252861
NODE_124392_length_632_cov_75.901610_g74075_i0	Control	Salt_treated	-2.550023171	4.298453619	2.07193E-05	0.001381401
BINPACKER_9524_1	Control	Salt_treated	-2.553938673	10.13430712	0.000403297	0.012022839
Contig5176	Control	Salt_treated	-2.55492031	1.984918483	0.000020271	0.001364801
NODE_128156_length_605_cov_3.635338_g77320_i0	Control	Salt_treated	-2.55629381	5.043448793	3.69873E-07	5.20228E-05
NODE_14499_length_3138_cov_21.821207_g7267_i0	Control	Salt_treated	-2.557699852	5.46925856	0.00012714	0.005380735
NODE_27585_length_2363_cov_23.834498_g13831_i0	Control	Salt_treated	-2.557865625	7.133720032	1.05453E-05	0.000776157
BINPACKER_16823_8	Control	Salt_treated	-2.568561823	3.140457699	1.58034E-05	0.001103822
NODE_89703_length_977_cov_18.655973_g48052_i0	Control	Salt_treated	-2.573543315	3.369430461	9.62924E-05	0.004345854
BINPACKER_41013_1	Control	Salt_treated	-2.579171768	4.373866996	8.07393E-06	0.000628023
Contig7050	Control	Salt_treated	-2.584387902	2.624165937	1.2346E-06	0.000143235
BINPACKER_18404_1	Control	Salt_treated	-2.586316649	2.267809813	0.00016979	0.006574468
NODE_6781_length_4072_cov_27.212303_g3422_i0	Control	Salt_treated	-2.600172398	2.744973279	0.003150112	0.047286223
NODE_95092_length_911_cov_22.519093_g51654_i0	Control	Salt_treated	-2.606462236	10.13552107	0.00020945	0.007421562
NODE_49912_length_1678_cov_19.519003_g25040_i0	Control	Salt_treated	-2.610477219	2.428532511	0.000147073	0.005930985
BINPACKER_2811_1	Control	Salt_treated	-2.610572858	2.089561884	0.000155912	0.006192744
BINPACKER_36224_1	Control	Salt_treated	-2.615495223	3.448689108	0.000014757	0.00104136
NODE_86474_length_1018_cov_43.617989_g45985_i0	Control	Salt_treated	-2.619583009	3.874114627	1.5913E-07	2.55291E-05
BINPACKER_2381_2	Control	Salt_treated	-2.625251362	9.951645929	0.000136342	0.005644731
NODE_65152_length_1357_cov_13.297508_g23774_i1	Control	Salt_treated	-2.625590333	4.36636677	6.84534E-08	1.28963E-05
NODE_108544_length_769_cov_33.191092_g37001_i2	Control	Salt_treated	-2.626025906	1.100957552	7.86083E-05	0.003740312
NODE_86575_length_1017_cov_26.363347_g19292_i1	Control	Salt_treated	-2.627125716	3.724104267	2.06714E-05	0.001381401
Contig5813	Control	Salt_treated	-2.637999436	2.221296191	0.003405279	0.04977198
BINPACKER_12842_5	Control	Salt_treated	-2.638415827	5.230792904	0.000368927	0.011341201
NODE_31481_length_2214_cov_32.840262_g15768_i0	Control	Salt_treated	-2.639796797	2.717880311	0.000635752	0.016380377
Contig3147	Control	Salt_treated	-2.641416503	3.039185616	0.000031835	0.00193211
NODE_101671_length_839_cov_4.065274_g56174_i0	Control	Salt_treated	-2.642173114	5.243783005	0.000368641	0.011341201
NODE_15606_length_3054_cov_24.073130_g7371_i1	Control	Salt_treated	-2.645221069	5.656116834	0.000723053	0.018019295
BINPACKER_2690_19	Control	Salt_treated	-2.655488215	2.483122207	5.91641E-06	0.000497924
NODE_35177_length_2083_cov_33.545274_g17625_i0	Control	Salt_treated	-2.657790654	3.936165191	5.81614E-08	1.12674E-05
BINPACKER_53096_2	Control	Salt_treated	-2.659544875	1.393566676	0.000974716	0.021923092
BINPACKER_3913_2	Control	Salt_treated	-2.66168786	3.114017125	0.000124741	0.005347721
BINPACKER_12202_2	Control	Salt_treated	-2.662070199	5.070948879	2.10825E-06	0.000218651
BINPACKER_6362_2	Control	Salt_treated	-2.662329847	5.085302368	0.002376133	0.039605434
BINPACKER_1481_6	Control	Salt_treated	-2.667536572	1.775935281	0.002336557	0.039073087
NODE_62827_length_1400_cov_33.107762_g29664_i1	Control	Salt_treated	-2.670977236	3.950663387	1.98895E-06	0.000210531
NODE_34914_length_2093_cov_26.572772_g3587_i2	Control	Salt_treated	-2.672428212	1.677966304	0.000380048	0.011527744
NODE_26174_length_2423_cov_24.943404_g13115_i0	Control	Salt_treated	-2.676271003	1.808271364	8.74486E-05	0.004026359
NODE_57073_length_1518_cov_36.331488_g28784_i0	Control	Salt_treated	-2.679863914	3.609816109	0.00028231	0.009216601
NODE_57651_length_1506_cov_31.495464_g29072_i0	Control	Salt_treated	-2.681394415	2.57200431	0.003405387	0.04977198
NODE_27144_length_2381_cov_29.334055_g13616_i0	Control	Salt_treated	-2.687391324	1.010824192	8.40563E-05	0.00393188
BINPACKER_42590_2	Control	Salt_treated	-2.688962739	0.259727061	0.002539632	0.041389956
BINPACKER_7060_2	Control	Salt_treated	-2.693229837	0.726362933	0.000150723	0.006021586
BINPACKER_12497_1	Control	Salt_treated	-2.693722042	1.381983119	1.54513E-05	0.001086619
NODE_79688_length_1113_cov_16.967308_g41654_i0	Control	Salt_treated	-2.694879979	4.705571569	6.01824E-05	0.003089615
BINPACKER_7327_3	Control	Salt_treated	-2.697529189	3.355639639	0.003146021	0.047259356
BINPACKER_85081_1	Control	Salt_treated	-2.697865223	2.503860785	0.000684611	0.017313402
NODE_38524_length_1974_cov_28.981063_g6234_i2	Control	Salt_treated	-2.706107722	3.353257705	0.000110027	0.00486939
BINPACKER_4728_9	Control	Salt_treated	-2.7061894	1.411377469	7.02718E-05	0.003419508
NODE_142263_length_518_cov_59.350562_g89625_i0	Control	Salt_treated	-2.710486999	3.931505109	0.002616275	0.042170494
BINPACKER_611_2	Control	Salt_treated	-2.71275799	2.680374832	2.89154E-06	0.000281411
BINPACKER_11165_1	Control	Salt_treated	-2.713877883	1.713473981	1.02711E-05	0.000758696
NODE_109276_length_762_cov_14.264151_g61686_i0	Control	Salt_treated	-2.714201684	5.767934145	0.000291412	0.009468593
NODE_39551_length_1944_cov_32.735970_g19822_i0	Control	Salt_treated	-2.715065295	4.543896014	0.000000128	2.15405E-05
BINPACKER_10495_1	Control	Salt_treated	-2.716664342	3.186685275	0.000000252	3.75006E-05
BINPACKER_9329_6	Control	Salt_treated	-2.726864522	2.524113977	5.27568E-07	7.17458E-05
Contig8081	Control	Salt_treated	-2.728073519	5.107558457	2.23289E-07	0.000033715
BINPACKER_8131_1	Control	Salt_treated	-2.734413515	3.891866411	0.000493393	0.013766068
BINPACKER_8325_5	Control	Salt_treated	-2.737668376	0.727245658	0.000556762	0.014906276
NODE_106084_length_794_cov_5.135922_g59299_i0	Control	Salt_treated	-2.741784111	2.695029845	0.001198597	0.025192625
BINPACKER_690_1	Control	Salt_treated	-2.74425395	5.081056892	6.12288E-08	1.17508E-05
NODE_21692_length_2651_cov_27.942591_g10817_i0	Control	Salt_treated	-2.746418739	1.401412771	8.46266E-05	0.003940607
BINPACKER_6570_4	Control	Salt_treated	-2.747416404	0.704666065	0.000669415	0.017033994
NODE_47356_length_1738_cov_28.883483_g23742_i0	Control	Salt_treated	-2.750205299	0.475626692	0.003438677	0.04997372
BINPACKER_379_2	Control	Salt_treated	-2.75341989	4.128976514	0.000033393	0.001993389
BINPACKER_1274_2	Control	Salt_treated	-2.759265026	3.523247044	6.2121E-08	1.18116E-05
NODE_60070_length_1454_cov_31.464156_g30350_i0	Control	Salt_treated	-2.760747676	10.78142559	0.000177002	0.006718551
BINPACKER_7614_4	Control	Salt_treated	-2.762546071	2.838230476	0.002372502	0.03957715
NODE_29011_length_2306_cov_23.732199_g14561_i0	Control	Salt_treated	-2.763054764	3.478845572	0.000000042	8.53972E-06
NODE_100018_length_855_cov_19.060102_g55046_i0	Control	Salt_treated	-2.769288362	5.559053218	1.97858E-06	0.000210519
NODE_176892_length_377_cov_7.006579_g121795_i0	Control	Salt_treated	-2.770841046	6.774258956	1.43444E-05	0.001015731
NODE_89029_length_985_cov_28.379386_g28877_i1	Control	Salt_treated	-2.775139652	2.294495116	0.000048443	0.00265983
BINPACKER_34216_4	Control	Salt_treated	-2.775294104	4.536843717	0.000202707	0.007315611
Contig240	Control	Salt_treated	-2.776340345	2.299534786	3.07932E-05	0.001876377
BINPACKER_2381_16	Control	Salt_treated	-2.778634439	9.834481929	0.00080108	0.019353149
Contig684	Control	Salt_treated	-2.778883989	3.136895257	0.00000805	0.000628023
NODE_22491_length_2607_cov_29.745462_g11238_i0	Control	Salt_treated	-2.780984273	3.324046704	0.000137781	0.005654591
NODE_47751_length_1730_cov_20.953530_g20835_i2	Control	Salt_treated	-2.782747788	3.696851259	3.53592E-05	0.002083537
BINPACKER_28247_1	Control	Salt_treated	-2.784265955	4.011294206	0.000248065	0.008433801
NODE_6784_length_4071_cov_29.583792_g3424_i0	Control	Salt_treated	-2.788390406	7.375120395	0.001308429	0.026393506
NODE_49614_length_1685_cov_24.249380_g24883_i0	Control	Salt_treated	-2.789231519	1.39505483	6.33129E-05	0.003215272

Contig7440	Control	Salt_treated	-2.792985344	0.771044091	0.002065183	0.035939433
BINPACKER_12202_7	Control	Salt_treated	-2.79473004	3.607244335	2.06086E-08	4.86434E-06
NODE_37970_length_1992_cov_23.769151_g19045_i0	Control	Salt_treated	-2.797126619	3.184576436	7.65638E-06	0.00060239
NODE_10302_length_3543_cov_33.529683_g5205_i0	Control	Salt_treated	-2.799377786	3.816407937	1.62193E-07	2.58189E-05
Contig3919	Control	Salt_treated	-2.799904537	4.041383946	5.36148E-06	0.000464549
BINPACKER_3408_7	Control	Salt_treated	-2.800038898	1.34400402	0.000178502	0.006762995
NODE_60480_length_1446_cov_32.147123_g30577_i0	Control	Salt_treated	-2.801458009	3.106217724	5.79196E-05	0.002995918
NODE_198620_length_317_cov_1.045082_g142860_i0	Control	Salt_treated	-2.818139874	2.473652068	7.35019E-06	0.000592458
NODE_108193_length_773_cov_5.520000_g60865_i0	Control	Salt_treated	-2.825300611	0.875606308	0.000918021	0.020963343
NODE_123529_length_639_cov_10.167845_g73336_i0	Control	Salt_treated	-2.826063024	4.380920627	1.38699E-07	2.31559E-05
Contig1850	Control	Salt_treated	-2.827358472	1.785922551	6.22227E-05	0.003170578
NODE_65791_length_1345_cov_18.290094_g33474_i0	Control	Salt_treated	-2.830113661	4.907568154	0.001530858	0.029324782
Contig10872	Control	Salt_treated	-2.831792463	5.174494112	8.74412E-07	0.000105006
NODE_80484_length_1101_cov_32.330739_g42133_i0	Control	Salt_treated	-2.836092492	4.035496646	9.24433E-06	0.000692818
BINPACKER_8727_5	Control	Salt_treated	-2.839442088	2.667375948	0.000189303	0.007016858
BINPACKER_6140_1	Control	Salt_treated	-2.844785593	2.14743806	1.75905E-06	0.000191123
NODE_56574_length_1529_cov_21.093407_g20752_i3	Control	Salt_treated	-2.847836375	0.920610562	0.001348485	0.026763574
BINPACKER_3885_4	Control	Salt_treated	-2.854901975	4.000149328	0.001872853	0.03364745
BINPACKER_3094_3	Control	Salt_treated	-2.858153513	3.464185729	3.68402E-07	5.20228E-05
BINPACKER_12853_2	Control	Salt_treated	-2.865276744	1.779025042	5.89635E-06	0.000497924
NODE_71414_length_1245_cov_27.168089_g36681_i0	Control	Salt_treated	-2.865838058	3.146226077	0.000000034	7.28324E-06
NODE_18844_length_2822_cov_22.405602_g9393_i0	Control	Salt_treated	-2.871275027	3.061634528	3.86997E-05	0.002238585
NODE_49538_length_1687_cov_22.351921_g24844_i0	Control	Salt_treated	-2.871984447	1.945092931	2.39344E-06	0.000239753
BINPACKER_3529_5	Control	Salt_treated	-2.878918566	4.308406777	0.002983934	0.045947576
BINPACKER_8843_1	Control	Salt_treated	-2.881128989	0.149595678	0.002633726	0.042318901
BINPACKER_23940_2	Control	Salt_treated	-2.882791964	2.370497184	7.64361E-07	9.57083E-05
NODE_16858_length_2958_cov_23.788908_g8362_i0	Control	Salt_treated	-2.887398434	1.603434508	5.66555E-05	0.002952845
BINPACKER_15729_1	Control	Salt_treated	-2.897802297	7.243574517	2.29211E-06	0.000233026
NODE_45397_length_1785_cov_28.336449_g22765_i0	Control	Salt_treated	-2.897859121	7.232891311	0.000783397	0.01901543
Contig4422	Control	Salt_treated	-2.900762539	3.727153869	7.3923E-06	0.000592458
NODE_94815_length_914_cov_34.680143_g51480_i0	Control	Salt_treated	-2.901458768	3.407135461	0.000999193	0.022326904
NODE_152016_length_469_cov_33.876263_g98547_i0	Control	Salt_treated	-2.901641108	1.238895946	0.000844695	0.020029807
NODE_892_length_7170_cov_29.649429_g481_i0	Control	Salt_treated	-2.905014846	6.278325442	0.000145892	0.00590658
NODE_74873_length_1188_cov_22.976682_g38728_i0	Control	Salt_treated	-2.905097287	2.085709902	5.88975E-06	0.000497924
BINPACKER_11352_2	Control	Salt_treated	-2.910612025	4.651970519	0.000030141	0.001847599
NODE_15964_length_3027_cov_28.523358_g7936_i0	Control	Salt_treated	-2.915067511	6.00104234	2.36363E-05	0.001512061
NODE_23067_length_2575_cov_35.130695_g1727_i4	Control	Salt_treated	-2.916055559	8.073826393	4.24529E-06	0.000382356
NODE_54893_length_1564_cov_16.075117_g27668_i0	Control	Salt_treated	-2.91645679	2.689010536	1.90404E-05	0.001298986
Contig3349	Control	Salt_treated	-2.918860419	1.854771806	1.27779E-06	0.000146589
NODE_36711_length_2032_cov_25.562532_g16828_i1	Control	Salt_treated	-2.924920826	2.124926968	6.73492E-05	0.003319577
NODE_28289_length_2334_cov_31.355595_g12058_i1	Control	Salt_treated	-2.938218567	3.222516269	7.68103E-08	1.42262E-05
NODE_31422_length_2216_cov_25.323845_g15734_i0	Control	Salt_treated	-2.938350938	1.785546139	6.4543E-07	8.28369E-05
Contig5405	Control	Salt_treated	-2.943220705	4.023083898	0.00017319	0.00667251
NODE_139199_length_535_cov_26.688312_g86736_i5	Control	Salt_treated	-2.944497061	4.87454864	8.67088E-06	0.000661077
Contig4547	Control	Salt_treated	-2.949115602	5.156119554	0.000159976	0.006293303
NODE_30577_length_2248_cov_25.819310_g15329_i0	Control	Salt_treated	-2.955021222	3.265849415	8.50834E-07	0.000103384
BINPACKER_9409_3	Control	Salt_treated	-2.957652736	3.765635623	0.000099067	0.004445456
BINPACKER_1274_1	Control	Salt_treated	-2.972889857	3.485132052	5.77892E-08	1.12674E-05
BINPACKER_6207_1	Control	Salt_treated	-2.975539616	3.982506068	2.96528E-05	0.001823112
NODE_17436_length_2912_cov_20.072561_g8651_i0	Control	Salt_treated	-2.979936449	1.857812531	6.86399E-05	0.003355999
NODE_188225_length_345_cov_5.974265_g132686_i0	Control	Salt_treated	-2.992585712	1.161765575	0.000819595	0.019562883
BINPACKER_24709_1	Control	Salt_treated	-3.000008332	4.640671047	5.17681E-05	0.002775607
NODE_23859_length_2533_cov_25.785772_g5472_i1	Control	Salt_treated	-3.000526655	1.671817325	3.50894E-06	0.000326045
BINPACKER_7098_2	Control	Salt_treated	-3.000904666	3.849774729	1.71008E-08	4.13135E-06
BINPACKER_6535_2	Control	Salt_treated	-3.021420145	3.478925363	2.50021E-05	0.001584626
NODE_33911_length_2127_cov_32.798442_g16987_i0	Control	Salt_treated	-3.025797776	4.621903073	3.23963E-08	7.00541E-06
BINPACKER_124458_1	Control	Salt_treated	-3.030071735	3.023154307	2.667E-08	6.15359E-06
NODE_20525_length_2716_cov_28.552781_g10228_i0	Control	Salt_treated	-3.032837288	3.35186508	0.000212537	0.007499042
NODE_54225_length_1577_cov_36.410239_g27333_i0	Control	Salt_treated	-3.038416942	3.166934169	0.00000022	3.34684E-05
NODE_56899_length_1522_cov_41.382333_g28686_i0	Control	Salt_treated	-3.041497492	1.664075638	9.2942E-07	0.000110322
NODE_111470_length_741_cov_37.540419_g63443_i0	Control	Salt_treated	-3.04236685	1.318213802	8.28586E-06	0.000642076
NODE_64323_length_1372_cov_27.213241_g32463_i1	Control	Salt_treated	-3.052192903	0.729209411	0.000158114	0.006268107
NODE_49130_length_1697_cov_19.546798_g24637_i0	Control	Salt_treated	-3.054256425	2.188536327	0.000176983	0.006718551
BINPACKER_24495_2	Control	Salt_treated	-3.054294925	4.656721817	2.83113E-06	0.000276844
BINPACKER_6356_2	Control	Salt_treated	-3.058935707	5.935656619	0.000119963	0.005186176
BINPACKER_4223_1	Control	Salt_treated	-3.059717224	7.1558104	3.99808E-07	5.51011E-05
NODE_20457_length_2721_cov_17.584970_g10187_i0	Control	Salt_treated	-3.068846525	3.022149382	1.92374E-08	4.5935E-06
BINPACKER_129880_1	Control	Salt_treated	-3.072077516	0.937520316	0.001820927	0.03306165
Contig11166	Control	Salt_treated	-3.083041016	2.539877273	0.000231734	0.008038286
BINPACKER_637_6	Control	Salt_treated	-3.08707498	3.285095882	0.000000017	4.13135E-06
BINPACKER_3209_2	Control	Salt_treated	-3.087445654	4.394410292	6.9113E-10	2.44696E-07
Contig3300	Control	Salt_treated	-3.087500618	3.853605327	8.01187E-08	1.46896E-05
BINPACKER_306_1	Control	Salt_treated	-3.095352891	1.957823665	0.001673372	0.031153842
NODE_35751_length_2064_cov_27.315922_g17928_i0	Control	Salt_treated	-3.100293509	8.519547049	3.48657E-05	0.002063303
NODE_51286_length_1645_cov_20.795165_g25737_i0	Control	Salt_treated	-3.103277864	5.6281492	4.06585E-05	0.002310874
BINPACKER_4485_2	Control	Salt_treated	-3.105138744	3.851162438	0.002631274	0.042312619
Contig7932	Control	Salt_treated	-3.11401798	2.108267422	1.28025E-05	0.000916024
NODE_114387_length_714_cov_32.798752_g54079_i1	Control	Salt_treated	-3.124590952	2.486137915	0.002503582	0.040867287
BINPACKER_4260_2	Control	Salt_treated	-3.125436649	7.787748083	3.31382E-06	0.000312153
NODE_88983_length_986_cov_15.939759_g39644_i1	Control	Salt_treated	-3.13198692	0.743825227	0.000019375	0.001313086
NODE_47023_length_1746_cov_27.377764_g23584_i0	Control	Salt_treated	-3.150587645	2.898904326	0.001812878	0.033003054
NODE_53928_length_1584_cov_32.868961_g20729_i1	Control	Salt_treated	-3.151478537	5.38270204	5.60309E-07	7.49262E-05
BINPACKER_4036_2	Control	Salt_treated	-3.172683347	1.282424599	0.000500055	0.013839128
NODE_60352_length_1449_cov_21.232558_g30510_i0	Control	Salt_treated	-3.180309393	2.382235318	6.08729E-07	7.96194E-05
NODE_69714_length_1275_cov_27.022463_g35672_i0	Control	Salt_treated	-3.183825883	0.788540835	0.000213608	0.007510353
BINPACKER_58955_1	Control	Salt_treated	-3.189731511	1.723933777	0.000381288	0.011548306
NODE_7179_length_4001_cov_26.627546_g3631_i0	Control	Salt_treated	-3.190687474	1.567267515	5.26963E-06	0.000458525
NODE_72584_length_1226_cov_24.276670_g37348_i0	Control	Salt_treated	-3.213709551	1.124704333	5.17389E-06	0.00045211
BINPACKER_25762_1	Control	Salt_treated	-3.216024939	3.156764918	3.24253E-06	0.000308266
NODE_76055_length_1169_cov_32.126825_g39447_i0	Control	Salt_treated	-3.224456874	1.326395387	1.81516E-05	0.001250814
BINPACKER_1792_1	Control	Salt_treated	-3.224507971	2.03643114	2.29225E-06	0.000233026
BINPACKER_8818_3	Control	Salt_treated	-3.241229417	1.202530585	1.73907E-05	0.00120648
NODE_38943_length_1962_cov_25.088936_g19516_i0	Control	Salt_treated	-3.245947315	1.805057988	0.000280166	0.009175756
NODE_34560_length_2105_cov_15.055610_g17315_i0	Control	Salt_treated	-3.268718219	6.157582097	1.51081E-08	3.73788E-06
BINPACKER_14_1	Control	Salt_treated	-3.273926928	7.981785403	0.001880753	0.033759842
BINPACKER_42406_1	Control	Salt_treated	-3.273968314	2.987075389	8.31891E-07	0.000102293
NODE_25259_length_2465_cov_27.476171_g3153_i3	Control	Salt_treated	-3.287969725	1.331073177	1.18067E-06	0.000137756
BINPACKER_6832_1	Control	Salt_treated	-3.297811496	4.609896452	3.46347E-08	7.33221E-06
BINPACKER_17823_1	Control	Salt_treated	-3.318895565	3.420343928	0.000000311	4.43499E-05
BINPACKER_11502_9	Control	Salt_treated	-3.323019874	3.708862305	2.2295E-08	5.20258E-06
BINPACKER_2663_6	Control	Salt_treated	-3.368603238	5.223448411	2.91966E-05	0.001800645
BINPACKER_3591_9	Control	Salt_treated	-3.379151605	5.811123278	0.000022918	0.001489307
NODE_167755_length_406_cov_6.375375_g113308_i0	Control	Salt_treated	-3.395758005	2.712759297	0.002913715	0.045105868
NODE_146614_length_495_cov_60.220379_g93588_i0	Control	Salt_treated	-3.401320873	1.549273844	0.000208196	0.007409526
BINPACKER_12853_5	Control	Salt_treated	-3.408738025	1.967637148	3.74472E-07	5.23114E-05
Contig5610	Control	Salt_treated	-3.439705484	1.185880334	0.001175687	0.024820621
BINPACKER_3668_1	Control	Salt_treated	-3.442085979	1.777451572	3.46437E-05	0.002056096
BINPACKER_28439_3	Control	Salt_treated	-3.461895984	5.875957878	8.13526E-09	2.16958E-06
NODE_203535_length_304_cov_3.220779_g147727_i0	Control	Salt_treated	-3.469840432	3.563434013	9.27889E-05	0.004235706
NODE_93922_length_925_cov_17.579812_g50871_i0	Control	Salt_treated	-3.477213436	1.579103724	3.60755E-08	7.48294E-06
NODE_38670_length_1969_cov_29.207278_g19394_i0	Control	Salt_treated	-3.480326603	2.077325173	7.18072E-06	0.000582843
BINPACKER_17244_3	Control	Salt_treated	-3.490829392	1.698594848	0.000005752	0.000492157
NODE_61829_length_1420_cov_22.960653_g31313_i0	Control	Salt_treated	-3.493691114	4.972944517	0.003441093	0.04997372

NODE_158850_length_440_cov_8.337875_g104912_i0	Control	Salt_treated	-3.504347312	3.733483639	0.003044224	0.046578805
NODE_31465_length_2215_cov_19.914099_g1986_i6	Control	Salt_treated	-3.505104445	6.119471889	3.54691E-05	0.002083537
NODE_112469_length_732_cov_10.962064_g64246_i0	Control	Salt_treated	-3.509999299	3.023143156	0.000058276	0.003006778
NODE_42388_length_1866_cov_22.403793_g21179_i0	Control	Salt_treated	-3.517889375	6.188955899	1.90283E-05	0.001298986
BINPACKER_1395_2	Control	Salt_treated	-3.543137543	4.789782201	0.000554574	0.014873328
NODE_32522_length_2177_cov_29.379278_g16272_i0	Control	Salt_treated	-3.544699782	5.629978878	2.03687E-06	0.000214498
Contig5271	Control	Salt_treated	-3.562493603	2.517475949	0.000503662	0.013871656
NODE_21771_length_2647_cov_22.940559_g10853_i0	Control	Salt_treated	-3.570953727	3.945290106	1.00227E-05	0.000743019
NODE_35950_length_2058_cov_20.484635_g7548_i2	Control	Salt_treated	-3.578068458	2.397326768	1.41128E-09	4.39102E-07
NODE_116312_length_698_cov_4.598400_g67309_i0	Control	Salt_treated	-3.606540118	4.781282458	0.002865705	0.044648902
NODE_4177_length_4756_cov_27.926543_g2112_i0	Control	Salt_treated	-3.609398302	6.068883267	4.38088E-05	0.002449262
BINPACKER_4402_5	Control	Salt_treated	-3.617203903	1.494302054	0.002170528	0.037121842
Contig249	Control	Salt_treated	-3.617225935	1.854748377	4.38662E-05	0.002449262
Contig7249	Control	Salt_treated	-3.618625313	3.053620145	0.000590429	0.015624289
BINPACKER_4908_5	Control	Salt_treated	-3.623395049	2.981660298	6.29445E-07	0.000081808
NODE_200177_length_312_cov_7.573222_g144390_i0	Control	Salt_treated	-3.630599742	2.404532528	8.01086E-05	0.00378168
BINPACKER_2404_5	Control	Salt_treated	-3.631164534	1.516119781	0.000118135	0.005128747
Contig151	Control	Salt_treated	-3.64870584	3.9914137	0.0007413	0.018406997
NODE_59828_length_1459_cov_37.405483_g30221_i0	Control	Salt_treated	-3.65333493	2.736992036	0.000173126	0.00667251
NODE_28745_length_2316_cov_23.781097_g14432_i0	Control	Salt_treated	-3.667117811	4.044369823	1.3548E-06	0.000153706
BINPACKER_18308_1	Control	Salt_treated	-3.67073427	1.490083268	7.18088E-06	0.000582843
NODE_71884_length_1237_cov_27.557560_g11942_i9	Control	Salt_treated	-3.674627558	3.144974029	6.08908E-05	0.003110432
BINPACKER_9333_2	Control	Salt_treated	-3.685095641	2.223631014	5.04505E-05	0.002746118
NODE_130273_length_590_cov_18.421663_g79140_i0	Control	Salt_treated	-3.701873086	2.011103681	0.001281466	0.026054367
Contig6925	Control	Salt_treated	-3.702801162	7.891570516	0.00344363	0.049975223
NODE_69846_length_1273_cov_29.374167_g35746_i0	Control	Salt_treated	-3.709186646	5.850786745	0.00000416	0.000376975
NODE_37914_length_1994_cov_21.561166_g19016_i0	Control	Salt_treated	-3.732486855	5.237216758	2.54284E-05	0.001606684
NODE_98269_length_874_cov_18.813983_g53856_i0	Control	Salt_treated	-3.75742063	9.836008653	0.002385419	0.039727957
BINPACKER_3473_1	Control	Salt_treated	-3.760154834	5.346965869	5.51817E-07	7.45498E-05
NODE_46861_length_1750_cov_13.169946_g16272_i1	Control	Salt_treated	-3.761690318	4.084912173	1.73877E-06	0.000190939
BINPACKER_1285_1	Control	Salt_treated	-3.764522868	7.227535888	3.02496E-07	4.34389E-05
NODE_53437_length_1595_cov_27.736531_g22454_i1	Control	Salt_treated	-3.764728369	5.299702301	9.33694E-08	1.65288E-05
BINPACKER_380_1	Control	Salt_treated	-3.767323151	2.899657586	1.70348E-07	2.69084E-05
NODE_16415_length_2993_cov_23.076370_g7297_i2	Control	Salt_treated	-3.768546639	3.438228716	3.44518E-11	1.81402E-08
NODE_67703_length_1311_cov_22.067044_g34507_i0	Control	Salt_treated	-3.769912077	4.442783593	0.000467057	0.013246925
NODE_29172_length_2300_cov_26.084868_g14640_i0	Control	Salt_treated	-3.779980845	4.499348526	6.42306E-07	8.28369E-05
BINPACKER_4950_1	Control	Salt_treated	-3.781901135	2.008200598	0.002912834	0.045105868
NODE_204457_length_301_cov_11.877193_g148647_i0	Control	Salt_treated	-3.788538943	1.866908678	0.001522213	0.029186404
NODE_42827_length_1854_cov_26.382931_g21395_i0	Control	Salt_treated	-3.794547973	0.875517511	2.27549E-05	0.001488127
NODE_69533_length_1278_cov_22.611618_g35565_i0	Control	Salt_treated	-3.811916085	4.757336097	5.05494E-05	0.002746118
NODE_55764_length_1545_cov_42.264266_g28130_i0	Control	Salt_treated	-3.816499909	5.02240714	2.75263E-05	0.001718092
BINPACKER_25738_1	Control	Salt_treated	-3.821218878	4.172050617	0.000017825	0.001232444
NODE_44244_length_1815_cov_24.769805_g22151_i0	Control	Salt_treated	-3.822695825	7.917686998	5.77425E-07	7.64995E-05
BINPACKER_11_10	Control	Salt_treated	-3.849724733	2.249287967	8.48273E-06	0.00065413
NODE_72811_length_1222_cov_21.645779_g37489_i0	Control	Salt_treated	-3.851794878	1.378807834	0.00000003	6.76574E-06
NODE_93581_length_929_cov_19.301402_g50656_i0	Control	Salt_treated	-3.85973086	6.663157303	0.001486454	0.028715271
NODE_76996_length_1154_cov_14.074006_g39999_i0	Control	Salt_treated	-3.865723647	9.941484621	0.002629359	0.042312619
NODE_21978_length_2636_cov_19.176356_g10963_i0	Control	Salt_treated	-3.872437346	7.994286804	0.001518894	0.029149985
NODE_44923_length_1798_cov_8.315362_g22511_i0	Control	Salt_treated	-3.891337487	3.717960102	6.52558E-12	4.18759E-09
Contig4949	Control	Salt_treated	-3.8943537	2.637862104	5.89894E-05	0.003035957
NODE_30717_length_2242_cov_110.990779_g15397_i0	Control	Salt_treated	-3.919984339	2.111329314	0.000956154	0.02159721
NODE_150799_length_475_cov_18.395522_g97405_i0	Control	Salt_treated	-3.972355255	8.28309996	0.001840303	0.033325064
NODE_66821_length_1327_cov_6.159490_g34041_i0	Control	Salt_treated	-3.974395843	7.787829223	0.001109737	0.023837293
NODE_56050_length_1540_cov_18.354465_g28268_i0	Control	Salt_treated	-3.987547263	2.809342291	2.2176E-10	8.59214E-08
NODE_119555_length_670_cov_14.782245_g69974_i0	Control	Salt_treated	-3.995771316	6.830206916	0.002098553	0.036335405
NODE_172737_length_390_cov_2.766562_g117980_i0	Control	Salt_treated	-3.998577596	4.627662898	0.001009934	0.022410059
BINPACKER_267_1	Control	Salt_treated	-4.004280291	7.261104357	0.000919794	0.020963343
NODE_10512_length_3521_cov_28.091937_g2081_i3	Control	Salt_treated	-4.021041084	2.197790827	2.36017E-06	0.000238413
NODE_91909_length_949_cov_23.432648_g49531_i0	Control	Salt_treated	-4.025930717	9.144549219	0.001866573	0.033622273
NODE_96568_length_893_cov_23.873171_g49531_i2	Control	Salt_treated	-4.053157204	7.875583769	0.002397715	0.039835829
NODE_161543_length_430_cov_2.784314_g107430_i0	Control	Salt_treated	-4.055361658	6.95200985	0.001914211	0.034092212
NODE_40213_length_1925_cov_29.536717_g20138_i0	Control	Salt_treated	-4.056148521	2.990737523	6.96884E-07	8.83364E-05
BINPACKER_11_8	Control	Salt_treated	-4.05761777	5.883904136	0.002108035	0.036389104
BINPACKER_6848_1	Control	Salt_treated	-4.06889019	2.116091448	0.000852635	0.020192013
NODE_122211_length_649_cov_20.015625_g72228_i0	Control	Salt_treated	-4.077856301	4.119082063	0.002497546	0.040801195
BINPACKER_11845_3	Control	Salt_treated	-4.100448357	6.28507456	5.46723E-05	0.002871236
NODE_13760_length_3198_cov_22.404800_g6913_i0	Control	Salt_treated	-4.141511492	1.540460636	4.55032E-09	0.00000128
NODE_42674_length_1858_cov_31.385994_g21330_i0	Control	Salt_treated	-4.143648928	8.53100839	2.74124E-08	6.2546E-06
BINPACKER_2386_3	Control	Salt_treated	-4.162984316	2.537932173	0.000176631	0.006718551
NODE_82305_length_1075_cov_25.474052_g42727_i1	Control	Salt_treated	-4.179118818	6.276965671	7.80429E-07	9.71279E-05
NODE_68899_length_1289_cov_34.111842_g23736_i4	Control	Salt_treated	-4.183946618	1.970018803	0.001065446	0.023250736
NODE_183245_length_359_cov_2.989510_g127873_i0	Control	Salt_treated	-4.194553524	4.977337579	0.00104864	0.023055475
NODE_121486_length_655_cov_13.383162_g71608_i0	Control	Salt_treated	-4.206800209	5.255911121	0.002914161	0.045105868
NODE_117198_length_690_cov_10.706645_g68013_i0	Control	Salt_treated	-4.213959049	0.576442538	8.37749E-07	0.0001024
NODE_169983_length_399_cov_2.950920_g115395_i0	Control	Salt_treated	-4.220747996	4.451849081	1.39635E-05	0.000992179
NODE_129518_length_595_cov_10.634100_g78490_i0	Control	Salt_treated	-4.238642452	6.298802063	0.001313413	0.026468038
BINPACKER_24785_1	Control	Salt_treated	-4.241606836	7.7946665	6.77404E-11	3.09122E-08
BINPACKER_670_1	Control	Salt_treated	-4.247021585	6.375864308	2.22964E-09	6.54082E-07
NODE_117314_length_689_cov_10.972403_g68107_i0	Control	Salt_treated	-4.255823658	3.700377287	0.000379541	0.011527744
NODE_138126_length_541_cov_40.724359_g85952_i0	Control	Salt_treated	-4.266015486	3.110202298	7.7925E-06	0.00061076
NODE_37761_length_1998_cov_30.427532_g18944_i0	Control	Salt_treated	-4.277248185	3.320019854	2.56495E-05	0.00161568
NODE_90447_length_968_cov_11.822346_g48551_i0	Control	Salt_treated	-4.283658303	4.849081052	0.001939	0.034325307
NODE_46714_length_1753_cov_23.877976_g23430_i0	Control	Salt_treated	-4.288335871	5.021540924	3.16824E-09	9.16337E-07
NODE_195598_length_325_cov_7.769841_g139878_i0	Control	Salt_treated	-4.318375627	3.705979967	0.000404506	0.012022839
NODE_17911_length_2879_cov_20.718817_g8898_i0	Control	Salt_treated	-4.337114046	6.32083221	1.96512E-12	1.44121E-09
NODE_70479_length_1262_cov_15.225399_g36116_i0	Control	Salt_treated	-4.343098219	10.65231274	0.002158026	0.037021765
BINPACKER_13295_1	Control	Salt_treated	-4.347044144	2.758884781	0.00023656	0.008191828
NODE_86417_length_1019_cov_24.873150_g45941_i0	Control	Salt_treated	-4.352540249	0.596596213	0.000267914	0.008916717
NODE_17125_length_2938_cov_26.625834_g8494_i0	Control	Salt_treated	-4.371732037	6.352935808	0.002480292	0.040616264
NODE_46414_length_1760_cov_25.970362_g23273_i0	Control	Salt_treated	-4.397033809	4.428388069	1.55739E-05	0.001091505
NODE_85607_length_1031_cov_21.082463_g26079_i1	Control	Salt_treated	-4.401491365	4.384448985	6.44337E-06	0.000529259
NODE_146855_length_494_cov_24.363420_g78490_i1	Control	Salt_treated	-4.410751396	7.842427698	0.001668017	0.03111057
NODE_136099_length_554_cov_5.827443_g84118_i0	Control	Salt_treated	-4.419871987	5.289695394	0.002038009	0.035647797
NODE_100089_length_854_cov_20.641485_g55097_i0	Control	Salt_treated	-4.474219941	8.314997749	0.000297402	0.009587352
BINPACKER_5362_1	Control	Salt_treated	-4.476395027	5.194524339	7.44359E-06	0.000592458
Contig7837	Control	Salt_treated	-4.478669326	3.188732345	0.00071552	0.01789672
NODE_101691_length_838_cov_34.048366_g56190_i0	Control	Salt_treated	-4.481812638	1.837183527	4.54153E-11	2.22838E-08
NODE_119297_length_672_cov_12.719533_g69760_i0	Control	Salt_treated	-4.526056858	5.458881994	0.003381198	0.049488876
NODE_21537_length_2659_cov_22.566899_g9880_i4	Control	Salt_treated	-4.531245109	5.100270887	0.00023942	0.008274854
NODE_22334_length_2615_cov_20.896145_g10963_i1	Control	Salt_treated	-4.557133469	7.050995598	0.002573601	0.041777781
BINPACKER_1411_1	Control	Salt_treated	-4.563437061	5.228573318	0.000647932	0.016569457
NODE_152147_length_469_cov_4.257576_g98670_i0	Control	Salt_treated	-4.563784666	2.432208999	0.001289992	0.02612424
BINPACKER_17905_2	Control	Salt_treated	-4.567936476	3.676034596	0.000146119	0.00590658
NODE_17250_length_2928_cov_21.857093_g8559_i0	Control	Salt_treated	-4.571492137	8.574502623	5.13065E-05	0.002758058
BINPACKER_19_2	Control	Salt_treated	-4.574238283	4.180286706	0.002778215	0.043784068
NODE_193764_length_330_cov_24.330739_g138082_i0	Control	Salt_treated	-4.600682463	2.396103542	0.000408348	0.012114773
BINPACKER_785_3	Control	Salt_treated	-4.604473188	4.315310719	0.00104738	0.023052467
NODE_175945_length_380_cov_5.322476_g120920_i0	Control	Salt_treated	-4.60451358	2.680787861	0.000009356	0.000698636
NODE_9415_length_3655_cov_30.104690_g4757_i0	Control	Salt_treated	-4.607031598	2.979756864	3.06237E-08	6.83542E-06
NODE_120080_length_666_cov_14.549747_g70412_i0	Control	Salt_treated	-4.612586597	5.39994139	0.00053095	0.01445058
BINPACKER_10164_6	Control	Salt_treated	-4.633076679	4.322351261	8.771E-11	3.91549E-08
Contig8508	Control	Salt_treated	-4.633922355	5.784566857	1.13475E-05	0.000823397
NODE_135739_length_556_cov_12.426501_g83799_i0	Control	Salt_treated	-4.635095939	5.65616205	0.000131542	0.005501454

BINPACKER_4279_1	Control	Salt_treated	-4.653118772	0.628363693	0.001894778	0.033867101
NODE_91137_length_959_cov_12.576749_g49026_i0	Control	Salt_treated	-4.680584976	3.118234479	1.17253E-14	1.26725E-11
NODE_143629_length_511_cov_3.655251_g90862_i0	Control	Salt_treated	-4.684019823	6.60482443	1.25491E-09	0.000000409
Contig7633	Control	Salt_treated	-4.685805342	5.858565398	0.000296754	0.009581501
NODE_131024_length_585_cov_9.890625_g79801_i0	Control	Salt_treated	-4.694327327	6.301315544	0.000812197	0.019506967
BINPACKER_5267_1	Control	Salt_treated	-4.69807975	1.366972942	0.000000013	3.33885E-06
BINPACKER_4825_6	Control	Salt_treated	-4.711411706	3.468121977	1.36809E-10	5.59438E-08
BINPACKER_8283_2	Control	Salt_treated	-4.725825109	1.94806652	1.11492E-09	3.69272E-07
NODE_162001_length_428_cov_3.394366_g107865_i0	Control	Salt_treated	-4.732880968	4.489011453	0.001387583	0.027371775
NODE_63146_length_1394_cov_22.635882_g32015_i0	Control	Salt_treated	-4.746908756	6.464812574	0.000583185	0.015472484
Contig7119	Control	Salt_treated	-4.76775941	7.149374742	6.75796E-05	0.003319967
BINPACKER_180_5	Control	Salt_treated	-4.775072712	4.254985536	7.86424E-05	0.003740312
Contig10922	Control	Salt_treated	-4.783276518	6.562012385	1.23193E-05	0.000884536
NODE_111403_length_742_cov_22.579970_g9088_i5	Control	Salt_treated	-4.846186004	0.765836402	2.89177E-07	4.19981E-05
NODE_68563_length_1295_cov_26.050736_g35002_i0	Control	Salt_treated	-4.862527396	3.012808665	0.000426394	0.012490742
NODE_51521_length_1639_cov_27.054278_g25847_i0	Control	Salt_treated	-4.866718767	4.625493812	4.65921E-06	0.000414186
NODE_62177_length_1413_cov_7.950000_g31509_i0	Control	Salt_treated	-4.941202232	6.835409656	6.66699E-05	0.003306926
NODE_43075_length_1847_cov_34.612740_g21528_i0	Control	Salt_treated	-4.949478926	7.584121201	4.05978E-05	0.002310874
BINPACKER_8299_4	Control	Salt_treated	-4.977774474	1.342046255	1.17139E-10	4.90909E-08
NODE_148876_length_484_cov_19.729927_g95662_i0	Control	Salt_treated	-4.978514967	3.268231935	1.46781E-07	2.37666E-05
NODE_79868_length_1110_cov_25.926712_g41768_i0	Control	Salt_treated	-5.002327886	0.923252501	4.5349E-08	9.12981E-06
NODE_63707_length_1384_cov_4.981693_g32341_i0	Control	Salt_treated	-5.059725062	2.646659779	2.18783E-10	8.59214E-08
NODE_110331_length_752_cov_13.356406_g54921_i3	Control	Salt_treated	-5.07585645	7.356963314	0.001665053	0.031093622
BINPACKER_37217_1	Control	Salt_treated	-5.080212216	3.145550236	1.13563E-07	1.95968E-05
NODE_104197_length_812_cov_22.414073_g57933_i0	Control	Salt_treated	-5.0935675	4.614247305	0.000111473	0.004917134
NODE_126719_length_615_cov_16.721402_g76070_i0	Control	Salt_treated	-5.119811801	4.053404345	5.37823E-08	1.06194E-05
NODE_170781_length_396_cov_3.839009_g116143_i0	Control	Salt_treated	-5.154996625	6.108410847	0.000320784	0.010087739
BINPACKER_2840_5	Control	Salt_treated	-5.216552159	3.348775835	1.22776E-11	7.41528E-09
BINPACKER_84_3	Control	Salt_treated	-5.29060821	2.931155535	0.002713029	0.04324296
BINPACKER_14_11	Control	Salt_treated	-5.298558813	4.205994839	2.09482E-13	2.04843E-10
NODE_204628_length_301_cov_3.631579_g148817_i0	Control	Salt_treated	-5.317504229	3.464799583	1.24888E-07	2.11948E-05
BINPACKER_39199_1	Control	Salt_treated	-5.475306549	4.018208751	7.66719E-19	2.6241E-15
NODE_133186_length_572_cov_23.322645_g81554_i0	Control	Salt_treated	-5.487058224	2.975973063	7.92881E-13	6.78409E-10
NODE_26162_length_2424_cov_18.319864_g12701_i1	Control	Salt_treated	-5.530889362	5.209626429	3.48323E-18	7.15281E-15
Contig3195	Control	Salt_treated	-5.57449291	5.323482958	1.39973E-06	0.000157068
NODE_71646_length_1241_cov_29.677226_g36820_i0	Control	Salt_treated	-5.594289118	0.995639162	7.94013E-10	2.76357E-07
NODE_19867_length_2759_cov_21.099777_g9880_i0	Control	Salt_treated	-5.63008919	3.983656027	8.45717E-05	0.003940607
NODE_34096_length_2121_cov_27.525391_g497_i6	Control	Salt_treated	-5.650853077	2.358883763	8.59919E-13	7.06337E-10
NODE_38485_length_1976_cov_17.818182_g19286_i0	Control	Salt_treated	-5.69030864	7.639552488	1.16059E-08	3.0168E-06
NODE_78906_length_1125_cov_27.942015_g41204_i0	Control	Salt_treated	-5.786302303	4.774399509	9.55955E-08	1.67782E-05
BINPACKER_62_1	Control	Salt_treated	-5.812041208	3.00777737	0.002740348	0.043429177
BINPACKER_225_4	Control	Salt_treated	-5.874666615	3.793640574	0.000403486	0.012022839
NODE_173084_length_389_cov_2.702532_g118312_i0	Control	Salt_treated	-5.902978206	3.270333299	0.000000394	0.000054665
NODE_40819_length_1908_cov_28.392916_g20430_i0	Control	Salt_treated	-5.905381652	3.529607289	6.57541E-10	2.36888E-07
NODE_26829_length_2395_cov_26.456934_g12040_i2	Control	Salt_treated	-5.920576065	5.137470578	1.0073E-06	0.000118879
NODE_82192_length_1077_cov_18.618526_g43193_i0	Control	Salt_treated	-5.951655398	5.868637343	8.29418E-18	1.54837E-14
NODE_151940_length_470_cov_3.846348_g98473_i0	Control	Salt_treated	-6.112157305	4.650149516	5.78739E-16	7.42776E-13
BINPACKER_29308_1	Control	Salt_treated	-6.229262735	2.91440412	2.8192E-14	2.89461E-11
NODE_160406_length_434_cov_12.673130_g106370_i0	Control	Salt_treated	-6.277690366	4.012845708	4.95688E-21	2.54474E-17
BINPACKER_38833_1	Control	Salt_treated	-6.29889828	7.773154627	2.7454E-12	1.94403E-09
BINPACKER_31170_1	Control	Salt_treated	-6.404404643	3.193945911	6.00697E-11	2.86868E-08
NODE_80068_length_1107_cov_34.284333_g41882_i0	Control	Salt_treated	-6.428800533	4.376310808	6.18075E-09	1.68915E-06
NODE_113929_length_719_cov_4.821981_g65401_i0	Control	Salt_treated	-6.477516035	1.98051307	2.8497E-13	2.65994E-10
NODE_18783_length_2826_cov_15.782056_g9362_i0	Control	Salt_treated	-6.492639607	5.708488515	3.58906E-16	4.91343E-13
Contig4452	Control	Salt_treated	-6.497147849	0.950000959	2.11778E-07	3.24541E-05
NODE_99125_length_864_cov_23.309735_g54437_i0	Control	Salt_treated	-6.558259234	2.124365011	2.56394E-11	1.42299E-08
BINPACKER_14_13	Control	Salt_treated	-6.617019922	4.384277616	1.60815E-09	4.92887E-07
NODE_134037_length_567_cov_5.032389_g82310_i0	Control	Salt_treated	-6.675683087	3.434341654	1.16868E-18	2.99985E-15
NODE_68906_length_1289_cov_30.866776_g31941_i1	Control	Salt_treated	-6.715318705	2.289596038	4.07137E-10	0.000000152
NODE_17429_length_2912_cov_32.642480_g8647_i0	Control	Salt_treated	-7.094574154	8.156342334	7.11911E-17	1.16991E-13
NODE_17651_length_2896_cov_22.881686_g8768_i0	Control	Salt_treated	-7.250234469	7.026465063	9.84005E-13	7.77174E-10
NODE_121043_length_658_cov_27.635897_g71239_i0	Control	Salt_treated	-7.482974076	3.536027998	4.06008E-22	4.16868E-18
BINPACKER_36955_3	Control	Salt_treated	-7.521385089	4.33992922	1.1337E-10	4.90909E-08
NODE_184213_length_356_cov_4.586572_g128814_i0	Control	Salt_treated	-7.682830131	4.140940941	9.17288E-25	1.88365E-20
NODE_36100_length_2052_cov_30.317332_g18108_i0	Control	Salt_treated	-7.789118972	0.349321054	1.67094E-06	0.000184478
Contig3444	Control	Salt_treated	-7.866889218	4.369256539	2.46379E-11	1.40539E-08
BINPACKER_795_4	Control	Salt_treated	-7.968609823	0.602771246	1.44427E-08	3.61685E-06
BINPACKER_20779_13	Control	Salt_treated	-8.093507243	0.582883621	1.30768E-06	0.000149185
NODE_86091_length_1024_cov_20.759201_g45736_i0	Control	Salt_treated	-8.111931155	6.266253957	4.19743E-21	2.54474E-17
NODE_155891_length_452_cov_23.854881_g102146_i0	Control	Salt_treated	-8.161013858	2.716875946	4.55768E-11	2.22838E-08
NODE_95795_length_903_cov_12.528916_g52122_i0	Control	Salt_treated	-8.250252479	0.684088008	2.64492E-06	0.000262004
BINPACKER_20953_1	Control	Salt_treated	-8.411980903	0.814188492	2.94676E-06	0.000284092
BINPACKER_14303_2	Control	Salt_treated	-8.713741549	1.334005453	2.71011E-11	1.46453E-08
BINPACKER_584_16	Control	Salt_treated	-8.917477754	1.324283247	4.44785E-07	0.000060891
NODE_175916_length_380_cov_7.084691_g120895_i0	Control	Salt_treated	-9.080834577	5.241878585	1.17085E-10	4.90909E-08
NODE_149916_length_479_cov_18.741379_g96613_i0	Control	Salt_treated	-9.108743485	1.379004367	4.64339E-05	0.002570133
BINPACKER_63066_1	Control	Salt_treated	-9.326036579	1.747463076	1.81341E-07	2.84263E-05
NODE_159911_length_436_cov_5.495868_g105905_i0	Control	Salt_treated	-10.11179096	2.873512673	1.5976E-18	3.64518E-15
NODE_158292_length_442_cov_19.593496_g104384_i0	Control	Salt_treated	-10.27626056	3.136311434	9.679E-12	0.000000006
BINPACKER_20431_3	Control	Salt_treated	-10.74744024	3.587791516	7.97599E-17	1.16991E-13
BINPACKER_20431_2	Control	Salt_treated	-10.74744024	3.587791516	7.97599E-17	1.16991E-13
BINPACKER_81_4	Control	Salt_treated	-10.92472318	3.667433863	1.14793E-18	2.99985E-15
Contig5127	Control	Salt_treated	-11.8294469	4.751123702	9.31477E-08	1.65288E-05
NODE_59089_length_1476_cov_19.667142_g29841_i0	Control	Salt_treated	-12.9938115	5.908392692	5.26816E-08	1.05031E-05

TableS2b. Matrix for DEGs (after edgeR)w

	Control_root1	Control_root2	Salt_treated_root1	Salt_treated_root2
NODE_29011_length_2306_cov_23.732199_g14561_i0	3.1	2.196	17.001	14.692
BINPACKER_14303_2	0	0	6.432	6.657
NODE_20457_length_2721_cov_17.584970_g10187_i0	1.543	1.137	12.53	7.331
Contig4422	3.868	3.724	39.263	12.287
NODE_38670_length_1969_cov_29.207278_g19394_i0	0.59	0.903	11.613	3.724
BINPACKER_20431_3	0.071	0.048	58.759	21.026
Contig3349	5.817	3.556	37.533	24.633
NODE_123529_length_639_cov_10.167845_g73336_i0	26.359	25.704	220.992	107.005
BINPACKER_4223_1	29.289	22.864	234.725	152.986
NODE_10302_length_3543_cov_33.529683_g5205_i0	1.835	2.1	16.467	8.328
BINPACKER_63066_1	0	0	4.049	15.718
NODE_42674_length_1858_cov_31.385994_g21330_i0	43.233	45.157	1047.898	392.596
NODE_69846_length_1273_cov_29.374167_g35746_i0	13.219	15.764	291.5	66.009
BINPACKER_795_4	0	0	4.479	4.633
BINPACKER_14303_1	12.7	9.41	0	0
NODE_34560_length_2105_cov_15.055610_g17315_i0	14.157	12.208	110.48	110.7
BINPACKER_9329_6	2.666	1.775	15.232	10.909
BINPACKER_3473_1	9.571	9.892	196.905	50.526
BINPACKER_584_16	0	0	1.323	4.457
NODE_12121_length_3358_cov_22.675799_g6071_i0	5.681	4.82	26.399	16.979
NODE_49890_length_1678_cov_29.505296_g25025_i0	14.001	17.311	0.98	0.557
NODE_173084_length_389_cov_2.702532_g118312_i0	10.51	1.42	129.099	430.102
NODE_46861_length_1750_cov_13.169946_g16272_i1	2.958	2.744	58.647	12.727
BINPACKER_39419_2	2.787	3.069	0	0
NODE_56899_length_1522_cov_41.382333_g28686_i0	0.917	1.209	8.433	7.302
Contig4452	0	0.656	20.819	43.869
NODE_53281_length_1598_cov_35.158033_g26811_i0	60.377	48.334	10.091	9.179
NODE_155891_length_452_cov_23.854881_g102146_i0	0	0.903	74.931	192.779
NODE_2758_length_5351_cov_32.404320_g1400_i0	1.337	1.27	0	0
NODE_40213_length_1925_cov_29.536717_g20138_i0	1.209	0.74	24.494	5.748
Contig294	4.465	6.227	0.375	0.381
NODE_8241_length_3829_cov_31.342918_g3327_i1	19.092	18.658	1.251	1.026
NODE_53928_length_1584_cov_32.868961_g20729_i1	9.855	13.087	133.547	53.781
BINPACKER_7850_20	3.292	4.609	0	0
NODE_84398_length_1046_cov_30.177801_g12630_i2	9.599	4.729	0	0
BINPACKER_690_1	7.025	6.643	46.811	34.574
NODE_38485_length_1976_cov_17.818182_g19286_i0	8.078	7.635	654.774	116.535
BINPACKER_11579_11	6.456	6.246	0.231	0.293
BINPACKER_39199_1	3.306	2.25	112.584	105.392
NODE_26086_length_2427_cov_25.486831_g12875_i1	2.297	3.02	0.223	0
BINPACKER_670_1	4.992	5.373	133.491	48.503
Contig3195	1.259	2.082	19.185	112.195
BINPACKER_1095_3	1.827	1.534	7.723	8.885
NODE_87937_length_1000_cov_3.678533_g46913_i0	45.082	37.172	231.641	160.551
NODE_111403_length_742_cov_22.579970_g9088_i5	0.967	0	12.195	14.457
BINPACKER_18504_1	29.886	31.577	5.006	4.897
BINPACKER_42406_1	2.546	1.655	27.331	9.765
NODE_72790_length_1222_cov_32.975631_g37477_i0	8.903	6.089	0.478	0
BINPACKER_380_1	0.235	0.542	6.791	2.786
NODE_111470_length_741_cov_37.540419_g63443_i0	3.022	1.276	19.918	12.61
NODE_44244_length_1815_cov_24.769805_g22151_i0	38.056	35.343	735.978	227.587
BINPACKER_5267_1	0.199	0	3.132	2.141
BINPACKER_11165_1	0.398	0.632	3.794	2.258
BINPACKER_24785_1	35.041	32.22	692.993	442.037
NODE_80068_length_1107_cov_34.284333_g41882_i0	0.811	1.029	145.638	20.175
NODE_53437_length_1595_cov_27.736531_g22454_i1	7.544	7.064	141.501	42.315
NODE_79618_length_1114_cov_18.319885_g41603_i0	8.746	6.962	0	0
NODE_17911_length_2879_cov_20.718817_g8898_i0	5.966	4.392	101.21	83.721
BINPACKER_5619_12	3.84	2.082	0	0
BINPACKER_5851_2	6.862	8.556	0	0
NODE_88501_length_992_cov_23.681175_g47272_i0	6.257	4.844	0	0
NODE_49538_length_1687_cov_22.351921_g24844_i0	1.621	0.866	10.115	6.187
NODE_44923_length_1798_cov_8.315362_g22511_i0	1.6	2.497	27.116	26.715
NODE_7349_length_3974_cov_15.034863_g3367_i1	2.027	3.995	0	0
NODE_25259_length_2465_cov_27.476171_g3153_i3	0.391	0.403	4.464	2.669
NODE_56919_length_1522_cov_26.035197_g28692_i0	5.07	5.698	21.114	25.952
NODE_159911_length_436_cov_5.495868_g105905_i0	0	0	196.251	117.063
BINPACKER_9160_1	1.678	1.258	0	0
BINPACKER_7098_2	7.338	10.872	78.948	51.435
NODE_117198_length_690_cov_10.706645_g68013_i0	1.052	0.379	11.478	13.401
NODE_47086_length_1745_cov_24.009569_g23611_i0	4.373	4.507	23.975	15.542
BINPACKER_38833_1	5.105	5.042	588.005	160.551

BINPACKER_7519_3	9.436	13.135	0	0
NODE_78906_length_1125_cov_27.942015_g41204_i0	2.24	1.787	189.157	24.163
BINPACKER_4260_2	57.618	51.847	615.16	257.058
BINPACKER_15729_1	17.194	20.253	159.778	92.783
BINPACKER_12784_3	6.499	5.241	0.47	0.762
BINPACKER_12202_2	10.225	12.353	87.589	41.963
BINPACKER_27905_2	13.005	11.041	1.339	1.378
NODE_103985_length_815_cov_4.334232_g57781_i0	20.329	18.941	0	1.789
NODE_27431_length_2369_cov_31.353223_g13755_i0	123.839	122.016	8.943	15.337
NODE_9551_length_3635_cov_31.888827_g4825_i0	3.619	3.117	0.183	0.176
NODE_18783_length_2826_cov_15.782056_g9362_i0	1.138	0.463	90.761	41.641
Contig7932	3.221	3.345	39.383	12.961
NODE_39551_length_1944_cov_32.735970_g19822_i0	5.632	7.762	41.622	36.333
BINPACKER_10650_1	14.712	18.712	1.674	1.525
NODE_26162_length_2424_cov_18.319864_g12701_i1	1.081	1.546	59.755	48.62
NODE_35177_length_2083_cov_33.545274_g17625_i0	4.913	3.664	27.355	20.498
BINPACKER_11342_2	6.584	6.402	45.895	19.501
NODE_17651_length_2896_cov_22.881686_g8768_i0	1.479	0.824	265.348	63.077
NODE_175945_length_380_cov_5.322476_g120920_i0	12.856	9.248	81.563	326.821
NODE_37970_length_1992_cov_23.769151_g19045_i0	2.297	2.834	11.525	19.618
NODE_64229_length_1374_cov_15.089931_g32617_i0	33.356	23.021	3.332	3.519
BINPACKER_11_10	1.664	1.715	11.294	31.025
Contig4168	22.505	27.274	0.972	1.994
BINPACKER_12924_2	17.386	13.899	59.963	58.326
NODE_51521_length_1639_cov_27.054278_g25847_i0	1.515	2.744	104.988	12.756
BINPACKER_24495_2	8.604	8.045	97.089	30.585
BINPACKER_156_2	4.174	4.001	21.018	19.149
BINPACKER_29308_1	0	0.548	16.993	20.586
NODE_26829_length_2395_cov_26.456934_g12040_i2	1.621	0.289	94.077	15.63
NODE_28745_length_2316_cov_23.781097_g14432_i0	2.169	1.998	40.355	9.56
BINPACKER_21821_1	3.427	3.73	0.135	0
BINPACKER_23604_2	3.925	7.972	0	0
NODE_113929_length_719_cov_4.821981_g65401_i0	0.327	0.349	41.288	20.615
NODE_133186_length_572_cov_23.322645_g81554_i0	4.43	0	98.269	80.026
BINPACKER_86140_1	47.777	38.61	1.977	4.105
NODE_30577_length_2248_cov_25.819310_g15329_i0	1.813	2.214	20.309	8.387
Contig3919	5.539	7.022	27.514	47.242
NODE_61670_length_1423_cov_27.660741_g31220_i0	7.125	6.901	35.357	21.641
NODE_65343_length_1353_cov_23.025000_g17471_i2	8.675	6.601	46.835	23.284
BINPACKER_35343_2	4.928	3.117	0.478	0.323
NODE_29172_length_2300_cov_26.084868_g14640_i0	2.894	2.479	55.786	13.343
NODE_47868_length_1727_cov_28.326481_g23850_i1	3.058	2.738	19.193	9.355
NODE_72584_length_1226_cov_24.276670_g37348_i0	0.604	1.131	7.843	6.627
NODE_13760_length_3198_cov_22.404800_g6913_i0	0.192	0.211	4.272	2.317
NODE_46714_length_1753_cov_23.877976_g23430_i0	3.683	3.911	106.574	31.729
NODE_32522_length_2177_cov_29.379278_g16272_i0	7.068	7.431	125.951	32.902
Contig645	2.546	2.918	0	0
NODE_3214_length_5115_cov_36.923840_g0_i32	1.415	0.686	0	0
BINPACKER_14_11	0.597	1.282	26.12	38.767
NODE_184213_length_356_cov_4.586572_g128814_i0	3.634	3.688	764.21	591.093
BINPACKER_3826_4	21.673	14.404	1.323	2.786
NODE_95795_length_903_cov_12.528916_g52122_i0	0	0	5.962	15.219
BINPACKER_115089_1	38.924	48.604	1.132	0
NODE_169983_length_399_cov_2.950920_g115395_i0	44.762	28.622	191.461	832.873
NODE_160406_length_434_cov_12.673130_g106370_i0	3.754	5.758	407.423	256.56
NODE_18274_length_2857_cov_27.122845_g9087_i0	4.75	5.012	0.725	0.557
NODE_3307_length_5078_cov_28.543257_g1703_i0	1.891	1.847	0.048	0.088
NODE_46908_length_1749_cov_17.919451_g23527_i0	19.22	15.897	0	0
BINPACKER_3082_1	14.413	13.321	0	0
NODE_86091_length_1024_cov_20.759201_g45736_i0	1.18	1.438	459.001	206.943
BINPACKER_156_4	7.054	8.484	38.434	27.829
BINPACKER_35519_1	29.659	23.809	0	2.375
BINPACKER_2191_3	9.841	22.569	0	0
BINPACKER_2840_5	0.306	0.277	15.072	5.161
BINPACKER_81_4	0	0	42.387	64.338
BINPACKER_8299_4	0.292	0.313	10.155	7.888
NODE_86474_length_1018_cov_43.617989_g45985_i0	11	9.398	69.416	41.435
BINPACKER_1274_1	6.229	10.505	66.395	49.265
Contig8081	9.016	10.854	71.982	46.039
BINPACKER_637_6	0.889	1.366	8.999	8.182
BINPACKER_33_2	1.735	1.221	0.104	0
BINPACKER_44392_1	2.418	3.111	0.12	0
BINPACKER_13772_2	1.465	2.371	0	0
BINPACKER_20953_1	0	0	12.123	36.04
NODE_74873_length_1188_cov_22.976682_g38728_i0	1.735	2.353	19.002	8.504

BINPACKER_2690_19	1.998	1.167	11.78	6.363
NODE_93922_length_925_cov_17.579812_g50871_i0	1.778	1.185	17.105	12.639
BINPACKER_611_2	1.984	1.276	8.465	10.352
NODE_121043_length_658_cov_27.635897_g71239_i0	1.145	0	124.923	79.147
Contig96	1.216	1.703	0.128	0
NODE_22954_length_2581_cov_18.747209_g11467_i0	7.999	2.587	0	0
NODE_151940_length_470_cov_3.846348_g98473_i0	3.797	9.549	577.436	257.498
NODE_47476_length_1735_cov_30.148014_g23807_i0	4.515	2.617	0	0
NODE_16405_length_2994_cov_13.841835_g8146_i0	1.237	1.456	8.194	5.63
NODE_14157_length_3166_cov_29.543485_g7091_i0	6.89	3.815	0	0.323
NODE_88857_length_988_cov_5.486339_g47499_i0	17.898	13.369	0	0
NODE_9415_length_3655_cov_30.104690_g4757_i0	0.128	0.542	10.896	4.311
BINPACKER_22535_1	2.88	4.585	0	0
Contig10922	15.864	13.598	96.268	552.326
NODE_34096_length_2121_cov_27.525391_g497_i6	0.313	0.084	12.418	6.569
NODE_9712_length_3615_cov_22.990966_g4909_i0	1.145	1.6	0	0
NODE_12856_length_3287_cov_24.713752_g6441_i0	1.465	1.57	0.112	0
BINPACKER_11352_4	5.333	10.379	0	0
NODE_138126_length_541_cov_40.724359_g85952_i0	4.416	7.1	177.6	28.386
BINPACKER_25762_1	2.069	4.422	39.765	15.425
NODE_175916_length_380_cov_7.084691_g120895_i0	2.851	1.54	2166.017	236.296
BINPACKER_20779_13	0	0	2.216	4.545
BINPACKER_6513_4	9.016	6.667	1.235	0.792
BINPACKER_36955_3	1.059	0.283	213.26	35.453
Contig4536	1.379	2.214	9.238	8.299
NODE_100018_length_855_cov_19.060102_g55046_i0	41.917	33.219	314.072	145.889
NODE_47156_length_1743_cov_29.588623_g23647_i0	8.682	9.061	0.159	0.997
NODE_59089_length_1476_cov_19.667142_g29841_i0	0	0	329.232	13.225
NODE_71414_length_1245_cov_27.168089_g36681_i0	4.295	4.116	26.821	26.979
NODE_68906_length_1289_cov_30.866776_g31941_i1	0.277	0	25.777	6.627
NODE_134037_length_567_cov_5.032389_g82310_i0	1.507	1.077	167.812	81.141
BINPACKER_41013_1	23.622	19.398	166.178	65.247
BINPACKER_6140_1	1.657	1.468	8.879	10.791
Contig10872	16.07	12.16	124.373	57.329
NODE_60352_length_1449_cov_21.232558_g30510_i0	1.223	2.154	17.87	9.091
NODE_85607_length_1031_cov_21.082463_g26079_i1	5.724	4.097	30.296	137.092
NODE_29978_length_2271_cov_26.885805_g15050_i1	3.442	3.039	0	0
NODE_23859_length_2533_cov_25.785772_g5472_i1	0.505	0.674	5.492	3.02
BINPACKER_11034_1	7.68	5.782	0	0
BINPACKER_14_13	0.661	0.241	15.67	57.711
BINPACKER_1285_1	31.401	26.865	550.694	181.254
NODE_28289_length_2334_cov_31.355595_g12058_i1	2.147	1.829	12.259	14.604
BINPACKER_20431_2	0.071	0.048	58.759	21.026
BINPACKER_31170_1	0.882	0.235	73.831	18.797
NODE_11039_length_3470_cov_28.317044_g5534_i0	1.55	1.3	0	0
NODE_27585_length_2363_cov_23.834498_g13831_i0	37.85	31.565	202.46	158.821
NODE_143629_length_511_cov_3.655251_g90862_i0	61.223	53.279	812.105	1567.187
BINPACKER_12853_2	1.33	1.919	13.542	7.595
Contig8508	0.761	1.221	42.515	5.19
NODE_128156_length_605_cov_3.635338_g77320_i0	54.873	52.118	324.45	222.602
BINPACKER_4908_5	1.479	1.468	25.857	7.566
BINPACKER_11502_9	3.214	1.769	18.651	23.899
BINPACKER_25624_2	4.025	5.223	0	0
BINPACKER_283_11	7.623	7.274	40.937	29.354
BINPACKER_2187_1	14.136	13.712	85.158	52.198
BINPACKER_3209_2	4.053	3.369	32.592	23.401
NODE_204628_length_301_cov_3.631579_g148817_i0	35.916	24.711	376.163	1359.101
NODE_198620_length_317_cov_1.045082_g142860_i0	32.993	80.277	359.999	264.301
NODE_7365_length_3971_cov_32.382247_g3718_i0	2.027	1.354	0.128	0
BINPACKER_20779_7	2.531	2.256	0.143	0
NODE_45180_length_1791_cov_29.323050_g22645_i0	12.849	10.59	1.235	1.29
BINPACKER_2461_5	2.169	4.91	0	0
NODE_16415_length_2993_cov_23.076370_g7297_i2	0.853	1.179	15.782	9.179
NODE_31422_length_2216_cov_25.323845_g15734_i0	0.939	0.602	5.946	4.897
BINPACKER_8283_2	0.206	0.56	12.841	6.099
Contig1468	9.095	7.431	0	0
NODE_23067_length_2575_cov_35.130695_g1727_i4	53.174	43.983	428.306	232.983
BINPACKER_17823_1	0.853	0.698	10.593	3.636
NODE_40819_length_1908_cov_28.392916_g20430_i0	0.526	0.283	38.187	8.68
NODE_7179_length_4001_cov_26.627546_g3631_i0	0.135	0.439	2.909	2.111
BINPACKER_2914_2	1.6	1.727	0	0
NODE_9216_length_3683_cov_28.312188_g4652_i0	9.166	7.629	53.578	28.885
NODE_72811_length_1222_cov_21.645779_g37489_i0	0.604	0.812	9.373	9.208
BINPACKER_1841_16	6.151	10.409	0	0
BINPACKER_8827_4	4.693	4.85	0.255	0.293

BINPACKER_37217_1	1.33	1.426	74.724	14.134
NODE_26900_length_2392_cov_27.932730_g9198_i1	11.128	10.361	0	0
NODE_21771_length_2647_cov_22.940559_g10853_i0	1.685	1.865	33.333	6.51
Contig3444	0.156	0	34.513	7.097
BINPACKER_1792_1	1.195	0.59	6.026	8.328
NODE_80484_length_1101_cov_32.330739_g42133_i0	9.528	8.093	87.333	27.653
BINPACKER_10495_1	4.302	4.916	34.712	19.53
NODE_79868_length_1110_cov_25.926712_g41768_i0	0.519	0	7.859	8.709
NODE_82305_length_1075_cov_25.474052_g42727_i1	21.41	14.753	500.767	111.492
NODE_65152_length_1357_cov_13.297508_g23774_i1	10.431	9.079	56.432	49.47
BINPACKER_1138_6	6.52	1.853	0	0
NODE_99125_length_864_cov_23.309735_g54437_i0	0	0.523	37.462	13.137
NODE_28600_length_2321_cov_27.559164_g8179_i4	1.813	1.937	0	0
Contig11604	23.693	21.48	2.001	2.053
Contig684	2.802	2.684	25.139	9.179
NODE_56050_length_1540_cov_18.354465_g28268_i0	1.273	1.185	23.84	11.407
NODE_63707_length_1384_cov_4.981693_g32341_i0	0.128	1.155	24.302	13.724
NODE_32339_length_2185_cov_10.960701_g16179_i0	16.483	18.869	1.475	3.05
BINPACKER_643_1	13.596	8.959	60.784	41.758
BINPACKER_34152_1	3.285	3.887	0	0
NODE_17429_length_2912_cov_32.642480_g8647_i0	2.809	2.708	503.819	195.125
BINPACKER_18308_1	0.441	0.668	10.242	3.255
NODE_91137_length_959_cov_12.576749_g49026_i0	1.849	1.913	41.614	42.198
BINPACKER_584_15	4.302	5.217	0	0
BINPACKER_6268_1	6.62	5.391	1.172	0.704
BINPACKER_9218_2	15.949	16.631	2.551	1.378
BINPACKER_1274_2	8.618	11.998	67.566	54.631
BINPACKER_10164_6	2.382	1.492	65.494	22.257
NODE_56108_length_1539_cov_9.210778_g28293_i0	5.191	7.094	0	0
BINPACKER_5362_1	2.716	3.803	122.563	16.158
BINPACKER_2296_5	6.172	4.687	0	0
BINPACKER_3094_3	1.6	1.697	15.104	6.745
NODE_139199_length_535_cov_26.688312_g86736_i5	49.362	49.699	206.693	412.449
BINPACKER_4825_6	1.166	0.83	35.11	11.671
BINPACKER_17244_3	0.796	0.156	6.161	3.724
NODE_126719_length_615_cov_16.721402_g76070_i0	7.914	3.309	76.765	231.605
Contig9283	109.433	100.566	0	0.909
NODE_82192_length_1077_cov_18.618526_g43193_i0	3.833	4.573	213.117	236.942
NODE_54225_length_1577_cov_36.410239_g27333_i0	2.752	3.057	17.272	24.222
NODE_36100_length_2052_cov_30.317332_g18108_i0	0	0	2.048	3.548
NODE_62827_length_1400_cov_33.107762_g29664_i1	7.274	6.264	54.774	23.284
NODE_80300_length_1104_cov_19.317168_g29241_i3	8.568	7.846	0	0
Contig3300	2.147	1.877	21.664	9.267
BINPACKER_8747_1	4.195	3.105	0	0
BINPACKER_24505_4	2.389	2.244	0.159	0.235
BINPACKER_4386_3	1.436	0.975	0	0
NODE_113402_length_723_cov_34.736923_g64977_i0	38.44	36.378	177.018	123.485
Contig5127	0	0	121.878	6.217
BINPACKER_16981_1	2.944	2.599	0	0
NODE_19709_length_2767_cov_25.770973_g799_i2	1.493	1.799	0	0
BINPACKER_23940_2	4.615	2.702	28.909	18.357
NODE_35536_length_2071_cov_26.419920_g17817_i0	28.308	22.1	2.861	2.229
Contig7050	1.799	1.402	10.322	6.833
BINPACKER_12853_5	0.946	1.823	17.073	8.651
BINPACKER_20431_1	16.333	13.213	0.574	0
NODE_158292_length_442_cov_19.593496_g104384_i0	0	0	290.129	64.866
BINPACKER_34092_11	6.897	9.001	0.406	0.411
NODE_71646_length_1241_cov_29.677226_g36820_i0	0.149	0.156	7.285	8.035
BINPACKER_12202_7	4.359	3.201	24.94	21.7
NODE_35950_length_2058_cov_20.484635_g7548_i2	0.74	1.053	11.167	7.507
NODE_33911_length_2127_cov_32.798442_g16987_i0	6.236	4.26	50.215	27.066
BINPACKER_6832_1	8.604	7.383	102.207	40.966
NODE_10512_length_3521_cov_28.091937_g2081_i3	0.356	0.235	7.134	1.789
BINPACKER_28439_3	6.691	6.558	88.649	44.163
NODE_101691_length_838_cov_34.048366_g56190_i0	1.216	0.884	25.689	17.36
BINPACKER_124458_1	106.568	63.995	618.962	466.552
NODE_148876_length_484_cov_19.729927_g95662_i0	4.956	5.319	254.923	45.541

TableS2c.Blastx Results for DEGs

BINPACKER_115089_1	sp_A2WZ60_IRO2_ORYSI_Protein_IRON-RELATED_TRANSCRIPTION_FACTOR_2_OS=Oryza_sativa_subsp._indica_OX=39946_GN=IRO2_PE=3_SV=1	71.667	120	33	1	357	1	1	120	1.11E-36	129
BINPACKER_1841_16	sp_A2WZ60_IRO2_ORYSI_Protein_IRON-RELATED_TRANSCRIPTION_FACTOR_2_OS=Oryza_sativa_subsp._indica_OX=39946_GN=IRO2_PE=3_SV=1	74.18	244	54	4	1623	919	3	246	3.4E-104	318
NODE_103985_length_815_cov_4.334232_g57781_i0	sp_A2WZ60_IRO2_ORYSI_Protein_IRON-RELATED_TRANSCRIPTION_FACTOR_2_OS=Oryza_sativa_subsp._indica_OX=39946_GN=IRO2_PE=3_SV=1	76.536	179	35	3	738	223	68	246	7.24E-90	270
NODE_32522_length_2177_cov_29.379278_g16272_i0	sp_A2XFI3_PDC2_ORYSI_Pyruvate_decarboxylase_2_OS=Oryza_sativa_subsp._indica_OX=39946_GN=PDC2_PE=2_SV=2	82.394	568	96	2	1994	291	43	606	0	932
NODE_111403_length_742_cov_22.579970_g9088_i5	sp_A3BI11_ZIP8_ORYSJ_Zinc_transporter_8_OS=Oryza_sativa_subsp._japonica_OX=39947_GN=ZIP8_PE=2_SV=1	77.273	132	24	1	570	175	49	174	3.36E-58	192
NODE_53281_length_1598_cov_35.158033_g26811_i0	sp_A3C6D7_ACT2_ORYSJ_Actin-2_OS=Oryza_sativa_subsp._japonica_OX=39947_GN=ACT2_PE=2_SV=1	98.939	377	4	0	1380	250	1	377	0	778
Contig4168	sp_A5H452_PER70_MAIZE_Peroxidase_70_OS=Zea_mays_OX=4577_GN=PER70_PE=1_SV=1	65.882	170	56	1	756	253	152	321	4.12E-55	182
BINPACKER_63066_1	sp_A6YIH8_C7D55_HYOMU_Premnaspirodiene_oxygenase_OS=Hyoscyamus_muticus_OX=35626_GN=CYP71D55_PE=1_SV=1	50.316	475	229	5	168	1586	31	500	1.6E-167	488
BINPACKER_11502_9	sp_A7PZL3_PGLR_VITVI_Probable_polygalacturonase_OS=Vitis_vinifera_OX=29760_GN=GSVIVT00026920001_PE=1_SV=1	66.578	377	119	4	626	1735	111	487	0	533
BINPACKER_10495_1	sp_B5FZA8_LYRM4_TAEGU_LYR_motif-containing_protein_4_OS=Taeniopygia_guttata_OX=59729_GN=LYRM4_PE=3_SV=1	43.21	81	45	1	181	423	5	84	1.14E-09	58.5
BINPACKER_5362_1	sp_B6TB21_DRE2_MAIZE_Anamorsin_homolog_OS=Zea_mays_OX=4577_PE=2_SV=1	93.156	263	18	0	286	1074	1	263	7.1E-127	378
NODE_51521_length_1639_cov_27.054278_g25847_i0	sp_B6TB21_DRE2_MAIZE_Anamorsin_homolog_OS=Zea_mays_OX=4577_PE=2_SV=1	90.494	263	25	0	1456	668	1	263	8.2E-122	362
NODE_56108_length_1539_cov_9.210778_g28293_i0	sp_C0LGG3_Y5182_ARATH_Probable_LRR_receptor-like_serine/threonine-protein_kinase_At1g51820_OS=Arabidopsis_thaliana_OX=3702_GN=At1g51820_PE=1_SV=1	43.624	447	219	9	1	1278	443	877	2.9E-106	340
NODE_9712_length_3615_cov_22.990966_g4909_i0	sp_C0LGH2_Y1561_ARATH_Probable_LRR_receptor-like_serine/threonine-protein_kinase_At1g56130_OS=Arabidopsis_thaliana_OX=3702_GN=At1g56130_PE=2_SV=2	49.18	61	31	0	3286	3104	249	309	2.1E-08	62.8
BINPACKER_11165_1	sp_C0LGJ1_Y1743_ARATH_Probable_LRR_receptor-like_serine/threonine-protein_kinase_At1g74360_OS=Arabidopsis_thaliana_OX=3702_GN=At1g74360_PE=1_SV=1	53.704	1026	431	11	350	3388	37	1031	0	1040
NODE_47156_length_1743_cov_29.588623_g23647_i0	sp_C5X2M4_THI42_SORBI_Thiamine_thiazole_synthase_2,_chloroplastic_OS=Sorghum_bicolor_OX=4558_GN=THI1-2_PE=3_SV=1	89.645	338	31	3	1189	188	15	352	0	572
BINPACKER_5267_1	sp_C7G304_GOLS2_SOLLC_Galactinol_synthase_2_OS=Solanum_lycopersicum_OX=4081_GN=GOLS2_PE=2_SV=1	74.412	340	82	3	102	1115	1	337	8.7E-175	521
BINPACKER_2690_19	sp_D7U6G6_ANRPN_VITVI_Anthocyanidin_reductase_((2S)-flavan-3-ol-forming)_OS=Vitis_vinifera_OX=29760_GN=ANR_PE=3_SV=1	40.741	216	96	3	901	254	8	191	1.19E-43	163
BINPACKER_14303_1	sp_F4I114_Y1960_ARATH_Probable_serine/threonine-protein_kinase_At1g09600_OS=Arabidopsis_thaliana_OX=3702_GN=At1g09600_PE=3_SV=1	48.822	467	205	10	1749	382	270	713	1.4E-111	352
BINPACKER_14303_2	sp_F4I114_Y1960_ARATH_Probable_serine/threonine-protein_kinase_At1g09600_OS=Arabidopsis_thaliana_OX=3702_GN=At1g09600_PE=3_SV=1	48.608	467	206	10	1741	374	270	713	1.4E-110	349
BINPACKER_6268_1	sp_F4I893_ILA_ARATH_Protein_ILITYHIA_OS=Arabidopsis_thaliana_OX=3702_GN=ILA_PE=1_SV=1	65.563	2326	728	10	805	7737	413	2680	0	2810
BINPACKER_33_2	sp_F4IRU3_MYO12_ARATH_Myosin-12_OS=Arabidopsis_thaliana_OX=3702_GN=XI-F_PE=2_SV=1	68.059	1556	490	4	338	4996	1	1552	0	2140
NODE_26086_length_2427_cov_25.486831_g12875_i1	sp_F4JVA6_SFH6_ARATH_Phosphatidylinositol/phosphatidylcholine_transfer_protein_SFH6_OS=Arabidopsis_thaliana_OX=3702_GN=SFH6_PE=2_SV=1	47.487	577	275	15	2106	403	56	613	5.1E-150	456
Contig7050	sp_O22174_ERF08_ARATH_Ethylene-responsive_transcription_factor_ERF008_OS=Arabidopsis_thaliana_OX=3702_GN=ERF008_PE=2_SV=1	64.602	113	37	1	1856	1518	18	127	1E-39	147
NODE_47868_length_1727_cov_28.326481_g23850_i1	sp_O22174_ERF08_ARATH_Ethylene-responsive_transcription_factor_ERF008_OS=Arabidopsis_thaliana_OX=3702_GN=ERF008_PE=2_SV=1	58.519	135	44	3	1121	744	33	164	2.69E-25	106
NODE_33911_length_2127_cov_32.798442_g16987_i0	sp_O22197_RHC1A_ARATH_Probable_E3_ubiquitin-protein_ligase_RHC1A_OS=Arabidopsis_thaliana_OX=3702_GN=RHC1A_PE=2_SV=1	51.899	237	109	5	1034	1732	1	236	8.17E-72	239
BINPACKER_36955_3	sp_O22259_ERF71_ARATH_Ethylene-responsive_transcription_factor_ERF071_OS=Arabidopsis_thaliana_OX=3702_GN=ERF071_PE=2_SV=1	72.973	74	20	0	316	95	34	107	2.5E-16	77.8
Contig11604	sp_O22898_LACS1_ARATH_Long_chain_acyl-CoA_synthetase_1_OS=Arabidopsis_thaliana_OX=3702_GN=LACS1_PE=2_SV=1	63.184	201	74	0	2027	2629	393	593	5.68E-80	275
NODE_38670_length_1969_cov_29.207278_g19394_i0	sp_O24645_HCBT1_DIACA_Anthranilate_N-benzoyltransferase_protein_1_OS=Dianthus_caryophyllus_OX=3570_GN=HCBT1_PE=1_SV=1	28.997	369	215	17	454	1476	78	427	1.87E-23	107
BINPACKER_4386_3	sp_O64728_STI_ARATH_Protein_STICHEL_OS=Arabidopsis_thaliana_OX=3702_GN=STI_PE=1_SV=2	47.119	972	431	21	3366	544	267	1186	0	737
BINPACKER_643_1	sp_O64818_Y2309_ARATH_Uncharacterized_protein_At2g23090_OS=Arabidopsis_thaliana_OX=3702_GN=At2g23090_PE=1_SV=1	78.205	78	16	1	315	545	1	78	9.98E-36	126
NODE_88501_length_992_cov_23.681175_g47272_i0	sp_O64937_EF1A_ORYSJ_Elongation_factor_1-alpha_OS=Oryza_sativa_subsp._japonica_OX=39947_GN=REFA1_PE=2_SV=2	100	276	0	0	829	2	1	276	0	574
NODE_10302_length_3543_cov_33.529683_g5205_i0	sp_O65238_Y5537_ARATH_G-type_lectin_S-receptor-like_serine/threonine-protein_kinase_At5g35370_OS=Arabidopsis_thaliana_OX=3702_GN=At5g35370_PE=2_SV=2	44.953	852	400	22	3407	912	26	828	0	687
BINPACKER_17244_3	sp_O81905_SD18_ARATH_Receptor-like_serine/threonine-protein_kinase_SD1-8_OS=Arabidopsis_thaliana_OX=3702_GN=SD18_PE=1_SV=1	49.746	591	263	7	2121	412	273	850	0	595
NODE_20457_length_2721_cov_17.584970_g10187_i0	sp_O81905_SD18_ARATH_Receptor-like_serine/threonine-protein_kinase_SD1-8_OS=Arabidopsis_thaliana_OX=3702_GN=SD18_PE=1_SV=1	48.505	769	379	11	441	2720	28	788	0	716
NODE_16405_length_2994_cov_13.841835_g8146_i0	sp_O81906_B120_ARATH_G-type_lectin_S-receptor-like_serine/threonine-protein_kinase_B120_OS=Arabidopsis_thaliana_OX=3702_GN=B120_PE=2_SV=1	46.056	862	394	21	222	2762	44	849	0	699
NODE_44244_length_1815_cov_24.769805_g22151_i0	sp_P00333_ADH1_MAIZE_Alcohol_dehydrogenase_1_OS=Zea_mays_OX=4577_GN=ADH1_PE=2_SV=1	94.987	379	19	0	1360	224	1	379	0	669
NODE_28289_length_2334_cov_31.355595_g12058_i1	sp_P09189_HSP7C_PETHY_Heat_shock_cognate_70_kDa_protein_OS=Petunia_hybrida_OX=4102_GN=HSP70_PE=2_SV=1	92.912	649	44	1	115	2055	3	651	0	1132
BINPACKER_11352_4	sp_P0C0M2_GH32_ORYSJ_Probable_indole-3-acetic_acid-amido_synthetase_GH3.2_OS=Oryza_sativa_subsp._japonica_OX=39947_GN=GH3.2_PE=2_SV=1	88.455	589	59	3	2068	329	26	614	0	1073
BINPACKER_3473_1	sp_P0C540_ACT7_ORYSJ_Actin-7_OS=Oryza_sativa_subsp._japonica_OX=39947_GN=ACT7_PE=3_SV=1	95.42	262	12	0	1290	505	115	376	0	527
NODE_29172_length_2300_cov_26.084868_g14640_i0	sp_P0C540_ACT7_ORYSJ_Actin-7_OS=Oryza_sativa_subsp._japonica_OX=39947_GN=ACT7_PE=3_SV=1	85.507	414	22	1	1751	510	1	376	0	736
NODE_69846_length_1273_cov_29.374167_g35746_i0	sp_P0C540_ACT7_ORYSJ_Actin-7_OS=Oryza_sativa_subsp._japonica_OX=39947_GN=ACT7_PE=3_SV=1	90.196	357	35	0	3	1073	20	376	0	676
BINPACKER_4260_2	sp_P12783_PGKY_WHEAT_Phosphoglycerate_kinase,_cytosolic_OS=Triticum_aestivum_OX=4565_PE=2_SV=1	90.796	402	36	1	220	1425	1	401	0	729
NODE_42674_length_1858_cov_31.385994_g21330_i0	sp_P12783_PGKY_WHEAT_Phosphoglycerate_kinase,_cytosolic_OS=Triticum_aestivum_OX=4565_PE=2_SV=1	91.521	401	34	0	123	1325	1	401	0	708
NODE_3214_length_5115_cov_36.923840_g0_i32	sp_P17933_RR2_WHEAT_30S_ribosomal_protein_S2,_chloroplastic_OS=Triticum_aestivum_OX=4565_GN=rps2_PE=3_SV=2	98.246	228	4	0	977	1660	1	228	2.9E-152	468
BINPACKER_41013_1	sp_P24805_TSJT1_TOBAC_Stem-specific_protein_TSJT1_OS=Nicotiana_tabacum_OX=4097_GN=TSJT1_PE=2_SV=1	50.617	81	39	1	413	171	69	148	5.78E-23	94.7
Contig5127	sp_P25776_ORYA_ORYSJ_Oryzain_alpha_chain_OS=Oryza_sativa_subsp._japonica_OX=39947_GN=Os04g0650000_PE=1_SV=2	67.788	416	130	3	1447	203	30	442	0	532
NODE_59089_length_1476_cov_19.667142_g29841_i0	sp_P25776_ORYA_ORYSJ_Oryzain_alpha_chain_OS=Oryza_sativa_subsp._japonica_OX=39947_GN=Os04g0650000_PE=1_SV=2	65.06	249	84	2	191	934	30	276	7.3E-99	308
BINPACKER_283_11	sp_P26517_G3PC1_HORVU_Glyceraldehyde-3-phosphate_dehydrogenase_1,_cytosolic_OS=Hordeum_vulgare_OX=4513_GN=GAPC_PE=2_SV=1	94.362	337	19	0	1498	488	1	337	0	623
Contig8081	sp_P29036_FRI1_MAIZE_Ferritin-1,_chloroplastic_OS=Zea_mays_OX=4577_GN=FER1_PE=1_SV=2	87.402	254	27	3	1549	803	1	254	2.2E-146	427
NODE_111470_length_741_cov_37.540419_g63443_i0	sp_P34106_ALA2_PANMI_Alanine_aminotransferase_2_OS=Panicum_miliaceum_OX=4540_PE=1_SV=1	90.741	108	10	0	2	325	375	482	5.48E-48	168
BINPACKER_156_2	sp_P37220_ASR3_SOLLC_Abscisic_stress-ripening_protein_3_OS=Solanum_lycopersicum_OX=4081_GN=ASR3_PE=3_SV=2	73.611	72	19	0	139	354	1	72	2.93E-09	57.4
BINPACKER_156_4	sp_P37220_ASR3_SOLLC_Abscisic_stress-ripening_protein_3_OS=Solanum_lycopersicum_OX=4081_GN=ASR3_PE=3_SV=2	73.077	52	14	0	449	604	21	72	5.13E-11	62.8
BINPACKER_10650_1	sp_P37891_CBP3_ORYSJ_Serine_carboxypeptidase_3_OS=Oryza_sativa_subsp._japonica_OX=39947_GN=CBP3_PE=2_SV=1	83.836	464	67	4	1663	272	30	485	0	796
NODE_49890_length_1678_cov_29.505296_g25025_i0	sp_P37891_CBP3_ORYSJ_Serine_carboxypeptidase_3_OS=Oryza_sativa_subsp._japonica_OX=39947_GN=CBP3_PE=2_SV=1	91.045	469	40	1	1	1407	19	485	0	884
BINPACKER_37217_1	sp_P43279_MAOC_ORYSJ_NADP-dependent_malic_enzyme,_chloroplastic_OS=Oryza_sativa_subsp._japonica_OX=39947_GN=ME6_PE=2_SV=2	89.671	213	22	0	1	639	427	639	7.4E-119	358
Contig8508	sp_P43279_MAOC_ORYSJ_NADP-dependent_malic_enzyme,_chloroplastic_OS=Oryza_sativa_subsp._japonica_OX=39947_GN=ME6_PE=2_SV=2	89.231	130	14	0	4642	5031	510	639	1.79E-61	228
BINPACKER_29308_1	sp_P48979_PGLR_PRUPE_Polygalacturonase_OS=Prunus_persica_OX=3760_PE=2_SV=1	55.707	368	161	1	333	1436	28	393	3.3E-137	406
BINPACKER_7519_3	sp_P49608_ACOC_CUCMA_Aconitate_hydratase,_cytoplasmic_OS=Cucurbita_maxima_OX=3661_PE=2_SV=1	87.342	237	29	1	570	1280	2	237	2.2E-123	387
NODE_38485_length_1976_cov_17.818182_g19286_i0	sp_P52420_PUR2_ARATH_Phosphoribosylamine--glycine_ligase,_chloroplastic_OS=Arabidopsis_thaliana_OX=3702_GN=PUR2_PE=2_SV=2	77.117	437	100	0	1623	313	92	528	0	724
NODE_93922_length_925_cov_17.579812_g50871_i0	sp_P52839_SOT12_ARATH_Cytosolic_sulfotransferase_12_OS=Arabidopsis_thaliana_OX=3702_GN=SOT12_PE=1_SV=2	38.589	241	138	4	698	6	6	246	3.7E-59	195
NODE_113402_length_723_cov_34.736923_g64977_i0	sp_P52877_SERC_SPIOL_Phosphoserine_aminotransferase,_chloroplastic_OS=Spinacia_oleracea_OX=3562_PE=2_SV=1	57.432	148	59	2	436	2	42	188	1.1E-46	163
NODE_47086_length_1745_cov_24.009569_g23611_i0	sp_P54797_TNG2_MOUSE_Transport_and_Golgi_organization_2_homolog_OS=Mus_musculus_OX=10090_GN=Tango2_PE=1_SV=1	37.363	182	101	6	669	1196	32	206	9.94E-25	107
NODE_113929_length_719_cov_4.821981_g65401_i0	sp_P59082_LFS_ALLCE_Lachrymatory-factor_synthase_OS=Allium_cepa_OX=4679_GN=LFS_PE=1_SV=1	28.261	138	91	3	93	494	20	153	2.9E-14	71.6
BINPACKER_25624_2	sp_P92941_CLCA_ARATH_Chloride_channel_protein_CLC-a_OS=Arabidopsis_thaliana_OX=3702_GN=CLC-A_PE=1_SV=2	65.074	816	236	3	2964	520	8	775	0	988
NODE_14157_length_3166_cov_29.543485_g7091_i0	sp_P92941_CLCA_ARATH_Chloride_channel_protein_CLC-a_OS=Arabidopsis_thaliana_OX=3702_GN=CLC-A_PE=1_SV=2	68.34	777	236	2	321	2648	8	775	0	1006
NODE_72811_length_1222_cov_21.645779_g37489_i0	sp_Q02921_NO93_SOYBN_Early_nodulin-93_OS=Glycine_max_OX=3847_PE=2_SV=1	72.093	86	24	0	628	885	14	99	7.78E-24	98.2
NODE_27431_length_2369_cov_31.353223_g13755_i0	sp_Q05085_PTR7_ARATH_Protein_NRT1/_PTR_FAMILY_6.3_OS=Arabidopsis_thaliana_OX=3702_GN=NPF6.3_PE=1_SV=1	61.111	594	216	7	1983	226	3	589	0	655
NODE_71646_length_1241_cov_29.677226_g36820_i0	sp_Q06398_GSTU6_ORYSJ_Probable_glutathione_S-transferase_GSTU6_OS=Oryza_sativa_subsp._japonica_OX=39947_GN=GSTU6_PE=2_SV=2	63.793	116	36	2	1060	722	1	113	1.62E-40	147
NODE_17429_length_2912_cov_32.642480_g8647_i0	sp_Q06572_AVP_HORVU_Pyrophosphate-energized_vacuolar_membrane_proton_pump_OS=Hordeum_vulgare_OX=4513_PE=2_SV=2	87.831	756	90	2	194	2461	1	754	0	1285
NODE_18783_length_2826_cov_15.782056_g9362_i0	sp_Q06572_AVP_HORVU_Pyrophosphate-energized_vacuolar_membrane_proton_pump_OS=Hordeum_vulgare_OX=4513_PE=2_SV=2	88.976	762	82	2	2730	445	1	760	0	1366
BINPACKER_15729_1	sp_Q0D5W6_SUI1_ORYSJ_Protein_translation_factor_SUI1_homolog_OS=Oryza_sativa_subsp._japonica_OX=39947_GN=GOS2_PE=3_SV=1	99.13	115	1	0	3371	3027	1	115	4.44E-61	207
NODE_23067_length_2575_cov_35.130695_g1727_i4	sp_Q0D5W6_SUI1_ORYSJ_Protein_translation_factor_SUI1_homolog_OS=Oryza_sativa_subsp._japonica_OX=39947_GN=GOS2_PE=3_SV=1	99.13	115	1	0	809	465	1	115	8.48E-62	207
NODE_159911_length_436_cov_5.495868_g105905_i0	sp_Q0DHF6_PDC1_ORYSJ_Pyruvate_decarboxylase_1_OS=Oryza_sativa_subsp._japonica_OX=39947_GN=PDC1_PE=2_SV=1	93.056	144	10	0	434	3	128	271	1.98E-93	285
BINPACKER_7850_20	sp_Q0J954_PAO5_ORYSJ_Polyamine_oxidase_5_OS=Oryza_sativa_subsp._japonica_OX=39947_GN=PAO5_PE=1_SV=1	79.913	229	16	1	630	1316	12	210	1.8E-93	293
BINPACKER_3826_4	sp_Q0WP01_PTR9_ARATH_Protein_NRT1/_PTR_FAMILY_5.10_OS=Arabidopsis_thaliana_OX=3702_GN=NPF5.10_PE=2_SV=1	55.493	537	206	5	535	2058	21	553	0	554
BINPACKER_12784_3	sp_Q0WSY2_FPP4_ARATH_Filament-like_plant_protein_4_OS=Arabidopsis_thaliana_OX=3702_GN=FPP4_PE=1_SV=1	45.234	1070	470	27	265	3390	1	982	0	687
NODE_35536_length_2071_cov_26.419920_g17817_i0	sp_Q0WSY2_FPP4_ARATH_Filament-like_plant_protein_4_OS=Arabidopsis_thaliana_OX=3702_GN=FPP4_PE=1_SV=1	59.844	513	178	10	1687	191	1	499	3E-167	508
BINPACKER_35343_2	sp_Q10E64_KN8B_ORYSJ_Kinesin-like_protein_KIN-8B_OS=Oryza_sativa_subsp._japonica_OX=39947_GN=KIN8B_PE=2_SV=1	91.193	704	62	0	2591	480	1	704	0	1291
NODE_47476_length_1735_cov_30.148014_g23807_i0	sp_Q10LQ2_CIPKA_ORYSJ_CBL-interacting_protein_kinase_10_OS=Oryza_sativa_subsp._japonica_OX=39947_GN=CIPK10_PE=2_SV=1	80.357	336	61	3	740	1735	1	335	0	561
Contig9283	sp_Q10MI9_NAS2_ORYSJ_Nicotianamine_synthase_2_OS=Oryza_sativa_subsp._japonica_OX=39947_GN=NAS2_PE=2_SV=2	78.788	330	63	3	1264	284	1	326	2.1E-179	506
NODE_46861_length_1750_cov_13.169946_g16272_i1	sp_Q10MW3_PDC2_ORYSJ_Pyruvate_decarboxylase_2_OS=Oryza_sativa_subsp._japonica_OX=39947_GN=PDC2_PE=2_SV=1	81.609	522	91	3	1567	2	43	559	0	827
NODE_72790_length_1222_cov_32.975631_g37477_i0	sp_Q10QE9_U496A_ORYSJ_UPF0496_protein_1_OS=Oryza_sativa_subsp._japonica_OX=39947_GN=Os03g0199100_PE=2_SV=1	93.586	343	16	1	1013	3	35	377	0	558
BINPACKER_23604_2	sp_Q10RB4_BGAL5_ORYSJ_Beta-galactosidase_5_OS=Oryza_sativa_subsp._japonica_OX=39947_GN=Os03g0165400_PE=2_SV=1	86.903	565	74	0	2179	485	277	841	0	1021
NODE_19709_length_2767_cov_25.770973_g799_i2	sp_Q2R712_ACBSY_ORYSJ_Achilleol_B_synthase_OS=Oryza_sativa_subsp._japonica_OX=39947_GN=Os11g0285000_PE=1_SV=1	71.341	164	47	0	2204	2695	500	663	5E-71	253
BINPACKER_1285_1	sp_Q2R8Z5_ADH1_ORYSJ_Alcohol_dehydrogenase_1_OS=Oryza_sativa_subsp._japonica_OX=39947_GN=ADH1_PE=2_SV=2	93.566	373	24	0	1439	321	6	378	0	651
BINPACKER_9160_1	sp_Q39191_WAK1_ARATH_Wall-associated_receptor_kinase_1_OS=Arabidopsis_thaliana_OX=3702_GN=WAK1_PE=1_SV=2	35.541	740	426	18	2690	504	1	700	4.9E-142	446
BINPACKER_14_11	sp_Q3BAI2_YCX91_PHAAO_Uncharacterized_protein_ORF91_OS=Phalaenopsis_aphrodite_subsp._formosana_OX=308872_PE=4_SV=1	70.175	57	17	0	849	1019	2	58	6E-14	71.2
BINPACKER_14_13	sp_Q3BAI2_YCX91_PHAAO_Uncharacterized_protein_ORF91_OS=Phalaenopsis_aphrodite_subsp._formosana_OX=308872_PE=4_SV=1	70.175	57	17	0	849	1019	2	58	6E-14	71.2
Contig3195	sp_Q3BAI2_YCX91_PHAAO_Uncharacterized_protein_ORF91_OS=Phalaenopsis_aphrodite_subsp._formosana_OX=308872_PE=4_SV=1	70.175	57	17	0	849	1019	2	58	6E-14	71.2
NODE_133186_length_572_cov_23.322645_g81554_i0	sp_Q3BAI2_YCX91_PHAAO_Uncharacterized_protein_ORF91_OS=Phalaenopsis_aphrodite_subsp._formosana_OX=308872_PE=4_SV=1	70.455	44	13	0	441	572	2	45	2.53E-11	60.8
NODE_134037_length_567_cov_5.032389_g82310_i0	sp_Q3BAI2_YCX91_PHAAO_Uncharacterized_protein_ORF91_OS=Phalaenopsis_aphrodite_subsp._formosana_OX=308872_PE=4_SV=1	75	44	11	0	436	567	2	45	2.87E-14	68.6
NODE_139199_length_535_cov_26.688312_g86736_i5	sp_Q3BAI2_YCX91_PHAAO_Uncharacterized_protein_ORF91_OS=Phalaenopsis_aphrodite_subsp._formosana_OX=308872_PE=4_SV=1	72.5	40	11	0	122	3	6	45	2.88E-11	60.1
NODE_151940_length_470_cov_3.846348_g98473_i0	sp_Q3BAI2_YCX91_PHAAO_Uncharacterized_protein_ORF91_OS=Phalaenopsis_aphrodite_subsp._formosana_OX=308872_PE=4_SV=1	73.684	57	15	0	307	137	2	58	6.63E-23	89.7
NODE_17651_length_2896_cov_22.881686_g8768_i0	sp_Q3BAI2_YCX91_PHAAO_Uncharacterized_protein_ORF91_OS=Phalaenopsis_aphrodite_subsp._formosana_OX=308872_PE=4_SV=1	67.241	58	19	0	848	1021	1	58	3.7E-16	78.2
NODE_17911_length_2879_cov_20.718817_g8898_i0	sp_Q3BAI2_YCX91_PHAAO_Uncharacterized_protein_ORF91_OS=Phalaenopsis_aphrodite_subsp._formosana_OX=308872_PE=4_SV=1	78.947	57	12	0	848	1018	2	58	2.22E-21	92.8
NODE_26162_length_2424_cov_18.319864_g12701_i1	sp_Q3BAI2_YCX91_PHAAO_Uncharacterized_protein_ORF91_OS=Phalaenopsis_aphrodite_subsp._formosana_OX=308872_PE=4_SV=1	71.93	57	16	0	362	532	2	58	7.98E-18	82.4
NODE_91137_length_959_cov_12.576749_g49026_i0	sp_Q3BAI2_YCX91_PHAAO_Uncharacterized_protein_ORF91_OS=Phalaenopsis_aphrodite_subsp._formosana_OX=308872_PE=4_SV=1	75	48	12	0	146	3	2	49	4.22E-15	73.2
NODE_100018_length_855_cov_19.060102_g55046_i0	sp_Q43247_G3PC3_MAIZE_Glyceraldehyde-3-phosphate_dehydrogenase_3,_cytosolic_OS=Zea_mays_OX=4577_GN=GAPC3_PE=2_SV=1	91.667	216	18	0	837	190	2	217	3.1E-142	406
BINPACKER_34152_1	sp_Q53QK0_Y1103_ORYSJ_Flavonoid_O-methyltransferase-like_protein_Os11g0303600_OS=Oryza_sativa_subsp._japonica_OX=39947_GN=Os11g0303600_PE=2_SV=1	60.8	375	131	5	49	1128	1	374	3.8E-154	446
BINPACKER_2187_1	sp_Q54IK2_TM120_DICDI_Transmembrane_protein_120_homolog_OS=Dictyostelium_discoideum_OX=44689_GN=tmem120_PE=3_SV=1	34.241	257	140	7	1744	995	106	340	2.83E-22	102
NODE_62827_length_1400_cov_33.107762_g29664_i1	sp_Q55EX9_Y8948_DICDI_Putative_methyltransferase_DDB_G0268948_OS=Dictyostelium_discoideum_OX=44689_GN=DDB_G0268948_PE=1_SV=2	34.351	131	77	4	1032	643	19	141	2.58E-14	76.3
Contig3444	sp_Q56YA5_SGAT_ARATH_Serine--glyoxylate_aminotransferase_OS=Arabidopsis_thaliana_OX=3702_GN=AGT1_PE=1_SV=2	85.802	162	23	0	761	1246	2	163	0	262
BINPACKER_3209_2	sp_Q5E9D6_HDHD3_BOVIN_Haloacid_dehalogenase-like_hydrolase_domain-containing_protein_3_OS=Bos_taurus_OX=9913_GN=HDHD3_PE=2_SV=1	30.151	199	128	4	509	1075	13	210	5.09E-15	79.3
BINPACKER_8283_2	sp_Q5N8F2_ILL2_ORYSJ_IAA-amino_acid_hydrolase_ILR1-like_2_OS=Oryza_sativa_subsp._japonica_OX=39947_GN=ILL2_PE=2_SV=1	70.936	406	72	3	138	1226	49	451	3.7E-163	474
NODE_138126_length_541_cov_40.724359_g85952_i0	sp_Q5N8F2_ILL2_ORYSJ_IAA-amino_acid_hydrolase_ILR1-like_2_OS=Oryza_sativa_subsp._japonica_OX=39947_GN=ILL2_PE=2_SV=1	84.906	106	16	0	320	3	49	154	5.33E-39	141
NODE_32339_length_2185_cov_10.960701_g16179_i0	sp_Q5NE24_NSP2_MEDTR_Nodulation-signaling_pathway_2_protein_OS=Medicago_truncatula_OX=3880_GN=NSP2_PE=1_SV=1	51.125	489	200	12	341	1780	34	492	2.8E-122	378
NODE_101691_length_838_cov_34.048366_g56190_i0	sp_Q5SMM8_PHT1_ORYSJ_Putrescine_hydroxycinnamoyltransferase_1_OS=Oryza_sativa_subsp._japonica_OX=39947_GN=PHT1_PE=2_SV=1	46.309	298	139	8	838	2	137	432	2.89E-60	200
NODE_56050_length_1540_cov_18.354465_g28268_i0	sp_Q5SMM8_PHT1_ORYSJ_Putrescine_hydroxycinnamoyltransferase_1_OS=Oryza_sativa_subsp._japonica_OX=39947_GN=PHT1_PE=2_SV=1	45.57	316	155	7	1053	157	127	442	1.99E-59	206
BINPACKER_1138_6	sp_Q5SMQ9_PIN1A_ORYSJ_Auxin_efflux_carrier_component_1a_OS=Oryza_sativa_subsp._japonica_OX=39947_GN=PIN1A_PE=2_SV=1	93.445	595	37	1	2405	627	1	595	0	1109
NODE_2758_length_5351_cov_32.404320_g1400_i0	sp_Q5VNN5_DEF1B_ORYSJ_Peptide_deformylase_1B,_chloroplastic_OS=Oryza_sativa_subsp._japonica_OX=39947_GN=PDF1B_PE=2_SV=1	81.041	269	50	1	5282	4479	1	269	3.7E-119	380
NODE_11039_length_3470_cov_28.317044_g5534_i0	sp_Q5VQ09_KINUB_ORYSJ_Kinesin-like_protein_KIN-UB_OS=Oryza_sativa_subsp._japonica_OX=39947_GN=KINUB_PE=2_SV=1	90.166	844	68	5	3243	739	1	838	0	1493
NODE_12856_length_3287_cov_24.713752_g6441_i0	sp_Q5VQ09_KINUB_ORYSJ_Kinesin-like_protein_KIN-UB_OS=Oryza_sativa_subsp._japonica_OX=39947_GN=KINUB_PE=2_SV=1	88.259	494	53	2	1366	2832	397	890	0	847
NODE_7349_length_3974_cov_15.034863_g3367_i1	sp_Q5W7C6_KN5A_ORYSJ_Kinesin-like_protein_KIN-5A_OS=Oryza_sativa_subsp._japonica_OX=39947_GN=KIN5A_PE=2_SV=1	93.274	892	58	2	486	3155	1	892	0	1703
BINPACKER_584_15	sp_Q653U3_ARFQ_ORYSJ_Auxin_response_factor_17_OS=Oryza_sativa_subsp._japonica_OX=39947_GN=ARF17_PE=2_SV=1	87.595	919	108	3	3341	597	1	917	0	1449
BINPACKER_584_16	sp_Q653U3_ARFQ_ORYSJ_Auxin_response_factor_17_OS=Oryza_sativa_subsp._japonica_OX=39947_GN=ARF17_PE=2_SV=1	87.595	919	108	3	3341	597	1	917	0	1449
BINPACKER_12202_2	sp_Q655X8_AAH_ORYSJ_Probable_allantoate_deiminase_OS=Oryza_sativa_subsp._japonica_OX=39947_GN=AAH_PE=1_SV=1	89.016	437	46	1	1523	219	41	477	0	800
BINPACKER_12202_7	sp_Q655X8_AAH_ORYSJ_Probable_allantoate_deiminase_OS=Oryza_sativa_subsp._japonica_OX=39947_GN=AAH_PE=1_SV=1	79	100	19	1	462	169	380	479	4.49E-43	164
NODE_9415_length_3655_cov_30.104690_g4757_i0	sp_Q65X92_C3H37_ORYSJ_Zinc_finger_CCCH_domain-containing_protein_37_OS=Oryza_sativa_subsp._japonica_OX=39947_GN=Os05g0525900_PE=2_SV=1	84.127	252	34	3	1459	719	5	255	3.6E-105	335
NODE_29978_length_2271_cov_26.885805_g15050_i1	sp_Q69Q02_DPE2_ORYSJ_4-alpha-glucanotransferase_DPE2_OS=Oryza_sativa_subsp._japonica_OX=39947_GN=DPE2_PE=2_SV=1	88.434	415	48	0	492	1736	5	419	0	740
NODE_46908_length_1749_cov_17.919451_g23527_i0	sp_Q69UE6_AT10_ORYSJ_Acyl_transferase_10_OS=Oryza_sativa_subsp._japonica_OX=39947_GN=AT10_PE=3_SV=1	80.405	444	83	3	1681	359	1	443	0	681
NODE_88857_length_988_cov_5.486339_g47499_i0	sp_Q69UE6_AT10_ORYSJ_Acyl_transferase_10_OS=Oryza_sativa_subsp._japonica_OX=39947_GN=AT10_PE=3_SV=1	79.725	291	45	4	867	7	1	281	4E-138	402
Contig4536	sp_Q6MRB2_TOLB_BDEBA_Tol-Pal_system_protein_TolB_OS=Bdellovibrio_bacteriovorus_(strain_ATCC_15356_/_DSM_50701_/_NCIB_9529_/_HD100)_OX=264462_GN=tolB_PE=3_SV=1	28.889	180	100	7	1402	1938	304	456	7.37E-11	68.9
NODE_35177_length_2083_cov_33.545274_g17625_i0	sp_Q6MRB2_TOLB_BDEBA_Tol-Pal_system_protein_TolB_OS=Bdellovibrio_bacteriovorus_(strain_ATCC_15356_/_DSM_50701_/_NCIB_9529_/_HD100)_OX=264462_GN=tolB_PE=3_SV=1	28.889	180	100	7	1018	482	304	456	7.54E-11	68.6
BINPACKER_8299_4	sp_Q6RYA0_SABP2_TOBAC_Salicylic_acid-binding_protein_2_OS=Nicotiana_tabacum_OX=4097_GN=SABP2_PE=1_SV=1	50.775	258	119	3	1186	428	5	259	8.97E-83	257
NODE_13760_length_3198_cov_22.404800_g6913_i0	sp_Q6RYA0_SABP2_TOBAC_Salicylic_acid-binding_protein_2_OS=Nicotiana_tabacum_OX=4097_GN=SABP2_PE=1_SV=1	54.237	118	54	0	1	354	8	125	7.86E-35	137
Contig1468	sp_Q6Z310_MAN9_ORYSJ_Putative_mannan_endo-1,4-beta-mannosidase_9_OS=Oryza_sativa_subsp._japonica_OX=39947_GN=MAN9_PE=2_SV=2	81.283	187	33	1	812	252	221	405	5.6E-107	318
NODE_65343_length_1353_cov_23.025000_g17471_i2	sp_Q6Z9F4_CIPK6_ORYSJ_CBL-interacting_protein_kinase_6_OS=Oryza_sativa_subsp._japonica_OX=39947_GN=CIPK6_PE=2_SV=1	81.663	409	69	4	70	1296	18	420	0	639
BINPACKER_18504_1	sp_Q6ZI50_NAR21_ORYSJ_High-affinity_nitrate_transporter-activating_protein_2.1_OS=Oryza_sativa_subsp._japonica_OX=39947_GN=NAR2.1_PE=1_SV=1	70.69	174	50	1	738	217	34	206	5.74E-72	226
NODE_82305_length_1075_cov_25.474052_g42727_i1	sp_Q75HE5_NAC2_ORYSJ_NAC_domain-containing_protein_2_OS=Oryza_sativa_subsp._japonica_OX=39947_GN=NAC002_PE=1_SV=1	97.183	142	4	0	780	355	1	142	4.57E-97	293
BINPACKER_38833_1	sp_Q75KR1_PPDK2_ORYSJ_Pyruvate,_phosphate_dikinase_2_OS=Oryza_sativa_subsp._japonica_OX=39947_GN=PPDK2_PE=3_SV=1	86.943	651	85	0	1953	1	155	805	0	1160
NODE_64229_length_1374_cov_15.089931_g32617_i0	sp_Q75L84_GT43A_ORYSJ_Probable_beta-1,4-xylosyltransferase_GT43A_OS=Oryza_sativa_subsp._japonica_OX=39947_GN=GT43A_PE=2_SV=1	74.352	347	78	5	1374	346	30	369	1E-147	428
BINPACKER_2191_3	sp_Q76BW5_XTH8_ORYSJ_Xyloglucan_endotransglycosylase/hydrolase_protein_8_OS=Oryza_sativa_subsp._japonica_OX=39947_GN=XTH8_PE=1_SV=1	65.53	264	79	3	1099	341	28	290	3E-115	343
NODE_28600_length_2321_cov_27.559164_g8179_i4	sp_Q7F9I1_CLPC1_ORYSJ_Chaperone_protein_ClpC1,_chloroplastic_OS=Oryza_sativa_subsp._japonica_OX=39947_GN=CLPC1_PE=2_SV=2	95.022	462	23	0	375	1760	457	918	0	889
BINPACKER_20431_1	sp_Q7XPL2_HEM6_ORYSJ_Oxygen-dependent_coproporphyrinogen-III_oxidase,_chloroplastic_OS=Oryza_sativa_subsp._japonica_OX=39947_GN=CPX_PE=2_SV=2	88.076	369	37	2	1086	1	1	369	0	612
BINPACKER_20431_2	sp_Q7XPL2_HEM6_ORYSJ_Oxygen-dependent_coproporphyrinogen-III_oxidase,_chloroplastic_OS=Oryza_sativa_subsp._japonica_OX=39947_GN=CPX_PE=2_SV=2	87.534	369	39	2	1086	1	1	369	0	608
BINPACKER_20431_3	sp_Q7XPL2_HEM6_ORYSJ_Oxygen-dependent_coproporphyrinogen-III_oxidase,_chloroplastic_OS=Oryza_sativa_subsp._japonica_OX=39947_GN=CPX_PE=2_SV=2	87.263	369	40	2	1086	1	1	369	0	608
BINPACKER_23940_2	sp_Q7XPL2_HEM6_ORYSJ_Oxygen-dependent_coproporphyrinogen-III_oxidase,_chloroplastic_OS=Oryza_sativa_subsp._japonica_OX=39947_GN=CPX_PE=2_SV=2	83.471	121	17	1	365	3	229	346	5.29E-65	212
NODE_80484_length_1101_cov_32.330739_g42133_i0	sp_Q7XPL2_HEM6_ORYSJ_Oxygen-dependent_coproporphyrinogen-III_oxidase,_chloroplastic_OS=Oryza_sativa_subsp._japonica_OX=39947_GN=CPX_PE=2_SV=2	85.489	317	39	2	932	3	1	317	3.1E-176	498
BINPACKER_611_2	sp_Q7XUJ5_HOX22_ORYSJ_Homeobox-leucine_zipper_protein_HOX22_OS=Oryza_sativa_subsp._japonica_OX=39947_GN=HOX22_PE=2_SV=2	64.727	275	57	7	512	1249	13	276	1.39E-78	256
NODE_68906_length_1289_cov_30.866776_g31941_i1	sp_Q8GWC3_PR1A2_ARATH_PRA1_family_protein_A2_OS=Arabidopsis_thaliana_OX=3702_GN=PRA1A2_PE=2_SV=1	64.593	209	74	0	258	884	1	209	3.24E-93	282
NODE_22954_length_2581_cov_18.747209_g11467_i0	sp_Q8GZN1_NPG1_ARATH_Protein_NPG1_OS=Arabidopsis_thaliana_OX=3702_GN=NPG1_PE=1_SV=1	66.021	671	220	4	267	2261	35	703	0	842
BINPACKER_6513_4	sp_Q8H0B2_ARAE3_ORYSJ_Probable_UDP-arabinose_4-epimerase_3_OS=Oryza_sativa_subsp._japonica_OX=39947_GN=UEL-3_PE=2_SV=1	89.567	393	40	1	1463	288	14	406	0	742
NODE_84398_length_1046_cov_30.177801_g12630_i2	sp_Q8H5T6_LTI6A_ORYSJ_Hydrophobic_protein_LTI6A_OS=Oryza_sativa_subsp._japonica_OX=39947_GN=LTI6A_PE=2_SV=1	94.545	55	3	0	957	793	2	56	9.54E-18	79.3
BINPACKER_20779_13	sp_Q8L4H4_NORK_MEDTR_Nodulation_receptor_kinase_OS=Medicago_truncatula_OX=3880_GN=NORK_PE=1_SV=2	75.076	329	79	2	874	1857	582	908	7E-172	518
BINPACKER_20779_7	sp_Q8L4H4_NORK_MEDTR_Nodulation_receptor_kinase_OS=Medicago_truncatula_OX=3880_GN=NORK_PE=1_SV=2	55.752	565	198	8	353	1948	363	908	0	589
NODE_23859_length_2533_cov_25.785772_g5472_i1	sp_Q8L5C6_XIP1_WHEAT_Xylanase_inhibitor_protein_1_OS=Triticum_aestivum_OX=4565_GN=XIPI_PE=1_SV=2	80	275	51	2	666	1478	30	304	6.4E-155	458
BINPACKER_42406_1	sp_Q8L970_P4H7_ARATH_Probable_prolyl_4-hydroxylase_7_OS=Arabidopsis_thaliana_OX=3702_GN=P4H7_PE=2_SV=1	66.418	268	88	1	335	1138	51	316	5.5E-131	386
BINPACKER_27905_2	sp_Q8LFU0_NFYAA_ARATH_Nuclear_transcription_factor_Y_subunit_A-10_OS=Arabidopsis_thaliana_OX=3702_GN=NFYA10_PE=2_SV=2	52.033	123	53	3	1209	847	80	198	1.41E-25	109
NODE_99125_length_864_cov_23.309735_g54437_i0	sp_Q8LGG8_USPAL_ARATH_Universal_stress_protein_A-like_protein_OS=Arabidopsis_thaliana_OX=3702_GN=At3g01520_PE=1_SV=2	27.211	147	90	6	694	263	30	162	1.53E-10	62.4
Contig684	sp_Q8LGI2_SCPDL_ARATH_Probable_mitochondrial_saccharopine_dehydrogenase-like_oxidoreductase_At5g39410_OS=Arabidopsis_thaliana_OX=3702_GN=At5g39410_PE=1_SV=1	59.864	441	173	3	185	1507	11	447	3.4E-150	443
BINPACKER_124458_1	sp_Q8LJJ9_STAD1_ORYSJ_Stearoyl-[acyl-carrier-protein]_9-desaturase_1,_chloroplastic_OS=Oryza_sativa_subsp._japonica_OX=39947_GN=Os01g0880800_PE=2_SV=1	93.069	101	7	0	2	304	54	154	2.11E-64	202
NODE_128156_length_605_cov_3.635338_g77320_i0	sp_Q8LJJ9_STAD1_ORYSJ_Stearoyl-[acyl-carrier-protein]_9-desaturase_1,_chloroplastic_OS=Oryza_sativa_subsp._japonica_OX=39947_GN=Os01g0880800_PE=2_SV=1	91.892	185	15	0	3	557	197	381	3.3E-123	356
BINPACKER_24505_4	sp_Q8LPT9_GWD1_CITRE_Alpha-glucan_water_dikinase,_chloroplastic_OS=Citrus_reticulata_OX=85571_GN=R1_PE=2_SV=1	67.474	1445	448	10	5677	1370	44	1475	0	1966
NODE_3307_length_5078_cov_28.543257_g1703_i0	sp_Q8RWY6_CLASP_ARATH_CLIP-associated_protein_OS=Arabidopsis_thaliana_OX=3702_GN=CLASP_PE=1_SV=1	57.164	1326	471	17	4880	1032	11	1282	0	1323
NODE_34560_length_2105_cov_15.055610_g17315_i0	sp_Q8RXK7_PME41_ARATH_Probable_pectinesterase/pectinesterase_inhibitor_41_OS=Arabidopsis_thaliana_OX=3702_GN=PME41_PE=2_SV=2	54.204	559	230	8	1972	353	22	573	0	607
NODE_44923_length_1798_cov_8.315362_g22511_i0	sp_Q8RXK7_PME41_ARATH_Probable_pectinesterase/pectinesterase_inhibitor_41_OS=Arabidopsis_thaliana_OX=3702_GN=PME41_PE=2_SV=2	57.38	542	202	10	1686	133	35	571	0	627
NODE_10512_length_3521_cov_28.091937_g2081_i3	sp_Q8RY59_RCD1_ARATH_Inactive_poly_[ADP-ribose]_polymerase_RCD1_OS=Arabidopsis_thaliana_OX=3702_GN=RCD1_PE=1_SV=1	32.051	546	309	11	1394	2935	54	569	6.86E-74	260
Contig645	sp_Q8S9J6_ASPA_ARATH_Aspartyl_protease_family_protein_At5g10770_OS=Arabidopsis_thaliana_OX=3702_GN=At5g10770_PE=2_SV=1	45	400	201	12	329	1474	62	460	2.4E-93	298
NODE_26829_length_2395_cov_26.456934_g12040_i2	sp_Q8VYL8_DTX10_ARATH_Protein_DETOXIFICATION_10_OS=Arabidopsis_thaliana_OX=3702_GN=DTX10_PE=2_SV=1	51.134	397	184	3	109	1284	5	396	3.1E-121	377
NODE_34096_length_2121_cov_27.525391_g497_i6	sp_Q8VZ80_PLT5_ARATH_Polyol_transporter_5_OS=Arabidopsis_thaliana_OX=3702_GN=PLT5_PE=1_SV=2	45.427	328	166	4	797	1744	172	498	1.42E-77	261
BINPACKER_670_1	sp_Q8VZF1_AEE7_ARATH_Acetate/butyrate--CoA_ligase_AAE7,_peroxisomal_OS=Arabidopsis_thaliana_OX=3702_GN=AAE7_PE=1_SV=1	70.515	563	166	0	2801	1113	7	569	0	807
NODE_80300_length_1104_cov_19.317168_g29241_i3	sp_Q8VZF6_EDR2L_ARATH_Protein_ENHANCED_DISEASE_RESISTANCE_2-like_OS=Arabidopsis_thaliana_OX=3702_GN=EDR2L_PE=2_SV=1	27.622	286	159	8	357	1094	9	286	2.88E-19	92.8
BINPACKER_6140_1	sp_Q8VZS0_PNC2_ARATH_Peroxisomal_adenine_nucleotide_carrier_2_OS=Arabidopsis_thaliana_OX=3702_GN=PNC2_PE=1_SV=1	68.75	48	14	1	1491	1634	5	51	4.58E-13	73.9
BINPACKER_1792_1	sp_Q8W031_ZWIP5_ARATH_Zinc_finger_protein_WIP5_OS=Arabidopsis_thaliana_OX=3702_GN=WIP5_PE=2_SV=1	75.472	159	37	1	1351	875	159	315	2.07E-85	275
BINPACKER_4223_1	sp_Q8W0E4_SAT1_ORYSJ_Probable_serine_acetyltransferase_1_OS=Oryza_sativa_subsp._japonica_OX=39947_GN=SAT1_PE=2_SV=1	91.045	268	23	1	246	1049	37	303	9.1E-131	394
NODE_53928_length_1584_cov_32.868961_g20729_i1	sp_Q8W0E4_SAT1_ORYSJ_Probable_serine_acetyltransferase_1_OS=Oryza_sativa_subsp._japonica_OX=39947_GN=SAT1_PE=2_SV=1	90.728	302	23	3	71	976	7	303	6.4E-147	426
NODE_49538_length_1687_cov_22.351921_g24844_i0	sp_Q93WV4_WRK71_ARATH_WRKY_transcription_factor_71_OS=Arabidopsis_thaliana_OX=3702_GN=WRKY71_PE=2_SV=1	81.928	83	15	0	944	696	120	202	3.45E-43	159
BINPACKER_2461_5	sp_Q93YV6_2A5K_ARATH_Serine/threonine_protein_phosphatase_2A_57_kDa_regulatory_subunit_B'_kappa_isoform_OS=Arabidopsis_thaliana_OX=3702_GN=B'KAPPA_PE=2_SV=1	60.817	416	162	1	1526	279	85	499	0	537
NODE_56899_length_1522_cov_41.382333_g28686_i0	sp_Q93ZR6_WSD1_ARATH_O-acyltransferase_WSD1_OS=Arabidopsis_thaliana_OX=3702_GN=WSD1_PE=1_SV=1	37.461	323	182	6	571	1521	72	380	3.5E-54	192
BINPACKER_4825_6	sp_Q94AA4_PFKA3_ARATH_ATP-dependent_6-phosphofructokinase_3_OS=Arabidopsis_thaliana_OX=3702_GN=PFK3_PE=1_SV=1	75.854	439	105	1	449	1765	25	462	0	696
NODE_28745_length_2316_cov_23.781097_g14432_i0	sp_Q94AA4_PFKA3_ARATH_ATP-dependent_6-phosphofructokinase_3_OS=Arabidopsis_thaliana_OX=3702_GN=PFK3_PE=1_SV=1	73.009	226	61	0	813	1490	1	226	2.5E-112	349
NODE_46714_length_1753_cov_23.877976_g23430_i0	sp_Q94AA4_PFKA3_ARATH_ATP-dependent_6-phosphofructokinase_3_OS=Arabidopsis_thaliana_OX=3702_GN=PFK3_PE=1_SV=1	77.578	446	98	2	145	1482	14	457	0	722
NODE_56919_length_1522_cov_26.035197_g28692_i0	sp_Q94CS9_TIP12_ORYSJ_Probable_aquaporin_TIP1-2_OS=Oryza_sativa_subsp._japonica_OX=39947_GN=TIP1-2_PE=2_SV=1	96.032	252	10	0	521	1276	1	252	1.1E-148	428
BINPACKER_35519_1	sp_Q96262_PCAP1_ARATH_Plasma_membrane-associated_cation-binding_protein_1_OS=Arabidopsis_thaliana_OX=3702_GN=PCAP1_PE=1_SV=1	53.788	132	59	2	137	532	1	130	3.44E-31	120
Contig3349	sp_Q96329_ACOX4_ARATH_Acyl-coenzyme_A_oxidase_4,_peroxisomal_OS=Arabidopsis_thaliana_OX=3702_GN=ACX4_PE=1_SV=1	63.415	123	45	0	369	1	5	127	8.33E-49	167
BINPACKER_16981_1	sp_Q9AUK4_MRS2A_ORYSJ_Magnesium_transporter_MRS2-A,_chloroplastic_OS=Oryza_sativa_subsp._japonica_OX=39947_GN=MRS2-A_PE=2_SV=1	95.389	347	16	0	1266	226	128	474	0	584
BINPACKER_39419_2	sp_Q9AWM8_KN7A_ORYSJ_Kinesin-like_protein_KIN-7A_OS=Oryza_sativa_subsp._japonica_OX=39947_GN=KIN7A_PE=2_SV=1	83.636	550	71	5	1598	3196	407	954	0	881
BINPACKER_39419_2	sp_Q9AWM8_KN7A_ORYSJ_Kinesin-like_protein_KIN-7A_OS=Oryza_sativa_subsp._japonica_OX=39947_GN=KIN7A_PE=2_SV=1	89.851	404	41	0	384	1595	1	404	0	757
NODE_8241_length_3829_cov_31.342918_g3327_i1	sp_Q9C698_FPP6_ARATH_Filament-like_plant_protein_6_OS=Arabidopsis_thaliana_OX=3702_GN=FPP6_PE=1_SV=1	44.063	1019	438	22	3494	669	1	964	0	589
NODE_26900_length_2392_cov_27.932730_g9198_i1	sp_Q9C8G5_CSCLD_ARATH_CSC1-like_protein_ERD4_OS=Arabidopsis_thaliana_OX=3702_GN=ERD4_PE=1_SV=1	58.885	574	226	3	223	1938	148	713	0	657
BINPACKER_18308_1	sp_Q9C9G7_MYB62_ARATH_Transcription_factor_MYB62_OS=Arabidopsis_thaliana_OX=3702_GN=MYB62_PE=2_SV=1	80.952	105	20	0	1089	775	18	122	7.45E-56	191
NODE_63707_length_1384_cov_4.981693_g32341_i0	sp_Q9C9G7_MYB62_ARATH_Transcription_factor_MYB62_OS=Arabidopsis_thaliana_OX=3702_GN=MYB62_PE=2_SV=1	78.571	140	30	0	1278	859	18	157	2.92E-75	240
NODE_45180_length_1791_cov_29.323050_g22645_i0	sp_Q9FGR9_CEP1_ARATH_KDEL-tailed_cysteine_endopeptidase_CEP1_OS=Arabidopsis_thaliana_OX=3702_GN=CEP1_PE=1_SV=1	66.565	329	107	2	1425	448	21	349	2.5E-165	478
NODE_40819_length_1908_cov_28.392916_g20430_i0	sp_Q9FI21_BBE28_ARATH_Berberine_bridge_enzyme-like_28_OS=Arabidopsis_thaliana_OX=3702_GN=At5g44440_PE=1_SV=1	45.318	534	267	9	1855	308	1	527	1E-152	454
NODE_36100_length_2052_cov_30.317332_g18108_i0	sp_Q9FIK0_PFKA2_ARATH_ATP-dependent_6-phosphofructokinase_2_OS=Arabidopsis_thaliana_OX=3702_GN=PFK2_PE=1_SV=1	74.638	414	102	2	1525	284	28	438	0	640
BINPACKER_637_6	sp_Q9FJ95_DHSO_ARATH_Sorbitol_dehydrogenase_OS=Arabidopsis_thaliana_OX=3702_GN=SDH_PE=1_SV=1	79.94	334	67	0	1031	2032	31	364	0	558
NODE_86474_length_1018_cov_43.617989_g45985_i0	sp_Q9FJ95_DHSO_ARATH_Sorbitol_dehydrogenase_OS=Arabidopsis_thaliana_OX=3702_GN=SDH_PE=1_SV=1	80.665	331	64	0	2	994	34	364	0	557
BINPACKER_44392_1	sp_Q9FLK4_GSXL8_ARATH_Flavin-containing_monooxygenase_FMO_GS-OX-like_8_OS=Arabidopsis_thaliana_OX=3702_GN=At5g61290_PE=1_SV=1	52.298	457	201	6	747	2114	12	452	1.9E-155	462

BINPACKER_11034_1	sp_Q9FSV7_SST_FESAR_Sucrose:sucrose_1-fructosyltransferase_OS=Festuca_arundinacea_OX=4606_GN=1-SST_PE=1_SV=1	66.011	356	103	6	1382	321	90	429	4.5E-164	490
NODE_9551_length_3635_cov_31.888827_g4825_i0	sp_Q9FW70_KN7K_ORYSJ_Kinesin-like_protein_KIN-7K,_chloroplastic_OS=Oryza_sativa_subsp._japonica_OX=39947_GN=KIN7K_PE=2_SV=2	89.546	947	97	1	459	3293	101	1047	0	1624
BINPACKER_17823_1	sp_Q9FWX7_AB11B_ARATH_ABC_transporter_B_family_member_11_OS=Arabidopsis_thaliana_OX=3702_GN=ABCB11_PE=2_SV=1	28.859	149	105	1	3964	3521	756	904	1.73E-11	73.6
BINPACKER_13772_2	sp_Q9FXI5_IQD32_ARATH_Protein_IQ-DOMAIN_32_OS=Arabidopsis_thaliana_OX=3702_GN=IQD32_PE=1_SV=3	32.012	656	328	26	934	2778	215	793	4.37E-60	224
BINPACKER_9218_2	sp_Q9LE59_GAUT1_ARATH_Polygalacturonate_4-alpha-galacturonosyltransferase_OS=Arabidopsis_thaliana_OX=3702_GN=GAUT1_PE=1_SV=1	76.407	551	122	3	1968	331	126	673	0	900
Contig3919	sp_Q9LFR7_CXE17_ARATH_Probable_carboxylesterase_17_OS=Arabidopsis_thaliana_OX=3702_GN=CXE17_PE=2_SV=1	30.838	334	209	8	164	1159	28	341	4.14E-34	134
BINPACKER_12924_2	sp_Q9LGE3_FLP1_ORYSJ_Flowering-promoting_factor_1-like_protein_1_OS=Oryza_sativa_subsp._japonica_OX=39947_GN=RAA1_PE=1_SV=1	77.982	109	22	1	89	409	1	109	5.79E-53	170
NODE_27585_length_2363_cov_23.834498_g13831_i0	sp_Q9LHA8_MD37C_ARATH_Probable_mediator_of_RNA_polymerase_II_transcription_subunit_37c_OS=Arabidopsis_thaliana_OX=3702_GN=MED37C_PE=1_SV=1	93.21	648	40	2	2101	170	3	650	0	1144
BINPACKER_2296_5	sp_Q9LIG2_RLK6_ARATH_Receptor-like_protein_kinase_At3g21340_OS=Arabidopsis_thaliana_OX=3702_GN=At3g21340_PE=1_SV=1	51.007	298	139	3	1253	372	577	871	1.78E-98	319
BINPACKER_3082_1	sp_Q9LIG2_RLK6_ARATH_Receptor-like_protein_kinase_At3g21340_OS=Arabidopsis_thaliana_OX=3702_GN=At3g21340_PE=1_SV=1	51.504	266	125	3	994	203	608	871	4.48E-84	275
NODE_39551_length_1944_cov_32.735970_g19822_i0	sp_Q9LIS3_GAE6_ARATH_UDP-glucuronate_4-epimerase_6_OS=Arabidopsis_thaliana_OX=3702_GN=GAE6_PE=1_SV=1	63.457	457	146	5	1938	580	10	449	1.2E-178	518
NODE_40213_length_1925_cov_29.536717_g20138_i0	sp_Q9LPL4_SKP2A_ARATH_F-box_protein_SKP2A_OS=Arabidopsis_thaliana_OX=3702_GN=SKP2A_PE=1_SV=1	64.986	357	101	5	1573	506	22	355	1.1E-140	417
BINPACKER_795_4	sp_Q9LQU5_NUD18_ARATH_Nudix_hydrolase_18,_mitochondrial_OS=Arabidopsis_thaliana_OX=3702_GN=NUDT18_PE=2_SV=1	55.714	140	53	4	421	822	1	137	1.15E-34	131
NODE_54225_length_1577_cov_36.410239_g27333_i0	sp_Q9LSG3_GAUT8_ARATH_Galacturonosyltransferase_8_OS=Arabidopsis_thaliana_OX=3702_GN=GAUT8_PE=1_SV=1	52.67	412	168	3	1164	4	150	559	2.2E-146	434
NODE_29011_length_2306_cov_23.732199_g14561_i0	sp_Q9LUC6_C7A14_ARATH_Cytochrome_P450_72A14_OS=Arabidopsis_thaliana_OX=3702_GN=CYP72A14_PE=2_SV=1	54.911	448	176	3	749	2092	91	512	2.3E-158	472
BINPACKER_8827_4	sp_Q9LUL4_SRF7_ARATH_Protein_STRUBBELIG-RECEPTOR_FAMILY_7_OS=Arabidopsis_thaliana_OX=3702_GN=SRF7_PE=1_SV=1	58.419	683	263	9	2387	381	27	702	0	698
NODE_148876_length_484_cov_19.729927_g95662_i0	sp_Q9LV58_MBF1C_ARATH_Multiprotein-bridging_factor_1c_OS=Arabidopsis_thaliana_OX=3702_GN=MBF1C_PE=1_SV=1	62.329	146	53	2	47	481	1	145	9.23E-47	152
NODE_78906_length_1125_cov_27.942015_g41204_i0	sp_Q9LV60_CRR55_ARATH_Cysteine-rich_repeat_secretory_protein_55_OS=Arabidopsis_thaliana_OX=3702_GN=CRRSP55_PE=2_SV=1	45.455	231	121	3	1008	331	20	250	1.09E-51	176
NODE_80068_length_1107_cov_34.284333_g41882_i0	sp_Q9LV60_CRR55_ARATH_Cysteine-rich_repeat_secretory_protein_55_OS=Arabidopsis_thaliana_OX=3702_GN=CRRSP55_PE=2_SV=1	45.887	231	120	3	120	797	20	250	5.18E-51	174
NODE_18274_length_2857_cov_27.122845_g9087_i0	sp_Q9LVM0_Y5830_ARATH_Probable_inactive_receptor_kinase_At5g58300_OS=Arabidopsis_thaliana_OX=3702_GN=At5g58300_PE=1_SV=1	58.966	619	238	8	387	2207	37	651	0	629
NODE_79868_length_1110_cov_25.926712_g41768_i0	sp_Q9LXG9_PCO1_ARATH_Plant_cysteine_oxidase_1_OS=Arabidopsis_thaliana_OX=3702_GN=PCO1_PE=1_SV=1	41.2	250	117	8	947	252	54	291	2.8E-51	176
BINPACKER_2840_5	sp_Q9LY62_GT644_ARATH_Glycosyltransferase_family_64_protein_C4_OS=Arabidopsis_thaliana_OX=3702_GN=EPC1_PE=2_SV=1	56.929	267	108	2	471	1250	68	334	3.98E-95	310
NODE_30577_length_2248_cov_25.819310_g15329_i0	sp_Q9LYU8_AK1_ARATH_Aspartokinase_1,_chloroplastic_OS=Arabidopsis_thaliana_OX=3702_GN=AK1_PE=1_SV=1	83.547	468	77	0	1854	451	83	550	0	785
BINPACKER_8747_1	sp_Q9LZA4_PMT7_ARATH_Probable_methyltransferase_PMT7_OS=Arabidopsis_thaliana_OX=3702_GN=At5g04060_PE=2_SV=1	64.02	592	208	3	89	1861	12	599	0	798
BINPACKER_690_1	sp_Q9LZF1_HIP6_ARATH_Heavy_metal-associated_isoprenylated_plant_protein_6_OS=Arabidopsis_thaliana_OX=3702_GN=HIPP06_PE=1_SV=1	58.462	65	27	0	1745	1551	155	219	2.87E-09	63.9
BINPACKER_21821_1	sp_Q9M1E2_PTR37_ARATH_Protein_NRT1/_PTR_FAMILY_2.7_OS=Arabidopsis_thaliana_OX=3702_GN=NPF2.7_PE=1_SV=1	45.37	540	279	5	1813	197	21	545	6.4E-161	476
BINPACKER_11579_11	sp_Q9M302_PP270_ARATH_Pentatricopeptide_repeat-containing_protein_At3g48810_OS=Arabidopsis_thaliana_OX=3702_GN=At3g48810_PE=2_SV=1	24.671	304	207	2	2953	2093	44	342	1.3E-26	121
NODE_53437_length_1595_cov_27.736531_g22454_i1	sp_Q9M886_LBD41_ARATH_LOB_domain-containing_protein_41_OS=Arabidopsis_thaliana_OX=3702_GN=LBD41_PE=2_SV=1	68.421	152	39	4	1258	821	1	149	6.19E-60	202
NODE_87937_length_1000_cov_3.678533_g46913_i0	sp_Q9M886_LBD41_ARATH_LOB_domain-containing_protein_41_OS=Arabidopsis_thaliana_OX=3702_GN=LBD41_PE=2_SV=1	64.138	145	49	1	106	531	1	145	3.29E-63	204
NODE_12121_length_3358_cov_22.675799_g6071_i0	sp_Q9M8S0_MBS1_ARATH_Protein_METHYLENE_BLUE_SENSITIVITY_1_OS=Arabidopsis_thaliana_OX=3702_GN=MBS1_PE=2_SV=1	62.069	116	33	1	286	633	1	105	2.16E-38	142
BINPACKER_22535_1	sp_Q9MAU3_TAF6_ARATH_Transcription_initiation_factor_TFIID_subunit_6_OS=Arabidopsis_thaliana_OX=3702_GN=TAF6_PE=1_SV=1	57.895	551	224	4	1955	321	1	549	0	600
BINPACKER_6832_1	sp_Q9S850_SUOX_ARATH_Sulfite_oxidase_OS=Arabidopsis_thaliana_OX=3702_GN=SOX_PE=1_SV=1	73.057	193	52	0	1098	520	4	196	5E-99	304
NODE_16415_length_2993_cov_23.076370_g7297_i2	sp_Q9S850_SUOX_ARATH_Sulfite_oxidase_OS=Arabidopsis_thaliana_OX=3702_GN=SOX_PE=1_SV=1	68.182	22	6	1	2850	2785	77	97	5.64E-10	30.4
BINPACKER_34092_11	sp_Q9SAY1_SUT11_ARATH_Sulfate_transporter_1.1_OS=Arabidopsis_thaliana_OX=3702_GN=SULTR1;1_PE=1_SV=2	55.556	45	20	0	1886	2020	593	637	1.62E-06	55.8
Contig7932	sp_Q9SBJ1_PDK_ARATH_[Pyruvate_dehydrogenase_(acetyl-transferring)]_kinase,_mitochondrial_OS=Arabidopsis_thaliana_OX=3702_GN=PDK_PE=1_SV=1	77.083	96	22	0	768	481	181	276	8.72E-46	160
NODE_37970_length_1992_cov_23.769151_g19045_i0	sp_Q9SGD1_ZWIP3_ARATH_Zinc_finger_protein_WIP3_OS=Arabidopsis_thaliana_OX=3702_GN=WIP3_PE=2_SV=1	46.567	335	126	8	1516	569	16	316	3.33E-86	276
BINPACKER_380_1	sp_Q9SGP2_HSL1_ARATH_Receptor-like_protein_kinase_HSL1_OS=Arabidopsis_thaliana_OX=3702_GN=HSL1_PE=2_SV=1	37.713	997	547	25	346	3261	26	973	2.1E-180	573
BINPACKER_86140_1	sp_Q9SIS3_MA656_ARATH_65-kDa_microtubule-associated_protein_6_OS=Arabidopsis_thaliana_OX=3702_GN=MAP65-6_PE=1_SV=1	54.313	313	142	1	1461	523	165	476	1.6E-114	353
BINPACKER_4908_5	sp_Q9SQL2_CB24_PEA_Chlorophyll_a-b_binding_protein_P4,_chloroplastic_OS=Pisum_sativum_OX=3888_GN=lhcA-P4_PE=1_SV=1	88.525	183	21	0	331	879	69	251	4E-115	345
Contig10872	sp_Q9SRM3_DIOX6_ARATH_Probable_2-oxoglutarate-dependent_dioxygenase_At3g111800_OS=Arabidopsis_thaliana_OX=3702_GN=At3g11180_PE=2_SV=1	56.25	352	150	3	1331	282	46	395	7E-140	410
Contig294	sp_Q9SX31_PERK9_ARATH_Proline-rich_receptor-like_protein_kinase_PERK9_OS=Arabidopsis_thaliana_OX=3702_GN=PERK9_PE=1_SV=1	57.871	451	175	4	1581	235	267	704	1E-168	511
Contig96	sp_Q9SX33_ALA9_ARATH_Putative_phospholipid-transporting_ATPase_9_OS=Arabidopsis_thaliana_OX=3702_GN=ALA9_PE=3_SV=1	67.282	1192	372	5	335	3889	13	1193	0	1671
NODE_25259_length_2465_cov_27.476171_g3153_i3	sp_Q9SY57_P2A10_ARATH_Protein_PHLOEM_PROTEIN_2-LIKE_A10_OS=Arabidopsis_thaliana_OX=3702_GN=PP2A10_PE=2_SV=1	35.78	327	183	5	2173	1274	81	407	1.25E-48	181
Contig3300	sp_Q9SZ67_WRK19_ARATH_Probable_WRKY_transcription_factor_19_OS=Arabidopsis_thaliana_OX=3702_GN=WRKY19_PE=3_SV=1	44.444	189	76	5	1314	793	91	265	2.08E-33	144
BINPACKER_2914_2	sp_Q9WX37_RBPE_NOSS1_Putative_RNA-binding_protein_RbpE_OS=Nostoc_sp._(strain_PCC_7120_/_SAG_25.82_/_UTEX_2576)_OX=103690_GN=rbpE_PE=3_SV=3	42.857	77	44	0	950	720	2	78	3.23E-15	75.5
NODE_21771_length_2647_cov_22.940559_g10853_i0	sp_Q9XIH7_PLT1_ARATH_Putative_polyol_transporter_1_OS=Arabidopsis_thaliana_OX=3702_GN=PLT1_PE=3_SV=1	45.781	474	246	5	1840	443	19	489	3.69E-88	293
NODE_7179_length_4001_cov_26.627546_g3631_i0	sp_Q9ZPS7_TMN3_ARATH_Transmembrane_9_superfamily_member_3_OS=Arabidopsis_thaliana_OX=3702_GN=TMN3_PE=2_SV=1	86.486	74	10	0	3247	3468	519	592	3.59E-26	119
BINPACKER_24495_2	sp_Q9ZQ31_STY13_ARATH_Serine/threonine-protein_kinase_STY13_OS=Arabidopsis_thaliana_OX=3702_GN=STY13_PE=1_SV=2	45.055	182	99	1	1440	895	209	389	8.73E-53	186
Contig4422	sp_Q9ZQ31_STY13_ARATH_Serine/threonine-protein_kinase_STY13_OS=Arabidopsis_thaliana_OX=3702_GN=STY13_PE=1_SV=2	40.138	289	153	3	1392	526	121	389	1.03E-68	231
BINPACKER_7098_2	sp_Q9ZUF4_RIPK_ARATH_Serine/threonine-protein_kinase_RIPK_OS=Arabidopsis_thaliana_OX=3702_GN=RIPK_PE=1_SV=1	71.429	168	47	1	606	103	61	227	5.82E-80	253
NODE_35950_length_2058_cov_20.484635_g7548_i2	sp_Q9ZUF4_RIPK_ARATH_Serine/threonine-protein_kinase_RIPK_OS=Arabidopsis_thaliana_OX=3702_GN=RIPK_PE=1_SV=1	75.556	45	11	0	1095	1229	137	181	1.26E-39	75.9
BINPACKER_1095_3	sp_Q9ZUX1_C94C1_ARATH_Cytochrome_P450_94C1_OS=Arabidopsis_thaliana_OX=3702_GN=CYP94C1_PE=1_SV=1	58.422	469	187	6	442	1836	31	495	0	548
BINPACKER_10164_6	sp_Q9ZW96_LBD40_ARATH_LOB_domain-containing_protein_40_OS=Arabidopsis_thaliana_OX=3702_GN=LBD40_PE=2_SV=1	74.453	137	34	1	1395	985	1	136	1.69E-59	201
