# Supplementary material for: De novo transcriptome assembly and analysis of Phragmites karka, an invasive halophyte, to study the mechanism of salinity stress tolerance
Source: Sci Rep. 2020 Mar 23;10:5192. doi: 10.1038/s41598-020-61857-8 (PMC7089983; doi:10.1038/s41598-020-61857-8)
Supplement: Supplementary file 4 — Supporting Information4. [file 41598_2020_61857_MOESM4_ESM.pdf]

TableS3a. In silico expression data for DEGs common to both leaf and root tissue of*P. karka* during exposure to salinity stres

Unigene ID	Control_leaf1	Control_leaf2	Salt_treated_leaf1	Salt_treated_leaf2	Control_root1	Control_root2	Salt_treated_root1	Salt_treated_root2
BINPACKER_102_1	3.154	1.661	7.754	8.592	2.652	1.98	0.741	0.469
BINPACKER_10495_1	29.764	46.051	11.997	11.892	4.302	4.916	34.712	19.53
BINPACKER_10852_2	1.101	0.469	0	0	0.476	0.415	2.032	1.202
BINPACKER_12014_1	3.144	5.614	0	0	1.003	1.456	0	0
BINPACKER_1274_1	155.244	313.382	33.713	45.346	6.229	10.505	66.395	49.265
BINPACKER_1274_2	193.391	313.02	40.54	54.568	8.618	11.998	67.566	54.631
BINPACKER_12853_2	33.013	63.912	6.887	8.202	1.33	1.919	13.542	7.595
BINPACKER_12853_5	39.428	68.655	8.128	10.134	0.946	1.823	17.073	8.651
BINPACKER_13772_2	0	0	1.65	1.352	1.465	2.371	0	0
BINPACKER_14118_2	10.87	1.742	0.629	0.514	19.533	13.201	44.396	84.806
BINPACKER_15872_1	10.479	5.574	19.658	18.933	2.51	1.655	0.542	0.235
BINPACKER_15897_5	2.583	4.73	0.349	0.265	5.852	7.593	1.124	0.792
BINPACKER_16_3	61.814	82.469	21.733	15.317	7.822	5.193	19.504	19.295
BINPACKER_17855_4	89.81	96.417	215.447	174.775	7.296	7.298	55.196	18.064
BINPACKER_2187_1	233.337	393.05	44.579	54.527	14.136	13.712	85.158	52.198
BINPACKER_23437_2	7.579	14.564	1.301	2.463	0.64	0.632	3.148	2.698
BINPACKER_2461_5	3.376	2.506	0	0	2.169	4.91	0	0
BINPACKER_2511_15	32.558	41.871	16.529	17.407	5.845	5.433	17.392	11.847
BINPACKER_283_11	5.028	5.239	1.343	0.954	7.623	7.274	40.937	29.354
BINPACKER_4115_1	30.198	50.768	15.16	9.122	3.968	2.792	20.58	6.891
BINPACKER_5619_12	0	0	3.529	1.327	3.84	2.082	0	0
BINPACKER_637_6	25.668	49.361	13.842	9.636	0.889	1.366	8.999	8.182
BINPACKER_6848_1	2.667	5.212	1.063	0.24	0.519	0	2.152	5.132
BINPACKER_7001_3	1.037	1.125	0	0	0.441	0.578	0	0
BINPACKER_7709_6	138.69	160.637	65.683	64.238	5.369	6.065	39.622	14.897
BINPACKER_8843_3	0	0	1.275	7.389	0.69	0.86	0	0
BINPACKER_9475_2	75.976	73.867	39.622	31.497	1.166	1.42	7.564	5.484
BINPACKER_99441_1	26.25	32.398	92.789	64.345	7.559	8.027	31.436	20.791
Contig10164	3.578	3.926	13.987	13.526	14.669	11.805	4.089	2.932
Contig11484	8.013	4.18	19.726	19.29	10.339	9.013	2.981	2.199
Contig11505	19.656	22.51	42.394	46.864	1.166	0.86	0	0
Contig11748	323.285	496.167	149.067	97.775	21.531	21.053	156.343	51.523
Contig3373	11.156	5.426	36.621	29.407	6.065	5.415	1.012	1.466
Contig3734	0	0	1.114	0.995	1.081	0.866	0.231	0
Contig4764	158.663	208.872	60.666	60.464	53.273	40.752	164.672	102.87
Contig5088	1.143	0.348	7.771	5.896	1.813	2.184	0.614	0.352
Contig5792	317.157	469.061	150.615	83.079	37.167	32.238	228.141	113.779
Contig7048	20.397	14.966	3.461	0.929	5.433	4.693	19.01	12.228
Contig729	62.269	56.65	25.227	30.883	20.223	17.148	99.736	37.418
Contig8508	43.492	28.861	92.984	88.702	0.761	1.221	42.515	5.19
Contig8714	24.948	30.549	3.486	2.438	0.86	1.294	5.779	5.014
Contig914	79.363	71.402	167.935	182.148	63.057	56.571	441.37	144.687
NODE_113847_length_719_cov_32.125387_g65339_i0	1.418	2.023	12.006	87.806	3.925	1.751	15.351	8.68
NODE_12121_length_3358_cov_22.675799_g6071_i0	17.613	38.896	10.314	9.462	5.681	4.82	26.399	16.979
NODE_124392_length_632_cov_75.901610_g74075_i0	239.339	283.677	140.327	92.641	24.887	33.64	223.877	84.836
NODE_17211_length_2932_cov_24.168940_g8539_i0	51.822	86.328	21.18	14.919	9.884	11.956	74.573	20.439
NODE_17436_length_2912_cov_20.072561_g8651_i0	0	0	0.978	1.343	0.597	0.499	6.209	1.847
NODE_18844_length_2822_cov_22.405602_g9393_i0	30.664	45.582	15.611	9.429	1.571	1.258	14.889	4.193
NODE_198620_length_317_cov_1.045082_g142860_i0	680.833	1098.223	124.299	195.624	32.993	80.277	359.999	264.301
NODE_20942_length_2692_cov_27.568156_g9710_i1	0.339	0	2.168	2.82	1.628	0.981	0	0
NODE_21453_length_2664_cov_22.591663_g10685_i0	8.203	2.68	17.209	21.114	6.364	6.504	1.355	1.437
NODE_23067_length_2575_cov_35.130695_g1727_i4	1826.35	2093.96	824.309	893.057	53.174	43.983	428.306	232.983
NODE_23146_length_2571_cov_26.692954_g11564_i0	0	0	1.684	0.887	1.33	0.909	0	0
NODE_24015_length_2525_cov_30.757749_g12037_i0	5.038	4.046	20.644	17.274	7.047	7.984	2.335	1.202
NODE_26900_length_2392_cov_27.932730_g9198_i1	31.457	18.209	9.761	5.357	11.128	10.361	0	0
NODE_30996_length_2232_cov_32.053265_g15531_i0	5.737	1.581	29.734	17.706	60.597	49.411	12.386	15.542
NODE_32549_length_2176_cov_31.285782_g16286_i0	2.35	2.439	8.945	7.157	1.685	2.058	0.279	0.117
NODE_37914_length_1994_cov_21.561166_g19016_i0	7.42	10.491	2.78	2.919	5.681	5.138	117.837	18.944
NODE_42638_length_1859_cov_26.571109_g21306_i0	11.58	26.275	4.132	5.93	2.901	3.087	7.987	11.642
NODE_47868_length_1727_cov_28.326481_g23850_i1	30.113	14.417	0	0	3.058	2.738	19.193	9.355
NODE_49520_length_1687_cov_29.903346_g24837_i0	48.89	42.702	7.083	4.553	11.576	10.668	49.123	28.415
NODE_49912_length_1678_cov_19.519003_g25040_i0	97.675	114.867	23.348	10.897	1.913	2.016	16.539	5.014
NODE_53773_length_1588_cov_25.504950_g24584_i1	1.471	3.162	6.717	38.057	25.115	22.611	88.84	49.558
NODE_53928_length_1584_cov_32.868961_g20729_i1	36.009	61.259	16.087	19.92	9.855	13.087	133.547	53.781
NODE_56160_length_1537_cov_35.086066_g28317_i0	1.567	2.921	0	0	1.97	1.33	0	0
NODE_57073_length_1518_cov_36.331488_g28784_i0	27.552	44.363	12.074	6.344	4.871	4.555	45.99	10.176
NODE_58188_length_1495_cov_30.462729_g29370_i0	78.135	94.447	34.104	20.592	16.575	16.691	100.421	33.43
NODE_62237_length_1411_cov_37.038864_g31542_i0	236.873	339.442	102.796	87.151	27.483	30.331	98.54	69.059
NODE_71884_length_1237_cov_27.557560_g11942_i9	26.239	58.927	9.693	4.495	3.136	1.534	48.78	7.859
NODE_78906_length_1125_cov_27.942015_g41204_i0	15.231	132.955	9.744	4.346	2.24	1.787	189.157	24.163
NODE_80068_length_1107_cov_34.284333_g41882_i0	19.55	101.857	4.736	2.198	0.811	1.029	145.638	20.175
NODE_86474_length_1018_cov_43.617989_g45985_i0	352.795	529.382	229.06	155.253	11	9.398	69.416	41.435
NODE_892_length_7170_cov_29.649429_g481_i0	3.387	3.752	7.474	9.023	4.679	4.657	53.06	12.785
NODE_91831_length_950_cov_21.492588_g49470_i2	2.826	0.67	8.945	14.14	4.238	4.043	0.351	0

TableS3b.Blastx results for common DEGs

Contig729	sp_Q2RAK2_KPYC1_ORYSJ_Pyruvate_kinase_1,_cytosolic_OS=Oryza_sativa_subsp._japonica_OX=39947_GN=Os11g0148500_PE=1_SV=1
Contig914	sp_P34106_ALA2_PANMI_Alanine_aminotransferase_2_OS=Panicum_miliaceum_OX=4540_PE=1_SV=1
Contig3373	sp_Q6Q1P4_SMC1_ARATH_Structural_maintenance_of_chromosomes_protein_1_OS=Arabidopsis_thaliana_OX=3702_GN=SMC1_PE=2_SV=2
Contig3734	sp_F4J8G7_VIR_ARATH_Protein_virilizer_homolog_OS=Arabidopsis_thaliana_OX=3702_GN=VIR_PE=1_SV=1
Contig4764	sp_Q9LP56_WRK65_ARATH_Probable_WRKY_transcription_factor_65_OS=Arabidopsis_thaliana_OX=3702_GN=WRKY65_PE=2_SV=1
Contig5088	sp_A4GSN8_NUA_ARATH_Nuclear-pore_anchor_OS=Arabidopsis_thaliana_OX=3702_GN=NUA_PE=1_SV=1
Contig5792	sp_P24805_TSJT1_TOBAC_Stem-specific_protein_TSJT1_OS=Nicotiana_tabacum_OX=4097_GN=TSJT1_PE=2_SV=1
Contig7048	sp_P20026_MYB1_HORVU_Myb-related_protein_Hv1_OS=Hordeum_vulgare_OX=4513_GN=MYB1_PE=2_SV=1
Contig8508	sp_P43279_MAOC_ORYSJ_NADP-dependent_malic_enzyme,_chloroplastic_OS=Oryza_sativa_subsp._japonica_OX=39947_GN=ME6_PE=2_SV=2
Contig8714	sp_Q69UI2_RS131_ORYSJ_40S_ribosomal_protein_S13-1_OS=Oryza_sativa_subsp._japonica_OX=39947_GN=Os08g0117200_PE=3_SV=2
Contig10164	sp_B9FMJ3_KN13A_ORYSJ_Kinesin-like_protein_KIN-13A_OS=Oryza_sativa_subsp._japonica_OX=39947_GN=KIN13A_PE=1_SV=1
Contig11484	sp_Q6YZW0_ARFU_ORYSJ_Auxin_response_factor_21_OS=Oryza_sativa_subsp._japonica_OX=39947_GN=ARF21_PE=2_SV=2
Contig11505	sp_P93431_RCA_ORYSJ_Ribulose_bisphosphate_carboxylase/oxygenase_activase,_chloroplastic_OS=Oryza_sativa_subsp._japonica_OX=39947_GN=RCA_PE=1_SV=2
Contig11748	sp_Q10QA5_D14_ORYSJ_Strigolactone_esterase_D14_OS=Oryza_sativa_subsp._japonica_OX=39947_GN=D14_PE=1_SV=1
NODE_20942_length_2692_cov_27.568156_g9710_i1	sp_F4K5J1_MYO17_ARATH_Myosin-17_OS=Arabidopsis_thaliana_OX=3702_GN=XI-K_PE=1_SV=2
NODE_23067_length_2575_cov_35.130695_g1727_i4	sp_Q0D5W6_SUI1_ORYSJ_Protein_translation_factor_SUI1_homolog_OS=Oryza_sativa_subsp._japonica_OX=39947_GN=GOS2_PE=3_SV=1
NODE_23146_length_2571_cov_26.692954_g11564_i0	sp_F4I460_MYO8_ARATH_Myosin-8_OS=Arabidopsis_thaliana_OX=3702_GN=XI-B_PE=3_SV=1
NODE_26900_length_2392_cov_27.932730_g9198_i1	sp_Q9C8G5_CSCLD_ARATH_CSC1-like_protein_ERD4_OS=Arabidopsis_thaliana_OX=3702_GN=ERD4_PE=1_SV=1
NODE_30996_length_2232_cov_32.053265_g15531_i0	sp_F4IIM1_CSI1_ARATH_Protein_CELLULOSE_SYNTHASE_INTERACTIVE_1_OS=Arabidopsis_thaliana_OX=3702_GN=CSI1_PE=1_SV=1
NODE_37914_length_1994_cov_21.561166_g19016_i0	sp_Q9C942_CSE_ARATH_Caffeoylshikimate_esterase_OS=Arabidopsis_thaliana_OX=3702_GN=CSE_PE=1_SV=1
NODE_47868_length_1727_cov_28.326481_g23850_i1	sp_O22174_ERF08_ARATH_Ethylene-responsive_transcription_factor_ERF008_OS=Arabidopsis_thaliana_OX=3702_GN=ERF008_PE=2_SV=1
NODE_49520_length_1687_cov_29.903346_g24837_i0	sp_P20026_MYB1_HORVU_Myb-related_protein_Hv1_OS=Hordeum_vulgare_OX=4513_GN=MYB1_PE=2_SV=1
NODE_49912_length_1678_cov_19.519003_g25040_i0	sp_P81713_IBB3_WHEAT_Bowman-Birk_type_trypsin_inhibitor_OS=Triticum_aestivum_OX=4565_PE=1_SV=1
NODE_53928_length_1584_cov_32.868961_g20729_i1	sp_Q8W0E4_SAT1_ORYSJ_Probable_serine_acetyltransferase_1_OS=Oryza_sativa_subsp._japonica_OX=39947_GN=SAT1_PE=2_SV=1
NODE_56160_length_1537_cov_35.086066_g28317_i0	sp_Q8LF05_RBL19_ARATH_Rhomboid-like_protein_19_OS=Arabidopsis_thaliana_OX=3702_GN=RBL19_PE=2_SV=1
NODE_57073_length_1518_cov_36.331488_g28784_i0	sp_Q9LW50_ERF2_NICSY_Ethylene-responsive_transcription_factor_2_OS=Nicotiana_sylvestris_OX=4096_GN=ERF2_PE=2_SV=1
NODE_71884_length_1237_cov_27.557560_g11942_i9	sp_Q1WM16_HXK7_ORYSJ_Hexokinase-7_OS=Oryza_sativa_subsp._japonica_OX=39947_GN=HXK7_PE=2_SV=2
NODE_80068_length_1107_cov_34.284333_g41882_i0	sp_Q9LV60_CRR55_ARATH_Cysteine-rich_repeat_secretory_protein_55_OS=Arabidopsis_thaliana_OX=3702_GN=CRRSP55_PE=2_SV=1
NODE_86474_length_1018_cov_43.617989_g45985_i0	sp_Q9FJ95_DHSO_ARATH_Sorbitol_dehydrogenase_OS=Arabidopsis_thaliana_OX=3702_GN=SDH_PE=1_SV=1
NODE_91831_length_950_cov_21.492588_g49470_i2	sp_F4IN58_PIEZO_ARATH_Piezo-type_mechanosensitive_ion_channel_homolog_OS=Arabidopsis_thaliana_OX=3702_GN=At2g48060/At2g48040/At2g48050_PE=2_SV=1
NODE_113847_length_719_cov_32.125387_g65339_i0	sp_Q40477_ERF4_TOBAC_Ethylene-responsive_transcription_factor_4_OS=Nicotiana_tabacum_OX=4097_GN=ERF4_PE=1_SV=1
NODE_124392_length_632_cov_75.901610_g74075_i0	sp_P24805_TSJT1_TOBAC_Stem-specific_protein_TSJT1_OS=Nicotiana_tabacum_OX=4097_GN=TSJT1_PE=2_SV=1
BINPACKER_10495_1	sp_B5FZA8_LYRM4_TAEGU_LYR_motif-containing_protein_4_OS=Taeniopygia_guttata_OX=59729_GN=LYRM4_PE=3_SV=1
BINPACKER_10852_2	sp_Q9LZF8_PCRK2_ARATH_Serine/threonine-protein_kinase_PCRK2_OS=Arabidopsis_thaliana_OX=3702_GN=PCRK2_PE=1_SV=1
BINPACKER_12014_1	sp_P40468_TAO3_YEAST_Cell_morphogenesis_protein_PAG1_OS=Saccharomyces_cerevisiae_(strain_ATCC_204508_/_S288c)_OX=559292_GN=TAO3_PE=1_SV=1
BINPACKER_13772_2	sp_Q9FXI5_IQD32_ARATH_Protein_IQ-DOMAIN_32_OS=Arabidopsis_thaliana_OX=3702_GN=IQD32_PE=1_SV=3
BINPACKER_15897_5	sp_Q9FF10_HPSE1_ARATH_Heparanase-like_protein_1_OS=Arabidopsis_thaliana_OX=3702_GN=At5g07830_PE=2_SV=1
BINPACKER_16_3	sp_Q99070_GRP2_SORBI_Glycine-rich_RNA-binding_protein_2_OS=Sorghum_bicolor_OX=4558_GN=GRP2_PE=2_SV=1
BINPACKER_2461_5	sp_Q93YV6_2A5K_ARATH_Serine/threonine_protein_phosphatase_2A_57_kDa_regulatory_subunit_B'_kappa_isoform_OS=Arabidopsis_thaliana_OX=3702_GN=B'KAPPA_PE=2_SV=1
BINPACKER_2511_15	sp_Q84J55_14331_ORYSJ_14-3-3-like_protein_GF14-A_OS=Oryza_sativa_subsp._japonica_OX=39947_GN=GF14A_PE=2_SV=1
BINPACKER_6848_1	sp_P10330_NU5M_OENBE_NADH-ubiquinone_oxidoreductase_chain_5_OS=Oenothera_berteroana_OX=3950_GN=ND5_PE=2_SV=2
BINPACKER_7001_3	sp_Q09263_EFR3_CAEEL_Protein_EFR3_homolog_OS=Caenorhabditis_elegans_OX=6239_GN=efr-3_PE=3_SV=1
BINPACKER_99441_1	sp_Q9LJE5_ECT2_ARATH_YTH_domain-containing_protein_ECT2_OS=Arabidopsis_thaliana_OX=3702_GN=ECT2_PE=1_SV=1
NODE_892_length_7170_cov_29.649429_g481_i0	sp_Q9SR86_AGT23_ARATH_Alanine--glyoxylate_aminotransferase_2_homolog_3,_mitochondrial_OS=Arabidopsis_thaliana_OX=3702_GN=At3g08860_PE=2_SV=1
NODE_12121_length_3358_cov_22.675799_g6071_i0	sp_Q9M8S0_MBS1_ARATH_Protein_METHYLENE_BLUE_SENSITIVITY_1_OS=Arabidopsis_thaliana_OX=3702_GN=MBS1_PE=2_SV=1
NODE_17211_length_2932_cov_24.168940_g8539_i0	sp_Q9ZQG9_E1314_ARATH_Glucan_endo-1,3-beta-glucosidase_14_OS=Arabidopsis_thaliana_OX=3702_GN=At2g27500_PE=2_SV=2
NODE_17436_length_2912_cov_20.072561_g8651_i0	sp_Q9M884_MPI1_ARATH_Mannose-6-phosphate_isomerase_1_OS=Arabidopsis_thaliana_OX=3702_GN=PMI1_PE=1_SV=1
NODE_18844_length_2822_cov_22.405602_g9393_i0	sp_Q9M8L2_FBK30_ARATH_F-box/kelch-repeat_protein_At1g80440_OS=Arabidopsis_thaliana_OX=3702_GN=At1g80440_PE=2_SV=1
NODE_21453_length_2664_cov_22.591663_g10685_i0	sp_Q94A27_SAC2_ARATH_Phosphoinositide_phosphatase_SAC2_OS=Arabidopsis_thaliana_OX=3702_GN=SAC2_PE=2_SV=1
NODE_24015_length_2525_cov_30.757749_g12037_i0	sp_Q5VQ78_COB21_ORYSJ_Coatomer_subunit_beta'-1_OS=Oryza_sativa_subsp._japonica_OX=39947_GN=Os06g0143900_PE=2_SV=1
NODE_32549_length_2176_cov_31.285782_g16286_i0	sp_Q8L7F9_B3GTF_ARATH_Beta-1,3-galactosyltransferase_GALT1_OS=Arabidopsis_thaliana_OX=3702_GN=GALT1_PE=1_SV=1
NODE_42638_length_1859_cov_26.571109_g21306_i0	sp_P60707_ACTB_TRIVU_Actin,_cytoplasmic_1_OS=Trichosurus_vulpecula_OX=9337_GN=ACTB_PE=2_SV=1
NODE_53773_length_1588_cov_25.504950_g24584_i1	sp_Q5XF51_3MMP_ARATH_Metalloendoproteinase_3-MMP_OS=Arabidopsis_thaliana_OX=3702_GN=3MMP_PE=1_SV=1
NODE_62237_length_1411_cov_37.038864_g31542_i0	sp_Q94F47_UBC28_ARATH_Ubiquitin-conjugating_enzyme_E2_28_OS=Arabidopsis_thaliana_OX=3702_GN=UBC28_PE=1_SV=1
NODE_78906_length_1125_cov_27.942015_g41204_i0	sp_Q9LV60_CRR55_ARATH_Cysteine-rich_repeat_secretory_protein_55_OS=Arabidopsis_thaliana_OX=3702_GN=CRRSP55_PE=2_SV=1
BINPACKER_102_1	sp_K7U9N8_OP1_MAIZE_Protein_OPAQUE1_OS=Zea_mays_OX=4577_GN=O1_PE=1_SV=1
BINPACKER_14118_2	sp_P27572_NU4M_WHEAT_NADH-ubiquinone_oxidoreductase_chain_4_OS=Triticum_aestivum_OX=4565_GN=ND4_PE=2_SV=2
BINPACKER_15872_1	sp_A3B9A0_ARFP_ORYSJ_Auxin_response_factor_16_OS=Oryza_sativa_subsp._japonica_OX=39947_GN=ARF16_PE=2_SV=1
BINPACKER_15872_1	sp_A3B9A0_ARFP_ORYSJ_Auxin_response_factor_16_OS=Oryza_sativa_subsp._japonica_OX=39947_GN=ARF16_PE=2_SV=1
BINPACKER_17855_4	sp_Q9FYC2_PAO_ARATH_Pheophorbide_a_oxygenase,_chloroplastic_OS=Arabidopsis_thaliana_OX=3702_GN=PAO_PE=1_SV=1
BINPACKER_2187_1	sp_Q54IK2_TM120_DICDI_Transmembrane_protein_120_homolog_OS=Dictyostelium_discoideum_OX=44689_GN=tmem120_PE=3_SV=1
BINPACKER_23437_2	sp_Q9SFV2_FHA2_ARATH_FHA_domain-containing_protein_FHA2_OS=Arabidopsis_thaliana_OX=3702_GN=FHA2_PE=1_SV=1
BINPACKER_283_11	sp_P26517_G3PC1_HORVU_Glyceraldehyde-3-phosphate_dehydrogenase_1,_cytosolic_OS=Hordeum_vulgare_OX=4513_GN=GAPC_PE=2_SV=1
BINPACKER_637_6	sp_Q9FJ95_DHSO_ARATH_Sorbitol_dehydrogenase_OS=Arabidopsis_thaliana_OX=3702_GN=SDH_PE=1_SV=1
BINPACKER_7709_6	sp_Q0DX67_DSP2_ORYSJ_Probable_tyrosine-protein_phosphatase_DSP2_OS=Oryza_sativa_subsp._japonica_OX=39947_GN=DSP2_PE=2_SV=2
BINPACKER_8843_3	sp_Q93YS6_GTE9_ARATH_Transcription_factor_GTE9_OS=Arabidopsis_thaliana_OX=3702_GN=GTE9_PE=1_SV=1
