# Supplementary material for: De novo transcriptome assembly and analysis of Phragmites karka, an invasive halophyte, to study the mechanism of salinity stress tolerance
Source: Sci Rep. 2020 Mar 23;10:5192. doi: 10.1038/s41598-020-61857-8 (PMC7089983; doi:10.1038/s41598-020-61857-8)
Supplement: Supplementary file 5 — Supporting Information5. [file 41598_2020_61857_MOESM5_ESM.pdf]

Supplementary file 5: Comparison of gene expression levels as obtained in qRT PCR and by *in silico* analysis

Expression levels observed in qRT PCR analysis

Primers	Unigene ID	RQ (qRT PCR)	SD
control		1	0.007744622
CAT2	NODE_80072_length_1107_cov_30.905222_g41884_i0	5.2	0.5
IC5	NODE_41354_length_1894_cov_31.994509_g20670_i0	25.74106787	0.493
P3	BINPACKER_20431_2	0.068062313	0.118
STLR1	Contig5088	12.42680579	0.096957229
STLR12	BINPACKER_6848_1	0.245714635	0.643715858
STLR13	BINPACKER_7001_3	0.378684846	0.541842639
STLR14	NODE_18844_length_2822_cov_22.405602_g9393_i0	0.664892007	0.285720497
STLR15	BINPACKER_102_1	4.547957621	0.492187232
STLR18	BINPACKER_8843_3	2.081499064	0.158273518
STLR3	Contig7048	0.495141998	0.220038146
STLR7	NODE_47868_length_1727_cov_28.326481_g23850_i1	0.496772703	0.350721151
STLR8	NODE_91831_length_950_cov_21.492588_g49470_i2	1.21661056	0.332282662
STLR9	BINPACKER_10495_1	0.06459	0.107707985
STLR3	Contig7048	2.882572915	0.521
STLR7	NODE_47868_length_1727_cov_28.326481_g23850_i1	32.83400683	0.357

Expression levels observed in *in silico* analysis

	Unigene ID	Control_leaf1	Control_leaf2	Salt_treated_leaf1	Salt_treated_leaf2
CAT2	NODE_80072_length_1107_cov_30.905222_g41884_i0	0	0	0	1.393
IC5	NODE_41354_length_1894_cov_31.994509_g20670_i0	2.117	1.353	4.651	4.163
P3	BINPACKER_20431_2	73.669	0.375	45.268	0.232
STLR1	Contig5088	1.143	0.348	7.771	5.896
STLR12	BINPACKER_6848_1	2.667	5.212	1.063	0.24
STLR13	BINPACKER_7001_3	1.037	1.125	0	0
STLR14	NODE_18844_length_2822_cov_22.405602_g9393_i0	30.664	45.582	15.611	9.429
STLR15	BINPACKER_102_1	3.154	1.661	7.754	8.592
STLR18	BINPACKER_8843_3	0	0	1.275	7.389
STLR3	Contig7048	20.397	14.966	3.461	0.929
STLR7	NODE_47868_length_1727_cov_28.326481_g23850_i1	30.113	14.417	0	0
STLR8	NODE_91831_length_950_cov_21.492588_g49470_i2	2.826	0.67	8.945	14.14
STLR9	BINPACKER_10495_1	29.764	46.051	11.997	11.892

	Unigene ID	Control_root1	Control_root2	Salt_treated_root1	Salt_treated_root2
STLR3	Contig7048	5.433	4.693	19.01	12.228
STLR7	NODE_47868_length_1727_cov_28.326481_g23850_i1	3.058	2.738	19.193	9.355
