# Supplementary material for: De novo transcriptome assembly and analysis of Phragmites karka, an invasive halophyte, to study the mechanism of salinity stress tolerance
Source: Sci Rep. 2020 Mar 23;10:5192. doi: 10.1038/s41598-020-61857-8 (PMC7089983; doi:10.1038/s41598-020-61857-8)
Supplement: Supplementary file 6 — Supporting Information6. [file 41598_2020_61857_MOESM6_ESM.pdf]

TableS5. Differentially expressed unigenes containing SSRs and their Uniprot ID

NODE_875_length_7193_cov_31.008567_g0_i26	sp_A1E9S3_PSAB_SORBI_Photosystem_I_P700_chlorophyll_a_apoprotein_A2_OS=Sorghum_bicolor_OX=4558_GN=psaB_PE=3_SV=1
NODE_89_length_12429_cov_28.028488_g43_i0	sp_A1E9T7_CYF_SORBI_Cytochrome_f_OS=Sorghum_bicolor_OX=4558_GN=petA_PE=3_SV=1
NODE_38671_length_1969_cov_28.831224_g19395_i0	sp_A1E9V0_PSBB_SORBI_Photosystem_II_CP47_reaction_center_protein_OS=Sorghum_bicolor_OX=4558_GN=psbB_PE=3_SV=1
BINPACKER_376_1	sp_A1E9X3_CCSA_SORBI_Cytochrome_c_biogenesis_protein_CcsA_OS=Sorghum_bicolor_OX=4558_GN=ccsA_PE=3_SV=1
BINPACKER_1841_16	sp_A2WZ60_IRO2_ORYSI_Protein_IRON-RELATED_TRANSCRIPTION_FACTOR_2_OS=Oryza_sativa_subsp._indica_OX=39946_GN=IRO2_PE=3_SV=1
BINPACKER_370_1	sp_A2XFB7_PRR73_ORYSI_Two-component_response_regulator-like_PRR73_OS=Oryza_sativa_subsp._indica_OX=39946_GN=PRR73_PE=2_SV=2
NODE_32522_length_2177_cov_29.379278_g16272_i0	sp_A2XFI3_PDC2_ORYSI_Pyruvate_decarboxylase_2_OS=Oryza_sativa_subsp._indica_OX=39946_GN=PDC2_PE=2_SV=2
NODE_111403_length_742_cov_22.579970_g9088_i5	sp_A3BI11_ZIP8_ORYSJ_Zinc_transporter_8_OS=Oryza_sativa_subsp._japonica_OX=39947_GN=ZIP8_PE=2_SV=1
NODE_53281_length_1598_cov_35.158033_g26811_i0	sp_A3C6D7_ACT2_ORYSJ_Actin-2_OS=Oryza_sativa_subsp._japonica_OX=39947_GN=ACT2_PE=2_SV=1
BINPACKER_11502_9	sp_A7PZL3_PGLR_VITVI_Probable_polygalacturonase_OS=Vitis_vinifera_OX=29760_GN=GSVIVT00026920001_PE=1_SV=1
BINPACKER_8269_4	sp_B5BT18_BTAF1_ARATH_TATA-binding_protein-associated_factor_BTAF1_OS=Arabidopsis_thaliana_OX=3702_GN=BTAF1_PE=1_SV=1
BINPACKER_8269_5	sp_B5BT18_BTAF1_ARATH_TATA-binding_protein-associated_factor_BTAF1_OS=Arabidopsis_thaliana_OX=3702_GN=BTAF1_PE=1_SV=1
BINPACKER_10495_1	sp_B5FZA8_LYRM4_TAEGU_LYR_motif-containing_protein_4_OS=Taeniopygia_guttata_OX=59729_GN=LYRM4_PE=3_SV=1
BINPACKER_5362_1	sp_B6TB21_DRE2_MAIZE_Anamorsin_homolog_OS=Zea_mays_OX=4577_PE=2_SV=1
BINPACKER_4_4	sp_B9FK36_ACC2_ORYSJ_Acetyl-CoA_carboxylase_2_OS=Oryza_sativa_subsp._japonica_OX=39947_GN=ACC2_PE=3_SV=2
NODE_56108_length_1539_cov_9.210778_g28293_i0	sp_C0LGG3_Y5182_ARATH_Probable_LRR_receptor-like_serine/threonine-protein_kinase_At1g51820_OS=Arabidopsis_thaliana_OX=3702_GN=At1g51820_PE=1_SV=1
NODE_9712_length_3615_cov_22.990966_g4909_i0	sp_C0LGH2_Y1561_ARATH_Probable_LRR_receptor-like_serine/threonine-protein_kinase_At1g56130_OS=Arabidopsis_thaliana_OX=3702_GN=At1g56130_PE=2_SV=2
BINPACKER_11165_1	sp_C0LGJ1_Y1743_ARATH_Probable_LRR_receptor-like_serine/threonine-protein_kinase_At1g74360_OS=Arabidopsis_thaliana_OX=3702_GN=At1g74360_PE=1_SV=1
BINPACKER_5267_1	sp_C7G304_GOLS2_SOLLC_Galactinol_synthase_2_OS=Solanum_lycopersicum_OX=4081_GN=GOLS2_PE=2_SV=1
BINPACKER_2690_19	sp_D7U6G6_ANRPN_VITVI_Anthocyanidin_reductase_((2S)-flavan-3-ol-forming)_OS=Vitis_vinifera_OX=29760_GN=ANR_PE=3_SV=1
BINPACKER_375_7	sp_F4HRS2_SWTIE_ARATH_Protein_SWEETIE_OS=Arabidopsis_thaliana_OX=3702_GN=SWEETIE_PE=1_SV=2
NODE_8149_length_3844_cov_22.826836_g4104_i0	sp_F4HRS2_SWTIE_ARATH_Protein_SWEETIE_OS=Arabidopsis_thaliana_OX=3702_GN=SWEETIE_PE=1_SV=2
Contig12375	sp_F4HZB2_BCHA1_ARATH_Protein_SPIRRIG_OS=Arabidopsis_thaliana_OX=3702_GN=SPI_PE=1_SV=1
NODE_3386_length_5048_cov_33.671156_g1737_i0	sp_F4HZB5_NET1D_ARATH_Protein_NETWORKED_1D_OS=Arabidopsis_thaliana_OX=3702_GN=NET1D_PE=3_SV=1
BINPACKER_14303_1	sp_F4I114_Y1960_ARATH_Probable_serine/threonine-protein_kinase_At1g09600_OS=Arabidopsis_thaliana_OX=3702_GN=At1g09600_PE=3_SV=1
BINPACKER_2723_1	sp_F4I4P3_MED23_ARATH_Mediator_of_RNA_polymerase_II_transcription_subunit_23_OS=Arabidopsis_thaliana_OX=3702_GN=MED23_PE=1_SV=1
BINPACKER_6268_1	sp_F4I893_ILA_ARATH_Protein_ILITYHIA_OS=Arabidopsis_thaliana_OX=3702_GN=ILA_PE=1_SV=1
NODE_43576_length_1834_cov_31.222033_g21802_i0	sp_F4I893_ILA_ARATH_Protein_ILITYHIA_OS=Arabidopsis_thaliana_OX=3702_GN=ILA_PE=1_SV=1
NODE_209_length_10332_cov_31.315820_g108_i0	sp_F4I9T0_BCHB_ARATH_BEACH_domain-containing_protein_B_OS=Arabidopsis_thaliana_OX=3702_GN=BCHB_PE=4_SV=1
NODE_113_length_11949_cov_23.605760_g53_i0	sp_F4IAT2_THOC2_ARATH_THO_complex_subunit_2_OS=Arabidopsis_thaliana_OX=3702_GN=THO2_PE=1_SV=1
BINPACKER_2161_4	sp_F4ICK8_IWS1_ARATH_Protein_IWS1_homolog_1_OS=Arabidopsis_thaliana_OX=3702_GN=IWS1_PE=1_SV=1
NODE_218_length_10247_cov_31.183900_g113_i0	sp_F4IG73_BCHC2_ARATH_BEACH_domain-containing_protein_C2_OS=Arabidopsis_thaliana_OX=3702_GN=BCHC2_PE=1_SV=1
BINPACKER_555_10	sp_F4IHS2_SYD_ARATH_Chromatin_structure-remodeling_complex_protein_SYD_OS=Arabidopsis_thaliana_OX=3702_GN=SYD_PE=1_SV=1
Contig8351	sp_F4IN58_PIEZO_ARATH_Piezo-type_mechanosensitive_ion_channel_homolog_OS=Arabidopsis_thaliana_OX=3702_GN=At2g48060/At2g48040/At2g48050_PE=2_SV=1
BINPACKER_33_2	sp_F4IRU3_MYO12_ARATH_Myosin-12_OS=Arabidopsis_thaliana_OX=3702_GN=XI-F_PE=2_SV=1
NODE_4725_length_4590_cov_24.807173_g1300_i1	sp_F4IS91_RSA1_ARATH_Protein_SHORT_ROOT_IN_SALT_MEDIUM_1_OS=Arabidopsis_thaliana_OX=3702_GN=RSA1_PE=1_SV=1
NODE_3096_length_5171_cov_30.269714_g1045_i1	sp_F4IVL6_GRV2_ARATH_DnaJ_homolog_subfamily_C_GRV2_OS=Arabidopsis_thaliana_OX=3702_GN=GRV2_PE=1_SV=1
BINPACKER_7774_3	sp_F4IXW2_BIG5_ARATH_Brefeldin_A-inhibited_guanine_nucleotide-exchange_protein_5_OS=Arabidopsis_thaliana_OX=3702_GN=BIG5_PE=1_SV=2
NODE_56911_length_1522_cov_28.762595_g28691_i0	sp_F4J6F6_IREH1_ARATH_Probable_serine/threonine_protein_kinase_IREH1_OS=Arabidopsis_thaliana_OX=3702_GN=IREH1_PE=1_SV=1
Contig3734	sp_F4J8G7_VIR_ARATH_Protein_virilizer_homolog_OS=Arabidopsis_thaliana_OX=3702_GN=VIR_PE=1_SV=1
NODE_23493_length_2553_cov_27.755242_g11735_i0	sp_F4J9M5_CHR12_ARATH_Probable_ATP-dependent_DNA_helicase_CHR12_OS=Arabidopsis_thaliana_OX=3702_GN=CHR12_PE=2_SV=1
NODE_73394_length_1212_cov_40.580334_g37844_i0	sp_F4JFN3_HS906_ARATH_Heat_shock_protein_90-6,_mitochondrial_OS=Arabidopsis_thaliana_OX=3702_GN=HSP90-6_PE=1_SV=1
BINPACKER_1743_1	sp_F4JGZ1_MED16_ARATH_Mediator_of_RNA_polymerase_II_transcription_subunit_16_OS=Arabidopsis_thaliana_OX=3702_GN=MED16_PE=1_SV=1
NODE_3954_length_4848_cov_26.911623_g1991_i0	sp_F4JKH6_TSS_ARATH_Protein_TSS_OS=Arabidopsis_thaliana_OX=3702_GN=TSS_PE=1_SV=1
NODE_5798_length_4289_cov_26.403463_g2737_i2	sp_F4JKH6_TSS_ARATH_Protein_TSS_OS=Arabidopsis_thaliana_OX=3702_GN=TSS_PE=1_SV=1
Contig4402	sp_F4JSZ5_BIG1_ARATH_Brefeldin_A-inhibited_guanine_nucleotide-exchange_protein_1_OS=Arabidopsis_thaliana_OX=3702_GN=BIG1_PE=2_SV=1
NODE_26086_length_2427_cov_25.486831_g12875_i1	sp_F4JVA6_SFH6_ARATH_Phosphatidylinositol/phosphatidylcholine_transfer_protein_SFH6_OS=Arabidopsis_thaliana_OX=3702_GN=SFH6_PE=2_SV=1
NODE_3061_length_5189_cov_26.893276_g1569_i0	sp_F4K5J1_MYO17_ARATH_Myosin-17_OS=Arabidopsis_thaliana_OX=3702_GN=XI-K_PE=1_SV=2
NODE_6290_length_4175_cov_30.597513_g3185_i0	sp_F4KBP5_CHR4_ARATH_Protein_CHROMATIN_REMODELING_4_OS=Arabidopsis_thaliana_OX=3702_GN=CHR4_PE=2_SV=1
NODE_4125_length_4781_cov_24.155055_g2082_i0	sp_F4KCC2_PRT6_ARATH_E3_ubiquitin-protein_ligase_PRT6_OS=Arabidopsis_thaliana_OX=3702_GN=PRT6_PE=2_SV=1
BINPACKER_2578_1	sp_H3K2Y6_MED12_ARATH_Mediator_of_RNA_polymerase_II_transcription_subunit_12_OS=Arabidopsis_thaliana_OX=3702_GN=MED12_PE=1_SV=1
BINPACKER_2578_4	sp_H3K2Y6_MED12_ARATH_Mediator_of_RNA_polymerase_II_transcription_subunit_12_OS=Arabidopsis_thaliana_OX=3702_GN=MED12_PE=1_SV=1
NODE_17825_length_2884_cov_22.620064_g8859_i0	sp_K7U9N8_OP1_MAIZE_Protein_OPAQUE1_OS=Zea_mays_OX=4577_GN=O1_PE=1_SV=1
Contig7050	sp_O22174_ERF08_ARATH_Ethylene-responsive_transcription_factor_ERF008_OS=Arabidopsis_thaliana_OX=3702_GN=ERF008_PE=2_SV=1
NODE_47868_length_1727_cov_28.326481_g23850_i1	sp_O22174_ERF08_ARATH_Ethylene-responsive_transcription_factor_ERF008_OS=Arabidopsis_thaliana_OX=3702_GN=ERF008_PE=2_SV=1
BINPACKER_36955_3	sp_O22259_ERF71_ARATH_Ethylene-responsive_transcription_factor_ERF071_OS=Arabidopsis_thaliana_OX=3702_GN=ERF071_PE=2_SV=1
Contig11604	sp_O22898_LACS1_ARATH_Long_chain_acyl-CoA_synthetase_1_OS=Arabidopsis_thaliana_OX=3702_GN=LACS1_PE=2_SV=1
BINPACKER_29842_1	sp_O22941_IDE1_ARATH_Insulin-degrading_enzyme-like_1,_peroxisomal_OS=Arabidopsis_thaliana_OX=3702_GN=PXM16_PE=2_SV=1
BINPACKER_17773_3	sp_O23461_ARAK_ARATH_L-arabinokinase_OS=Arabidopsis_thaliana_OX=3702_GN=ARA1_PE=1_SV=1
Contig3905	sp_O48847_LUH_ARATH_Transcriptional_corepressor_LEUNIG_HOMOLOG_OS=Arabidopsis_thaliana_OX=3702_GN=LUH_PE=1_SV=1
BINPACKER_4386_3	sp_O64728_STI_ARATH_Protein_STICHEL_OS=Arabidopsis_thaliana_OX=3702_GN=STI_PE=1_SV=2
BINPACKER_643_1	sp_O64818_Y2309_ARATH_Uncharacterized_protein_At2g23090_OS=Arabidopsis_thaliana_OX=3702_GN=At2g23090_PE=1_SV=1
NODE_88501_length_992_cov_23.681175_g47272_i0	sp_O64937_EF1A_ORYSJ_Elongation_factor_1-alpha_OS=Oryza_sativa_subsp._japonica_OX=39947_GN=REFA1_PE=2_SV=2
NODE_10302_length_3543_cov_33.529683_g5205_i0	sp_O65238_Y5537_ARATH_G-type_lectin_S-receptor-like_serine/threonine-protein_kinase_At5g35370_OS=Arabidopsis_thaliana_OX=3702_GN=At5g35370_PE=2_SV=2
BINPACKER_3224_11	sp_O65717_CNGC1_ARATH_Cyclic_nucleotide-gated_ion_channel_1_OS=Arabidopsis_thaliana_OX=3702_GN=CNGC1_PE=1_SV=1
NODE_17897_length_2879_cov_32.516393_g8889_i0	sp_O80738_TPS10_ARATH_Probable_alpha,alpha-trehalose-phosphate_synthase_[UDP-forming]_10_OS=Arabidopsis_thaliana_OX=3702_GN=TPS10_PE=2_SV=1
BINPACKER_10217_1	sp_O80920_PYL4_ARATH_Abscisic_acid_receptor_PYL4_OS=Arabidopsis_thaliana_OX=3702_GN=PYL4_PE=1_SV=1
NODE_74558_length_1193_cov_29.506250_g38535_i0	sp_O80920_PYL4_ARATH_Abscisic_acid_receptor_PYL4_OS=Arabidopsis_thaliana_OX=3702_GN=PYL4_PE=1_SV=1
BINPACKER_17244_3	sp_O81905_SD18_ARATH_Receptor-like_serine/threonine-protein_kinase_SD1-8_OS=Arabidopsis_thaliana_OX=3702_GN=SD18_PE=1_SV=1
NODE_20457_length_2721_cov_17.584970_g10187_i0	sp_O81905_SD18_ARATH_Receptor-like_serine/threonine-protein_kinase_SD1-8_OS=Arabidopsis_thaliana_OX=3702_GN=SD18_PE=1_SV=1
NODE_16405_length_2994_cov_13.841835_g8146_i0	sp_O81906_B120_ARATH_G-type_lectin_S-receptor-like_serine/threonine-protein_kinase_B120_OS=Arabidopsis_thaliana_OX=3702_GN=B120_PE=2_SV=1
NODE_44244_length_1815_cov_24.769805_g22151_i0	sp_P00333_ADH1_MAIZE_Alcohol_dehydrogenase_1_OS=Zea_mays_OX=4577_GN=ADH1_PE=2_SV=1
NODE_45189_length_1791_cov_24.700815_g22652_i0	sp_P05642_CYB6_MAIZE_Cytochrome_b6_OS=Zea_mays_OX=4577_GN=petB_PE=2_SV=2
BINPACKER_21195_1	sp_P08926_RUBA_PEA_RuBisCO_large_subunit-binding_protein_subunit_alpha,_chloroplastic_OS=Pisum_sativum_OX=3888_PE=1_SV=2
NODE_28289_length_2334_cov_31.355595_g12058_i1	sp_P09189_HSP7C_PETHY_Heat_shock_cognate_70_kDa_protein_OS=Petunia_hybrida_OX=4102_GN=HSP70_PE=2_SV=1
BINPACKER_11352_4	sp_P0C0M2_GH32_ORYSJ_Probable_indole-3-acetic_acid-amido_synthetase_GH3.2_OS=Oryza_sativa_subsp._japonica_OX=39947_GN=GH3.2_PE=2_SV=1
NODE_10112_length_3563_cov_34.381375_g5108_i0	sp_P0C314_CLPP_ORYSJ_ATP-dependent_Clp_protease_proteolytic_subunit_OS=Oryza_sativa_subsp._japonica_OX=39947_GN=clpP_PE=3_SV=1
BINPACKER_41932_1	sp_P0C319_PETD_ORYSJ_Cytochrome_b6-f_complex_subunit_4_OS=Oryza_sativa_subsp._japonica_OX=39947_GN=petD_PE=3_SV=1
BINPACKER_335_7	sp_P0C355_PSAA_ORYSJ_Photosystem_I_P700_chlorophyll_a_apoprotein_A1_OS=Oryza_sativa_subsp._japonica_OX=39947_GN=psaA_PE=3_SV=1
NODE_29172_length_2300_cov_26.084868_g14640_i0	sp_P0C540_ACT7_ORYSJ_Actin-7_OS=Oryza_sativa_subsp._japonica_OX=39947_GN=ACT7_PE=3_SV=1
BINPACKER_9419_2	sp_P0DM58_DI3L2_ARATH_DIS3-like_exonuclease_2_OS=Arabidopsis_thaliana_OX=3702_GN=SOV_PE=1_SV=1
BINPACKER_9778_1	sp_P11734_CU08_LOCMI_Cuticle_protein_8_OS=Locusta_migratoria_OX=7004_PE=1_SV=1
NODE_65062_length_1358_cov_111.919066_g27862_i51	sp_P12329_CB21_MAIZE_Chlorophyll_a-b_binding_protein_1,_chloroplastic_OS=Zea_mays_OX=4577_GN=CAB1_PE=1_SV=1
NODE_64957_length_1360_cov_27.984460_g32998_i0	sp_P17703_RR15_MAIZE_30S_ribosomal_protein_S15,_chloroplastic_OS=Zea_mays_OX=4577_GN=rps15-A_PE=3_SV=1
NODE_3214_length_5115_cov_36.923840_g0_i32	sp_P17933_RR2_WHEAT_30S_ribosomal_protein_S2,_chloroplastic_OS=Triticum_aestivum_OX=4565_GN=rps2_PE=3_SV=2
Contig7048	sp_P20026_MYB1_HORVU_Myb-related_protein_Hv1_OS=Hordeum_vulgare_OX=4513_GN=MYB1_PE=2_SV=1
NODE_49520_length_1687_cov_29.903346_g24837_i0	sp_P20026_MYB1_HORVU_Myb-related_protein_Hv1_OS=Hordeum_vulgare_OX=4513_GN=MYB1_PE=2_SV=1
NODE_15365_length_3072_cov_30.894632_g7657_i0	sp_P24794_COX1_BETVU_Cytochrome_c_oxidase_subunit_1_OS=Beta_vulgaris_OX=161934_GN=COX1_PE=3_SV=2
BINPACKER_41013_1	sp_P24805_TSJT1_TOBAC_Stem-specific_protein_TSJT1_OS=Nicotiana_tabacum_OX=4097_GN=TSJT1_PE=2_SV=1
Contig5127	sp_P25776_ORYA_ORYSJ_Oryzain_alpha_chain_OS=Oryza_sativa_subsp._japonica_OX=39947_GN=Os04g0650000_PE=1_SV=2
BINPACKER_283_11	sp_P26517_G3PC1_HORVU_Glyceraldehyde-3-phosphate_dehydrogenase_1,_cytosolic_OS=Hordeum_vulgare_OX=4513_GN=GAPC_PE=2_SV=1
BINPACKER_9470_2	sp_P32456_GBP2_HUMAN_Guanylate-binding_protein_2_OS=Homo_sapiens_OX=9606_GN=GBP2_PE=1_SV=3
BINPACKER_631_6	sp_P33679_ZEAM_MAIZE_Zeamatin_OS=Zea_mays_OX=4577_GN=Zlp_PE=1_SV=2
Contig12297	sp_P35684_RL3_ORYSJ_60S_ribosomal_protein_L3_OS=Oryza_sativa_subsp._japonica_OX=39947_GN=RPL3_PE=2_SV=2
BINPACKER_156_2	sp_P37220_ASR3_SOLLC_Abscisic_stress-ripening_protein_3_OS=Solanum_lycopersicum_OX=4081_GN=ASR3_PE=3_SV=2
BINPACKER_156_4	sp_P37220_ASR3_SOLLC_Abscisic_stress-ripening_protein_3_OS=Solanum_lycopersicum_OX=4081_GN=ASR3_PE=3_SV=2
BINPACKER_10650_1	sp_P37891_CBP3_ORYSJ_Serine_carboxypeptidase_3_OS=Oryza_sativa_subsp._japonica_OX=39947_GN=CBP3_PE=2_SV=1
NODE_49890_length_1678_cov_29.505296_g25025_i0	sp_P37891_CBP3_ORYSJ_Serine_carboxypeptidase_3_OS=Oryza_sativa_subsp._japonica_OX=39947_GN=CBP3_PE=2_SV=1
BINPACKER_20985_1	sp_P40340_TBP7_YEAST_Tat-binding_homolog_7_OS=Saccharomyces_cerevisiae_(strain_ATCC_204508_/_S288c)_OX=559292_GN=YTA7_PE=1_SV=2
BINPACKER_12014_1	sp_P40468_TAO3_YEAST_Cell_morphogenesis_protein_PAG1_OS=Saccharomyces_cerevisiae_(strain_ATCC_204508_/_S288c)_OX=559292_GN=TAO3_PE=1_SV=1
Contig8508	sp_P43279_MAOC_ORYSJ_NADP-dependent_malic_enzyme,_chloroplastic_OS=Oryza_sativa_subsp._japonica_OX=39947_GN=ME6_PE=2_SV=2
NODE_248_length_9957_cov_29.699110_g132_i0	sp_P46620_NU5C_MAIZE_NAD(P)H-quinone_oxidoreductase_subunit_5,_chloroplastic_OS=Zea_mays_OX=4577_GN=ndhF_PE=3_SV=1
BINPACKER_29308_1	sp_P48979_PGLR_PRUPE_Polygalacturonase_OS=Prunus_persica_OX=3760_PE=2_SV=1
BINPACKER_7519_3	sp_P49608_ACOC_CUCMA_Aconitate_hydratase,_cytoplasmic_OS=Cucurbita_maxima_OX=3661_PE=2_SV=1
NODE_38485_length_1976_cov_17.818182_g19286_i0	sp_P52420_PUR2_ARATH_Phosphoribosylamine--glycine_ligase,_chloroplastic_OS=Arabidopsis_thaliana_OX=3702_GN=PUR2_PE=2_SV=2
NODE_93922_length_925_cov_17.579812_g50871_i0	sp_P52839_SOT12_ARATH_Cytosolic_sulfotransferase_12_OS=Arabidopsis_thaliana_OX=3702_GN=SOT12_PE=1_SV=2
NODE_113402_length_723_cov_34.736923_g64977_i0	sp_P52877_SERC_SPIOL_Phosphoserine_aminotransferase,_chloroplastic_OS=Spinacia_oleracea_OX=3562_PE=2_SV=1
NODE_18736_length_2829_cov_25.564224_g9340_i0	sp_P53393_SUT3_STYHA_Low_affinity_sulfate_transporter_3_OS=Stylosanthes_hamata_OX=37660_GN=ST3_PE=2_SV=1
NODE_47086_length_1745_cov_24.009569_g23611_i0	sp_P54797_TNG2_MOUSE_Transport_and_Golgi_organization_2_homolog_OS=Mus_musculus_OX=10090_GN=Tango2_PE=1_SV=1
NODE_107160_length_783_cov_28.885915_g60065_i0	sp_P54873_HMCS_ARATH_Hydroxymethylglutaryl-CoA_synthase_OS=Arabidopsis_thaliana_OX=3702_GN=HMGS_PE=1_SV=2
NODE_113929_length_719_cov_4.821981_g65401_i0	sp_P59082_LFS_ALLCE_Lachrymatory-factor_synthase_OS=Allium_cepa_OX=4679_GN=LFS_PE=1_SV=1
NODE_47440_length_1736_cov_29.565845_g23787_i0	sp_P68105_EF1A1_RABIT_Elongation_factor_1-alpha_1_OS=Oryctolagus_cuniculus_OX=9986_GN=EEF1A1_PE=1_SV=1
NODE_49912_length_1678_cov_19.519003_g25040_i0	sp_P81713_IBB3_WHEAT_Bowman-Birk_type_trypsin_inhibitor_OS=Triticum_aestivum_OX=4565_PE=1_SV=1
NODE_80759_length_1097_cov_28.265625_g42299_i0	sp_P81713_IBB3_WHEAT_Bowman-Birk_type_trypsin_inhibitor_OS=Triticum_aestivum_OX=4565_PE=1_SV=1
BINPACKER_4611_8	sp_P85193_PK01_HELAN_Putative_serine/threonine-protein_kinase_(Fragment)_OS=Helianthus_annuus_OX=4232_PE=1_SV=1
BINPACKER_224_2	sp_P93647_LONP2_MAIZE_Lon_protease_homolog_2,_peroxisomal_OS=Zea_mays_OX=4577_GN=LON1_PE=2_SV=1
NODE_72811_length_1222_cov_21.645779_g37489_i0	sp_Q02921_NO93_SOYBN_Early_nodulin-93_OS=Glycine_max_OX=3847_PE=2_SV=1
NODE_101460_length_840_cov_279.384615_g56022_i0	sp_Q03663_GSTX2_TOBAC_Probable_glutathione_S-transferase_OS=Nicotiana_tabacum_OX=4097_PE=2_SV=1
NODE_27431_length_2369_cov_31.353223_g13755_i0	sp_Q05085_PTR7_ARATH_Protein_NRT1/_PTR_FAMILY_6.3_OS=Arabidopsis_thaliana_OX=3702_GN=NPF6.3_PE=1_SV=1
NODE_71646_length_1241_cov_29.677226_g36820_i0	sp_Q06398_GSTU6_ORYSJ_Probable_glutathione_S-transferase_GSTU6_OS=Oryza_sativa_subsp._japonica_OX=39947_GN=GSTU6_PE=2_SV=2
NODE_17429_length_2912_cov_32.642480_g8647_i0	sp_Q06572_AVP_HORVU_Pyrophosphate-energized_vacuolar_membrane_proton_pump_OS=Hordeum_vulgare_OX=4513_PE=2_SV=2
NODE_18783_length_2826_cov_15.782056_g9362_i0	sp_Q06572_AVP_HORVU_Pyrophosphate-energized_vacuolar_membrane_proton_pump_OS=Hordeum_vulgare_OX=4513_PE=2_SV=2
BINPACKER_7001_3	sp_Q09263_EFR3_CAEEL_Protein_EFR3_homolog_OS=Caenorhabditis_elegans_OX=6239_GN=efr-3_PE=3_SV=1
NODE_19991_length_2752_cov_27.438223_g9945_i0	sp_Q0D3F2_NAS3_ORYSJ_Nicotianamine_synthase_3_OS=Oryza_sativa_subsp._japonica_OX=39947_GN=NAS3_PE=2_SV=1
BINPACKER_15729_1	sp_Q0D5W6_SUI1_ORYSJ_Protein_translation_factor_SUI1_homolog_OS=Oryza_sativa_subsp._japonica_OX=39947_GN=GOS2_PE=3_SV=1
NODE_23067_length_2575_cov_35.130695_g1727_i4	sp_Q0D5W6_SUI1_ORYSJ_Protein_translation_factor_SUI1_homolog_OS=Oryza_sativa_subsp._japonica_OX=39947_GN=GOS2_PE=3_SV=1
BINPACKER_15159_4	sp_Q0DJS1_TOR_ORYSJ_Serine/threonine-protein_kinase_TOR_OS=Oryza_sativa_subsp._japonica_OX=39947_GN=TOR_PE=1_SV=3
Contig11185	sp_Q0DJS1_TOR_ORYSJ_Serine/threonine-protein_kinase_TOR_OS=Oryza_sativa_subsp._japonica_OX=39947_GN=TOR_PE=1_SV=3
BINPACKER_18589_1	sp_Q0E3C8_CLPB3_ORYSJ_Chaperone_protein_ClpB3,_mitochondrial_OS=Oryza_sativa_subsp._japonica_OX=39947_GN=CLPB3_PE=2_SV=3
BINPACKER_3264_2	sp_Q0IQN5_RTOR2_ORYSJ_Regulatory-associated_protein_of_TOR_2_OS=Oryza_sativa_subsp._japonica_OX=39947_GN=RAPTOR2_PE=1_SV=2
BINPACKER_7850_20	sp_Q0J954_PAO5_ORYSJ_Polyamine_oxidase_5_OS=Oryza_sativa_subsp._japonica_OX=39947_GN=PAO5_PE=1_SV=1
BINPACKER_11298_1	sp_Q0JDM0_C3H27_ORYSJ_Zinc_finger_CCCH_domain-containing_protein_27_OS=Oryza_sativa_subsp._japonica_OX=39947_GN=Os04g0394300_PE=2_SV=2
BINPACKER_11298_4	sp_Q0JDM0_C3H27_ORYSJ_Zinc_finger_CCCH_domain-containing_protein_27_OS=Oryza_sativa_subsp._japonica_OX=39947_GN=Os04g0394300_PE=2_SV=2
NODE_19050_length_2809_cov_29.687500_g6389_i1	sp_Q0JKD0_GLT1_ORYSJ_Glutamate_synthase_1_[NADH],_chloroplastic_OS=Oryza_sativa_subsp._japonica_OX=39947_GN=Os01g0681900_PE=2_SV=1
NODE_96083_length_899_cov_28.601695_g52331_i0	sp_Q0JKD0_GLT1_ORYSJ_Glutamate_synthase_1_[NADH],_chloroplastic_OS=Oryza_sativa_subsp._japonica_OX=39947_GN=Os01g0681900_PE=2_SV=1
BINPACKER_3826_4	sp_Q0WP01_PTR9_ARATH_Protein_NRT1/_PTR_FAMILY_5.10_OS=Arabidopsis_thaliana_OX=3702_GN=NPF5.10_PE=2_SV=1
Contig214	sp_Q0WPK4_PAT1H_ARATH_Protein_PAT1_homolog_OS=Arabidopsis_thaliana_OX=3702_GN=PAT1_PE=1_SV=1
BINPACKER_12784_3	sp_Q0WSY2_FPP4_ARATH_Filament-like_plant_protein_4_OS=Arabidopsis_thaliana_OX=3702_GN=FPP4_PE=1_SV=1
NODE_35536_length_2071_cov_26.419920_g17817_i0	sp_Q0WSY2_FPP4_ARATH_Filament-like_plant_protein_4_OS=Arabidopsis_thaliana_OX=3702_GN=FPP4_PE=1_SV=1
BINPACKER_1164_1	sp_Q0WVE9_PLST1_ARATH_Probable_plastidic_glucose_transporter_1_OS=Arabidopsis_thaliana_OX=3702_GN=At1g05030_PE=2_SV=2
NODE_99489_length_860_cov_26.058450_g43621_i2	sp_Q10716_CYSP1_MAIZE_Cysteine_proteinase_1_OS=Zea_mays_OX=4577_GN=CCP1_PE=2_SV=1
BINPACKER_182_10	sp_Q10L71_VLN2_ORYSJ_Villin-2_OS=Oryza_sativa_subsp._japonica_OX=39947_GN=VLN2_PE=2_SV=1
Contig8570	sp_Q10LP5_SUS4_ORYSJ_Sucrose_synthase_4_OS=Oryza_sativa_subsp._japonica_OX=39947_GN=SUS4_PE=2_SV=1
NODE_47476_length_1735_cov_30.148014_g23807_i0	sp_Q10LQ2_CIPKA_ORYSJ_CBL-interacting_protein_kinase_10_OS=Oryza_sativa_subsp._japonica_OX=39947_GN=CIPK10_PE=2_SV=1
Contig9283	sp_Q10MI9_NAS2_ORYSJ_Nicotianamine_synthase_2_OS=Oryza_sativa_subsp._japonica_OX=39947_GN=NAS2_PE=2_SV=2

NODE_46861_length_1750_cov_13.169946_g16272_i1	sp_Q10MW3_PDC2_ORYSJ_Pyruvate_decarboxylase_2_OS=Oryza_sativa_subsp._japonica_OX=39947_GN=PDC2_PE=2_SV=1
NODE_72790_length_1222_cov_32.975631_g37477_i0	sp_Q10QE9_U496A_ORYSJ_UPF0496_protein_1_OS=Oryza_sativa_subsp._japonica_OX=39947_GN=Os03g0199100_PE=2_SV=1
NODE_43251_length_1842_cov_30.750141_g21629_i0	sp_Q10R54_SUT1_ORYSJ_Sucrose_transport_protein_SUT1_OS=Oryza_sativa_subsp._japonica_OX=39947_GN=SUT1_PE=1_SV=1
BINPACKER_23604_2	sp_Q10RB4_BGAL5_ORYSJ_Beta-galactosidase_5_OS=Oryza_sativa_subsp._japonica_OX=39947_GN=Os03g0165400_PE=2_SV=1
BINPACKER_1205_5	sp_Q14692_BMS1_HUMAN_Ribosome_biogenesis_protein_BMS1_homolog_OS=Homo_sapiens_OX=9606_GN=BMS1_PE=1_SV=1
BINPACKER_3622_5	sp_Q1JPX3_SYFA_DANRE_Phenylalanine--tRNA_ligase_alpha_subunit_OS=Danio_rerio_OX=7955_GN=farsa_PE=2_SV=2
NODE_20777_length_2701_cov_26.247336_g10351_i0	sp_Q2H102_BST1_CHAGB_GPI_inositol-deacylase_OS=Chaetomium_globosum_(strain_ATCC_6205_/_CBS_148.51_/_DSM_1962_/_NBRC_6347_/_NRRL_1970)_OX=306901_GN=BST1_PE=3_SV=2
NODE_116334_length_697_cov_38.048077_g67330_i0	sp_Q2MHE4_HT1_ARATH_Serine/threonine-protein_kinase_HT1_OS=Arabidopsis_thaliana_OX=3702_GN=HT1_PE=1_SV=1
NODE_19703_length_2767_cov_29.785820_g9823_i1	sp_Q2QMX9_ACA10_ORYSJ_Calcium-transporting_ATPase_10,_plasma_membrane-type_OS=Oryza_sativa_subsp._japonica_OX=39947_GN=ACA10_PE=2_SV=1
BINPACKER_7486_1	sp_Q2QNU0_TR130_ORYSJ_Trafficking_protein_particle_complex_II-specific_subunit_130_homolog_OS=Oryza_sativa_subsp._japonica_OX=39947_GN=TRS130_PE=2_SV=1
NODE_9547_length_3636_cov_27.134718_g4676_i2	sp_Q2R2B4_RFC1_ORYSJ_Replication_factor_C_subunit_1_OS=Oryza_sativa_subsp._japonica_OX=39947_GN=RFC1_PE=2_SV=2
NODE_19709_length_2767_cov_25.770973_g799_i2	sp_Q2R712_ACBSY_ORYSJ_Achilleol_B_synthase_OS=Oryza_sativa_subsp._japonica_OX=39947_GN=Os11g0285000_PE=1_SV=1
BINPACKER_1285_1	sp_Q2R8Z5_ADH1_ORYSJ_Alcohol_dehydrogenase_1_OS=Oryza_sativa_subsp._japonica_OX=39947_GN=ADH1_PE=2_SV=2
BINPACKER_4945_5	sp_Q2RBN7_CLH1_ORYSJ_Clathrin_heavy_chain_1_OS=Oryza_sativa_subsp._japonica_OX=39947_GN=Os11g0104900_PE=3_SV=1
BINPACKER_15835_4	sp_Q336N8_JM706_ORYSJ_Lysine-specific_demethylase_JMJ706_OS=Oryza_sativa_subsp._japonica_OX=39947_GN=JMJ706_PE=2_SV=1
NODE_36144_length_2051_cov_22.107685_g9688_i1	sp_Q38933_LCYB_ARATH_Lycopene_beta_cyclase,_chloroplastic_OS=Arabidopsis_thaliana_OX=3702_GN=LCY1_PE=1_SV=1
BINPACKER_9160_1	sp_Q39191_WAK1_ARATH_Wall-associated_receptor_kinase_1_OS=Arabidopsis_thaliana_OX=3702_GN=WAK1_PE=1_SV=2
BINPACKER_14_1	sp_Q3BAI2_YCX91_PHAAO_Uncharacterized_protein_ORF91_OS=Phalaenopsis_aphrodite_subsp._formosana_OX=308872_PE=4_SV=1
Contig3195	sp_Q3BAI2_YCX91_PHAAO_Uncharacterized_protein_ORF91_OS=Phalaenopsis_aphrodite_subsp._formosana_OX=308872_PE=4_SV=1
Contig7614	sp_Q3BAI2_YCX91_PHAAO_Uncharacterized_protein_ORF91_OS=Phalaenopsis_aphrodite_subsp._formosana_OX=308872_PE=4_SV=1
NODE_17651_length_2896_cov_22.881686_g8768_i0	sp_Q3BAI2_YCX91_PHAAO_Uncharacterized_protein_ORF91_OS=Phalaenopsis_aphrodite_subsp._formosana_OX=308872_PE=4_SV=1
NODE_8622_length_3766_cov_36.254806_g2948_i1	sp_Q3BAI2_YCX91_PHAAO_Uncharacterized_protein_ORF91_OS=Phalaenopsis_aphrodite_subsp._formosana_OX=308872_PE=4_SV=1
NODE_235_length_10039_cov_29.675597_g32_i4	sp_Q41365_PRS7_SPIOL_26S_proteasome_regulatory_subunit_7_OS=Spinacia_oleracea_OX=3562_GN=RPT1_PE=2_SV=1
NODE_71784_length_1239_cov_29.917667_g36906_i0	sp_Q41542_XTH_WHEAT_Probable_xyloglucan_endotransglucosylase/hydrolase_OS=Triticum_aestivum_OX=4565_GN=XTH_PE=2_SV=1
Contig3874	sp_Q42456_ASPR1_ORYSJ_Aspartic_proteinase_oryzasin-1_OS=Oryza_sativa_subsp._japonica_OX=39947_GN=Os05g0567100_PE=2_SV=2
NODE_100018_length_855_cov_19.060102_g55046_i0	sp_Q43247_G3PC3_MAIZE_Glyceraldehyde-3-phosphate_dehydrogenase_3,_cytosolic_OS=Zea_mays_OX=4577_GN=GAPC3_PE=2_SV=1
BINPACKER_869_4	sp_Q43697_TBB5_MAIZE_Tubulin_beta-5_chain_OS=Zea_mays_OX=4577_GN=TUBB5_PE=2_SV=1
NODE_105282_length_801_cov_29.458791_g11442_i4	sp_Q53LQ0_PDI11_ORYSJ_Protein_disulfide_isomerase-like_1-1_OS=Oryza_sativa_subsp._japonica_OX=39947_GN=PDIL1-1_PE=2_SV=1
BINPACKER_2187_1	sp_Q54IK2_TM120_DICDI_Transmembrane_protein_120_homolog_OS=Dictyostelium_discoideum_OX=44689_GN=tmem120_PE=3_SV=1
NODE_80828_length_1096_cov_23.658847_g32405_i1	sp_Q54R62_UCKC_DICDI_Uridine-cytidine_kinase_C_OS=Dictyostelium_discoideum_OX=44689_GN=udkC_PE=3_SV=1
Contig3444	sp_Q56YA5_SGAT_ARATH_Serine--glyoxylate_aminotransferase_OS=Arabidopsis_thaliana_OX=3702_GN=AGT1_PE=1_SV=2
BINPACKER_3574_3	sp_Q56YP2_PI5K1_ARATH_Phosphatidylinositol_4-phosphate_5-kinase_1_OS=Arabidopsis_thaliana_OX=3702_GN=PIP5K1_PE=1_SV=1
BINPACKER_3574_6	sp_Q56YP2_PI5K1_ARATH_Phosphatidylinositol_4-phosphate_5-kinase_1_OS=Arabidopsis_thaliana_OX=3702_GN=PIP5K1_PE=1_SV=1
BINPACKER_3209_2	sp_Q5E9D6_HDHD3_BOVIN_Haloacid_dehalogenase-like_hydrolase_domain-containing_protein_3_OS=Bos_taurus_OX=9913_GN=HDHD3_PE=2_SV=1
NODE_31028_length_2231_cov_29.982391_g1851_i3	sp_Q5F3V3_PDS5A_CHICK_Sister_chromatid_cohesion_protein_PDS5_homolog_A_OS=Gallus_gallus_OX=9031_GN=PDS5A_PE=2_SV=2
BINPACKER_15128_2	sp_Q5HZ38_GRIK2_ARATH_Serine/threonine-protein_kinase_GRIK2_OS=Arabidopsis_thaliana_OX=3702_GN=GRIK2_PE=1_SV=1
NODE_138126_length_541_cov_40.724359_g85952_i0	sp_Q5N8F2_ILL2_ORYSJ_IAA-amino_acid_hydrolase_ILR1-like_2_OS=Oryza_sativa_subsp._japonica_OX=39947_GN=ILL2_PE=2_SV=1
BINPACKER_10190_2	sp_Q5NBT9_TPR1_ORYSJ_Protein_TPR1_OS=Oryza_sativa_subsp._japonica_OX=39947_GN=TPR1_PE=1_SV=1
NODE_39372_length_1950_cov_23.713905_g1408_i4	sp_Q5NBT9_TPR1_ORYSJ_Protein_TPR1_OS=Oryza_sativa_subsp._japonica_OX=39947_GN=TPR1_PE=1_SV=1
NODE_32339_length_2185_cov_10.960701_g16179_i0	sp_Q5NE24_NSP2_MEDTR_Nodulation-signaling_pathway_2_protein_OS=Medicago_truncatula_OX=3880_GN=NSP2_PE=1_SV=1
NODE_101691_length_838_cov_34.048366_g56190_i0	sp_Q5SMM8_PHT1_ORYSJ_Putrescine_hydroxycinnamoyltransferase_1_OS=Oryza_sativa_subsp._japonica_OX=39947_GN=PHT1_PE=2_SV=1
NODE_56050_length_1540_cov_18.354465_g28268_i0	sp_Q5SMM8_PHT1_ORYSJ_Putrescine_hydroxycinnamoyltransferase_1_OS=Oryza_sativa_subsp._japonica_OX=39947_GN=PHT1_PE=2_SV=1
BINPACKER_1138_6	sp_Q5SMQ9_PIN1A_ORYSJ_Auxin_efflux_carrier_component_1a_OS=Oryza_sativa_subsp._japonica_OX=39947_GN=PIN1A_PE=2_SV=1
BINPACKER_9064_5	sp_Q5U241_PD5BB_XENLA_Sister_chromatid_cohesion_protein_PDS5_homolog_B-B_OS=Xenopus_laevis_OX=8355_GN=pds5b-b_PE=2_SV=2
NODE_2758_length_5351_cov_32.404320_g1400_i0	sp_Q5VNN5_DEF1B_ORYSJ_Peptide_deformylase_1B,_chloroplastic_OS=Oryza_sativa_subsp._japonica_OX=39947_GN=PDF1B_PE=2_SV=1
NODE_11039_length_3470_cov_28.317044_g5534_i0	sp_Q5VQ09_KINUB_ORYSJ_Kinesin-like_protein_KIN-UB_OS=Oryza_sativa_subsp._japonica_OX=39947_GN=KINUB_PE=2_SV=1
NODE_12856_length_3287_cov_24.713752_g6441_i0	sp_Q5VQ09_KINUB_ORYSJ_Kinesin-like_protein_KIN-UB_OS=Oryza_sativa_subsp._japonica_OX=39947_GN=KINUB_PE=2_SV=1
NODE_24015_length_2525_cov_30.757749_g12037_i0	sp_Q5VQ78_COB21_ORYSJ_Coatomer_subunit_beta'-1_OS=Oryza_sativa_subsp._japonica_OX=39947_GN=Os06g0143900_PE=2_SV=1
Contig4882	sp_Q5VQG4_RFS_ORYSJ_Galactinol--sucrose_galactosyltransferase_OS=Oryza_sativa_subsp._japonica_OX=39947_GN=RFS_PE=1_SV=1
NODE_7349_length_3974_cov_15.034863_g3367_i1	sp_Q5W7C6_KN5A_ORYSJ_Kinesin-like_protein_KIN-5A_OS=Oryza_sativa_subsp._japonica_OX=39947_GN=KIN5A_PE=2_SV=1
NODE_42937_length_1851_cov_23.165354_g21450_i0	sp_Q5ZLW3_DYM_CHICK_Dymeclin_OS=Gallus_gallus_OX=9031_GN=DYM_PE=2_SV=1
BINPACKER_4692_2	sp_Q620W3_EFR3_CAEBR_Protein_EFR3_homolog_OS=Caenorhabditis_briggsae_OX=6238_GN=CBG02625_PE=3_SV=1
BINPACKER_584_15	sp_Q653U3_ARFQ_ORYSJ_Auxin_response_factor_17_OS=Oryza_sativa_subsp._japonica_OX=39947_GN=ARF17_PE=2_SV=1
BINPACKER_584_16	sp_Q653U3_ARFQ_ORYSJ_Auxin_response_factor_17_OS=Oryza_sativa_subsp._japonica_OX=39947_GN=ARF17_PE=2_SV=1
BINPACKER_12202_2	sp_Q655X8_AAH_ORYSJ_Probable_allantoate_deiminase_OS=Oryza_sativa_subsp._japonica_OX=39947_GN=AAH_PE=1_SV=1
BINPACKER_12202_7	sp_Q655X8_AAH_ORYSJ_Probable_allantoate_deiminase_OS=Oryza_sativa_subsp._japonica_OX=39947_GN=AAH_PE=1_SV=1
NODE_53142_length_1602_cov_15.655984_g26729_i0	sp_Q65X71_ACA6_ORYSJ_Probable_calcium-transporting_ATPase_6,_plasma_membrane-type_OS=Oryza_sativa_subsp._japonica_OX=39947_GN=ACA6_PE=3_SV=1
NODE_9415_length_3655_cov_30.104690_g4757_i0	sp_Q65X92_C3H37_ORYSJ_Zinc_finger_CCCH_domain-containing_protein_37_OS=Oryza_sativa_subsp._japonica_OX=39947_GN=Os05g0525900_PE=2_SV=1
BINPACKER_1614_1	sp_Q67ZB3_FRL3_ARATH_FRIGIDA-like_protein_3_OS=Arabidopsis_thaliana_OX=3702_GN=FRL3_PE=1_SV=1
NODE_29978_length_2271_cov_26.885805_g15050_i1	sp_Q69Q02_DPE2_ORYSJ_4-alpha-glucanotransferase_DPE2_OS=Oryza_sativa_subsp._japonica_OX=39947_GN=DPE2_PE=2_SV=1
NODE_36257_length_2047_cov_28.080041_g18187_i0	sp_Q69TY5_SPP_ORYSJ_Stromal_processing_peptidase,_chloroplastic_OS=Oryza_sativa_subsp._japonica_OX=39947_GN=SPP_PE=2_SV=2
NODE_46908_length_1749_cov_17.919451_g23527_i0	sp_Q69UE6_AT10_ORYSJ_Acyl_transferase_10_OS=Oryza_sativa_subsp._japonica_OX=39947_GN=AT10_PE=3_SV=1
NODE_88857_length_988_cov_5.486339_g47499_i0	sp_Q69UE6_AT10_ORYSJ_Acyl_transferase_10_OS=Oryza_sativa_subsp._japonica_OX=39947_GN=AT10_PE=3_SV=1
BINPACKER_59_5	sp_Q6AT10_IAA15_ORYSJ_Auxin-responsive_protein_IAA15_OS=Oryza_sativa_subsp._japonica_OX=39947_GN=IAA15_PE=2_SV=1
BINPACKER_2511_14	sp_Q6AUG0_U2AFB_ORYSJ_Splicing_factor_U2af_small_subunit_B_OS=Oryza_sativa_subsp._japonica_OX=39947_GN=U2AF35B_PE=2_SV=1
BINPACKER_3980_3	sp_Q6AVI0_RSLE2_ORYSJ_Zinc_finger_BED_domain-containing_protein_RICESLEEPER_2_OS=Oryza_sativa_subsp._japonica_OX=39947_GN=Os03g0733400_PE=2_SV=1
Contig6666	sp_Q6CQE5_TAR1_KLULA_Protein_TAR1_OS=Kluyveromyces_lactis_(strain_ATCC_8585_/_CBS_2359_/_DSM_70799_/_NBRC_1267_/_NRRL_Y-1140_/_WM37)_OX=284590_GN=TAR1-A_PE=4_SV=2
Contig7942	sp_Q6CQE5_TAR1_KLULA_Protein_TAR1_OS=Kluyveromyces_lactis_(strain_ATCC_8585_/_CBS_2359_/_DSM_70799_/_NBRC_1267_/_NRRL_Y-1140_/_WM37)_OX=284590_GN=TAR1-A_PE=4_SV=2
NODE_9780_length_3603_cov_115.428329_g4942_i0	sp_Q6ENQ5_YCF68_SACOF_Uncharacterized_protein_ycf68_OS=Saccharum_officinarum_OX=4547_GN=ycf68-1_PE=3_SV=1
NODE_21020_length_2688_cov_33.556405_g6301_i81	sp_Q6ER94_BAS1_ORYSJ_2-Cys_peroxiredoxin_BAS1,_chloroplastic_OS=Oryza_sativa_subsp._japonica_OX=39947_GN=BAS1_PE=1_SV=1
BINPACKER_5641_6	sp_Q6EVK6_BRM_ARATH_ATP-dependent_helicase_BRM_OS=Arabidopsis_thaliana_OX=3702_GN=BRM_PE=1_SV=1
BINPACKER_7853_4	sp_Q6GQT9_NOMO1_MOUSE_Nodal_modulator_1_OS=Mus_musculus_OX=10090_GN=Nomo1_PE=1_SV=1
NODE_98777_length_868_cov_21.090566_g54202_i0	sp_Q6H8D5_COB22_ORYSJ_Coatomer_subunit_beta'-2_OS=Oryza_sativa_subsp._japonica_OX=39947_GN=Os02g0209100_PE=2_SV=1
NODE_113199_length_725_cov_34.136503_g64816_i0	sp_Q6K4V2_SWET4_ORYSJ_Bidirectional_sugar_transporter_SWEET4_OS=Oryza_sativa_subsp._japonica_OX=39947_GN=SWEET4_PE=2_SV=1
BINPACKER_14318_1	sp_Q6K8Z4_FH7_ORYSJ_Formin-like_protein_7_OS=Oryza_sativa_subsp._japonica_OX=39947_GN=FH7_PE=3_SV=2
NODE_39723_length_1939_cov_21.869775_g19908_i0	sp_Q6L4L4_SIZ1_ORYSJ_E3_SUMO-protein_ligase_SIZ1_OS=Oryza_sativa_subsp._japonica_OX=39947_GN=SIZ1_PE=1_SV=1
Contig4536	sp_Q6MRB2_TOLB_BDEBA_Tol-Pal_system_protein_TolB_OS=Bdellovibrio_bacteriovorus_(strain_ATCC_15356_/_DSM_50701_/_NCIB_9529_/_HD100)_OX=264462_GN=tolB_PE=3_SV=1
NODE_35177_length_2083_cov_33.545274_g17625_i0	sp_Q6MRB2_TOLB_BDEBA_Tol-Pal_system_protein_TolB_OS=Bdellovibrio_bacteriovorus_(strain_ATCC_15356_/_DSM_50701_/_NCIB_9529_/_HD100)_OX=264462_GN=tolB_PE=3_SV=1
BINPACKER_8299_4	sp_Q6RYA0_SABP2_TOBAC_Salicylic_acid-binding_protein_2_OS=Nicotiana_tabacum_OX=4097_GN=SABP2_PE=1_SV=1
NODE_13760_length_3198_cov_22.404800_g6913_i0	sp_Q6RYA0_SABP2_TOBAC_Salicylic_acid-binding_protein_2_OS=Nicotiana_tabacum_OX=4097_GN=SABP2_PE=1_SV=1
BINPACKER_12252_2	sp_Q6WWW4_UPL3_ARATH_E3_ubiquitin-protein_ligase_UPL3_OS=Arabidopsis_thaliana_OX=3702_GN=UPL3_PE=1_SV=1
BINPACKER_337_5	sp_Q6YSF3_AN321_ORYSJ_Acidic_leucine-rich_nuclear_phosphoprotein_32-related_protein_1_OS=Oryza_sativa_subsp._japonica_OX=39947_GN=Os07g0607800_PE=2_SV=1
BINPACKER_627_2	sp_Q6YWQ4_HAK25_ORYSJ_Potassium_transporter_25_OS=Oryza_sativa_subsp._japonica_OX=39947_GN=HAK25_PE=2_SV=1
BINPACKER_627_5	sp_Q6YWQ4_HAK25_ORYSJ_Potassium_transporter_25_OS=Oryza_sativa_subsp._japonica_OX=39947_GN=HAK25_PE=2_SV=1
NODE_145609_length_500_cov_52.327869_g92656_i0	sp_Q6YZX6_ACOC_ORYSJ_Putative_aconitate_hydratase,_cytoplasmic_OS=Oryza_sativa_subsp._japonica_OX=39947_GN=Os08g0191100_PE=3_SV=1
BINPACKER_5175_2	sp_Q6Z0E2_CCC1_ORYSJ_Cation-chloride_cotransporter_1_OS=Oryza_sativa_subsp._japonica_OX=39947_GN=CCC1_PE=2_SV=1
NODE_65343_length_1353_cov_23.025000_g17471_i2	sp_Q6Z9F4_CIPK6_ORYSJ_CBL-interacting_protein_kinase_6_OS=Oryza_sativa_subsp._japonica_OX=39947_GN=CIPK6_PE=2_SV=1
NODE_4706_length_4596_cov_23.638072_g2395_i0	sp_Q6ZFZ4_DEK1_ORYSJ_Calpain-type_cysteine_protease_ADL1_OS=Oryza_sativa_subsp._japonica_OX=39947_GN=ADL1_PE=1_SV=1
BINPACKER_18504_1	sp_Q6ZI50_NAR21_ORYSJ_High-affinity_nitrate_transporter-activating_protein_2.1_OS=Oryza_sativa_subsp._japonica_OX=39947_GN=NAR2.1_PE=1_SV=1
Contig11043	sp_Q709C8_VP13C_HUMAN_Vacuolar_protein_sorting-associated_protein_13C_OS=Homo_sapiens_OX=9606_GN=VPS13C_PE=1_SV=1
BINPACKER_7641_1	sp_Q70PP2_SMG1_DROME_Serine/threonine-protein_kinase_Smg1_OS=Drosophila_melanogaster_OX=7227_GN=nonC_PE=1_SV=2
NODE_82305_length_1075_cov_25.474052_g42727_i1	sp_Q75HE5_NAC2_ORYSJ_NAC_domain-containing_protein_2_OS=Oryza_sativa_subsp._japonica_OX=39947_GN=NAC002_PE=1_SV=1
NODE_64229_length_1374_cov_15.089931_g32617_i0	sp_Q75L84_GT43A_ORYSJ_Probable_beta-1,4-xylosyltransferase_GT43A_OS=Oryza_sativa_subsp._japonica_OX=39947_GN=GT43A_PE=2_SV=1
BINPACKER_13876_1	sp_Q75W16_AROG_ORYSJ_Phospho-2-dehydro-3-deoxyheptonate_aldolase_2,_chloroplastic_OS=Oryza_sativa_subsp._japonica_OX=39947_GN=DAHPS2_PE=2_SV=1
BINPACKER_2191_3	sp_Q76BW5_XTH8_ORYSJ_Xyloglucan_endotransglycosylase/hydrolase_protein_8_OS=Oryza_sativa_subsp._japonica_OX=39947_GN=XTH8_PE=1_SV=1
NODE_28600_length_2321_cov_27.559164_g8179_i4	sp_Q7F9I1_CLPC1_ORYSJ_Chaperone_protein_ClpC1,_chloroplastic_OS=Oryza_sativa_subsp._japonica_OX=39947_GN=CLPC1_PE=2_SV=2
Contig9002	sp_Q7LHG5_YI31B_YEAST_Transposon_Ty3-I_Gag-Pol_polyprotein_OS=Saccharomyces_cerevisiae_(strain_ATCC_204508_/_S288c)_OX=559292_GN=TY3B-I_PE=3_SV=2
BINPACKER_6475_20	sp_Q7XCL0_EXLA2_ORYSJ_Expansin-like_A2_OS=Oryza_sativa_subsp._japonica_OX=39947_GN=EXLA2_PE=2_SV=1
NODE_37534_length_2006_cov_22.611485_g18823_i0	sp_Q7XCL0_EXLA2_ORYSJ_Expansin-like_A2_OS=Oryza_sativa_subsp._japonica_OX=39947_GN=EXLA2_PE=2_SV=1
NODE_42009_length_1876_cov_30.190238_g7261_i1	sp_Q7XD99_EXEC1_ORYSJ_Protein_EXECUTER_1,_chloroplastic_OS=Oryza_sativa_subsp._japonica_OX=39947_GN=EX1_PE=2_SV=1
BINPACKER_14225_1	sp_Q7XHL3_TDC2_ORYSJ_Tryptophan_decarboxylase_2_OS=Oryza_sativa_subsp._japonica_OX=39947_GN=TDC2_PE=1_SV=1
BINPACKER_14225_7	sp_Q7XHL3_TDC2_ORYSJ_Tryptophan_decarboxylase_2_OS=Oryza_sativa_subsp._japonica_OX=39947_GN=TDC2_PE=1_SV=1
BINPACKER_7391_11	sp_Q7XJE6_MCA1_ARATH_Metacaspase-1_OS=Arabidopsis_thaliana_OX=3702_GN=AMC1_PE=1_SV=1
Contig9474	sp_Q7XLC6_HAK11_ORYSJ_Probable_potassium_transporter_11_OS=Oryza_sativa_subsp._japonica_OX=39947_GN=HAK11_PE=2_SV=4
BINPACKER_1177_8	sp_Q7XPY2_PMA1_ORYSJ_Plasma_membrane_ATPase_OS=Oryza_sativa_subsp._japonica_OX=39947_GN=Os04g0656100_PE=2_SV=1
BINPACKER_11666_3	sp_Q7XQ12_AMT11_ORYSJ_Ammonium_transporter_1_member_1_OS=Oryza_sativa_subsp._japonica_OX=39947_GN=AMT1-1_PE=2_SV=1
BINPACKER_611_2	sp_Q7XUJ5_HOX22_ORYSJ_Homeobox-leucine_zipper_protein_HOX22_OS=Oryza_sativa_subsp._japonica_OX=39947_GN=HOX22_PE=2_SV=2
NODE_42111_length_1873_cov_27.469444_g21045_i0	sp_Q7XZU0_SAC9_ARATH_Probable_phosphoinositide_phosphatase_SAC9_OS=Arabidopsis_thaliana_OX=3702_GN=SAC9_PE=1_SV=1
BINPACKER_1516_15	sp_Q7ZWL6_MAF1_XENLA_Repressor_of_RNA_polymerase_III_transcription_MAF1_homolog_OS=Xenopus_laevis_OX=8355_GN=maf1_PE=2_SV=1
BINPACKER_13620_1	sp_Q84LK0_MSH1_ARATH_DNA_mismatch_repair_protein_MSH1,_mitochondrial_OS=Arabidopsis_thaliana_OX=3702_GN=MSH1_PE=1_SV=1
NODE_39113_length_1957_cov_27.431529_g17930_i2	sp_Q84WU2_UBP13_ARATH_Ubiquitin_carboxyl-terminal_hydrolase_13_OS=Arabidopsis_thaliana_OX=3702_GN=UBP13_PE=1_SV=1
BINPACKER_12996_3	sp_Q84ZL0_FH5_ORYSJ_Formin-like_protein_5_OS=Oryza_sativa_subsp._japonica_OX=39947_GN=FH5_PE=2_SV=2
NODE_68906_length_1289_cov_30.866776_g31941_i1	sp_Q8GWC3_PR1A2_ARATH_PRA1_family_protein_A2_OS=Arabidopsis_thaliana_OX=3702_GN=PRA1A2_PE=2_SV=1
Contig11882	sp_Q8GY23_UPL1_ARATH_E3_ubiquitin-protein_ligase_UPL1_OS=Arabidopsis_thaliana_OX=3702_GN=UPL1_PE=1_SV=3
NODE_2057_length_5796_cov_30.540800_g1080_i0	sp_Q8GY23_UPL1_ARATH_E3_ubiquitin-protein_ligase_UPL1_OS=Arabidopsis_thaliana_OX=3702_GN=UPL1_PE=1_SV=3
NODE_2130_length_5751_cov_29.685981_g1105_i0	sp_Q8GY23_UPL1_ARATH_E3_ubiquitin-protein_ligase_UPL1_OS=Arabidopsis_thaliana_OX=3702_GN=UPL1_PE=1_SV=3
BINPACKER_6513_4	sp_Q8H0B2_ARAE3_ORYSJ_Probable_UDP-arabinose_4-epimerase_3_OS=Oryza_sativa_subsp._japonica_OX=39947_GN=UEL-3_PE=2_SV=1
NODE_84398_length_1046_cov_30.177801_g12630_i2	sp_Q8H5T6_LTI6A_ORYSJ_Hydrophobic_protein_LTI6A_OS=Oryza_sativa_subsp._japonica_OX=39947_GN=LTI6A_PE=2_SV=1
NODE_23859_length_2533_cov_25.785772_g5472_i1	sp_Q8L5C6_XIP1_WHEAT_Xylanase_inhibitor_protein_1_OS=Triticum_aestivum_OX=4565_GN=XIPI_PE=1_SV=2
BINPACKER_11212_2	sp_Q8L7S3_C3H24_ARATH_Zinc_finger_CCCH_domain-containing_protein_24_OS=Arabidopsis_thaliana_OX=3702_GN=At2g28450_PE=1_SV=1
NODE_67921_length_1307_cov_35.444084_g30860_i1	sp_Q8LGG8_USPAL_ARATH_Universal_stress_protein_A-like_protein_OS=Arabidopsis_thaliana_OX=3702_GN=At3g01520_PE=1_SV=2
NODE_99125_length_864_cov_23.309735_g54437_i0	sp_Q8LGG8_USPAL_ARATH_Universal_stress_protein_A-like_protein_OS=Arabidopsis_thaliana_OX=3702_GN=At3g01520_PE=1_SV=2
BINPACKER_124458_1	sp_Q8LJJ9_STAD1_ORYSJ_Stearoyl-[acyl-carrier-protein]_9-desaturase_1,_chloroplastic_OS=Oryza_sativa_subsp._japonica_OX=39947_GN=Os01g0880800_PE=2_SV=1
Contig10910	sp_Q8LLD0_NUP96_ARATH_Nuclear_pore_complex_protein_NUP96_OS=Arabidopsis_thaliana_OX=3702_GN=NUP96_PE=1_SV=1
NODE_22129_length_2626_cov_26.583627_g11047_i0	sp_Q8LPQ5_BTS_ARATH_Zinc_finger_protein_BRUTUS_OS=Arabidopsis_thaliana_OX=3702_GN=BTS_PE=1_SV=1
BINPACKER_24505_4	sp_Q8LPT9_GWD1_CITRE_Alpha-glucan_water_dikinase,_chloroplastic_OS=Citrus_reticulata_OX=85571_GN=R1_PE=2_SV=1
NODE_64849_length_1362_cov_37.215671_g2133_i1	sp_Q8R3Q2_PP6R2_MOUSE_Serine/threonine-protein_phosphatase_6_regulatory_subunit_2_OS=Mus_musculus_OX=10090_GN=Ppp6r2_PE=1_SV=1
NODE_6291_length_4175_cov_27.213067_g3186_i0	sp_Q8RVL1_DEK1_MAIZE_Calpain-type_cysteine_protease_DEK1_OS=Zea_mays_OX=4577_GN=DEK1_PE=1_SV=2
BINPACKER_5433_10	sp_Q8RWW0_ALE2_ARATH_Receptor-like_serine/threonine-protein_kinase_ALE2_OS=Arabidopsis_thaliana_OX=3702_GN=ALE2_PE=1_SV=1
BINPACKER_3965_6	sp_Q8RWY6_CLASP_ARATH_CLIP-associated_protein_OS=Arabidopsis_thaliana_OX=3702_GN=CLASP_PE=1_SV=1
NODE_3307_length_5078_cov_28.543257_g1703_i0	sp_Q8RWY6_CLASP_ARATH_CLIP-associated_protein_OS=Arabidopsis_thaliana_OX=3702_GN=CLASP_PE=1_SV=1
Contig3190	sp_Q8RX56_UNC13_ARATH_Protein_unc-13_homolog_OS=Arabidopsis_thaliana_OX=3702_GN=PATROL1_PE=2_SV=1
NODE_43689_length_1831_cov_28.917520_g4080_i2	sp_Q8RX56_UNC13_ARATH_Protein_unc-13_homolog_OS=Arabidopsis_thaliana_OX=3702_GN=PATROL1_PE=2_SV=1
NODE_44923_length_1798_cov_8.315362_g22511_i0	sp_Q8RXK7_PME41_ARATH_Probable_pectinesterase/pectinesterase_inhibitor_41_OS=Arabidopsis_thaliana_OX=3702_GN=PME41_PE=2_SV=2
NODE_10512_length_3521_cov_28.091937_g2081_i3	sp_Q8RY59_RCD1_ARATH_Inactive_poly_[ADP-ribose]_polymerase_RCD1_OS=Arabidopsis_thaliana_OX=3702_GN=RCD1_PE=1_SV=1
BINPACKER_1935_8	sp_Q8RY89_PI5K8_ARATH_Phosphatidylinositol_4-phosphate_5-kinase_8_OS=Arabidopsis_thaliana_OX=3702_GN=PIP5K8_PE=1_SV=1
NODE_77447_length_1147_cov_21.022346_g21017_i2	sp_Q8RY89_PI5K8_ARATH_Phosphatidylinositol_4-phosphate_5-kinase_8_OS=Arabidopsis_thaliana_OX=3702_GN=PIP5K8_PE=1_SV=1
BINPACKER_495_1	sp_Q8S9J0_TAUE4_ARATH_Sulfite_exporter_TauE/SafE_family_protein_4_OS=Arabidopsis_thaliana_OX=3702_GN=At2g36630_PE=2_SV=1
Contig645	sp_Q8S9J6_ASPA_ARATH_Aspartyl_protease_family_protein_At5g10770_OS=Arabidopsis_thaliana_OX=3702_GN=At5g10770_PE=2_SV=1
NODE_56107_length_1539_cov_9.309686_g28292_i0	sp_Q8TGM5_ART3_YEAST_Putative_uncharacterized_protein_ART3_OS=Saccharomyces_cerevisiae_(strain_ATCC_204508_/_S288c)_OX=559292_GN=ART3_PE=5_SV=1
NODE_8056_length_3857_cov_237.693446_g4058_i0	sp_Q8TGM7_ART2_YEAST_Putative_uncharacterized_protein_ART2_OS=Saccharomyces_cerevisiae_(strain_ATCC_204508_/_S288c)_OX=559292_GN=ART2_PE=5_SV=1
BINPACKER_11327_4	sp_Q8VCY6_UTP6_MOUSE_U3_small_nucleolar_RNA-associated_protein_6_homolog_OS=Mus_musculus_OX=10090_GN=Utp6_PE=2_SV=1
NODE_86346_length_1020_cov_31.897571_g22918_i1	sp_Q8VYD6_PP374_ARATH_Pentatricopeptide_repeat-containing_protein_At5g10690_OS=Arabidopsis_thaliana_OX=3702_GN=CBSPPR1_PE=2_SV=1
NODE_26829_length_2395_cov_26.456934_g12040_i2	sp_Q8VYL8_DTX10_ARATH_Protein_DETOXIFICATION_10_OS=Arabidopsis_thaliana_OX=3702_GN=DTX10_PE=2_SV=1
NODE_34096_length_2121_cov_27.525391_g497_i6	sp_Q8VZ80_PLT5_ARATH_Polyol_transporter_5_OS=Arabidopsis_thaliana_OX=3702_GN=PLT5_PE=1_SV=2
BINPACKER_670_1	sp_Q8VZF1_AEE7_ARATH_Acetate/butyrate--CoA_ligase_AAE7,_peroxisomal_OS=Arabidopsis_thaliana_OX=3702_GN=AAE7_PE=1_SV=1
NODE_80300_length_1104_cov_19.317168_g29241_i3	sp_Q8VZF6_EDR2L_ARATH_Protein_ENHANCED_DISEASE_RESISTANCE_2-like_OS=Arabidopsis_thaliana_OX=3702_GN=EDR2L_PE=2_SV=1
BINPACKER_2253_1	sp_Q8VZG7_TSN1_ARATH_Ribonuclease_TUDOR_1_OS=Arabidopsis_thaliana_OX=3702_GN=TSN1_PE=1_SV=1
BINPACKER_6140_1	sp_Q8VZS0_PNC2_ARATH_Peroxisomal_adenine_nucleotide_carrier_2_OS=Arabidopsis_thaliana_OX=3702_GN=PNC2_PE=1_SV=1

BINPACKER_6301_1	sp_Q8VZT0_NLAL1_ARATH_Putative_H/ACA_ribonucleoprotein_complex_subunit_1-like_protein_1_OS=Arabidopsis_thaliana_OX=3702_GN=At3g03920_PE=2_SV=1
BINPACKER_1792_1	sp_Q8W031_ZWIP5_ARATH_Zinc_finger_protein_WIP5_OS=Arabidopsis_thaliana_OX=3702_GN=WIP5_PE=2_SV=1
NODE_53928_length_1584_cov_32.868961_g20729_i1	sp_Q8W0E4_SAT1_ORYSJ_Probable_serine_acetyltransferase_1_OS=Oryza_sativa_subsp._japonica_OX=39947_GN=SAT1_PE=2_SV=1
Contig6487	sp_Q8W1L6_MFP_ORYSJ_Peroxisomal_fatty_acid_beta-oxidation_multifunctional_protein_OS=Oryza_sativa_subsp._japonica_OX=39947_GN=MFP_PE=1_SV=2
NODE_53485_length_1594_cov_23.708087_g6470_i1	sp_Q8W1Y0_SSC14_ARATH_Sister_chromatid_cohesion_1_protein_4_OS=Arabidopsis_thaliana_OX=3702_GN=SYN4_PE=2_SV=1
BINPACKER_11104_5	sp_Q93VQ0_VCL1_ARATH_Protein_VACUOLELESS1_OS=Arabidopsis_thaliana_OX=3702_GN=VCL1_PE=1_SV=1
NODE_49538_length_1687_cov_22.351921_g24844_i0	sp_Q93WV4_WRK71_ARATH_WRKY_transcription_factor_71_OS=Arabidopsis_thaliana_OX=3702_GN=WRKY71_PE=2_SV=1
BINPACKER_8843_3	sp_Q93YS6_GTE9_ARATH_Transcription_factor_GTE9_OS=Arabidopsis_thaliana_OX=3702_GN=GTE9_PE=1_SV=1
NODE_12690_length_3303_cov_21.344582_g6352_i1	sp_Q93YU8_NRG2_ARATH_Nitrate_regulatory_gene2_protein_OS=Arabidopsis_thaliana_OX=3702_GN=NRG2_PE=1_SV=1
BINPACKER_2461_5	sp_Q93YV6_2A5K_ARATH_Serine/threonine_protein_phosphatase_2A_57_kDa_regulatory_subunit_B'_kappa_isoform_OS=Arabidopsis_thaliana_OX=3702_GN=B'KAPPA_PE=2_SV=1
BINPACKER_13766_3	sp_Q941T1_P5CS2_ORYSJ_Delta-1-pyrroline-5-carboxylate_synthase_2_OS=Oryza_sativa_subsp._japonica_OX=39947_GN=P5CS2_PE=2_SV=1
Contig3130	sp_Q941T1_P5CS2_ORYSJ_Delta-1-pyrroline-5-carboxylate_synthase_2_OS=Oryza_sativa_subsp._japonica_OX=39947_GN=P5CS2_PE=2_SV=1
BINPACKER_4825_6	sp_Q94AA4_PFKA3_ARATH_ATP-dependent_6-phosphofructokinase_3_OS=Arabidopsis_thaliana_OX=3702_GN=PFK3_PE=1_SV=1
NODE_28745_length_2316_cov_23.781097_g14432_i0	sp_Q94AA4_PFKA3_ARATH_ATP-dependent_6-phosphofructokinase_3_OS=Arabidopsis_thaliana_OX=3702_GN=PFK3_PE=1_SV=1
NODE_46714_length_1753_cov_23.877976_g23430_i0	sp_Q94AA4_PFKA3_ARATH_ATP-dependent_6-phosphofructokinase_3_OS=Arabidopsis_thaliana_OX=3702_GN=PFK3_PE=1_SV=1
NODE_71321_length_1247_cov_24.484668_g33464_i1	sp_Q94AH3_NIPA4_ARATH_Probable_magnesium_transporter_NIPA4_OS=Arabidopsis_thaliana_OX=3702_GN=At1g71900_PE=2_SV=1
BINPACKER_11333_7	sp_Q94CC0_Y5994_ARATH_Uncharacterized_protein_At5g49945_OS=Arabidopsis_thaliana_OX=3702_GN=At5g49945_PE=2_SV=1
NODE_56919_length_1522_cov_26.035197_g28692_i0	sp_Q94CS9_TIP12_ORYSJ_Probable_aquaporin_TIP1-2_OS=Oryza_sativa_subsp._japonica_OX=39947_GN=TIP1-2_PE=2_SV=1
BINPACKER_20082_3	sp_Q94FB9_AB1D_ARATH_ABC_transporter_D_family_member_1_OS=Arabidopsis_thaliana_OX=3702_GN=ABCD1_PE=1_SV=1
NODE_27886_length_2349_cov_50.690685_g13995_i0	sp_Q94K49_ALP1_ARATH_Protein_ANTAGONIST_OF_LIKE_HETEROCHROMATIN_PROTEIN_1_OS=Arabidopsis_thaliana_OX=3702_GN=ALP1_PE=1_SV=1
NODE_35179_length_2083_cov_30.670149_g17626_i0	sp_Q94K49_ALP1_ARATH_Protein_ANTAGONIST_OF_LIKE_HETEROCHROMATIN_PROTEIN_1_OS=Arabidopsis_thaliana_OX=3702_GN=ALP1_PE=1_SV=1
BINPACKER_35519_1	sp_Q96262_PCAP1_ARATH_Plasma_membrane-associated_cation-binding_protein_1_OS=Arabidopsis_thaliana_OX=3702_GN=PCAP1_PE=1_SV=1
Contig3349	sp_Q96329_ACOX4_ARATH_Acyl-coenzyme_A_oxidase_4,_peroxisomal_OS=Arabidopsis_thaliana_OX=3702_GN=ACX4_PE=1_SV=1
BINPACKER_7553_1	sp_Q9ASS2_FREE1_ARATH_Protein_FREE1_OS=Arabidopsis_thaliana_OX=3702_GN=FREE1_PE=1_SV=1
BINPACKER_7553_7	sp_Q9ASS2_FREE1_ARATH_Protein_FREE1_OS=Arabidopsis_thaliana_OX=3702_GN=FREE1_PE=1_SV=1
BINPACKER_16981_1	sp_Q9AUK4_MRS2A_ORYSJ_Magnesium_transporter_MRS2-A,_chloroplastic_OS=Oryza_sativa_subsp._japonica_OX=39947_GN=MRS2-A_PE=2_SV=1
NODE_57469_length_1510_cov_29.632568_g28978_i0	sp_Q9AWS0_Y1410_ORYSJ_AP2/ERF_and_B3_domain-containing_protein_Os01g0141000_OS=Oryza_sativa_subsp._japonica_OX=39947_GN=Os01g0141000_PE=2_SV=1
NODE_8241_length_3829_cov_31.342918_g3327_i1	sp_Q9C698_FPP6_ARATH_Filament-like_plant_protein_6_OS=Arabidopsis_thaliana_OX=3702_GN=FPP6_PE=1_SV=1
NODE_26900_length_2392_cov_27.932730_g9198_i1	sp_Q9C8G5_CSCLD_ARATH_CSC1-like_protein_ERD4_OS=Arabidopsis_thaliana_OX=3702_GN=ERD4_PE=1_SV=1
NODE_53686_length_1590_cov_17.008570_g18273_i2	sp_Q9C8G9_AB1C_ARATH_ABC_transporter_C_family_member_1_OS=Arabidopsis_thaliana_OX=3702_GN=ABCC1_PE=1_SV=1
BINPACKER_18308_1	sp_Q9C9G7_MYB62_ARATH_Transcription_factor_MYB62_OS=Arabidopsis_thaliana_OX=3702_GN=MYB62_PE=2_SV=1
NODE_63707_length_1384_cov_4.981693_g32341_i0	sp_Q9C9G7_MYB62_ARATH_Transcription_factor_MYB62_OS=Arabidopsis_thaliana_OX=3702_GN=MYB62_PE=2_SV=1
NODE_45180_length_1791_cov_29.323050_g22645_i0	sp_Q9FGR9_CEP1_ARATH_KDEL-tailed_cysteine_endopeptidase_CEP1_OS=Arabidopsis_thaliana_OX=3702_GN=CEP1_PE=1_SV=1
NODE_40819_length_1908_cov_28.392916_g20430_i0	sp_Q9FI21_BBE28_ARATH_Berberine_bridge_enzyme-like_28_OS=Arabidopsis_thaliana_OX=3702_GN=At5g44440_PE=1_SV=1
NODE_36100_length_2052_cov_30.317332_g18108_i0	sp_Q9FIK0_PFKA2_ARATH_ATP-dependent_6-phosphofructokinase_2_OS=Arabidopsis_thaliana_OX=3702_GN=PFK2_PE=1_SV=1
NODE_44498_length_1808_cov_28.997695_g22281_i0	sp_Q9FJ45_GDL83_ARATH_GDSL_esterase/lipase_At5g45910_OS=Arabidopsis_thaliana_OX=3702_GN=At5g45910_PE=2_SV=1
BINPACKER_637_6	sp_Q9FJ95_DHSO_ARATH_Sorbitol_dehydrogenase_OS=Arabidopsis_thaliana_OX=3702_GN=SDH_PE=1_SV=1
BINPACKER_44392_1	sp_Q9FLK4_GSXL8_ARATH_Flavin-containing_monooxygenase_FMO_GS-OX-like_8_OS=Arabidopsis_thaliana_OX=3702_GN=At5g61290_PE=1_SV=1
BINPACKER_9271_1	sp_Q9FPT1_UBP12_ARATH_Ubiquitin_carboxyl-terminal_hydrolase_12_OS=Arabidopsis_thaliana_OX=3702_GN=UBP12_PE=1_SV=2
BINPACKER_11034_1	sp_Q9FSV7_SST_FESAR_Sucrose:sucrose_1-fructosyltransferase_OS=Festuca_arundinacea_OX=4606_GN=1-SST_PE=1_SV=1
NODE_9551_length_3635_cov_31.888827_g4825_i0	sp_Q9FW70_KN7K_ORYSJ_Kinesin-like_protein_KIN-7K,_chloroplastic_OS=Oryza_sativa_subsp._japonica_OX=39947_GN=KIN7K_PE=2_SV=2
BINPACKER_17823_1	sp_Q9FWX7_AB11B_ARATH_ABC_transporter_B_family_member_11_OS=Arabidopsis_thaliana_OX=3702_GN=ABCB11_PE=2_SV=1
BINPACKER_13772_2	sp_Q9FXI5_IQD32_ARATH_Protein_IQ-DOMAIN_32_OS=Arabidopsis_thaliana_OX=3702_GN=IQD32_PE=1_SV=3
BINPACKER_9218_2	sp_Q9LE59_GAUT1_ARATH_Polygalacturonate_4-alpha-galacturonosyltransferase_OS=Arabidopsis_thaliana_OX=3702_GN=GAUT1_PE=1_SV=1
BINPACKER_12924_2	sp_Q9LGE3_FLP1_ORYSJ_Flowering-promoting_factor_1-like_protein_1_OS=Oryza_sativa_subsp._japonica_OX=39947_GN=RAA1_PE=1_SV=1
BINPACKER_2296_5	sp_Q9LIG2_RLK6_ARATH_Receptor-like_protein_kinase_At3g21340_OS=Arabidopsis_thaliana_OX=3702_GN=At3g21340_PE=1_SV=1
NODE_39551_length_1944_cov_32.735970_g19822_i0	sp_Q9LIS3_GAE6_ARATH_UDP-glucuronate_4-epimerase_6_OS=Arabidopsis_thaliana_OX=3702_GN=GAE6_PE=1_SV=1
Contig2101	sp_Q9LKW9_NHX7_ARATH_Sodium/hydrogen_exchanger_7_OS=Arabidopsis_thaliana_OX=3702_GN=NHX7_PE=1_SV=1
NODE_48763_length_1705_cov_30.152574_g24438_i0	sp_Q9LNC5_CLO_ARATH_110_kDa_U5_small_nuclear_ribonucleoprotein_component_CLO_OS=Arabidopsis_thaliana_OX=3702_GN=CLO_PE=1_SV=1
Contig3654	sp_Q9LPL4_SKP2A_ARATH_F-box_protein_SKP2A_OS=Arabidopsis_thaliana_OX=3702_GN=SKP2A_PE=1_SV=1
BINPACKER_795_4	sp_Q9LQU5_NUD18_ARATH_Nudix_hydrolase_18,_mitochondrial_OS=Arabidopsis_thaliana_OX=3702_GN=NUDT18_PE=2_SV=1
NODE_54225_length_1577_cov_36.410239_g27333_i0	sp_Q9LSG3_GAUT8_ARATH_Galacturonosyltransferase_8_OS=Arabidopsis_thaliana_OX=3702_GN=GAUT8_PE=1_SV=1
NODE_29011_length_2306_cov_23.732199_g14561_i0	sp_Q9LUC6_C7A14_ARATH_Cytochrome_P450_72A14_OS=Arabidopsis_thaliana_OX=3702_GN=CYP72A14_PE=2_SV=1
BINPACKER_8827_4	sp_Q9LUL4_SRF7_ARATH_Protein_STRUBBELIG-RECEPTOR_FAMILY_7_OS=Arabidopsis_thaliana_OX=3702_GN=SRF7_PE=1_SV=1
NODE_2063_length_5793_cov_25.525699_g456_i2	sp_Q9LUM0_FAB1B_ARATH_1-phosphatidylinositol-3-phosphate_5-kinase_FAB1B_OS=Arabidopsis_thaliana_OX=3702_GN=FAB1B_PE=2_SV=1
NODE_148876_length_484_cov_19.729927_g95662_i0	sp_Q9LV58_MBF1C_ARATH_Multiprotein-bridging_factor_1c_OS=Arabidopsis_thaliana_OX=3702_GN=MBF1C_PE=1_SV=1
NODE_78906_length_1125_cov_27.942015_g41204_i0	sp_Q9LV60_CRR55_ARATH_Cysteine-rich_repeat_secretory_protein_55_OS=Arabidopsis_thaliana_OX=3702_GN=CRRSP55_PE=2_SV=1
NODE_80068_length_1107_cov_34.284333_g41882_i0	sp_Q9LV60_CRR55_ARATH_Cysteine-rich_repeat_secretory_protein_55_OS=Arabidopsis_thaliana_OX=3702_GN=CRRSP55_PE=2_SV=1
NODE_18274_length_2857_cov_27.122845_g9087_i0	sp_Q9LVM0_Y5830_ARATH_Probable_inactive_receptor_kinase_At5g58300_OS=Arabidopsis_thaliana_OX=3702_GN=At5g58300_PE=1_SV=1
NODE_79868_length_1110_cov_25.926712_g41768_i0	sp_Q9LXG9_PCO1_ARATH_Plant_cysteine_oxidase_1_OS=Arabidopsis_thaliana_OX=3702_GN=PCO1_PE=1_SV=1
BINPACKER_2840_5	sp_Q9LY62_GT644_ARATH_Glycosyltransferase_family_64_protein_C4_OS=Arabidopsis_thaliana_OX=3702_GN=EPC1_PE=2_SV=1
NODE_55018_length_1561_cov_27.586022_g27740_i0	sp_Q9LYS2_AB10C_ARATH_ABC_transporter_C_family_member_10_OS=Arabidopsis_thaliana_OX=3702_GN=ABCC10_PE=2_SV=2
NODE_30577_length_2248_cov_25.819310_g15329_i0	sp_Q9LYU8_AK1_ARATH_Aspartokinase_1,_chloroplastic_OS=Arabidopsis_thaliana_OX=3702_GN=AK1_PE=1_SV=1
BINPACKER_8747_1	sp_Q9LZA4_PMT7_ARATH_Probable_methyltransferase_PMT7_OS=Arabidopsis_thaliana_OX=3702_GN=At5g04060_PE=2_SV=1
NODE_41225_length_1897_cov_30.594298_g12887_i1	sp_Q9LZA6_SDP1_ARATH_Triacylglycerol_lipase_SDP1_OS=Arabidopsis_thaliana_OX=3702_GN=SDP1_PE=1_SV=1
BINPACKER_690_1	sp_Q9LZF1_HIP6_ARATH_Heavy_metal-associated_isoprenylated_plant_protein_6_OS=Arabidopsis_thaliana_OX=3702_GN=HIPP06_PE=1_SV=1
BINPACKER_10187_2	sp_Q9LZI2_UXS2_ARATH_UDP-glucuronic_acid_decarboxylase_2_OS=Arabidopsis_thaliana_OX=3702_GN=UXS2_PE=1_SV=1
BINPACKER_4614_6	sp_Q9M0E5_VPS15_ARATH_Serine/threonine-protein_kinase_VPS15_OS=Arabidopsis_thaliana_OX=3702_GN=VPS15_PE=1_SV=1
NODE_71726_length_1240_cov_26.986290_g7216_i3	sp_Q9M0E5_VPS15_ARATH_Serine/threonine-protein_kinase_VPS15_OS=Arabidopsis_thaliana_OX=3702_GN=VPS15_PE=1_SV=1
Contig8	sp_Q9M658_MOM1_ARATH_Helicase_protein_MOM1_OS=Arabidopsis_thaliana_OX=3702_GN=MOM1_PE=1_SV=1
BINPACKER_18532_2	sp_Q9M876_SYYM_ARATH_Tyrosine--tRNA_ligase,_chloroplastic/mitochondrial_OS=Arabidopsis_thaliana_OX=3702_GN=EMB2768_PE=2_SV=1
NODE_53437_length_1595_cov_27.736531_g22454_i1	sp_Q9M886_LBD41_ARATH_LOB_domain-containing_protein_41_OS=Arabidopsis_thaliana_OX=3702_GN=LBD41_PE=2_SV=1
NODE_87937_length_1000_cov_3.678533_g46913_i0	sp_Q9M886_LBD41_ARATH_LOB_domain-containing_protein_41_OS=Arabidopsis_thaliana_OX=3702_GN=LBD41_PE=2_SV=1
NODE_12121_length_3358_cov_22.675799_g6071_i0	sp_Q9M8S0_MBS1_ARATH_Protein_METHYLENE_BLUE_SENSITIVITY_1_OS=Arabidopsis_thaliana_OX=3702_GN=MBS1_PE=2_SV=1
NODE_69162_length_1285_cov_22.133663_g35349_i0	sp_Q9MBC2_MMT1_HORVU_Methionine_S-methyltransferase_OS=Hordeum_vulgare_OX=4513_GN=MMT1_PE=1_SV=1
BINPACKER_4117_8	sp_Q9S834_CLPP5_ARATH_ATP-dependent_Clp_protease_proteolytic_subunit_5,_chloroplastic_OS=Arabidopsis_thaliana_OX=3702_GN=CLPP5_PE=1_SV=1
BINPACKER_6832_1	sp_Q9S850_SUOX_ARATH_Sulfite_oxidase_OS=Arabidopsis_thaliana_OX=3702_GN=SOX_PE=1_SV=1
NODE_16415_length_2993_cov_23.076370_g7297_i2	sp_Q9S850_SUOX_ARATH_Sulfite_oxidase_OS=Arabidopsis_thaliana_OX=3702_GN=SOX_PE=1_SV=1
Contig7932	sp_Q9SBJ1_PDK_ARATH_[Pyruvate_dehydrogenase_(acetyl-transferring)]_kinase,_mitochondrial_OS=Arabidopsis_thaliana_OX=3702_GN=PDK_PE=1_SV=1
BINPACKER_7879_6	sp_Q9SDQ3_SCL1_ARATH_Scarecrow-like_protein_1_OS=Arabidopsis_thaliana_OX=3702_GN=SCL1_PE=2_SV=1
NODE_29650_length_2282_cov_33.137619_g14886_i0	sp_Q9SF47_PAH1_ARATH_Phosphatidate_phosphatase_PAH1_OS=Arabidopsis_thaliana_OX=3702_GN=PAH1_PE=1_SV=1
BINPACKER_23437_2	sp_Q9SFV2_FHA2_ARATH_FHA_domain-containing_protein_FHA2_OS=Arabidopsis_thaliana_OX=3702_GN=FHA2_PE=1_SV=1
NODE_37970_length_1992_cov_23.769151_g19045_i0	sp_Q9SGD1_ZWIP3_ARATH_Zinc_finger_protein_WIP3_OS=Arabidopsis_thaliana_OX=3702_GN=WIP3_PE=2_SV=1
BINPACKER_380_1	sp_Q9SGP2_HSL1_ARATH_Receptor-like_protein_kinase_HSL1_OS=Arabidopsis_thaliana_OX=3702_GN=HSL1_PE=2_SV=1
BINPACKER_833_3	sp_Q9SIV5_C3H19_ARATH_Zinc_finger_CCCH_domain-containing_protein_19_OS=Arabidopsis_thaliana_OX=3702_GN=NERD_PE=1_SV=3
NODE_39365_length_1950_cov_26.508791_g6383_i2	sp_Q9SIW1_BLH7_ARATH_BEL1-like_homeodomain_protein_7_OS=Arabidopsis_thaliana_OX=3702_GN=BLH7_PE=1_SV=1
BINPACKER_8504_1	sp_Q9SK66_NDUA9_ARATH_NADH_dehydrogenase_[ubiquinone]_1_alpha_subcomplex_subunit_9,_mitochondrial_OS=Arabidopsis_thaliana_OX=3702_GN=At2g20360_PE=1_SV=2
NODE_58754_length_1483_cov_24.571631_g26965_i2	sp_Q9SLX0_IMA1B_ORYSJ_Importin_subunit_alpha-1b_OS=Oryza_sativa_subsp._japonica_OX=39947_GN=Os05g0155601_PE=1_SV=2
BINPACKER_1807_8	sp_Q9SMV6_XPO1A_ARATH_Protein_EXPORTIN_1A_OS=Arabidopsis_thaliana_OX=3702_GN=XPO1_PE=1_SV=1
Contig9624	sp_Q9SMZ4_AASS_ARATH_Alpha-aminoadipic_semialdehyde_synthase_OS=Arabidopsis_thaliana_OX=3702_GN=LKR/SDH_PE=1_SV=1
NODE_44104_length_1819_cov_26.987973_g8997_i5	sp_Q9SND9_Y3028_ARATH_Uncharacterized_acetyltransferase_At3g50280_OS=Arabidopsis_thaliana_OX=3702_GN=At3g50280_PE=3_SV=1
BINPACKER_4908_5	sp_Q9SQL2_CB24_PEA_Chlorophyll_a-b_binding_protein_P4,_chloroplastic_OS=Pisum_sativum_OX=3888_GN=lhcA-P4_PE=1_SV=1
Contig10872	sp_Q9SRM3_DIOX6_ARATH_Probable_2-oxoglutarate-dependent_dioxygenase_At3g111800_OS=Arabidopsis_thaliana_OX=3702_GN=At3g11180_PE=2_SV=1
BINPACKER_20230_2	sp_Q9STN3_SPT51_ARATH_Putative_transcription_elongation_factor_SPT5_homolog_1_OS=Arabidopsis_thaliana_OX=3702_GN=At4g08350_PE=1_SV=2
Contig96	sp_Q9SX33_ALA9_ARATH_Putative_phospholipid-transporting_ATPase_9_OS=Arabidopsis_thaliana_OX=3702_GN=ALA9_PE=3_SV=1
NODE_25259_length_2465_cov_27.476171_g3153_i3	sp_Q9SY57_P2A10_ARATH_Protein_PHLOEM_PROTEIN_2-LIKE_A10_OS=Arabidopsis_thaliana_OX=3702_GN=PP2A10_PE=2_SV=1
Contig3300	sp_Q9SZ67_WRK19_ARATH_Probable_WRKY_transcription_factor_19_OS=Arabidopsis_thaliana_OX=3702_GN=WRKY19_PE=3_SV=1
NODE_11190_length_3455_cov_31.498817_g5600_i0	sp_Q9SZL8_FRS5_ARATH_Protein_FAR1-RELATED_SEQUENCE_5_OS=Arabidopsis_thaliana_OX=3702_GN=FRS5_PE=1_SV=1
NODE_12371_length_3334_cov_31.317694_g5600_i1	sp_Q9SZL8_FRS5_ARATH_Protein_FAR1-RELATED_SEQUENCE_5_OS=Arabidopsis_thaliana_OX=3702_GN=FRS5_PE=1_SV=1
BINPACKER_2716_8	sp_Q9TIC1_MATK_PHRAU_Maturase_K_OS=Phragmites_australis_OX=29695_GN=matK_PE=3_SV=1
NODE_60280_length_1450_cov_27.898330_g19063_i2	sp_Q9UNX4_WDR3_HUMAN_WD_repeat-containing_protein_3_OS=Homo_sapiens_OX=9606_GN=WDR3_PE=1_SV=1
Contig10215	sp_Q9UXR9_DNAJ_METTE_Chaperone_protein_DnaJ_OS=Methanosarcina_thermophila_OX=2210_GN=dnaJ_PE=3_SV=1
BINPACKER_2914_2	sp_Q9WX37_RBPE_NOSS1_Putative_RNA-binding_protein_RbpE_OS=Nostoc_sp._(strain_PCC_7120_/_SAG_25.82_/_UTEX_2576)_OX=103690_GN=rbpE_PE=3_SV=3
NODE_21771_length_2647_cov_22.940559_g10853_i0	sp_Q9XIH7_PLT1_ARATH_Putative_polyol_transporter_1_OS=Arabidopsis_thaliana_OX=3702_GN=PLT1_PE=3_SV=1
BINPACKER_3203_11	sp_Q9ZPS7_TMN3_ARATH_Transmembrane_9_superfamily_member_3_OS=Arabidopsis_thaliana_OX=3702_GN=TMN3_PE=2_SV=1
NODE_7179_length_4001_cov_26.627546_g3631_i0	sp_Q9ZPS7_TMN3_ARATH_Transmembrane_9_superfamily_member_3_OS=Arabidopsis_thaliana_OX=3702_GN=TMN3_PE=2_SV=1
BINPACKER_24495_3	sp_Q9ZQ31_STY13_ARATH_Serine/threonine-protein_kinase_STY13_OS=Arabidopsis_thaliana_OX=3702_GN=STY13_PE=1_SV=2
Contig4422	sp_Q9ZQ31_STY13_ARATH_Serine/threonine-protein_kinase_STY13_OS=Arabidopsis_thaliana_OX=3702_GN=STY13_PE=1_SV=2
BINPACKER_7098_2	sp_Q9ZUF4_RIPK_ARATH_Serine/threonine-protein_kinase_RIPK_OS=Arabidopsis_thaliana_OX=3702_GN=RIPK_PE=1_SV=1
NODE_35950_length_2058_cov_20.484635_g7548_i2	sp_Q9ZUF4_RIPK_ARATH_Serine/threonine-protein_kinase_RIPK_OS=Arabidopsis_thaliana_OX=3702_GN=RIPK_PE=1_SV=1
BINPACKER_1095_3	sp_Q9ZUX1_C94C1_ARATH_Cytochrome_P450_94C1_OS=Arabidopsis_thaliana_OX=3702_GN=CYP94C1_PE=1_SV=1
BINPACKER_10164_6	sp_Q9ZW96_LBD40_ARATH_LOB_domain-containing_protein_40_OS=Arabidopsis_thaliana_OX=3702_GN=LBD40_PE=2_SV=1
