# Supplementary material for: De novo transcriptome assembly and analysis of Phragmites karka, an invasive halophyte, to study the mechanism of salinity stress tolerance
Source: Sci Rep. 2020 Mar 23;10:5192. doi: 10.1038/s41598-020-61857-8 (PMC7089983; doi:10.1038/s41598-020-61857-8)
Supplement: Supplementary file 7 — Supporting Information7. [file 41598_2020_61857_MOESM7_ESM.pdf]

TableS4. List of primers used for qRT PCR

Sl No	Unigene ID	Primer Code	Forward Sequence	Reverse Sequence
1	NODE_41354_length_1894_cov_31.994509_g20670_i0	IC5	TACAGTACGAGAGCAGCTACA	CACAGAAGGATGGCAGGATAAA
2	BINPACKER_20431_2	P3	GGTCGCTATGTGGAGTTCAA	CAGTAAGTGGAAGAGACACCAG
3	NODE_80072_length_1107_cov_30.905222_g41884_i0	CAT2	CCGAAGCTAACTGAGGGAAG	CACACATTCACACGCTCAAC
4	Contig5088	STLR1	GTCCTCAACTTAGTCAGCTCTTC	GAACGGACCTGATGGTTCTATT
5	Contig7048	STLR3	CGTTTGTTGCTATGTCTCTGTTC	ATGTGCGTGTTCCAGTAGTT
6	NODE_47868_length_1727_cov_28.326481_g23850_i1	STLR7	TCACTCTCCATCCCTCGATT	CACGCCTAATCCCTTCCTATTT
7	NODE_91831_length_950_cov_21.492588_g49470_i2	STLR8	TGACCGCTTCTCTGGTTTC	GAGAGGTCCCTCCAAACTAATG
8	BINPACKER_10495_1	STLR9	CCGGATAGCCTTTGACATAGAG	GGCTCTGTAGAGGCTGAGATA
9	BINPACKER_6848_1	STLR12	CGACGAAATGCCAGGAAGAA	TGAACAGCCAGCGACTAAAC
10	BINPACKER_7001_3	STLR13	CTCTTACGTCGGTCTGACTTTG	CGCACTTTATTTGGCTCTTCAC
11	NODE_18844_length_2822_cov_22.405602_g9393_i0	STLR14	GCGGATCTTACGTGATGATGA	TAGCCGAAATAGATGTGCGTAG
12	BINPACKER_102_1	STLR15	CGGGAGATGGTCAACAAAGATA	CATCGACAAATCCTCGGTAGAA
13	BINPACKER_8843_3	STLR18	CGCTGTCCTGTACACACTTT	ACCACCATGGATTTCCCATAC
